# Assessing Global, Regional, and National Time Trends and Associated Risk Factors of the Mortality in Ischemic Heart Disease Through Global Burden of Disease 2019 Study: Population-Based Study

**DOI:** 10.2196/46821

**Published:** 2024-01-24

**Authors:** Tingting Shu, Ming Tang, Bo He, Xiaozhu Liu, Yu Han, Chang Liu, Pedro A Jose, Hongyong Wang, Qing-Wei Zhang, Chunyu Zeng

**Affiliations:** 1 Department of Cardiology Daping Hospital The Third Military Medical University (Army Medical University) Chongqing China; 2 Chongqing Key Laboratory for Hypertension Research Chongqing Cardiovascular Clinical Research Center Chongqing Institute of Cardiology Chongqing China; 3 Department of Critical Care Medicine Beijing Shijitan Hospital Capital Medical University Beijing China; 4 Department of Cardiac Surgery Daping Hospital The Third Military Medical University (Army Medical University) Chongqing China; 5 Division of Renal Diseases and Hypertension The George Washington University School of Medicine and Health Sciences Washington, DC United States; 6 Division of Gastroenterology and Hepatology, Key Laboratory of Gastroenterology and Hepatology Ministry of Health, Renji Hospital School of Medicine, Shanghai Jiao Tong University, Shanghai Institute of Digestive Disease Shanghai China; 7 Key Laboratory of Geriatric Cardiovascular and Cerebrovascular Disease Research, Ministry of Education of China Daping Hospital The Third Military Medical University (Army Medical University) Chongqing China; 8 State Key Laboratory of Trauma, Burns and Combined Injury Daping Hospital The Third Military Medical University (Army Medical University) Chongqing China; 9 Cardiovascular Research Center of Chongqing College Chinese Academy of Sciences University of Chinese Academy of Sciences Chongqing China

**Keywords:** age-period-cohort analysis, GBD 2019, Global Burden of Disease 2019 study, ischemic heart disease, mortality, risk factors

## Abstract

**Background:**

Ischemic heart disease (IHD) is the leading cause of death among noncommunicable diseases worldwide, but data on current epidemiological patterns and associated risk factors are lacking.

**Objective:**

This study assessed the global, regional, and national trends in IHD mortality and attributable risks since 1990.

**Methods:**

Mortality data were obtained from the Global Burden of Disease 2019 Study. We used an age-period-cohort model to calculate longitudinal age curves (expected longitudinal age-specific rate), net drift (overall annual percentage change), and local drift (annual percentage change in each age group) from 15 to >95 years of age and estimate cohort and period effects between 1990 and 2019. Deaths from IHD attributable to each risk factor were estimated on the basis of risk exposure, relative risks, and theoretical minimum risk exposure level.

**Results:**

IHD is the leading cause of death in noncommunicable disease–related mortality (118.1/598.8, 19.7%). However, the age-standardized mortality rate for IHD decreased by 30.8% (95% CI –34.83% to –27.17%) over the past 30 years, and its net drift ranged from –2.89% (95% CI –3.07% to –2.71%) in high sociodemographic index (SDI) region to –0.24% (95% CI –0.32% to –0.16%) in low-middle–SDI region. The greatest decrease in IHD mortality occurred in the Republic of Korea (high SDI) with net drift –6.06% (95% CI –6.23% to –5.88%), followed by 5 high-SDI nations (Denmark, Norway, Estonia, the Netherlands, and Ireland) and 2 high-middle–SDI nations (Israel and Bahrain) with net drift less than –5.00%. Globally, age groups of >60 years continued to have the largest proportion of IHD-related mortality, with slightly higher mortality in male than female group. For period and birth cohort effects, the trend of rate ratios for IHD mortality declined across successive period groups from 2000 to 2004 and birth cohort groups from 1985 to 2000, with noticeable improvements in high-SDI regions. In low-SDI regions, IHD mortality significantly declined in female group but fluctuated in male group across successive periods; sex differences were greater in those born after 1945 in middle- and low-middle–SDI regions and after 1970 in low-SDI regions. Metabolic risks were the leading cause of mortality from IHD worldwide in 2019. Moreover, smoking, particulate matter pollution, and dietary risks were also important risk factors, increasingly occurring at a younger age. Diets low in whole grains and legumes were prominent dietary risks in both male and female groups, and smoking and high-sodium diet mainly affect male group.

**Conclusions:**

IHD, a major concern, needs focused health care attention, especially for older male individuals and those in low-SDI regions. Metabolic risks should be prioritized for prevention, and behavioral and environmental risks should attract more attention to decrease IHD mortality.

## Introduction

Ischemic heart disease (IHD) is the leading cause of death among noncommunicable diseases (NCDs) and cardiovascular diseases (CVDs) in the world, accounting for 9.14 million deaths in 2019 [1,2]. The United Nations set, as part of the Sustainable Development Goals, a global target to reduce by one-third the total premature mortality (ages 30-70 years) from NCDs by 2030 [3]. However, the trend and distribution of IHD-related mortality vary greatly in countries from high sociodemographic index (SDI) to low SDI [4]. In Central Asia, the age-standardized mortality rate (ASMR) increased by 16.7% from 1990 to 2017 [5]. By contrast, in Korea, Japan, and China, the ASMR decreased significantly in both male and female groups during this time period [6,7].

Rapid economic development and social advancements have greatly increased the incidence of metabolic risk factors, such as hypertension, hyperlipidemia, diabetes, obesity, and aging, which have largely contributed to the majority of IHD-related deaths shifting from developed to developing countries [2,8]. However, the relative contribution of risk factors to mortality from IHD varies with age, sex, metabolic state, behavior, geographical area, and environmental exposure, and therefore different interventions may be required to effectively address these risk factors [9].

Previous studies have also explored IHD epidemic trends over time in many countries [2,10]. However, typical statistical analysis cannot break down the risks when estimating mortality. Therefore, the age-period-cohort (APC) model was developed to improve the traditional method for mortality description and analysis, which separates age effects, period effects, and cohort effects and quantifies the influence of age, time, and birth cohort factors on mortality rate [11,12]. In this study, we used data from the Global Burden of Disease 2019 Study (GBD) to assess the time trends in IHD mortality at the global, regional, and national levels and associations with age, period, and birth cohort using the APC model over the past 30 years. We also aimed to shed light on attributable risk factors for IHD at global, regional, and national levels to support policy makers making informed decisions about the potential benefits of risk reduction policies.

## Methods

### Data Sources

GBD, coordinated by the Institute for Health Metrics and Evaluation, which assessed the global burden of 369 diseases and injuries and 87 risk factors in 204 countries and territories from 1990 to 2019, provided a unique opportunity to understand the global burden and landscape of IHD mortality [13,14]. In our study, we obtained the publication estimates of deaths for IHD with “ischemic heart disease” from the “cause blank” of the GBD website across 204 countries and territories [2,13]. The statistical code used for GBD estimation is publicly available on the internet [15]. The GBD study uses deidentified data, and a waiver of informed consent was reviewed and approved by the University of Washington Institutional Review Board. All results and original data sources used to compute estimates are publicly available on the internet [16].

SDI was used to quantify the development level for each location year, which is scaled from 0 to 1, with higher values indicating high SDI levels. GBD assigned 204 countries and territories into 5 groups by SDI quintiles: low SDI, low-middle SDI, middle SDI, high-middle SDI, and high SDI [13,14].

### Statistical Analyses

The overall temporal trends in mortality over the study period were assessed by all-age mortality rate (AAMR), ASMR, percentage of mortality, and the relative change in percentage between 1990 and 2019. We calculated ASMR with global age-standard population data from GBD [14].

APC models were developed to determine independent effect estimates of age, period, and birth cohort on IHD mortality [17]. Net drift and local drift are important parameters in the APC models. Net drift represents the overall log-linear trend by period and birth cohort, which indicates the overall annual percentage change of the expected age-adjusted rates over time. Local drift represents the log-linear trend by period and cohort for each age group, which indicates the annual percentage change. The longitudinal age curve indicates the expected age-specific rate in a reference cohort adjusted for period effects, which was assigned to 19 age groups using successive 5-year age intervals from 15-19 years to over 95 years among individuals with IHD. The age effects represent a differing risk of the outcome associated with different age brackets. The period effects represent variations in the outcome over time that influence all age groups simultaneously. The mortality and population data were arranged into consecutive 5-year periods from 1990 to 2019, with the mid-year of the 2000-2004 survey year as the reference period group [18]. Cohort effects are the ratio of age-specific rates of mortality risks, which were arranged into consecutive 5-year cohorts from the 1895 cohort to the 2000 cohort, as referenced by the mid-year of birth (1945 cohort) [18]. All analyses were conducted with 3 sex groups: the male group, the female group, and both groups (male and female).

A total of 87 risk factors associated with IHD-related death were selected in accordance with the World Cancer Research Fund grades of convincing or probable evidence [19,20]. To estimate the IHD-related deaths attributable to a specific risk, these IHD-related deaths were multiplied by the population attributable fractions (PAF) for the IHD risk-outcome pair for a given sex, age, year, and location. PAF show that the proportion by which the deaths would decrease in a specific year if the exposure to a risk factor in the past was equal to the theoretical minimum risk exposure level, which were adjusted by other risk factors included in this study [21,22].

We obtained the estimated parameters from the APC web tool (freely available R tools) provided by the National Cancer Institute of the United States, the methodological details of which have been described previously [23]. We hypothesized that changes in age, period, and birth cohort would mainly affect IHD mortality. The Joinpoint Trend Analysis Software (version 4.9; National Cancer Institute) was used to determine the average annual percentage change in IHD mortality from 1990 to 2019 [24]. Wald chi-square tests were adapted for the significance of the estimable parameters and functions. Statistical tests were 2-sided, and a *P* value <.05 was considered significant. We performed all the analysis in R software (version 3.6.3; R Foundation for Statistical Computing).

### Ethical Considerations

Ethics approval and consent are not required because the original data in this study were aggregated and nonidentified, which are freely available from the GBD Study.

## Results

### Overview and Trends for IHD Mortality

**Figure 1:**
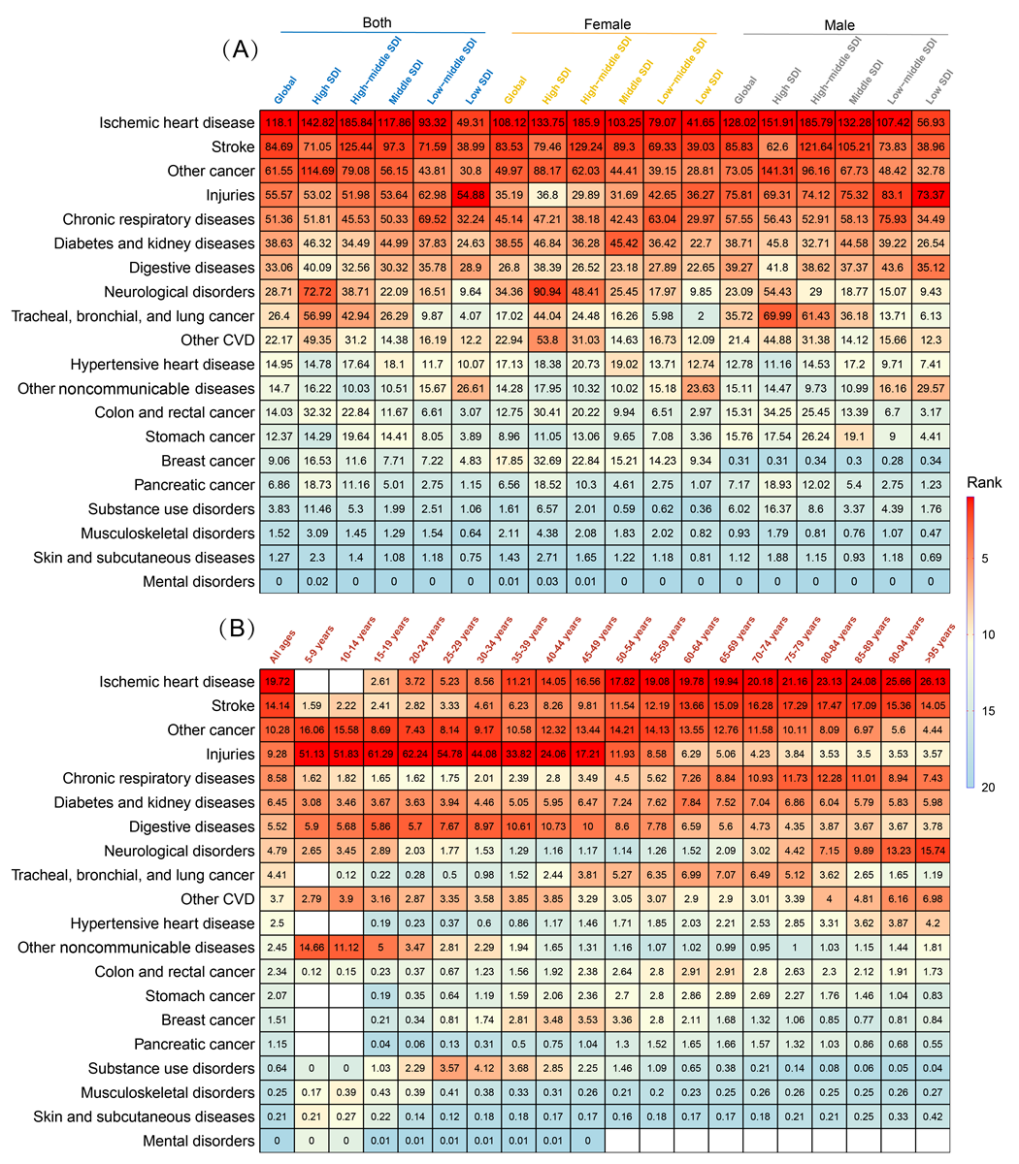
The leading cause of global mortality from noncommunicable diseases (NCDs) in the all-age population. (A) All-age mortality rate in 2019 for NCDs in female and male, individually, and as a group. (B) The proportion of the mortality rate in 2019 for NCDs among age groups. CVD: cardiovascular disease; SDI: sociodemographic index.

In 2019, IHD was the leading cause of NCD-related AAMR in the global and each SDI quintile, accounting for 118.10 deaths per 10,000 (95% UI [uncertainty interval] 125.93-108.51) population worldwide (Table 1 and Figure 1A). IHD-related AAMRs were lower in low-SDI quintiles and about 35% higher in male group than female group. IHD had the largest proportion of NCD-related mortality with 19.7% (118.1/598.8) in all ages and maintained this dominant proportion in the >50-year-old age group (Figure 1B).

Globally, the number of mortalities for IHD increased by 60.43% from 1990 to 2019, and the ASMR for IHD decreased by 30.8%, with marked reduction in high-SDI (–58.68%) and high-middle–SDI (–35.28%) regions (Table 2). However, ASMR for IHD increased in 4 lower-income GBD regions, with a percentage change of 15.75% in East Asia, 11.71% in Central Asia, 8.31% in Oceania, and 4.63% in Southern Sub-Saharan Africa (Table 2).

Over the past 30 years (1990-2019), the IHD mortality rate increased in the older age groups (>60 years), but the increase declined in later periods that occurred mainly in the high-SDI regions (Figure S1 in Multimedia Appendix 1). There was a relatively declining risk of IHD mortality in cohorts born before 1945 in high-SDI regions, but the risk remained high in the low-SDI regions (Figure S1 in Multimedia Appendix 1). Similar to the trend by global and 5 SDI quintiles, IHD ASMRs across nations increased with age and declined between 1990-1994 and 2015-2019 (Figures S1A-S1F in Multimedia Appendix 1). There was a downward trend of mortality for IHD in high-SDI countries across successive birth cohorts, but it fluctuated and trended to increase with aging in low-SDI countries (Figures S2A-S2F in Multimedia Appendix 1).

**Figure 2:**
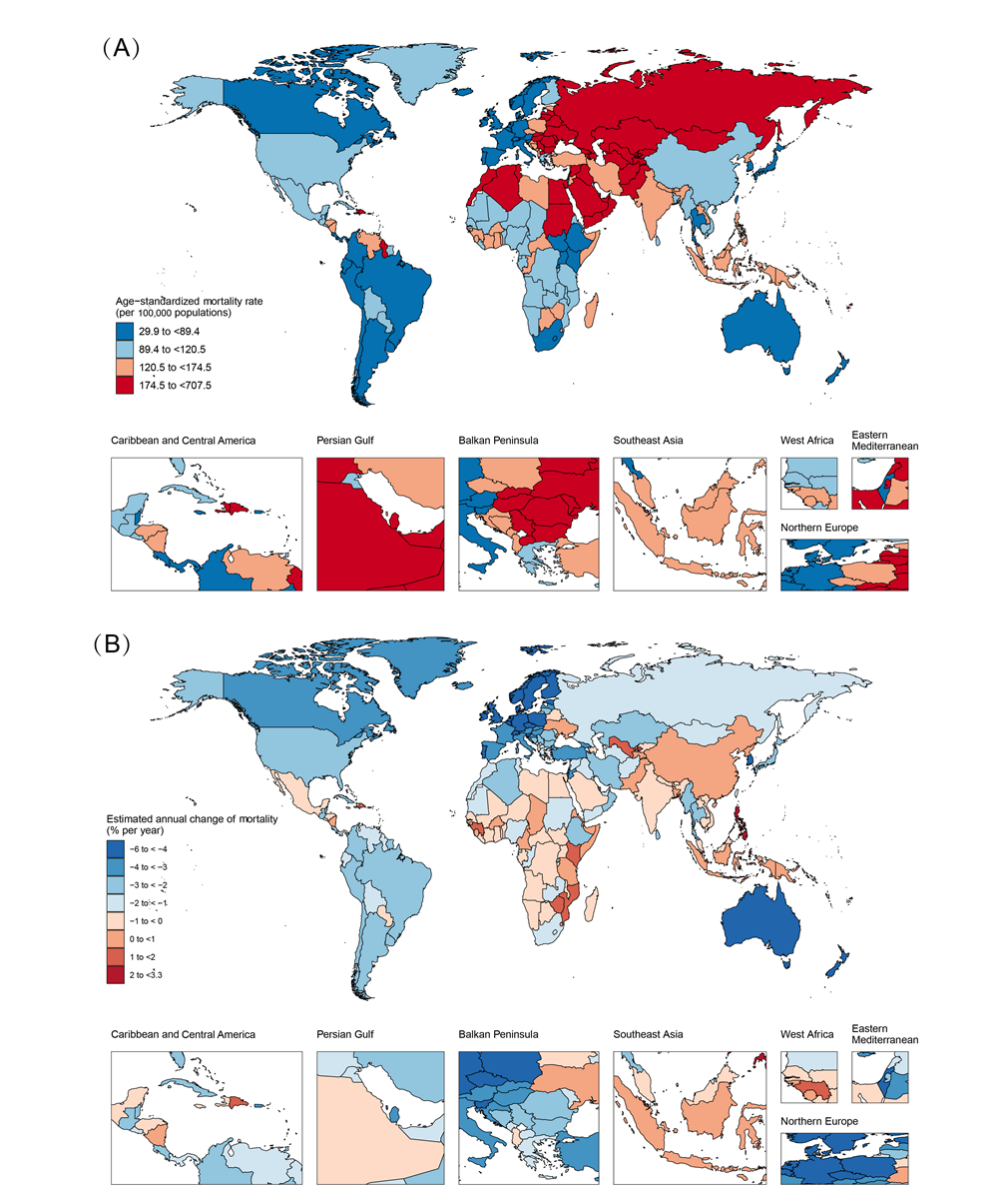
The age-standardized mortality in 2019 and net drift of mortality during 1990-2019 for ischemic heart disease in 204 countries and territories. (A) World map of age-standardized mortality for ischemic heart disease in 2019. (B) World map of net drifts for ischemic heart disease mortality, that is, estimated annual percentage change of mortality from the age-period-cohort model. Net drift captures components of the trends attributable to calendar time and successive birth cohorts.

Among 204 countries and territories in 2019, ASMRs for IHD in 107 countries were higher than the global average, 32 of which were more than 2-fold higher (Figures 2A and 2B and Table S1 in Multimedia Appendix 2). The highest ASMR for IHD was observed in Uzbekistan (707.51, 95% UI 638.23-780.68 per 100,000 population; middle SDI) with the largest increase of 119.01% (95% UI 96.32%-143.33%) over 30 years, followed by Azerbaijan (middle SDI) with 35.76% (95% UI 19.27%-54.74%) increase, and Tajikistan (low-middle SDI), with 82.1% (95% UI 53.66%-116.8%) increase. However, the net drift increased in Uzbekistan (1.98%, 95% CI 1.8%-2.17%), decreased in Azerbaijan (–0.95%, 95% CI –1.23% to –0.68%), and was essentially unchanged in Tajikistan (0.52%, 95% CI 0.13%-0.9%). In Qatar (high SDI), deaths related to IHD increased by 203.32% (95% CI 126.08%-308.55%), with a net drift of –3.69% (95% CI –4.28% to –3.09%). In Guam (high SDI) and Liberia (low SDI), ASMR in IHD decreased by 14.32% (95% CI –29.31% to 2.29%) and 14.04% (95% CI –33.47% to 10.41%), but the net drifts in mortality were 0.27% (95% CI –0.88% to 1.44%) and –0.36% (95% CI –0.77% to 0.05%), respectively. China and India, the 2 most populous countries, had the largest number of deaths with 1.87 (95% CI 1.61-2.13) million and 1.52 (95% CI 1.31-1.75) million, respectively, with relatively modest change of net drifts, 0.13% in China and –0.48% in India. These results show that IHD mortality trends were uneven across countries and not strictly commensurate with SDI levels. The directions of change in IHD mortality indicated by the traditional trend analysis were not entirely consistent with net drift from the APC model, suggesting the necessity to differentiate the relative contributions of period and cohort effects in IHD mortality.

**Table 1:** Trends of death cases and all-age mortality rate (AAMR) in ischemic heart disease across 5 sociodemographic index (SDI) quintiles and 21 regions in the Global Burden of Disease 2019 Study from 1990 to 2019.

Location	Number of death cases in 1990 (95% UI^a^)	Number of death cases in 2019 (95% UI)	Percentage change of cases (95% UI)	AAMR in 1990 per 100,000 population (95% UI)	AAMR in 2019 per 100,000 population (95% UI)	Percentage change of AAMR (95% UI)
Global	5,695,890 (5,405,191 to 5,895,398)	9,137,791 (8,395,682 to 9,743,550)	60.43 (50.23 to 69.14)	106.47 (101.03 to 110.2)	118.1 (108.51 to 125.93)	10.92 (3.88 to 16.94)
High SDI	1,688,791 (1,572,959 to 1,744,989)	1,447,271 (1,270,022 to 1,553,837)	–14.3 (–19.35 to –9.59)	205.45 (191.35 to 212.28)	142.82 (125.32 to 153.33)	–30.49 (–34.58 to –26.67)
High-middle SDI	1,870,951 (1,782,685 to 1,923,524)	2,658,293 (2,411,867 to 2,832,480)	42.08 (32.95 to 50.53)	162.63 (154.96 to 167.2)	185.84 (168.61 to 198.02)	14.27 (6.92 to 21.07)
Middle SDI	1,151,131 (1,087,976 to 1,217,989)	2,824,552 (2,576,474 to 3,047,018)	145.37 (122.94 to 167)	67.05 (63.37 to 70.95)	117.86 (107.51 to 127.14)	75.77 (59.7 to 91.26)
Low-middle SDI	712,691 (654,082 to 773,458)	1,646,065 (1,488,073 to 1,801,773)	130.96 (104.7 to 156.95)	63.09 (57.9 to 68.47)	93.32 (84.36 to 102.14)	47.91 (31.09 to 64.55)
Low SDI	269,141 (240,679 to 301,919)	556,602 (495,167 to 627,058)	106.81 (78.43 to 134.17)	50.96 (45.57 to 57.17)	49.31 (43.87 to 55.56)	–3.23 (–16.51 to 9.58)
High-income Asia Pacific	149,217 (139,101 to 155,395)	172,964 (139,728 to 191,814)	15.91 (0.45 to 25.23)	86 (80.17 to 89.56)	92.35 (74.6 to 102.42)	7.39 (–6.94 to 16.01)
High-income North America	655,487 (606,259 to 680,515)	606,489 (539,683 to 647,145)	–7.48 (–11.06 to –3.11)	233.33 (215.81 to 242.24)	166.36 (148.04 to 177.51)	–28.7 (–31.46 to –25.34)
Western Europe	916,253 (860,097 to 945,855)	666,760 (586,750 to 718,140)	–27.23 (–32.04 to –22.79)	238.24 (223.64 to 245.94)	152.82 (134.48 to 164.6)	–35.86 (–40.1 to –31.94)
Australasia	40,271 (37,696 to 41,643)	33,125 (28,421 to 35,760)	–17.74 (–24.2 to –13.33)	198.6 (185.9 to 205.36)	113.97 (97.79 to 123.04)	–42.61 (–47.12 to –39.53)
Andean Latin America	19,641 (17,398 to 21,868)	34,446 (28,404 to 40,971)	75.37 (41.25 to 114.39)	51.45 (45.57 to 57.28)	54.16 (44.66 to 64.42)	5.28 (–15.21 to 28.7)
Tropical Latin America	119,468 (113,614 to 123,476)	175,999 (160,324 to 185,401)	47.32 (39.13 to 54.49)	78.14 (74.32 to 80.77)	78.71 (71.7 to 82.92)	0.73 (–4.87 to 5.63)
Central Latin America	92,607 (87,113 to 96,026)	219,022 (189,141 to 250,607)	136.51 (109.03 to 167.84)	56.43 (53.08 to 58.51)	87.6 (75.65 to 100.23)	55.25 (37.22 to 75.82)
Southern Latin America	66,488 (62,693 to 68,727)	61,452 (55,939 to 65,419)	–7.58 (–12.51 to –2.8)	134.21 (126.55 to 138.72)	92.06 (83.8 to 98)	–31.41 (–35.07 to –27.86)
Caribbean	44,030 (41,268 to 46,039)	63,535 (55,083 to 72,649)	44.3 (27.09 to 62.53)	124.82 (116.99 to 130.52)	134.7 (116.78 to 154.03)	7.91 (–4.95 to 21.55)
Central Europe	389,917 (371,996 to 398,754)	354,125 (308,352 to 395,305)	–9.18 (–18.94 to 0.39)	317.09 (302.52 to 324.28)	310.03 (269.95 to 346.08)	–2.23 (–12.74 to 8.07)
Eastern Europe	796,168 (762,557 to 815,230)	986,560 (879,471 to 1,075,171)	23.91 (13.8 to 34.01)	351.5 (336.66 to 359.92)	469.86 (418.85 to 512.06)	33.67 (22.76 to 44.56)
Central Asia	131,655 (124,727 to 135,840)	200,135 (183,535 to 218,271)	52.01 (40.02 to 65.82)	190.07 (180.07 to 196.11)	213.98 (196.23 to 233.37)	12.58 (3.69 to 22.8)
North Africa and Middle East	444,690 (411,688 to 478,245)	799,484 (706,349 to 909,787)	79.78 (58.25 to 100.46)	128.89 (119.32 to 138.61)	131.34 (116.04 to 149.46)	1.9 (–10.3 to 13.62)
South Asia	754,477 (675,262 to 830,037)	1,857,949 (1,633,946 to 2,091,633)	146.26 (106.82 to 183.81)	68.74 (61.52 to 75.62)	102.92 (90.51 to 115.87)	49.73 (25.75 to 72.57)
Southeast Asia	248,618 (226,364 to 270,439)	588,556 (527,652 to 644,283)	136.73 (107.34 to 167.98)	53.26 (48.49 to 57.94)	87.35 (78.31 to 95.62)	64.01 (43.64 to 85.66)
East Asia	631,081 (559,209 to 703,062)	1,926,477 (1,662,056 to 2,182,277)	205.27 (158.4 to 261.21)	51.51 (45.64 to 57.39)	130.86 (112.9 to 148.23)	154.04 (115.04 to 200.59)
Oceania	4,771 (3,879 to 6,050)	12,296 (9,869 to 15,450)	157.72 (112.98 to 215.56)	73.75 (59.95 to 93.51)	92.62 (74.33 to 116.37)	25.59 (3.79 to 53.78)
Western Sub-Saharan Africa	87,324 (72,430 to 108,234)	166,504 (139,342 to 195,399)	90.67 (45.29 to 128.5)	45.34 (37.61 to 56.2)	36.49 (30.54 to 42.82)	–19.53 (–38.68 to –3.56)
Eastern Sub-Saharan Africa	59,328 (51,116 to 66,823)	120,310 (96,907 to 143,565)	102.79 (52.77 to 151.07)	31.2 (26.88 to 35.14)	29.22 (23.53 to 34.86)	–6.35 (–29.45 to 15.95)
Central Sub-Saharan Africa	22,977 (19,148 to 28,107)	47,752 (36,504 to 62,352)	107.83 (66.12 to 162.71)	41.38 (34.49 to 50.63)	36.3 (27.75 to 47.4)	–12.28 (–29.89 to 10.88)
Southern Sub-Saharan Africa	21,423 (19,366 to 23,428)	43,851 (39,702 to 48,020)	104.69 (86.5 to 125.45)	40.81 (36.89 to 44.63)	55.81 (50.53 to 61.11)	36.75 (24.59 to 50.62)

^a^UI: uncertainty interval.

**Table 2:** Trends of age-standardized mortality rate (ASMR) and net drift in ischemic heart disease across 5 socio-demographic index (SDI) quintiles and 21 regions of the Global Burden of Disease 2019 Study from 1990 to 2019.

Location	ASMR in 1990 per 100,000 population (95% UI^a^)	ASMR in 2019 per 100,000 population (95% UI)	Percentage change of ASMR (95% UI)	AAPC^b^, % (95% CI)	Net drift, % (95% CI)
Global	170.45 (159.61 to 176.94)	117.95 (107.83 to 125.92)	–30.8 (–34.83 to –27.17)	–1.26 (–1.38 to –1.13)	–1.15 (–1.21 to –1.09)
High SDI	162.39 (150.62 to 168.15)	67.1 (60.07 to 71.54)	–58.68 (–60.3 to –56.69)	–3.08 (–3.19 to –2.97)	–2.89 (–3.07 to –2.71)
High-middle SDI	209.2 (196.25 to 216.23)	135.41 (122.68 to 144.44)	–35.28 (–39.01 to –31.69)	–1.56 (–1.77 to –1.35)	–2 (–2.17 to –1.83)
Middle SDI	143.11 (133.18 to 152.13)	134.12 (121.51 to 145.22)	–6.28 (–14.41 to 1.84)	–0.22 (–0.35 to –0.1)	–0.31 (–0.37 to –0.25)
Low-middle SDI	144.21 (132.05 to 156.58)	136.59 (122.96 to 149.5)	–5.28 (–15.25 to 5.09)	–0.14 (–0.19 to –0.08)	–0.24 (–0.32 to –0.16)
Low SDI	139.2 (124 to 156.79)	127.99 (113.13 to 143.9)	–8.05 (–20.59 to 3.17)	–0.29 (–0.41 to –0.17)	–0.43 (–0.5 to –0.37)
High-income Asia Pacific	85.48 (78.44 to 89.52)	30.47 (25.73 to 33.34)	–64.36 (–67.28 to –62.35)	–3.51 (–3.77 to –3.25)	–3.46 (–3.73 to –3.19)
High-income North America	179.79 (166.53 to 186.54)	88.09 (79.57 to 93.4)	–51.01 (–52.49 to –48.8)	–2.47 (–2.59 to –2.35)	–2.4 (–2.57 to –2.24)
Western Europe	156.31 (146.05 to 161.8)	60.69 (54.18 to 64.89)	–61.17 (–62.94 to –59.05)	–3.27 (–3.39 to –3.14)	–3.92 (–4.2 to –3.64)
Australasia	178.69 (165.54 to 185.48)	59.04 (51.31 to 63.44)	–66.96 (–68.94 to –65.45)	–3.79 (–3.95 to –3.62)	–4.32 (–4.66 to –3.97)
Andean Latin America	109.84 (97.27 to 121.84)	64.48 (52.96 to 76.52)	–41.29 (–52.35 to –28.5)	–1.81 (–1.98 to –1.63)	–1.98 (–2.07 to –1.88)
Tropical Latin America	156.35 (145.4 to 162.47)	75.23 (68.22 to 79.42)	–51.88 (–54.21 to –49.69)	–2.48 (–2.57 to –2.4)	–2.18 (–2.27 to –2.1)
Central Latin America	131.13 (121.2 to 136.69)	97.37 (83.84 to 111.34)	–25.74 (–33.94 to –16.03)	–1.12 (–1.35 to –0.88)	–1.09 (–1.19 to –0.99)
Southern Latin America	161.14 (150.08 to 167.35)	72.05 (65.77 to 76.66)	–55.29 (–57.27 to –53.1)	–2.76 (–2.96 to –2.56)	–2.74 (–2.95 to –2.53)
Caribbean	185.06 (172 to 193.88)	122.12 (106.01 to 139.69)	–34.01 (–41.86 to –25.76)	–1.4 (–1.64 to –1.16)	–1.16 (–1.25 to –1.07)
Central Europe	300.44 (283.07 to 308.93)	159.8 (139.02 to 178.42)	–46.81 (–52.44 to –41.39)	–2.19 (–2.43 to –1.96)	–3.34 (–3.59 to –3.09)
Eastern Europe	326.57 (308.96 to 336.05)	284.55 (253.63 to 310.14)	–12.86 (–19.52 to –6.08)	–0.6 (–1.04 to –0.15)	–1.19 (–1.49 to –0.9)
Central Asia	322.28 (301.66 to 333.6)	360.02 (330.9 to 390.24)	11.71 (3.8 to 20.22)	0.41 (0.22 to 0.6)	–0.62 (–0.76 to –0.48)
North Africa and Middle East	309.32 (284.33 to 332.13)	219.01 (194.15 to 246.75)	–29.2 (–36.92 to –21.85)	–1.19 (–1.31 to –1.07)	–1.71 (–1.77 to –1.64)
South Asia	159.72 (143 to 177.5)	149.21 (130.6 to 167.88)	–6.59 (–21.1 to 6.7)	–0.25 (–0.35 to –0.14)	–0.27 (–0.39 to –0.16)
Southeast Asia	117.63 (106.32 to 128.2)	112.63 (100.54 to 122.88)	–4.26 (–16.26 to 7.52)	–0.13 (–0.18 to –0.08)	–0.06 (–0.1 to –0.03)
East Asia	98.76 (88.2 to 109.52)	114.31 (98.93 to 128.75)	15.75 (–0.97 to 36.12)	0.52 (0.17 to 0.86)	0.12 (–0.04 to 0.28)
Oceania	183.67 (150.74 to 228.78)	198.93 (162.3 to 246.64)	8.31 (–8.61 to 29.3)	0.26 (0.19 to 0.34)	0.33 (0.18 to 0.48)
Western Sub-Saharan Africa	126.66 (106.08 to 154.95)	114.61 (95.9 to 132.21)	–9.51 (–31.88 to 7.22)	–0.27 (–0.39 to –0.15)	–0.54 (–0.61 to –0.47)
Eastern Sub-Saharan Africa	99.72 (86.19 to 111.65)	93.65 (74.72 to 111.98)	–6.09 (–27.99 to 13.35)	–0.26 (–0.31 to –0.22)	–0.64 (–0.69 to –0.6)
Central Sub-Saharan Africa	130.41 (108.75 to 160.36)	116.78 (88.97 to 150.45)	–10.45 (–27.43 to 10.48)	–0.38 (–0.53 to –0.23)	–0.66 (–0.74 to –0.58)
Southern Sub-Saharan Africa	89.43 (80.06 to 97.89)	93.57 (84.43 to 102.05)	4.63 (–4.08 to 14.8)	0.45 (0.13 to 0.78)	–0.64 (–0.85 to –0.43)

^a^UI: uncertainty interval.

^b^AAPC: average annual percentage change.

### Time Trends for IHD Mortality in APC Test

**Figure 3:**
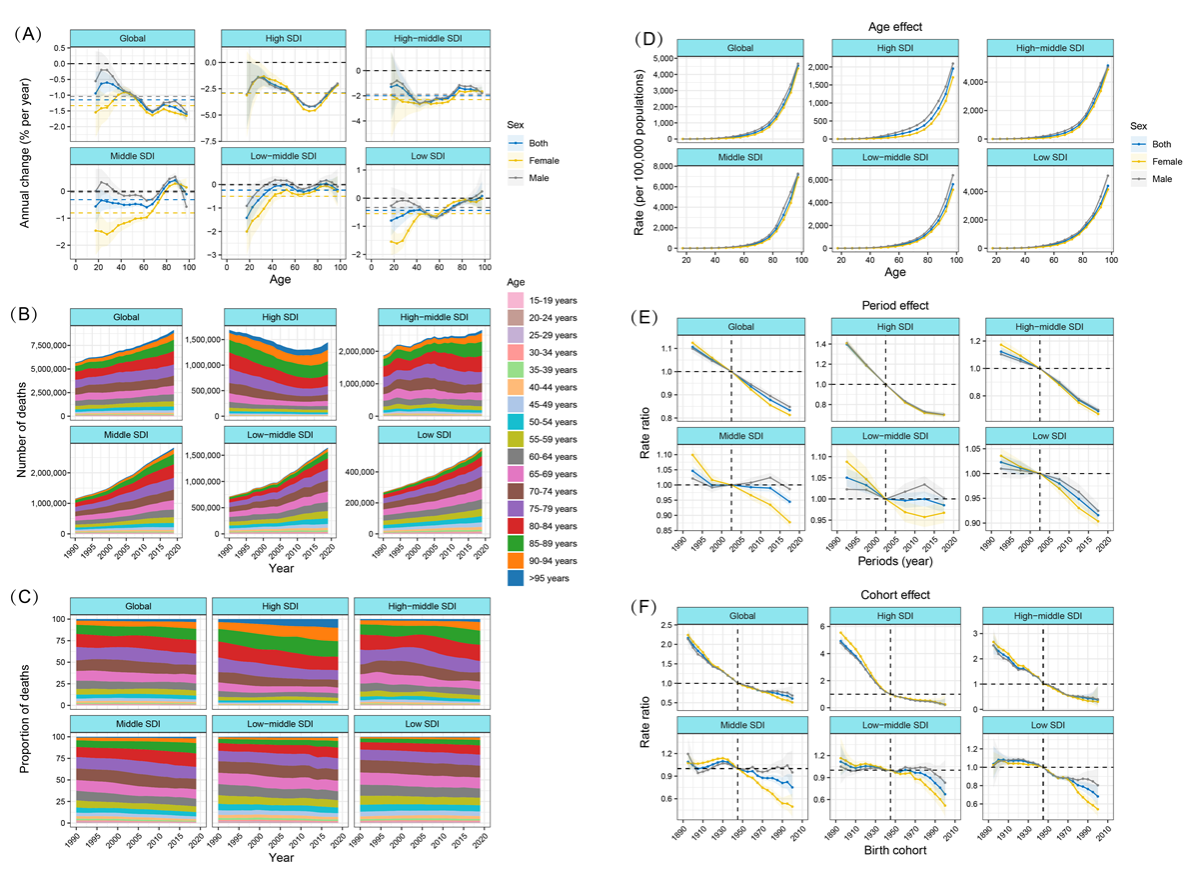
Time trends and age-period-cohort effects on ischemic heart disease mortality by global and sociodemographic index (SDI) quintiles, 1990-2019. (A) Net and local drifts of ischemic heart disease mortality (estimates from age-period-cohort models) for 19 age groups (5-9 to >95 years), 1990-2019. The dots and shaded areas indicate the annual percentage change of mortality (%) and the corresponding 95% CIs. The topmost black horizontal line marks “0” change. The horizontal discontinuous lines show overall annual percentage changes in mortality (net drifts), with yellow for female, gray for male, and blue for both. The continuous solid lines show annual percentage changes from 5-9 to >95 years (local drifts), in male (gray), female (yellow), and blue for both. (B) Temporal change in the absolute cases of ischemic heart disease mortality across age groups, 1990-2019. (C) Temporal change in the relative proportion of ischemic heart disease mortality across age groups, 1990-2019. (D) Age effects are shown by the fitted longitudinal age curves of mortality (per 100,000 person-years) adjusted for period deviations. (E) Period effects are shown by the relative risk of mortality (mortality rate ratio) and computed as the ratio of age-specific rates (adjusted for age and nonlinear period effects) from 1990-1994 to 2015-2019 (2000-2004 as the referent period). (F) Cohort effects are shown by the relative risk of mortality and computed as the ratio of age-specific rates (adjusted for age and nonlinear period effects) from the 1895 cohort to the 2000 cohort, with the referent cohort set at 1945. The dots and shaded areas denote mortality rates or rate ratios and their corresponding 95% CIs.

Globally, the overall net drift for IHD mortality was –1.15% (95% CI –1.21% to –1.09%), which was greater in the female (–1.33%, 95% CI –1.41% to –1.25%) than in the male group (–1.04%, 95% CI –1.11% to –0.96%), with local drifts fluctuating downward across all age groups (<0%; *P*<.001; Tables S2 and S3 in Multimedia Appendix 2 and Figure 3A). The global local drift went below the net drift after the 55- to 59-year-old age group and reached nadirs in 65- to 69-year-old age group (–1.54%, 95% CI –1.59% to 1.48%) and >95-year-old age group (–1.61%, 95% CI –1.79% to –1.43%). Among SDI quintiles, IHD mortality was substantially reduced in high-SDI (net drift –2.89%, 95% CI –3.07% to –2.71) and high-middle–SDI (net drift –2.00%, 95% CI –2.17% to –1.83) regions, especially between the ages of 40 and >95 years and 35-69 years (local drift <–2.0%), respectively. However, the reductions in IHD mortality in low-SDI regions were less striking, with the death risk increasing with age, especially in the low-SDI region.

At the national level, 43 countries and territories had increased trends (net drifts *≥*0.0%) and 29 had modest reductions (–0.5 to 0.0%) in mortality from IHD (Figure 2B and Figures S3A-S3F in Multimedia Appendix 1). There were 9 nations with net drifts >1.00%, with 2 from middle-SDI (Philippines and Uzbekistan), 5 from low-middle–SDI (Lesotho, Zimbabwe, the Dominican Republic, Timor-Leste, and Kenya), and 2 from low-SDI (Mozambique and Guinea) regions. Large decreases in IHD mortality were estimated in 90 nations (net drift <–2.00%), in which the biggest decline occurred in the Republic of Korea (high SDI) with net drift –6.06% (95% CI –6.23% to –5.88%), followed by nations from 5 high-SDI (Denmark, Norway, Estonia, the Netherlands, and Ireland) and 2 high-middle–SDI (Israel and Bahrain) regions with net drift <–5.00%. With significant decreases in IHD-related deaths over the past 30 years, Saint Lucia (net drift –3.21%, 95% CI –5.33% to –1.04%) and Equatorial Guinea (–2.9%, 95% CI –3.75% to –2.03%) were typical countries in middle-SDI, the Maldives (–4.03%, 95% CI –4.98% to –3.06%) and Belize (–2.18%, 95% CI –3.43% to –0.93%) in low-middle–SDI, and Afghanistan (–1.35%, 95% CI –1.43% to –1.27%) and Yemen (–1.05%, 95% CI –1.14% to –0.95%) in low-SDI regions. Temporal changes in the age distribution of death number and death percent, as an indirect marker of IHD population survival, are shown in Figures 3B and 3C and Figures S4A-S4F and S5A-S5F in Multimedia Appendix 1.

Sex variations in time trends of IHD mortality are presented in some regions and nations with local drifts. Globally, the local drift in male group resembled that in female group, reaching the bottom in the age group of 65-69 years (female: –1.64%, 95% CI –1.71% to –1.56%; male: –1.51%, 95% CI –1.58% to –1.43%), but the reduction in IHD mortality in male group aged 45 years or younger was not as steep as in female group, which was more pronounced in low-SDI regions (Figure 3A). For high- and high-middle–SDI regions, the improvements in trends in IHD mortality were mostly similar in male and female groups (Figures S3B and S3C in Multimedia Appendix 1). It is worth noting that in American Samoa (high-middle SDI), female group had a significantly higher IHD mortality than male group. For lower-income nations (middle, low-middle, and low SDI), the time trends for IHD mortality were greater in female than male group in many countries (Figures S3D-S3F in Multimedia Appendix 1).

### APC Effects on IHD Mortality

For age effect, IHD mortality increased with age, with a sharp increase in the later years of life (>60 years; Figure 3D). Similar patterns of IHD mortality trends were found across different SDI quintiles, with modestly higher mortality in male than female group, but high-SDI region showed an overall lower mortality across all age groups, with a nadir between 60 and 90 years of age. For period and cohort effects, trends of rate ratios for IHD mortality declined across successive period groups (refer to 2000-2004) and birth cohort groups (refer to 1985-2000) and showed similar directions in SDI quintiles, with noticeable improvements in high-SDI region (Figures 3E and 3F). In low-SDI regions, IHD mortality declined significantly for female group but fluctuated horizontally for male group (middle SDI and low-middle SDI) across successive periods. For cohort effects, sex differences (decline in female greater than male) were more remarkable in those born after 1945 in middle- and low-middle–SDI regions, and after 1970 in low-SDI region.

**Figure 4:**
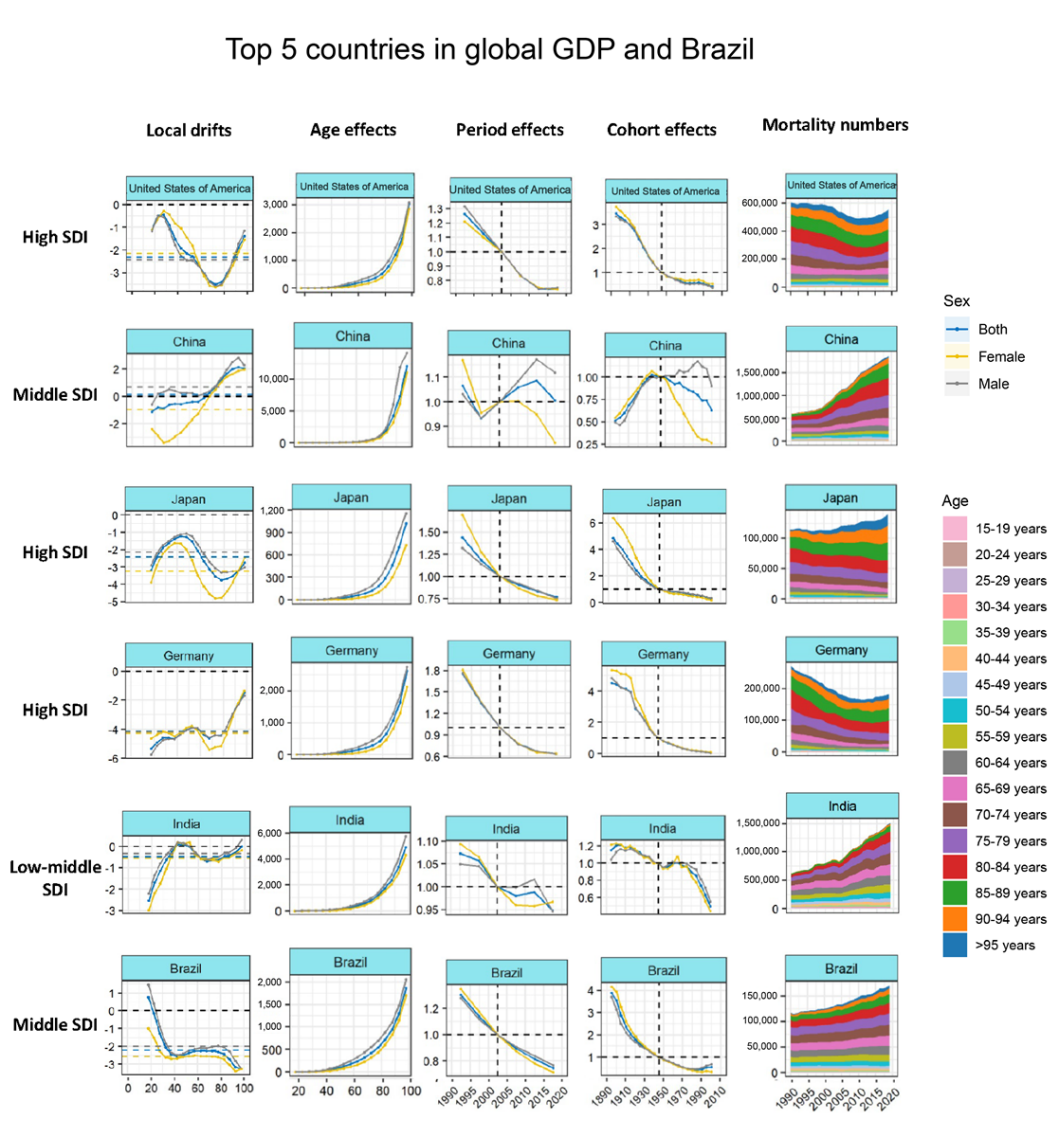
Examples of countries with age-period-cohort effects on mortality from ischemic heart disease (IHD) across sociodemographic index (SDI) quintiles. Age-period-cohort effects for IHD mortality in the top 5 countries in global gross domestic product (GDP) and Brazil.

APC effects for IHD mortality in the top 5 countries and Brazil, according to the rank of gross domestic product obtained from the International Monetary Fund in 2019 [25], are shown in Figure 4. The United States, Japan, Germany, and Brazil had decreased trends for IHD mortality but with less age effects and declining period and cohort risks. China had significantly increased risks in people aged 75 years or older and those born before 1945. India also had high mortality among those aged 40 years or older, with little difference among period groups. The age, period, and cohort effects on IHD mortality across 21 GBD regions and 204 countries and territories are shown in Figures S6A-S6F, S7A-S7F, and S8A-S8F in Multimedia Appendix 1.

### Risk Factors for Mortality From IHD

**Figure 5:**
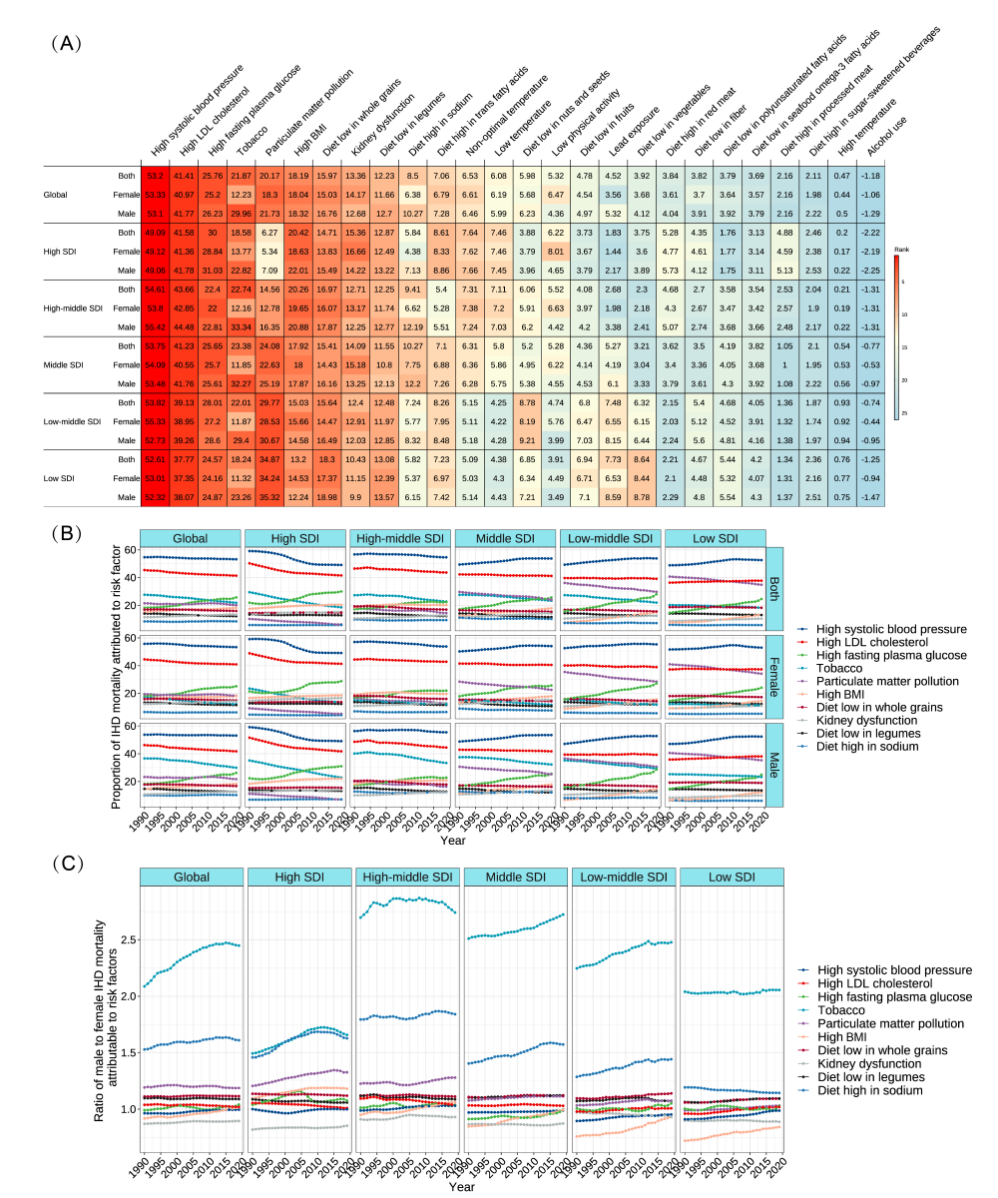
Top risk factors with population attributable fractions and time trends among the most specific Global Burden of Disease 2019 Study risks for mortality from ischemic heart disease (IHD). (A) Ranked contribution of the top 26 risk factors to the age-standardized death rate of ischemic heart disease by global and sociodemographic index (SDI) quintiles, 2019, for combined sex, female, and male. (B) Time trends of the top 10 risk factors to the age-standardized death rate of ischemic heart disease by global and SDI quintiles, 1990 to 2019, for combined sex, female, and male. (C) Time trends of the top 10 risk factors for mortality from ischemic heart disease with the ratio of male to female by global and SDI quintiles, 1990 to 2019. Risk factors are ranked according to colors (leading risk factor for age-standardized death=dark red; lowest risk factor for age-standardized death=dark blue). LDL: low-density lipoprotein.

**Figure 6:**
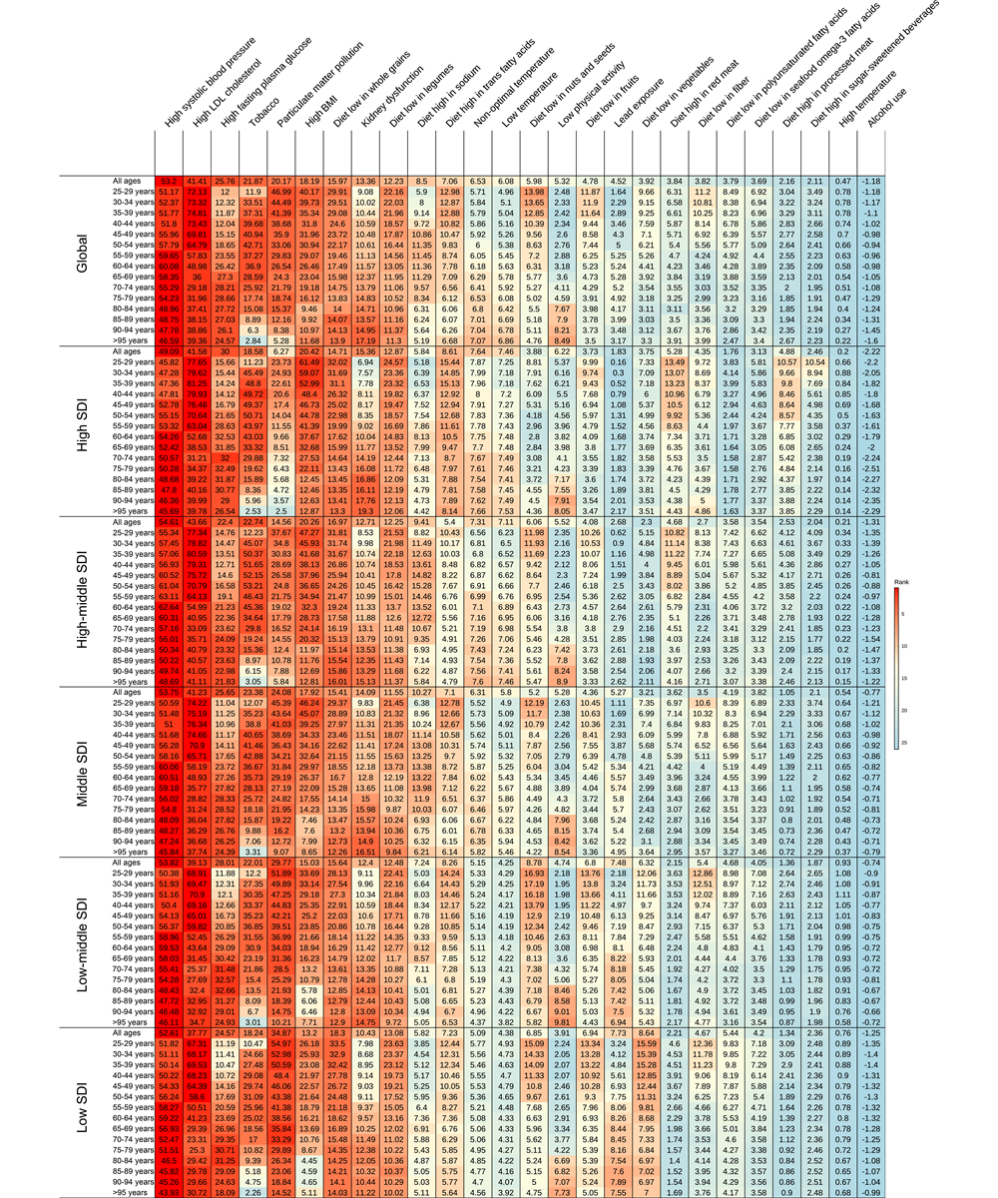
The trends of population attributable fractions for 26 factors with age by global and sociodemographic index (SDI) quintiles, 2019, for both sexes combined. LDL: low-density lipoprotein.

Across the top 26 risk factors (5 metabolic, 16 behavioral, and 5 environmental), with PAF, high systolic blood pressure (SBP), air pollution, and 3 other metabolic risks (high low-density lipoprotein [LDL] cholesterol, high fasting plasma glucose [FPG], and BMI) were the key contributors to IHD mortality in all SDI regions (Figure 5A and Figures S9A-S9H in Multimedia Appendix 1). In 2019, high SBP and LDL cholesterol were the 2 leading risk factors, contributing to 53.20% (95% CI 43.38%-62.76%) and 41.41% (95% CI 31.48%-52.05%) in global total deaths, respectively. From 1990 to 2019, the high FPG contribution to IHD mortality increased markedly in global and 5 SDI quintiles. During the same period, the high SBP risk increased slightly in 3 low-SDI regions but decreased in high- and high-middle–SDI regions, especially in the high-SDI region (Figure 5B). High LDL cholesterol and high BMI were prominent among young people, while high FPG and kidney dysfunction were prominent in older people (Figure 6).

Behavioral risks associated with increased IHD mortality included smoking, 13 dietary risks, low physical activity, and alcohol use. The proportion of deaths from IHD that was attributable to smoking, which was the third hazard in 3 low-SDI regions, varied substantially by sex: 29.96% (95% CI 28.74%-31.33%) in male compared with 12.23% (95% CI 11.27%-13.30%) in female (Figure 5), and was concentrated in the population 35-64 years of age (>35%; Figure 6). Dietary risks decreased with age, in which diets low in whole grains and legumes were prominent (Figure 6), and the risk of a high-sodium diet was more skewed toward male than female (Figure 5C). The risk of low physical activity increased with age (Figure 6). Interestingly, alcohol use had noticeable protective effects for IHD at the global and national levels (Figure 6 and Figures S9A-S9H in Multimedia Appendix 1).

Particulate matter pollution, an environmental risk, was the third-highest specific risk factor for IHD mortality in 3 low-SDI regions (Figure 5) and the third factor in several GBD regions (Eastern, Western, Central Sub-Saharan Africa, and South Asia; Figure S9A in Multimedia Appendix 1), but only the 12th in high-SDI regions (Figure 5), the risk of which increased with age (Figure 6). The older individuals were more susceptible to low temperatures, and lead exposure was more hazardous in low-SDI regions (Figure 6). The ranks, time trends, sex differences, and age trends of regional and national risk factors for IHD mortality are shown in Figures S9A-S9G, S10A-S10D, S11A-S11C, and S12A-S12B in Multimedia Appendix 1.

## Discussion

### Overview

This report is the first to use the APC model to analyze temporal trends in IHD mortality on a global scale and across 204 countries and territories. Globally, the total number of IHD-related deaths increased over the past 30 years and continued to be the leading cause of NCD deaths in 2019. By contrast, ASMR decreased, with the biggest decline in mortality in high-SDI regions. Aging is an independent risk factor for mortality from IHD, with noticeable sex differences in period and cohort risks in lower-income countries. Although metabolic risks were the leading factors for mortality from IHD worldwide in 2019, smoking, particulate matter pollution, and dietary risks were also important risk factors, increasingly occurring at a younger age. Diets low in whole grains and legumes were prominent dietary risks, regardless of sex, while smoking and a high-sodium diet were more frequent in the male than female group.

The trends for mortality from IHD varied across different regions and countries and were not completely commensurate with SDI levels. Overall, high-SDI regions and countries had higher mortality rate for IHD, but with an impressive annual downward trend. However, ASMR increased in Guam (high SDI) and Ukraine (high-middle SDI), whereas a significant decrease occurred in lower-income countries, for example, Rwanda, Ethiopia, and Afghanistan. In higher-income countries, hypertension, hyperlipemia, diabetes, and obesity have gradually become an increasing threat, which are key risk factors accounting for a large proportion of the mortality rate [26-31]. However, newer modalities of disease prevention and a better-integrated health care system contributed to the greatest reduction in IHD mortality in higher-income countries. For lower-income countries, the Westernized lifestyle with increased metabolic risks and severe air pollution is causing IHD to emerge as a growing health hazard [32-36]. A previous study showed that CVD mortality was much higher in patients with diabetes in lower- than higher-income countries. This increased risk was not altered even when risk factors such as physical inactivity, smoking, BMI, and hypertension and treatments are taken into account [37] but may be related to the fact that lower-income countries are severely underresourced in the number of health care providers, technological infrastructure, and implementation of treatment [38-40]. Therefore, countries with different degrees of SDI, especially lower-income countries with more complex and challenging situations, need to formulate appropriate prevention and intervention strategies based on local health-related resources.

The marked upsurge of IHD mortality in people aged 65 years or older shows that aging is an independent risk factor for mortality from IHD [41,42]. Aging is a nonmodifiable risk factor for IHD, with cellular senescence and vascular aging accounting for atherosclerosis, which is the primary cause of IHD [43,44]. The older individual is susceptible to immune dysfunction and chronic metabolic diseases, which increase the risk of IHD mortality [45]. We found that risk due to low physical activity increased with age, which was associated with CVD mortality [46]. Furthermore, the aging of the population caused by low birth rate and longer life expectancy also indirectly leads to an increase in IHD mortality [14]. In China, IHD mortality, controlled for period and cohort effects, increased sharply after the age of 70 years, where 65.6% of the health burden were projected to occur in older adults in 2050 [47]. The age at which there is a rapid rise in mortality is also delayed in other SDI countries where death trends are increasing, such as Ukraine, the Philippines, Lesotho, and Mozambique, among others. These data suggest that prevention and treatment strategies and multidisciplinary management for IHD in the older population should be a major focus of health systems.

The period and cohort effects on IHD mortality have shown an overall downward trend, which may be attributable to the implementation of better public health policies and improvements in health care [48]. Social stability, improvement in living standards, and advances in medical technologies (medical green channels, eg, Chest Pain Center, advanced interventional techniques, medical devices, and therapeutic drugs) have contributed to the improvement in survival rates of patients with IHD [49,50]. However, unfavorable period and cohort effects continue in some countries, especially in those with lower income. From 1990 to 2019, the high SBP contribution to IHD mortality increased slightly in low-SDI regions, but the risk related to high FPG increased across 5 SDI quintiles. Hazards for IHD mortality caused by high LDL cholesterol, particulate matter pollution, high BMI, and 13 dietary risks are mainly concentrated in the younger age group. The United States (high SDI) is one example of a country that has begun to control air pollution, reducing by more than 80% the atmospheric particulate matter concentration in 2017; the Clean Air Act was passed in 1970 and amended in 1977 and 1990 [51]. China (middle SDI) and India (low-middle SDI) also showed a decline in household air pollution–attributable IHD mortality with a series of measures to decrease air pollution [52,53]. One recent study showed that greater adherence to healthy eating patterns, as recommended by the Dietary Guidelines for Americans, was consistently associated with lower risk of total and IHD-caused mortality [54]. Okinawans, 65 years of age or older, have a lower risk for metabolic diseases and mortality, which may be related to the traditional Okinawan diet consisting of 85% calories from carbohydrates and 9% from protein [55]. The above reports show that current resources are largely inadequate to manage patients with IHD in many countries, and health care strategies from countries with significant improvements in IHD mortality may serve as a model for countries at the same level.

The association between alcohol use and IHD remains an open question. On the one hand, alcohol consumption has been proven to be a leading risk factor for disease burden, but on the other hand, some researchers have suggested that low levels of alcohol consumption can have a beneficial effect on IHD outcomes [56]. Our analysis showed that alcohol use had noticeable protective effects for IHD, but the effect was small compared with the other attributable burdens in those locations. Although the results of our analysis are consistent with previous analyses [56,57], it does not prove that higher alcohol consumption is beneficial for IHD. When considered with overall health risks, alcohol use was strongly associated with cancer, injuries, and communicable disease. Factors such as sex, ethnicity, alcohol consumption, and frequency also need to be considered in future analyses [58].

Sex differences in IHD mortality trends have gradually emerged, especially in lower-income countries [59,60]. In this study, we found that risks from smoking, a diet high in sodium, and particulate matter pollution were greater in male than female individuals globally. By contrast, risks from kidney dysfunction were greater in female than male individuals in high-SDI regions, and risks from high BMI were higher in female than male individuals in low-SDI regions. The traditional risk factors linked to sex-related determinants may be related to shifting roles and relations under the double burden of employment and caregiving responsibilities, which exacerbate the burden of IHD, especially in developing counties [61-64]. However, high-SDI regions had the lowest ratio of male-to-female IHD mortality attributable to smoking, suggesting that smoking control programs and policies are becoming successful in high-income countries [65,66]. These indicate that country-specific strategies to incorporate sex determinants into health research are essential to the improvement in IHD mortality.

There are several limitations to this study. First, the gathering and quality of data sources are different across countries in GBD. Limitations in some low- and middle-income countries are unavoidable, such as unavailable or incomplete data, delayed and inaccurate reporting, and misclassified coding [1]. Second, although the time trends analysis was performed across about 30 years, this research was based on cross-sectional data from GBD, which was not a cohort study. Third, mortality data with different trends at the global and national level were analyzed, but subnational differences were not calculated. Moreover, the national data on health system quality, including medication use, interventional operation implementation, and access to medical care were not included in the data source in this study. In addition, SDI as a composite indicator of income per capita is time-varying, but this study only used SDI of 2019 for the analysis of data from 1990 to 2019 due to data acquisition limitations. Finally, the mortality data for IHD presented in this report were collected before the COVID-19 pandemic. Studies have reported that COVID-19 can induce CVD and increase the risk of death in those with preexisting CVD [67,68], which may be a key focus for IHD mortality analysis in the future.

### Conclusions

Although there have been reductions in IHD mortality over the past 30 years, especially in high-SDI regions and countries, IHD remains the leading cause of global mortality from NCDs. Aging is highly correlated with IHD-related death. Sex-related differences in period and cohort risks occurred across higher- and lower-income countries. Metabolic risks were the leading factors for IHD mortality worldwide in 2019, followed by smoking, particulate matter pollution, and dietary risks.

## Appendix

Multimedia Appendix 1 Supplemental Figures.

Multimedia Appendix 2 Supplemental Tables.

## Abbreviations

AAMR: all-age mortality rate
APC: age-period-cohort
ASMR: age-standardized mortality rate
CVD: cardiovascular disease
FPG: fasting plasma glucose
GBD: Global Burden of Disease 2019 Study
IHD: ischemic heart disease
LDL: low-density lipoprotein
NCD: noncommunicable disease
PAF: population attributable fraction
SBP: systolic blood pressure
SDI: sociodemographic index
UI: uncertainty interval

## Data Availability

The original data sources for this study are publicly available from the Global Burden of Disease 2019 Study data [16].
