# Supplementary material for: Assessing Global, Regional, and National Time Trends and Associated Risk Factors of the Mortality in Ischemic Heart Disease Through Global Burden of Disease 2019 Study: Population-Based Study
Source: JMIR Public Health Surveill. 2024 Jan 24;10:e46821. doi: 10.2196/46821 (PMC10851120; doi:10.2196/46821)

**Supplemental materials Figure S1 to S12B**

**Figure S1** Mortality rates of ischemic heart disease across age groups by periods and birth cohorts, globally, 1990-2019.


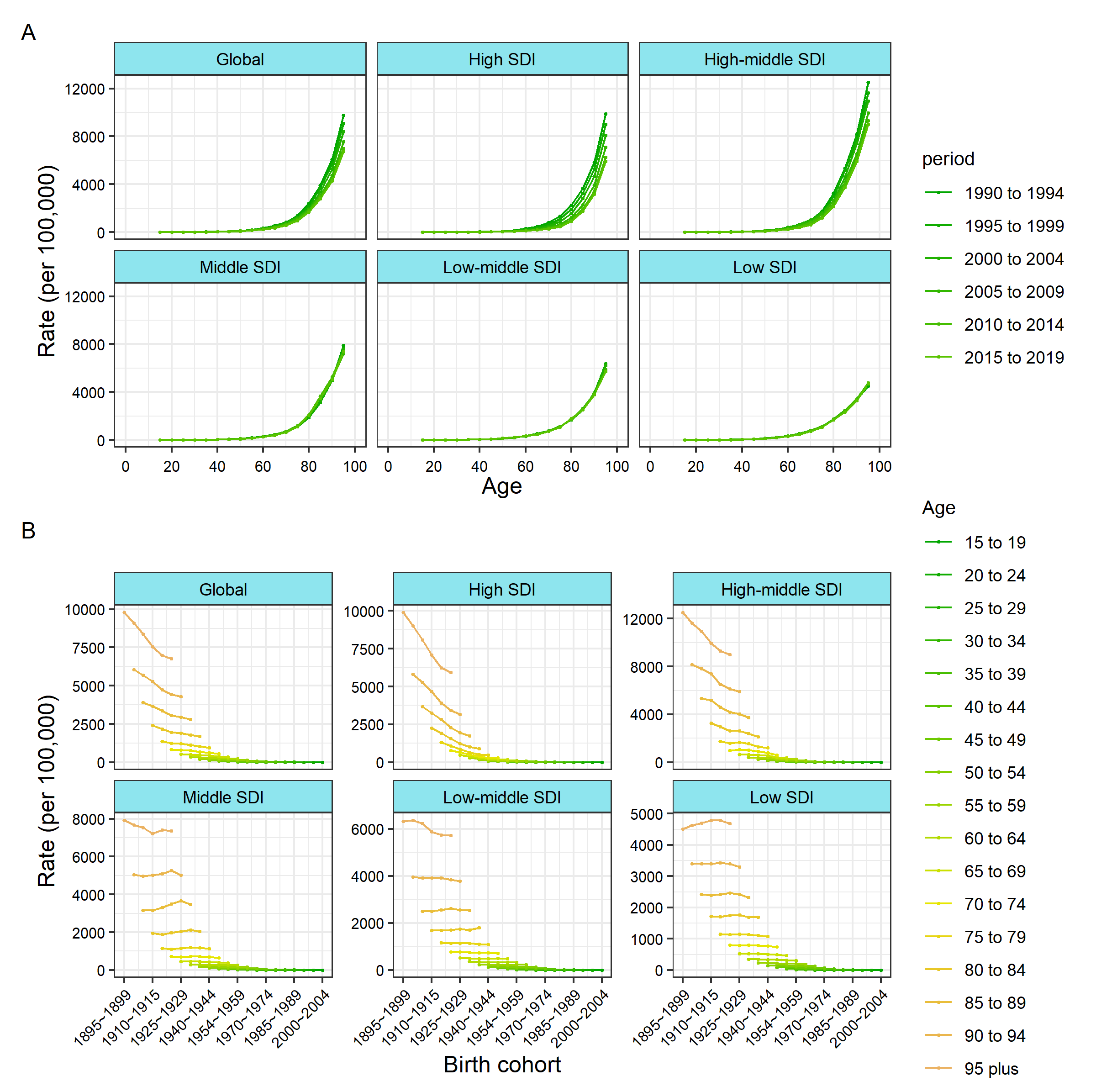


(A) Mortality rates of IHD across age groups by periods. The increased IHD mortality rate (red arrows) with age group and a declining trend in IHD mortality between 1990 to 1994 and 2015 to 2019 (blue arrows). (B) Mortality rates of ischemic heart disease across age groups by birth cohorts. High SDI and high-middle SDI region had a decreased trend of IHD mortality across birth cohorts (blue arrows), whereas three lower SDI regions showed a minimally fluctuant and stable trend across all age groups. IHD, ischemic heart disease; SDI, socio-demographic index.

**Figure S1A** Mortality rates of ischemic heart disease across different age groups by periods in 21 GBD regions, 1990-2019.


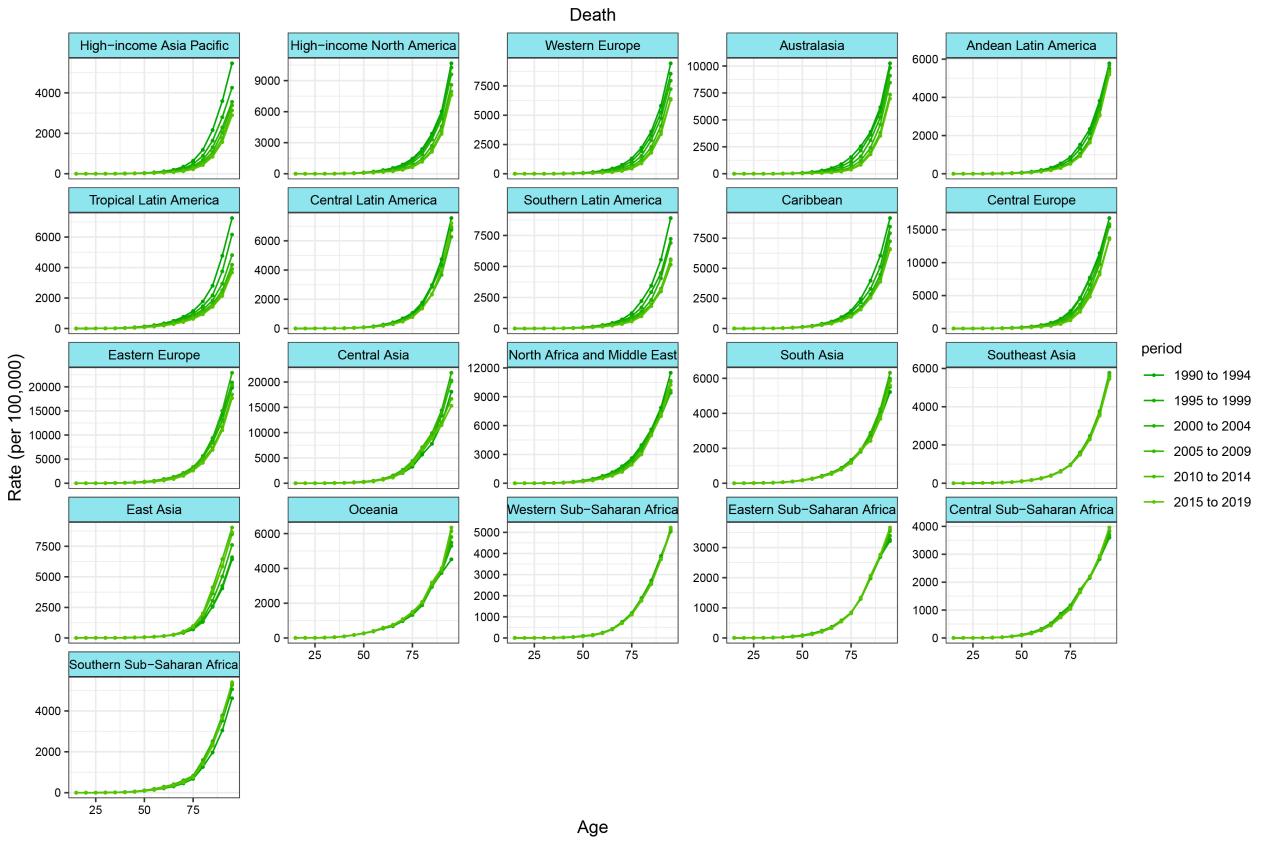


**Figure S1B** Mortality rates of ischemic heart disease across different age groups by periods in high SDI countries, 1990-2019.


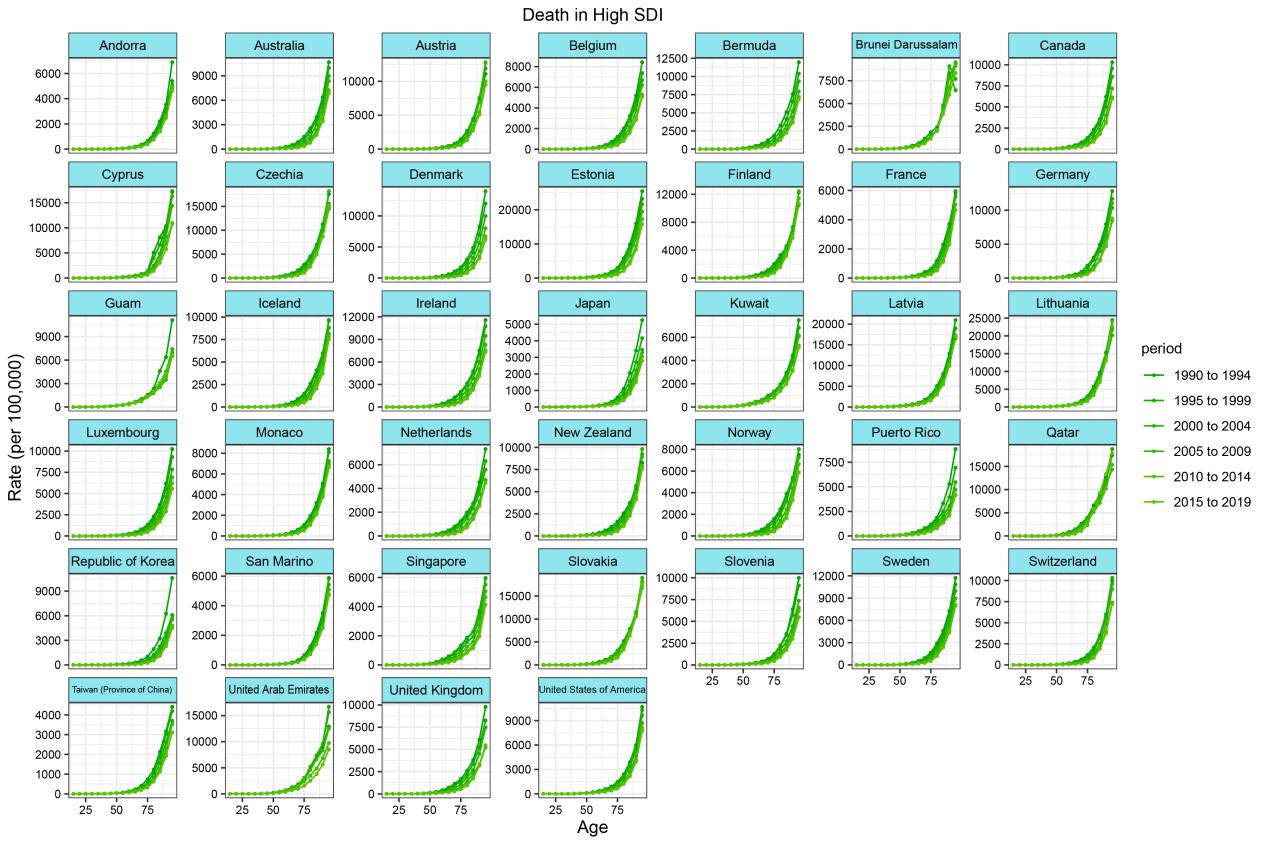


**Figure S1C** Mortality rates of ischemic heart disease across different age groups by periods in high-middle SDI countries, 1990-2019.


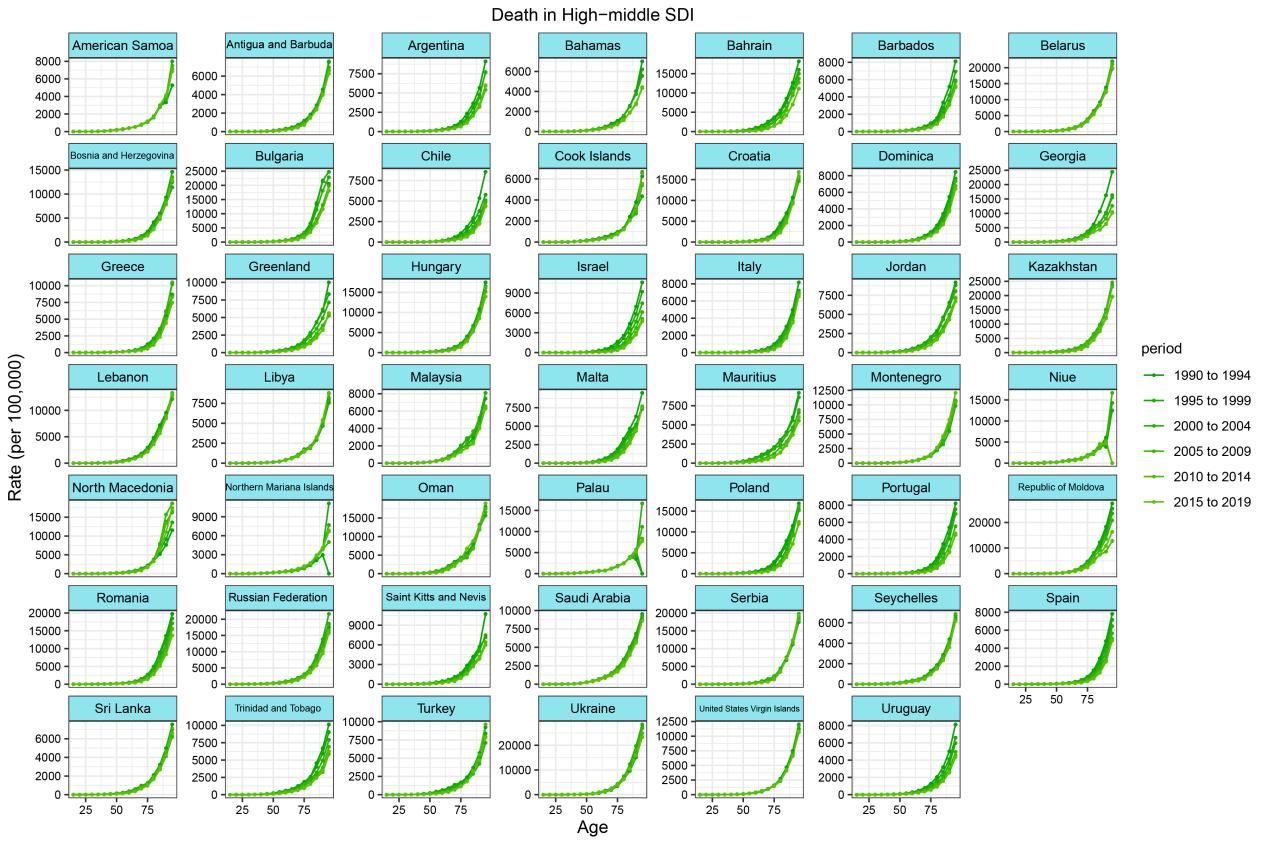


**Figure S1D** Mortality rates of ischemic heart disease across different age groups by periods in middle SDI countries, 1990-2019.


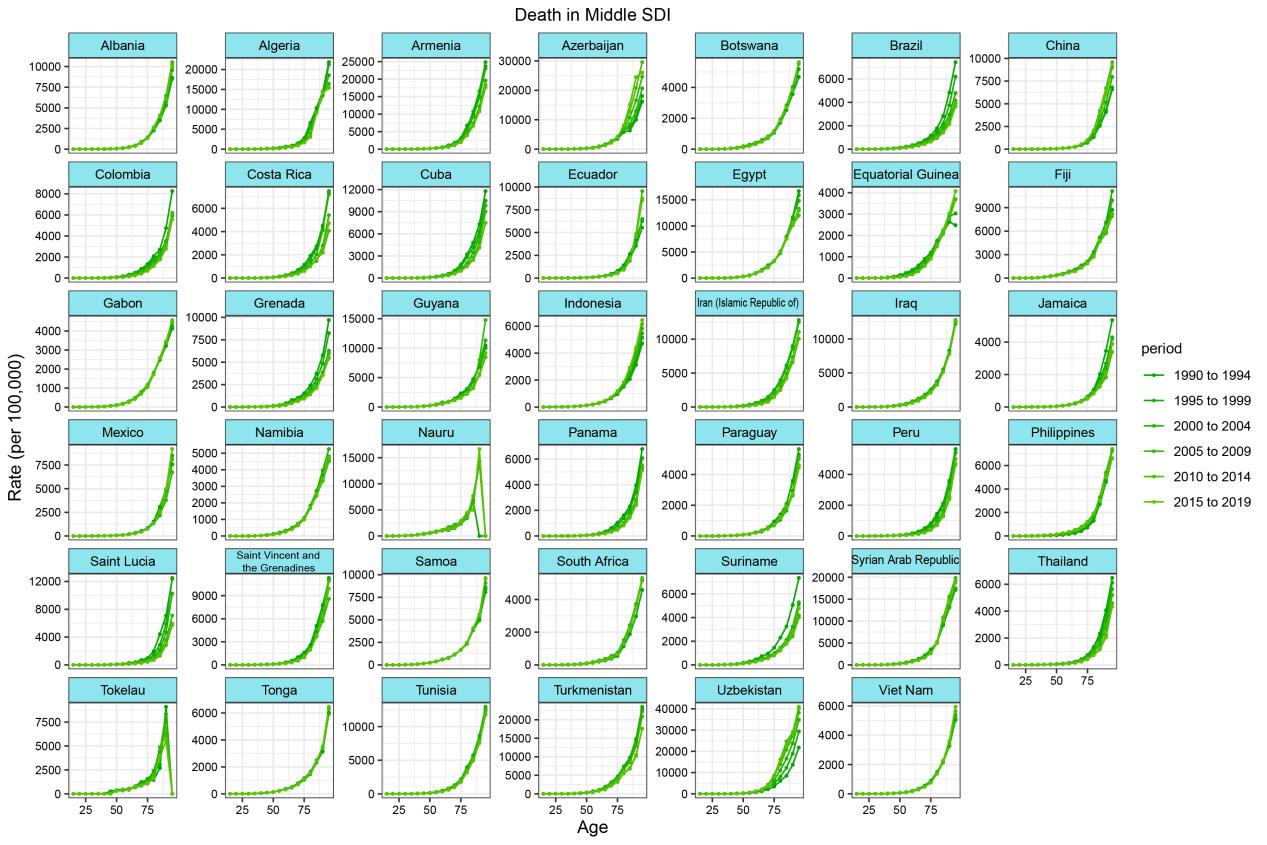


**Figure S1E** Mortality rates of ischemic heart disease across different age groups by periods in low-middle SDI countries, 1990-2019.


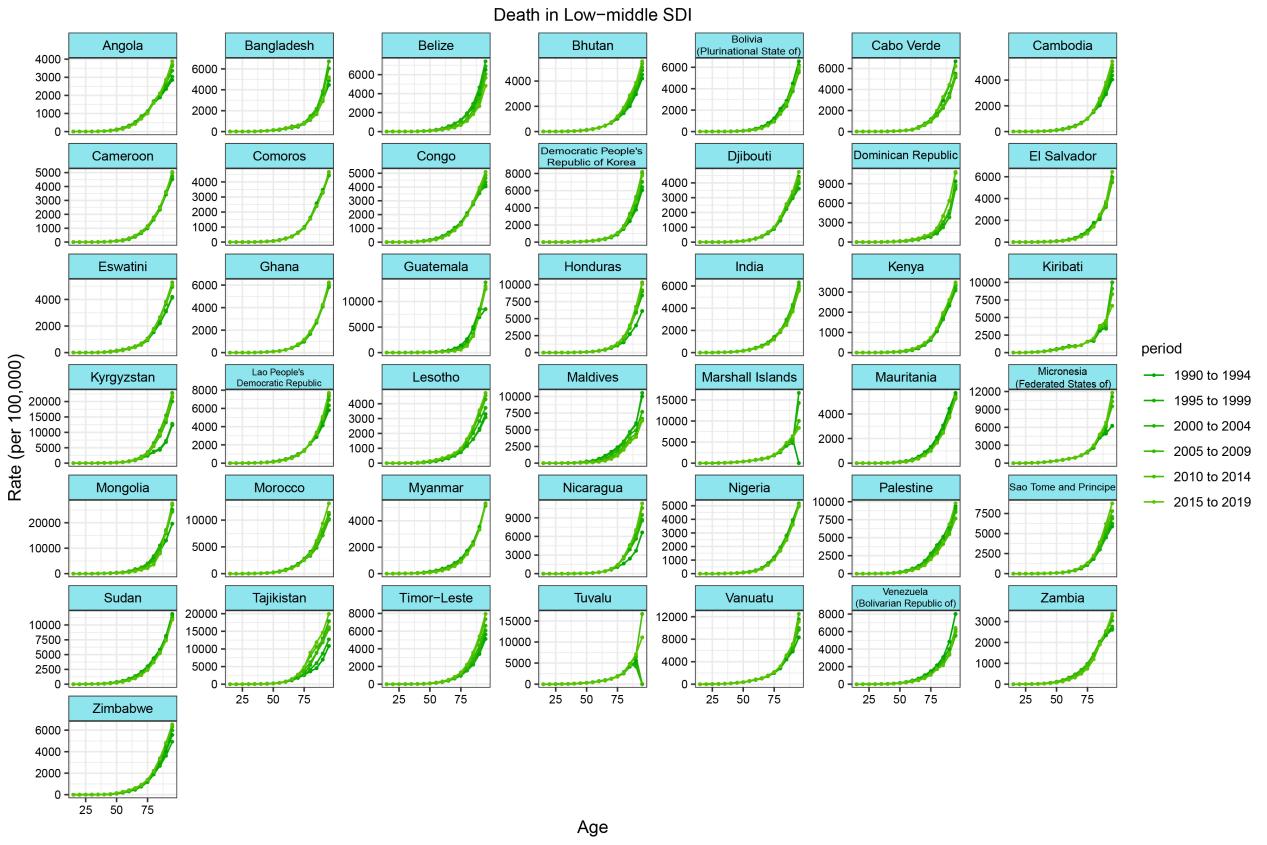


**Figure S1F** Mortality rates of ischemic heart disease across different age groups by periods in low SDI countries, 1990-2019.

**
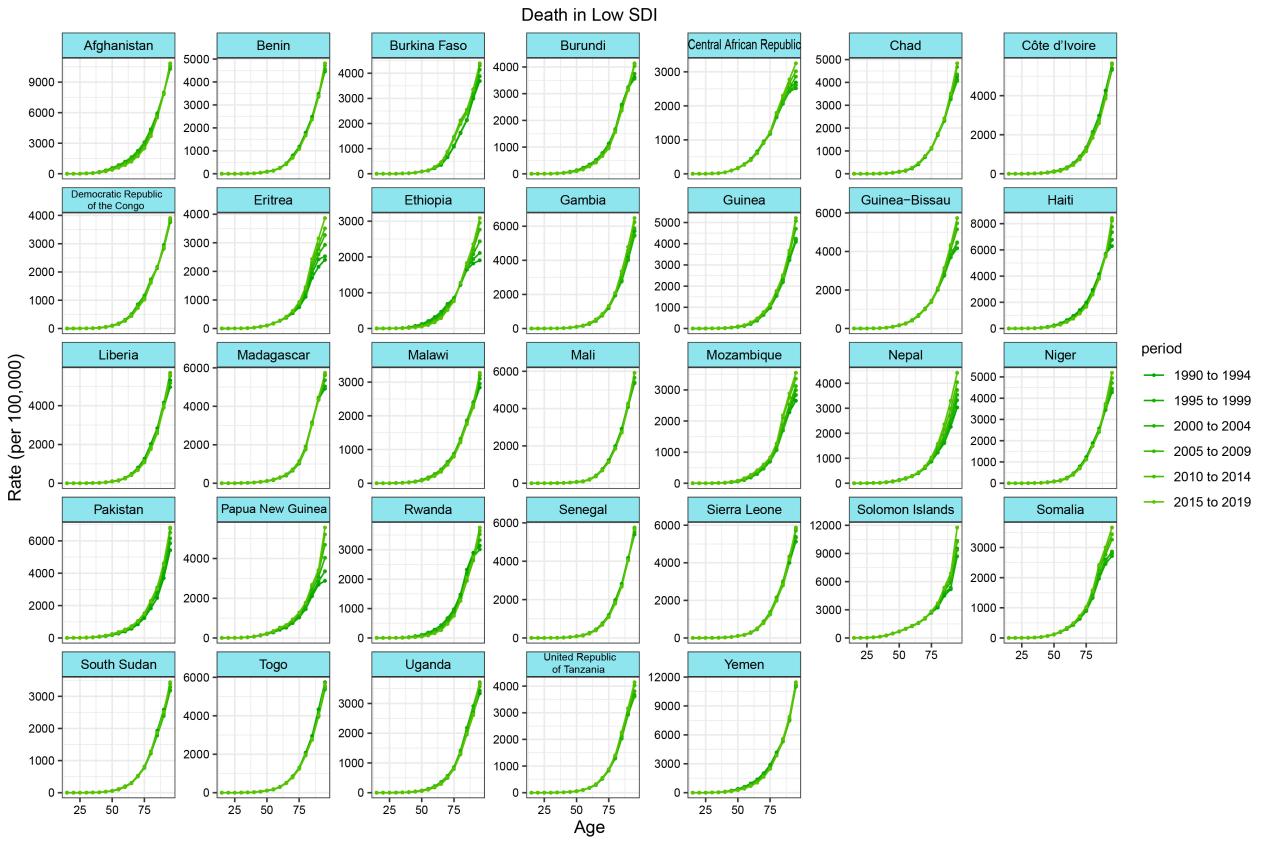
**

**Figure S2A** Mortality rates of ischemic heart disease across different age groups by birth cohorts in 21 GBD regions, 1990-2019.


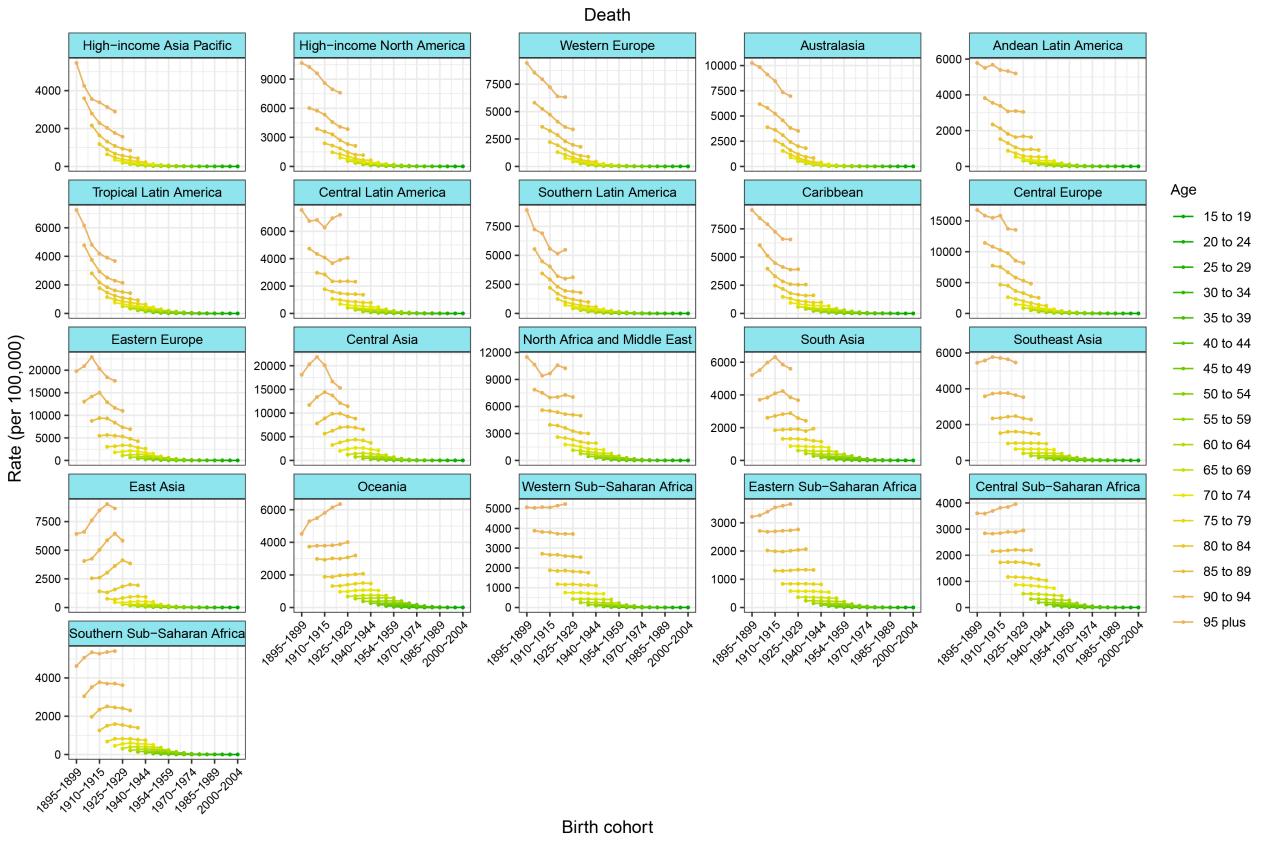


**Figure S2B** Mortality rates of ischemic heart disease across different age groups by birth cohorts in high SDI countries, 1990-2019.


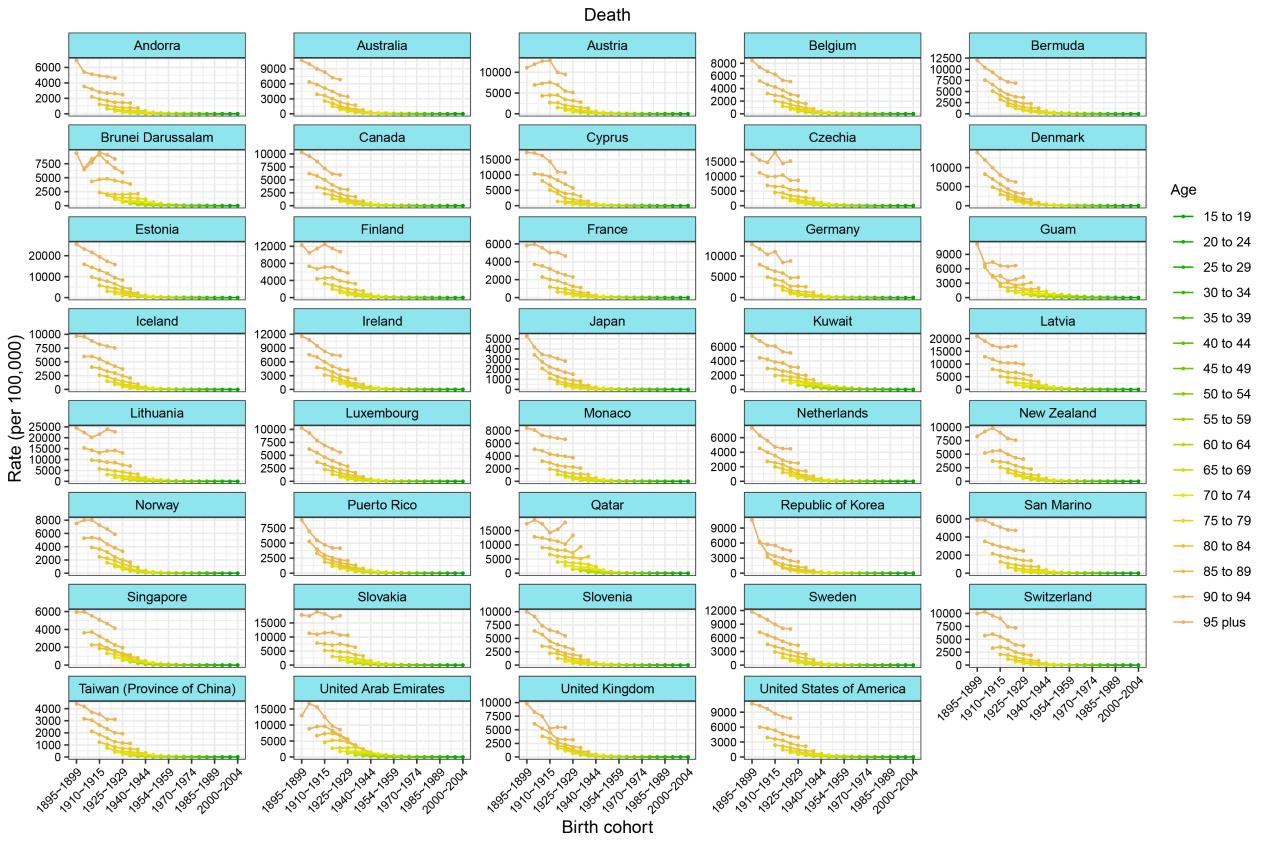


**Figure S2C** Mortality rates of ischemic heart disease across different age groups by birth cohorts in high-middle SDI countries, 1990-2019.


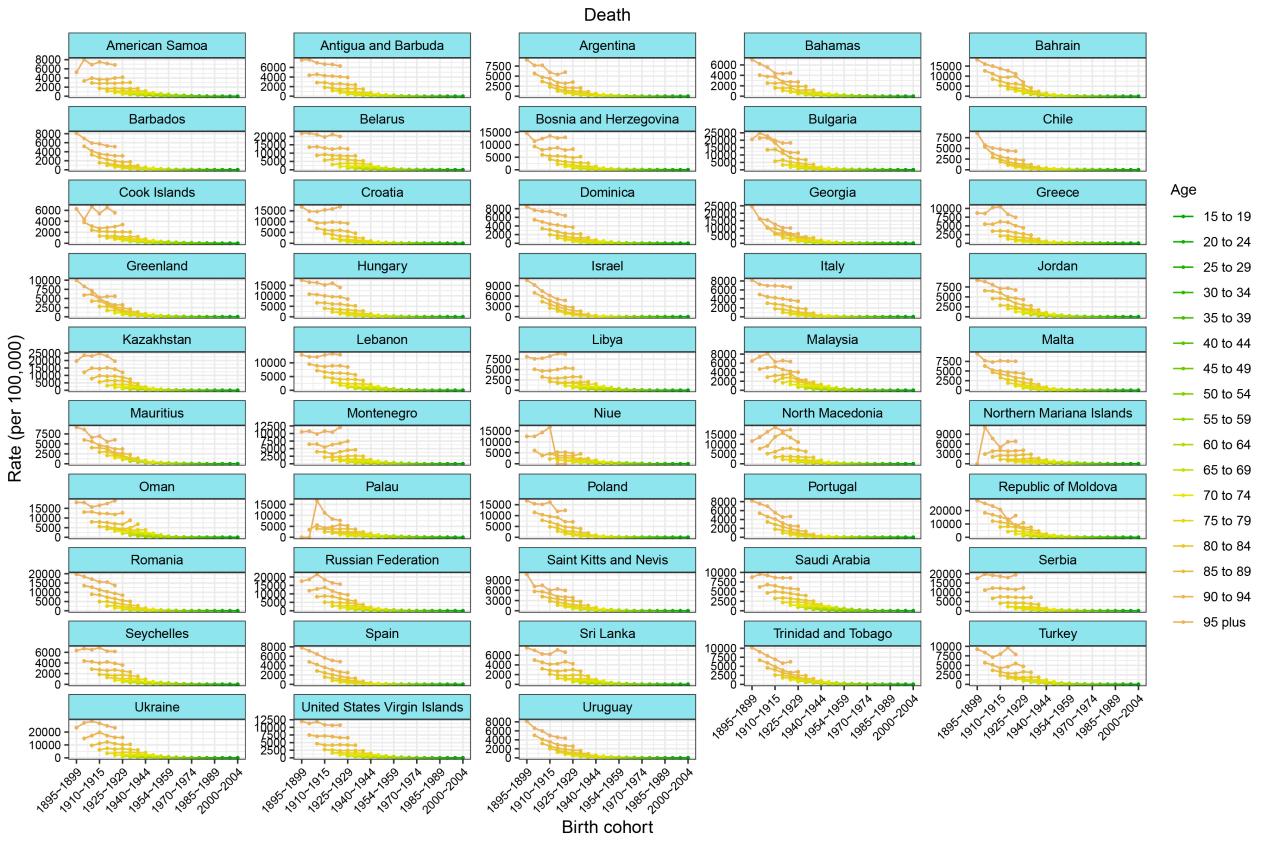


**Figure S2D** Mortality rates of ischemic heart disease across different age groups by birth cohorts in middle SDI countries, 1990-2019.


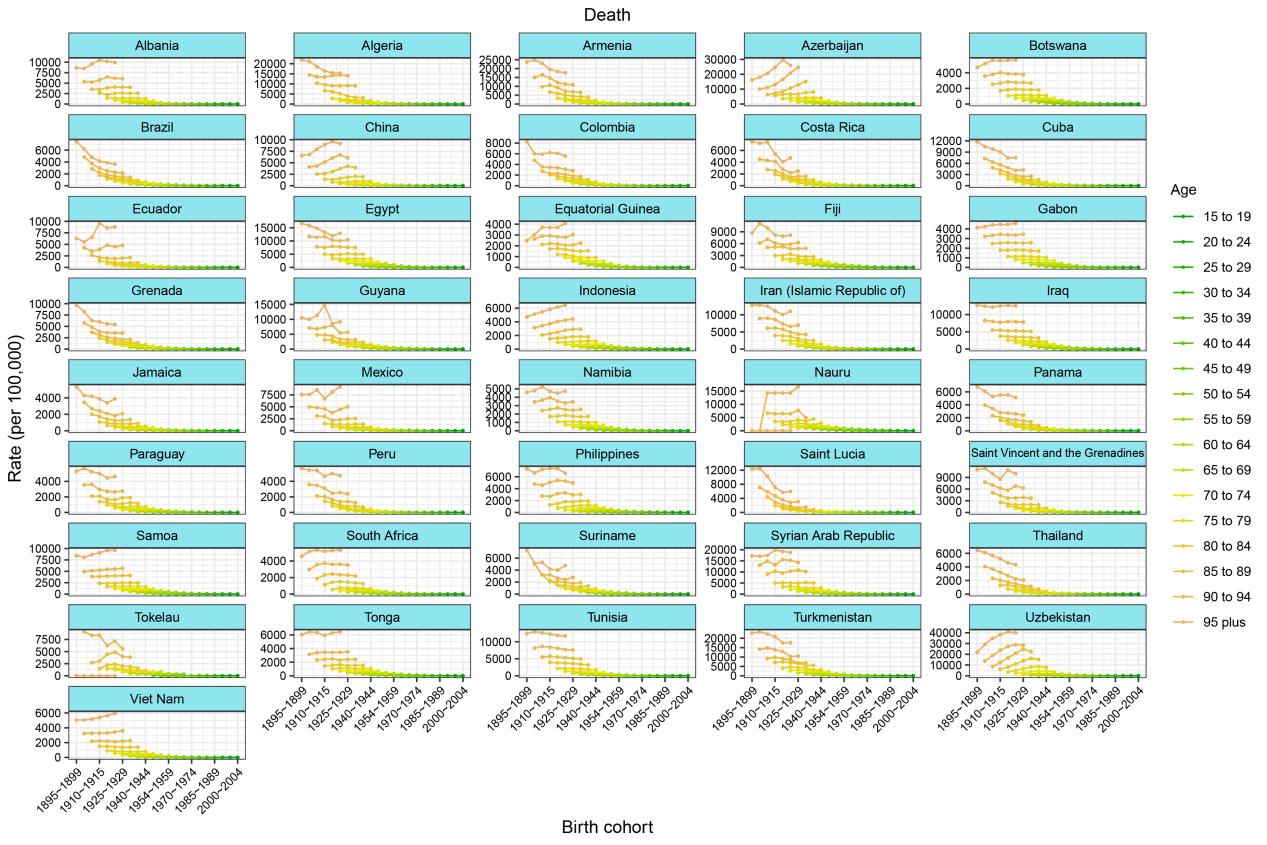


**Figure S2E** Mortality rates of ischemic heart disease across different age groups by birth cohorts in low-middle SDI countries, 1990-2019.


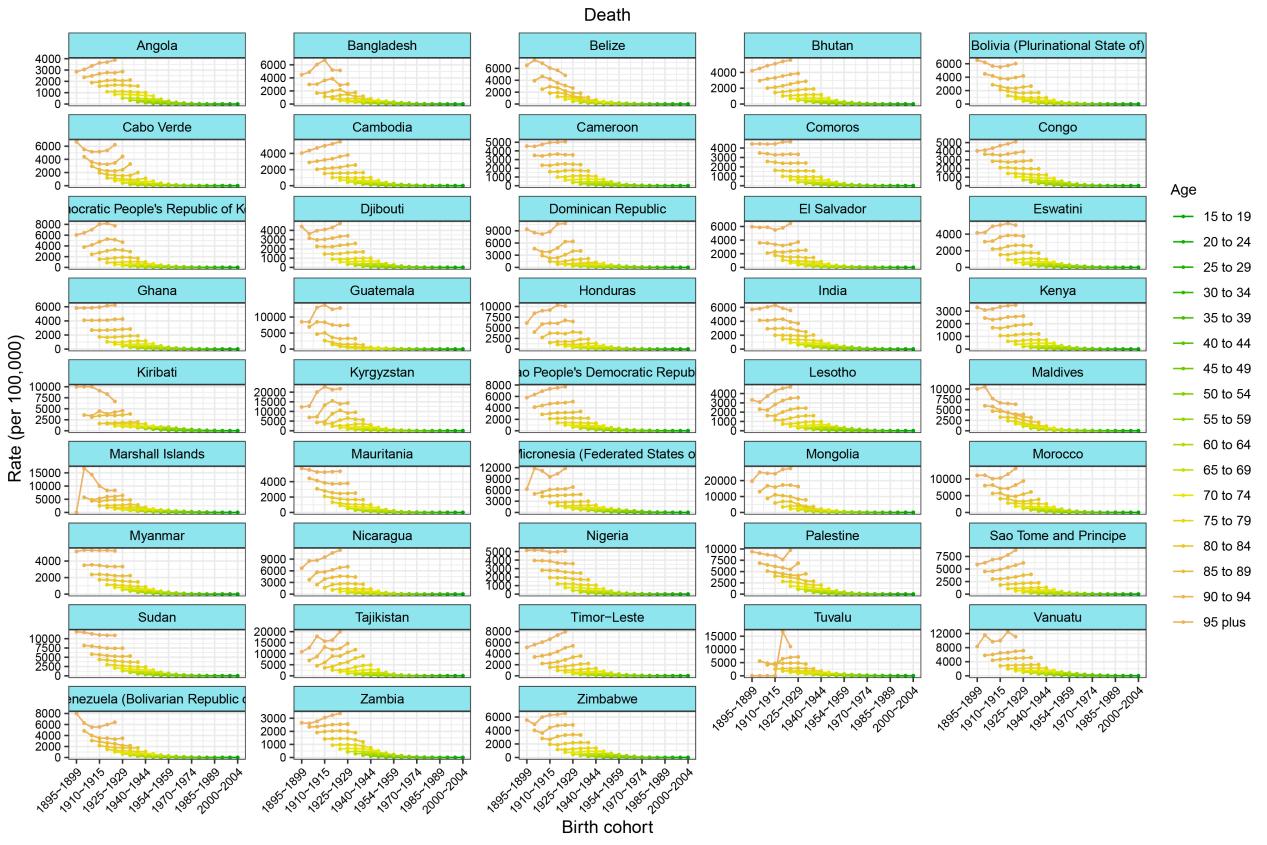


**Figure S2F** Mortality rates of ischemic heart disease across different age groups by birth cohorts in low-SDI countries, 1990-2019.

**
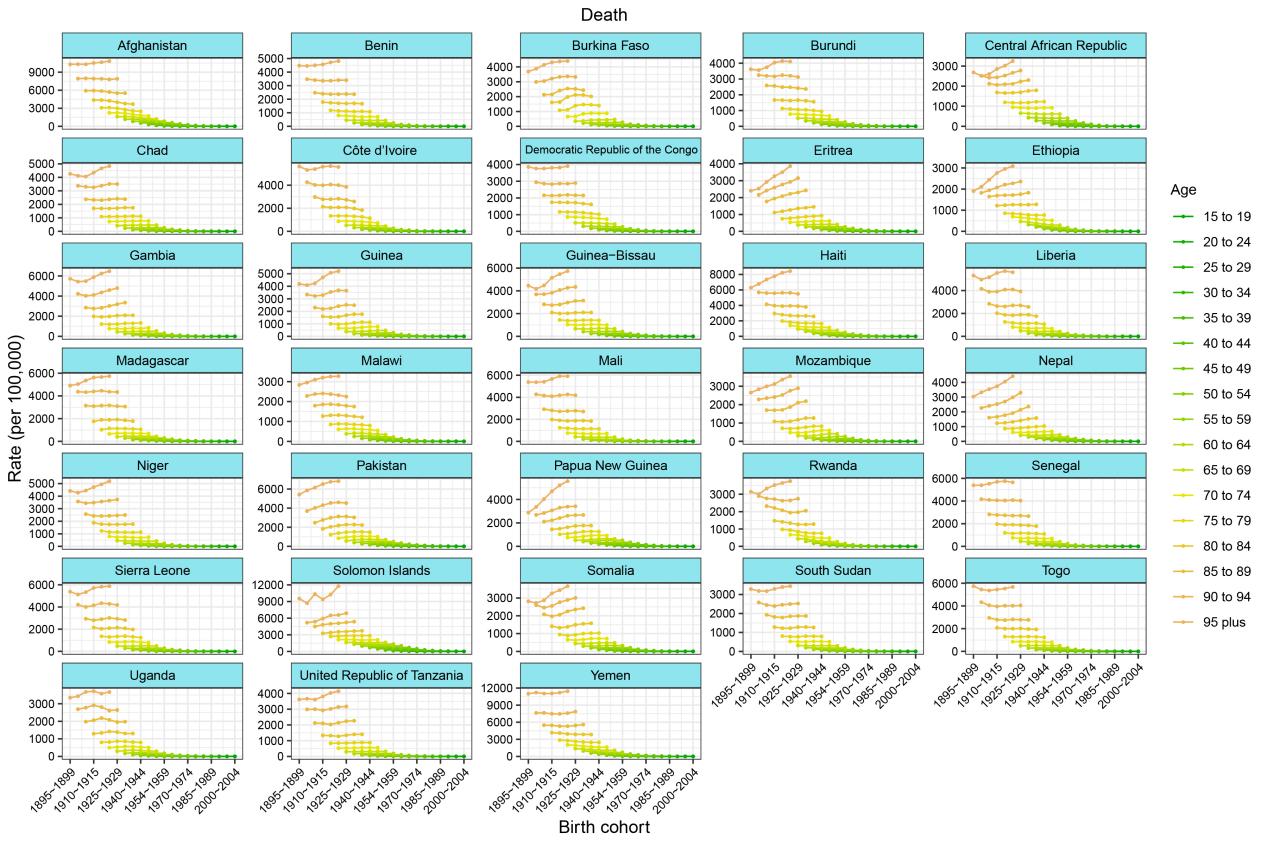
**

**Figure S3A** Net and local drifts of ischemic heart disease mortality in 21 GBD regions, 1990-2019. Local drifts of ischemic heart disease mortality (estimates from age-period-cohort models) for 19 age groups (5−9 to 95 plus years), 1990−2019. The dots and shaded areas indicate the annual percentage change of mortality (%) and the corresponding 95% CIs.


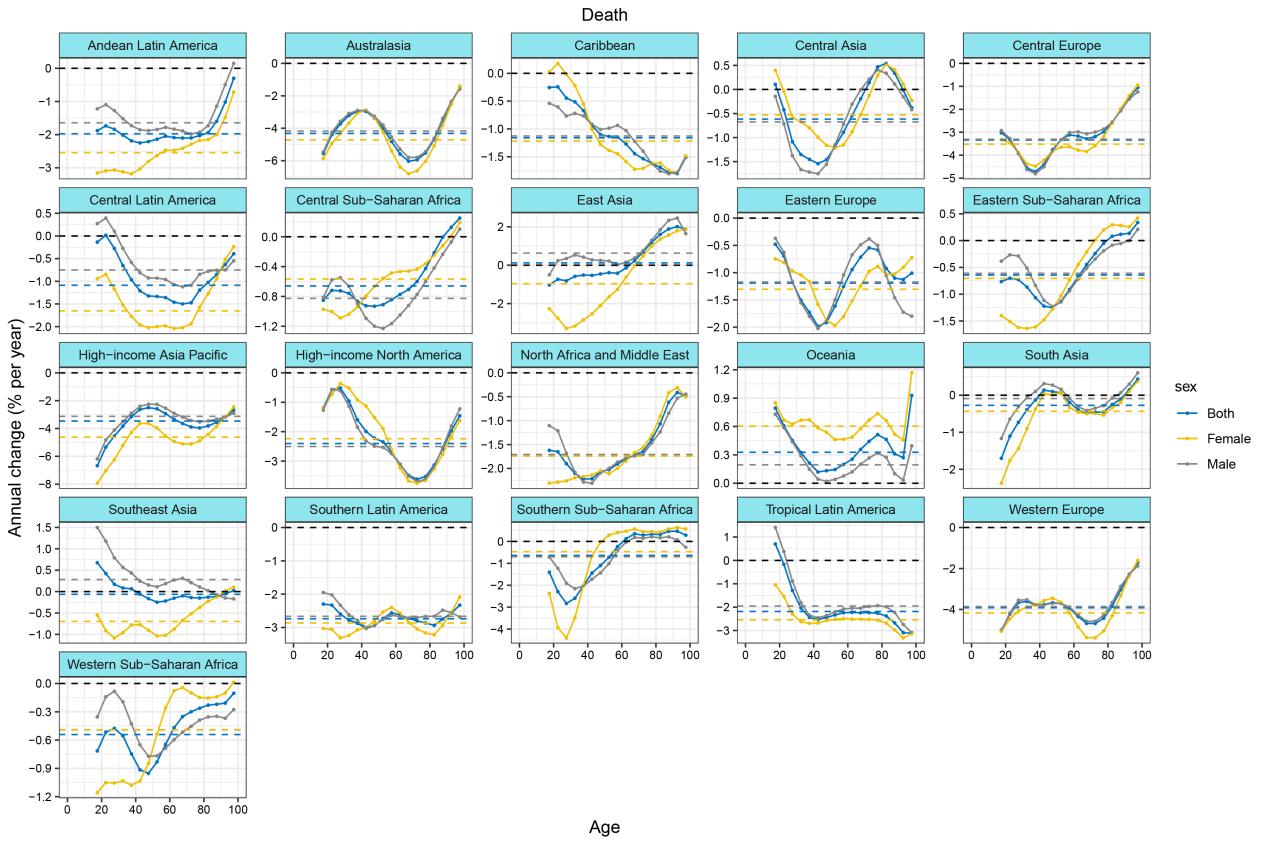


**Figure S3B** The local drifts of ischemic heart disease mortality in high SDI countries, 1990-2019.


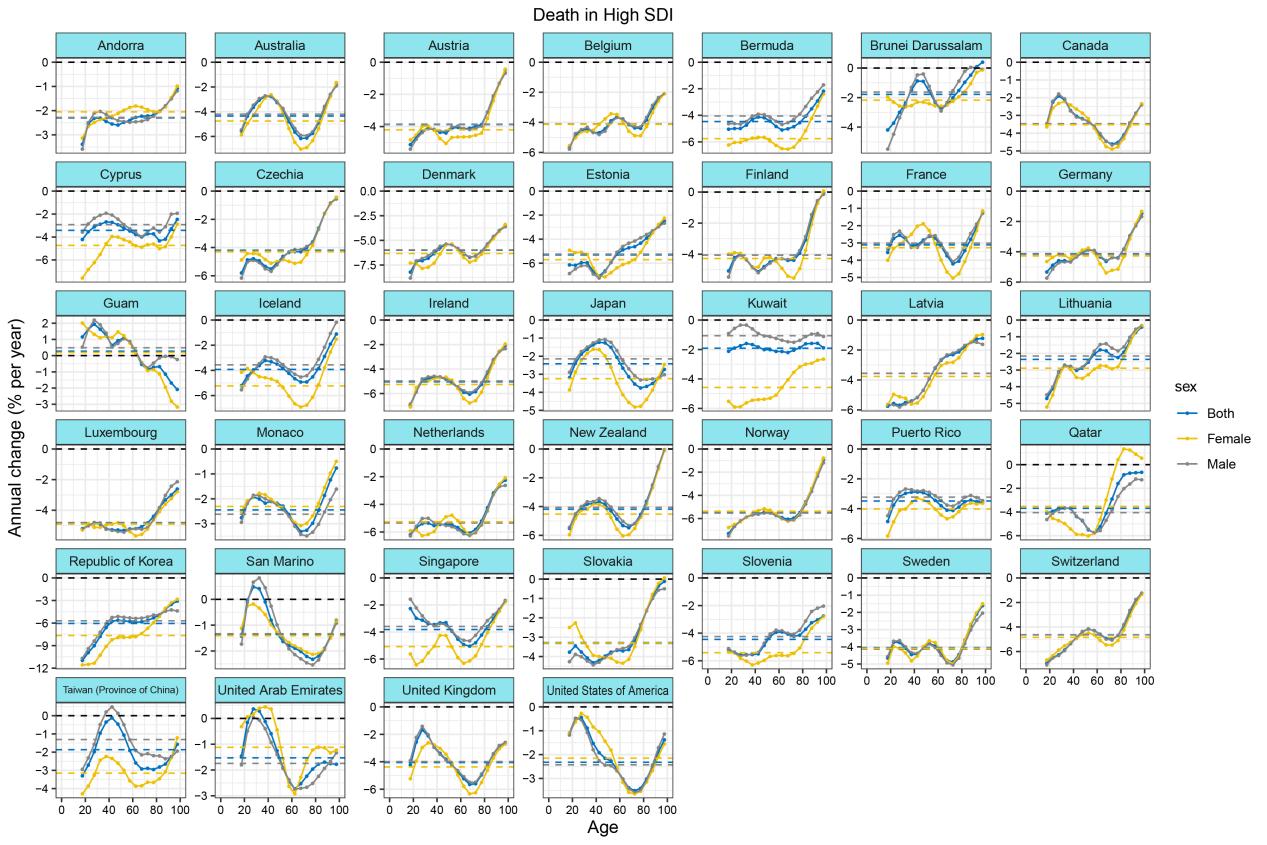


**Figure S3C** Local drifts of ischemic heart disease mortality in high-middle SDI countries, 1990-2019.


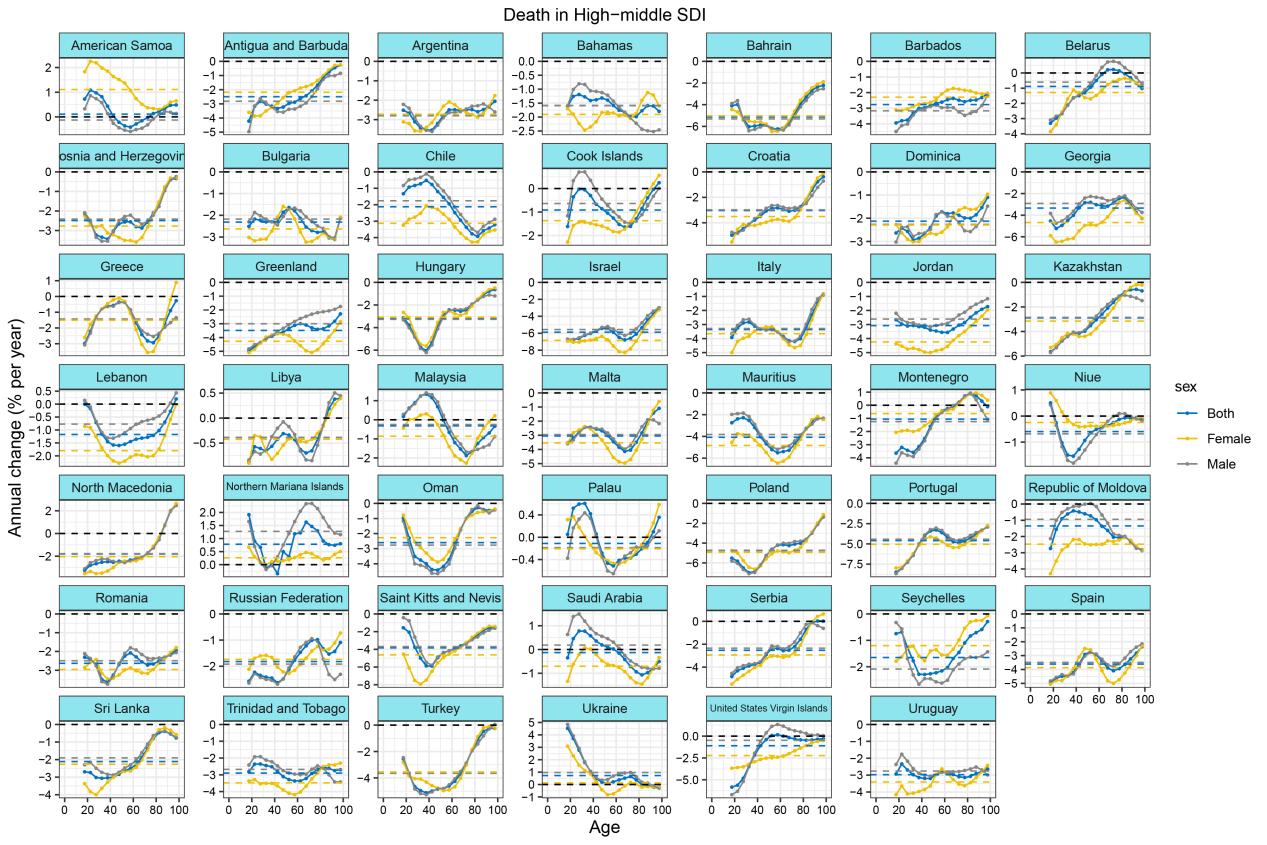


**Figure S3D** The local drifts of ischemic heart disease mortality in middle SDI countries, 1990-2019.


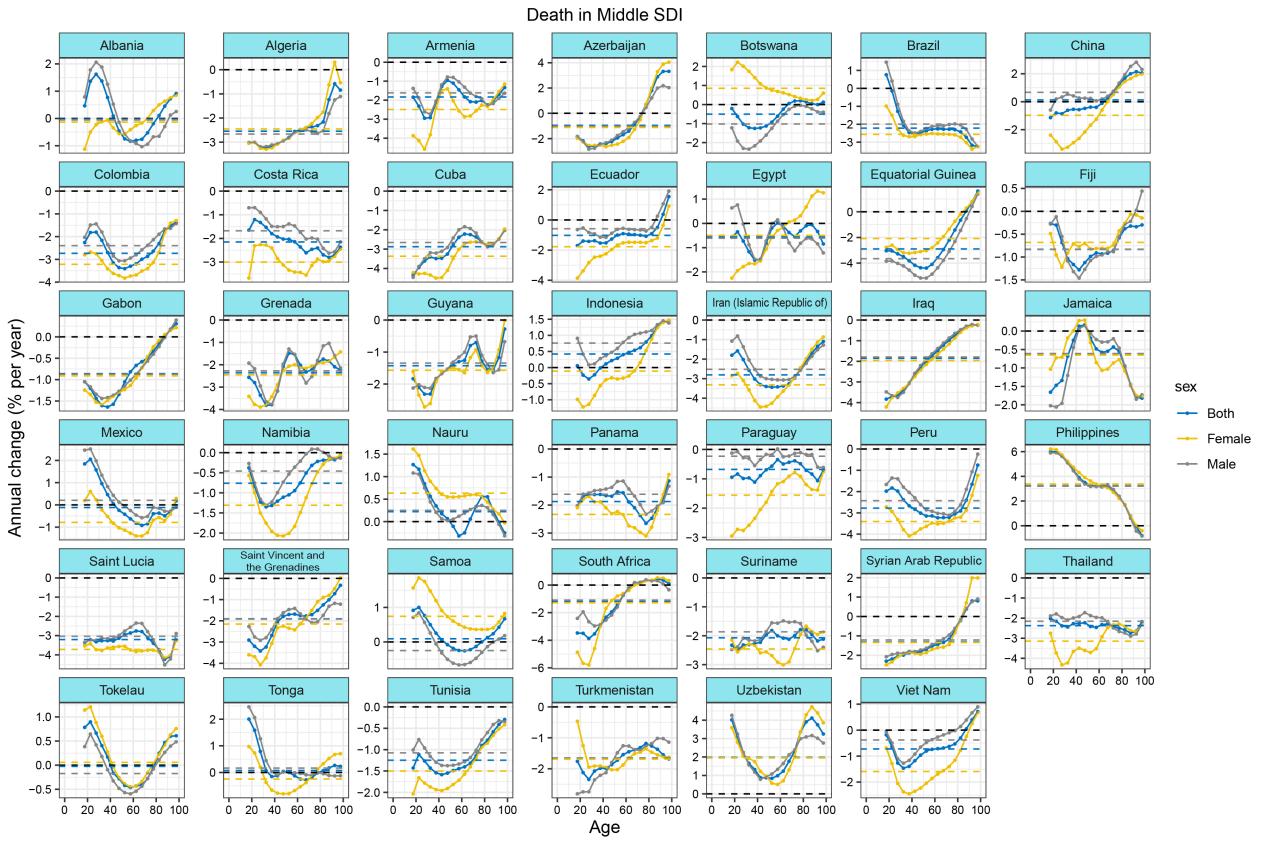


**Figure S3E** The local drifts of ischemic heart disease mortality in low-middle SDI countries, 1990-2019.


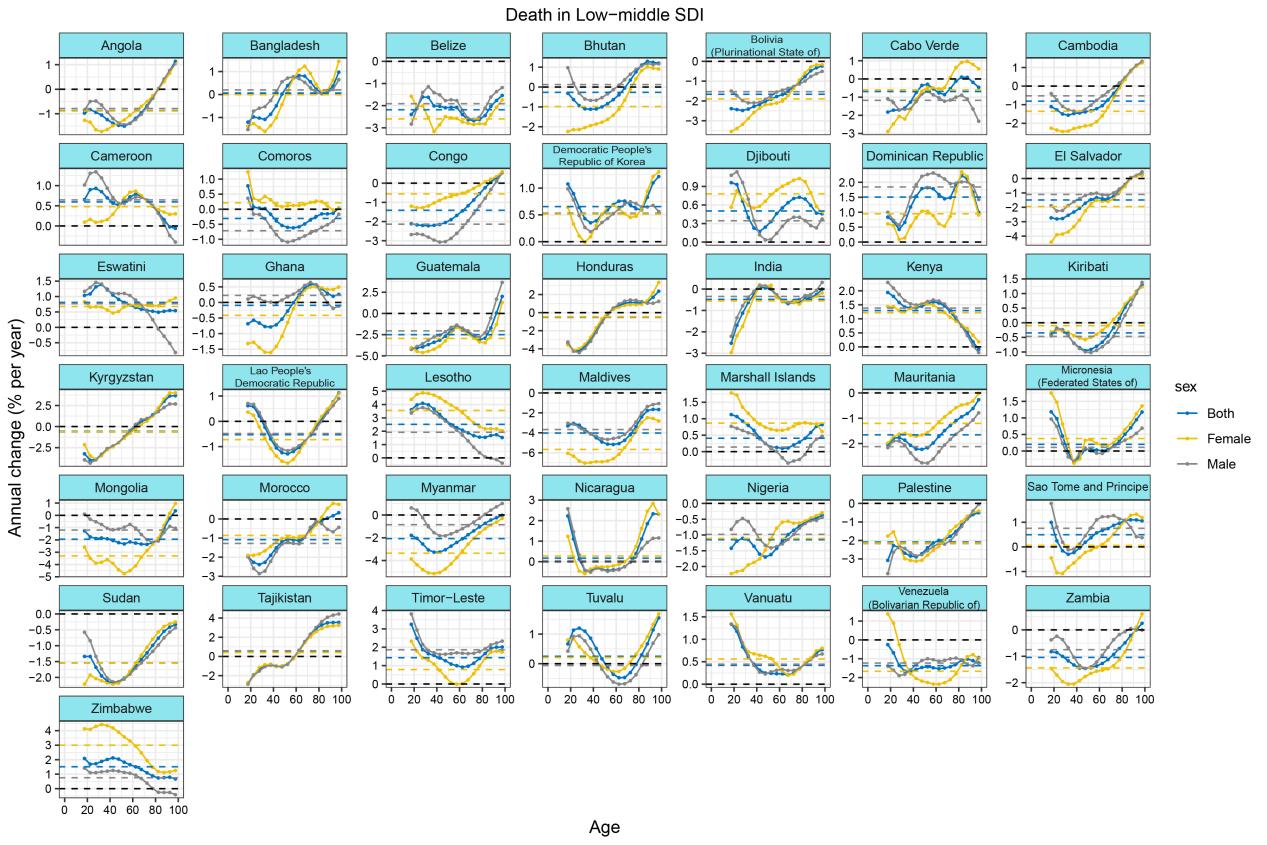


**Figure S3F** The local drifts of ischemic heart disease mortality in low SDI countries, 1990-2019.


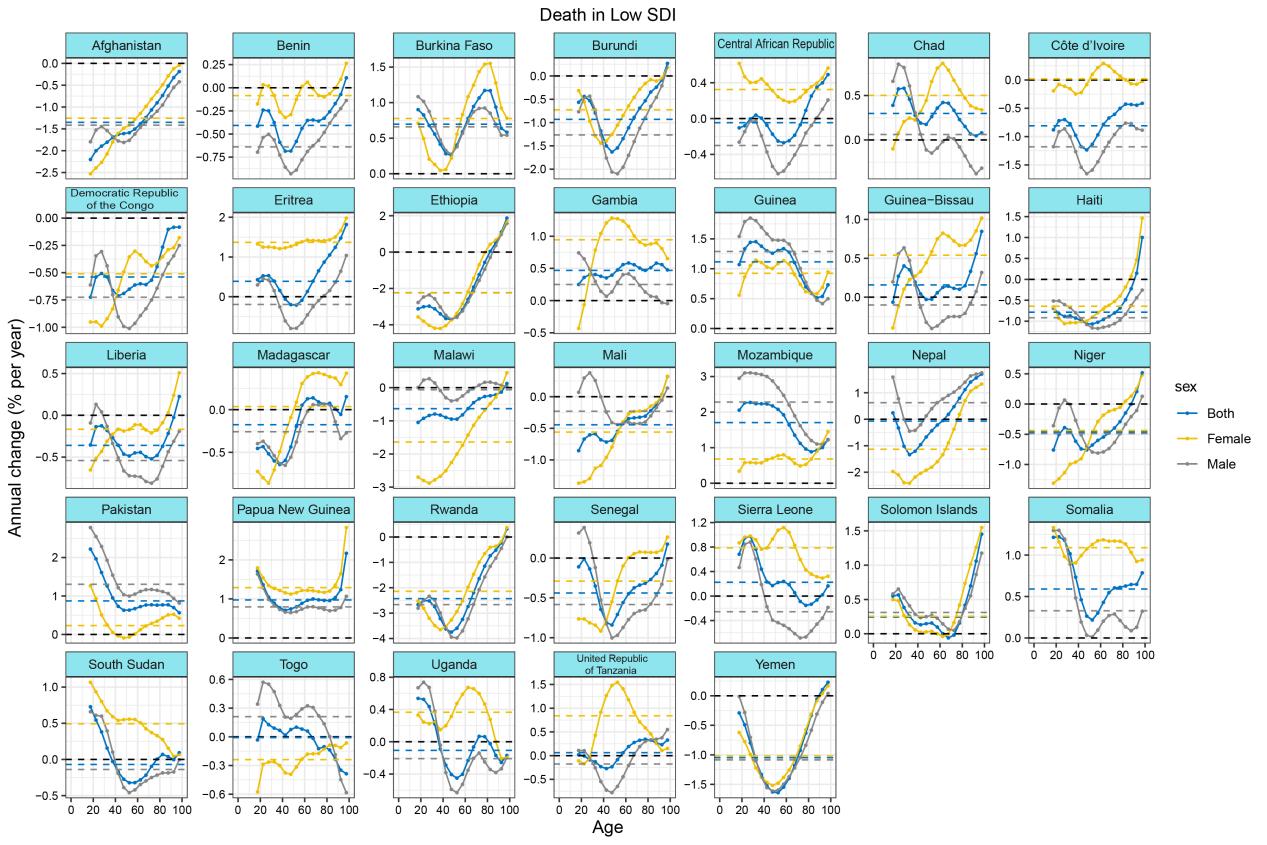


**Figure S4A** Age distribution of absolute cases of ischemic heart disease mortality in 21 GBD regions, 1990-2019.


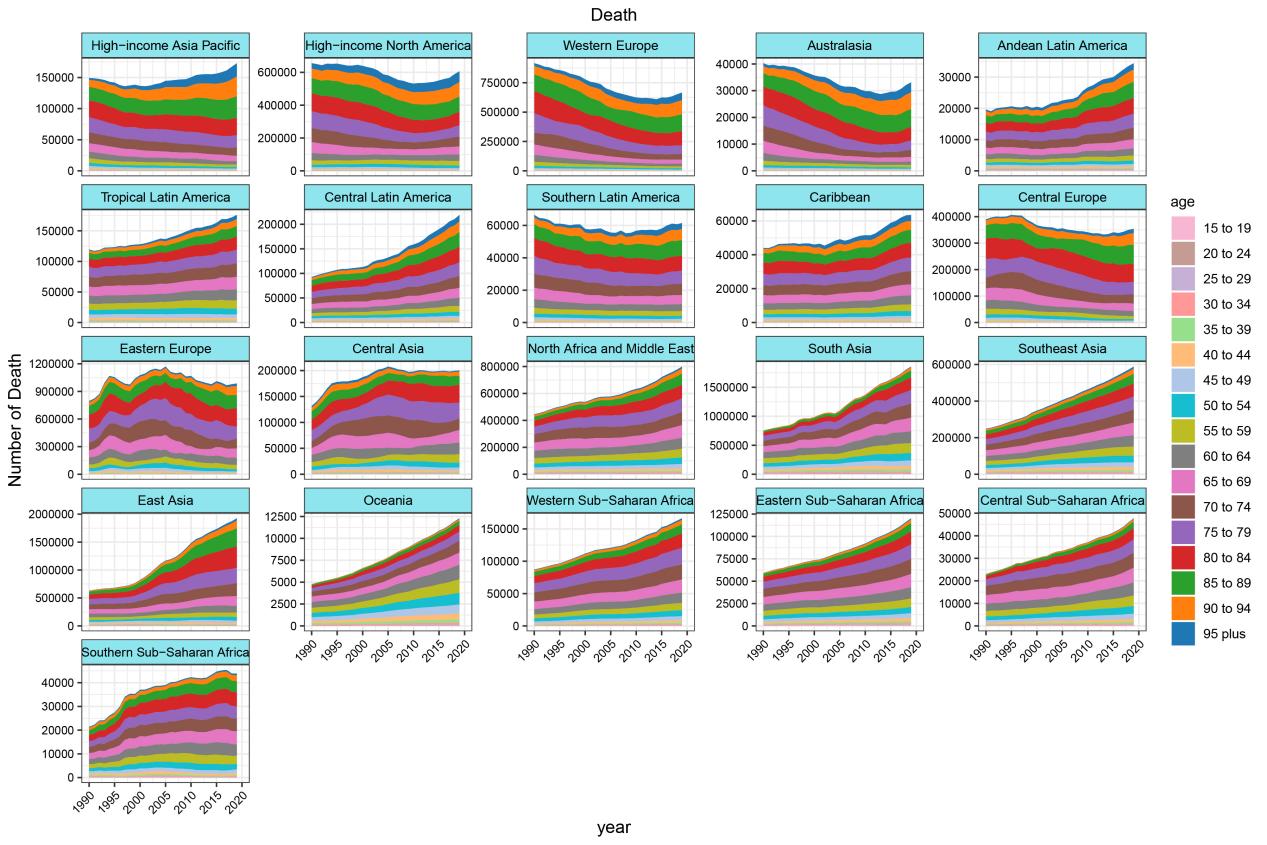


**Figure S4B** Age distribution of absolute cases of ischemic heart disease mortality in high SDI countries, 1990-2019.


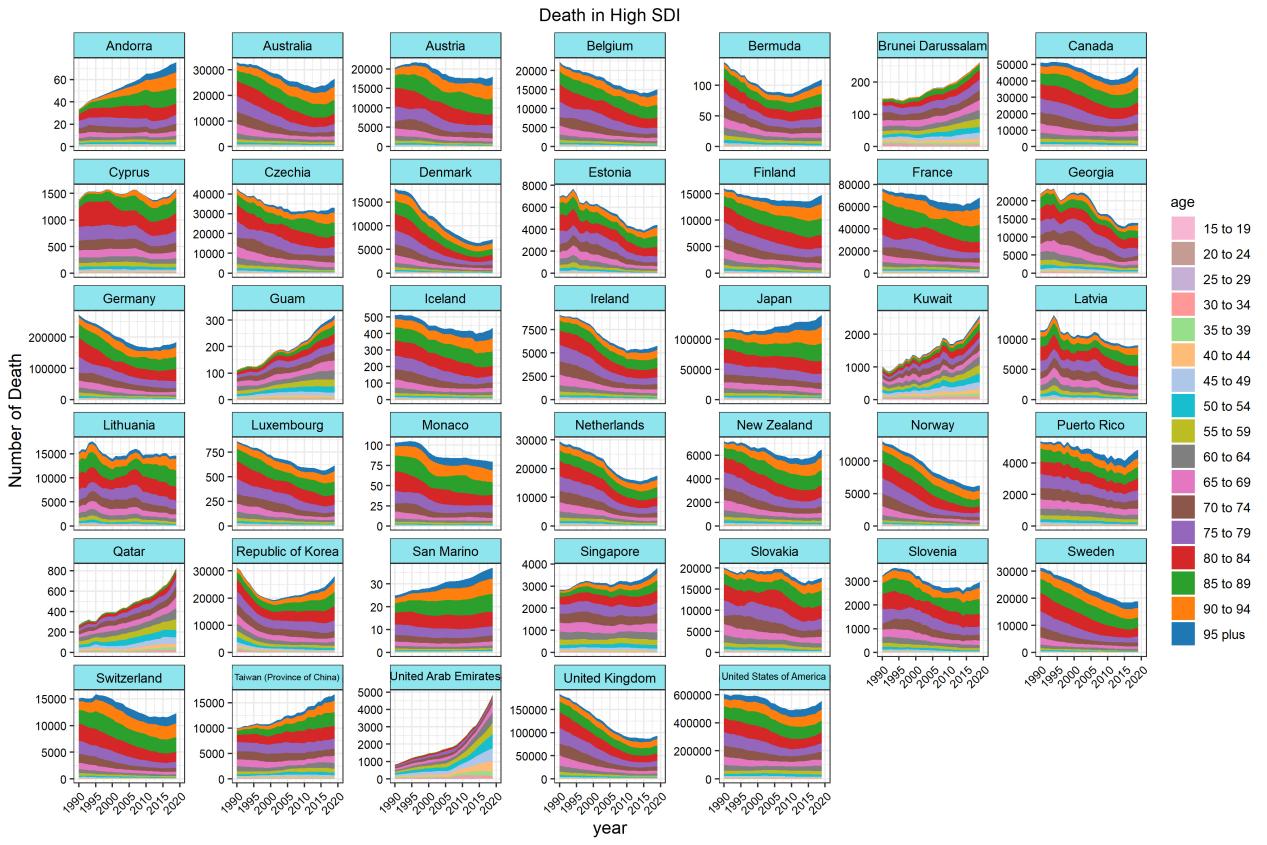


**Figure S4C** Age distribution of absolute cases of ischemic heart disease mortality in high-middle SDI countries, 1990-2019.


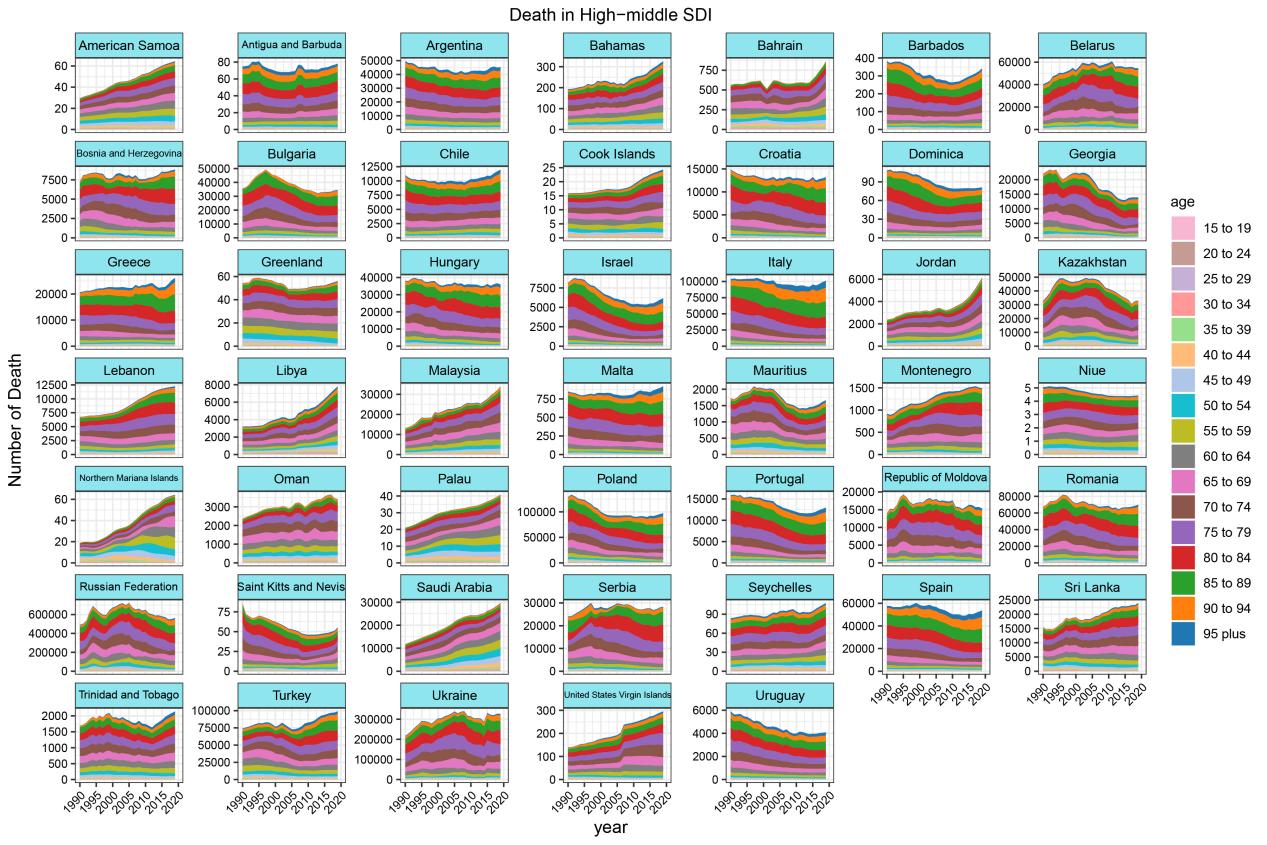


**Figure S4D** Age distribution of absolute cases of ischemic heart disease mortality in middle SDI countries, 1990-2019.


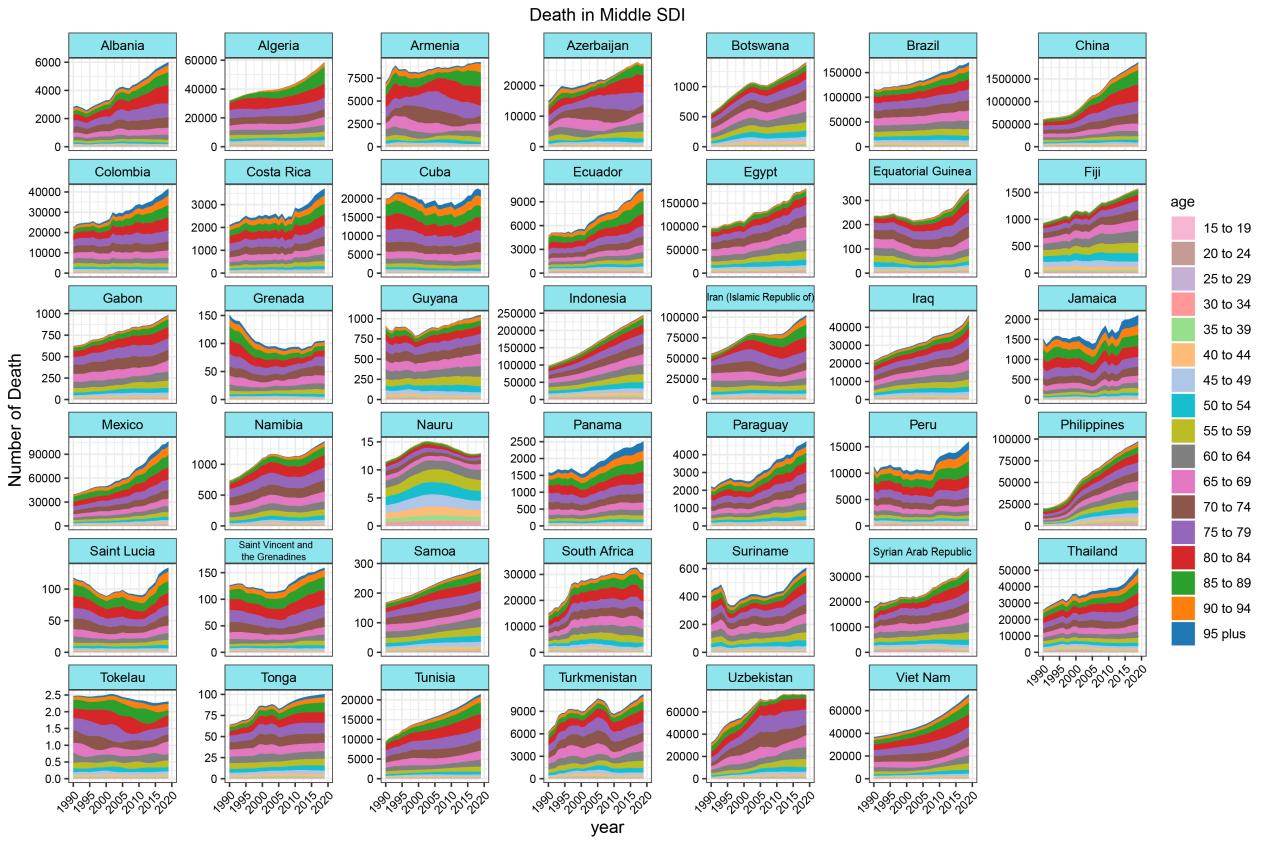


**Figure S4E** Age distribution of absolute cases of ischemic heart disease mortality in low-middle SDI countries, 1990-2019.


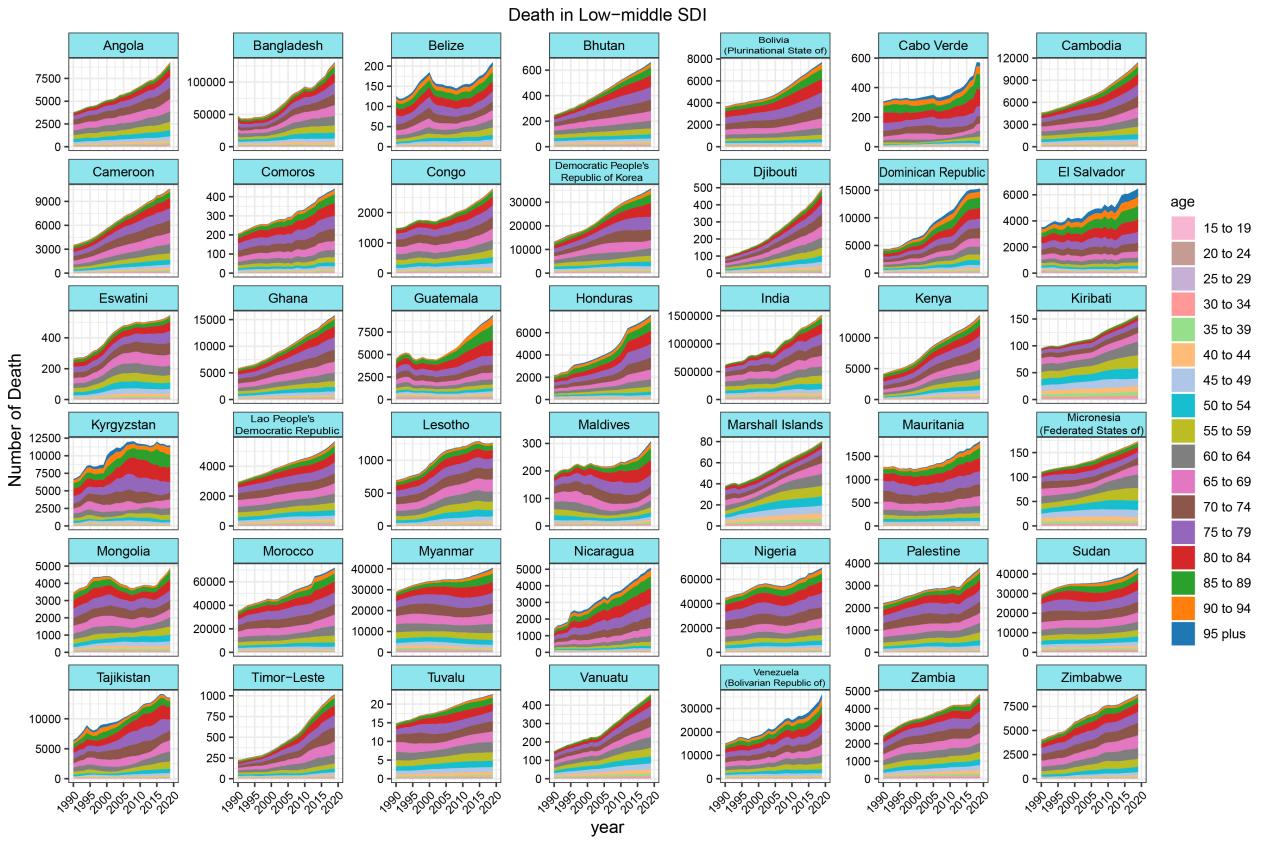


**Figure S4F** Age distribution of absolute cases of ischemic heart disease mortality in low SDI countries, 1990-2019.


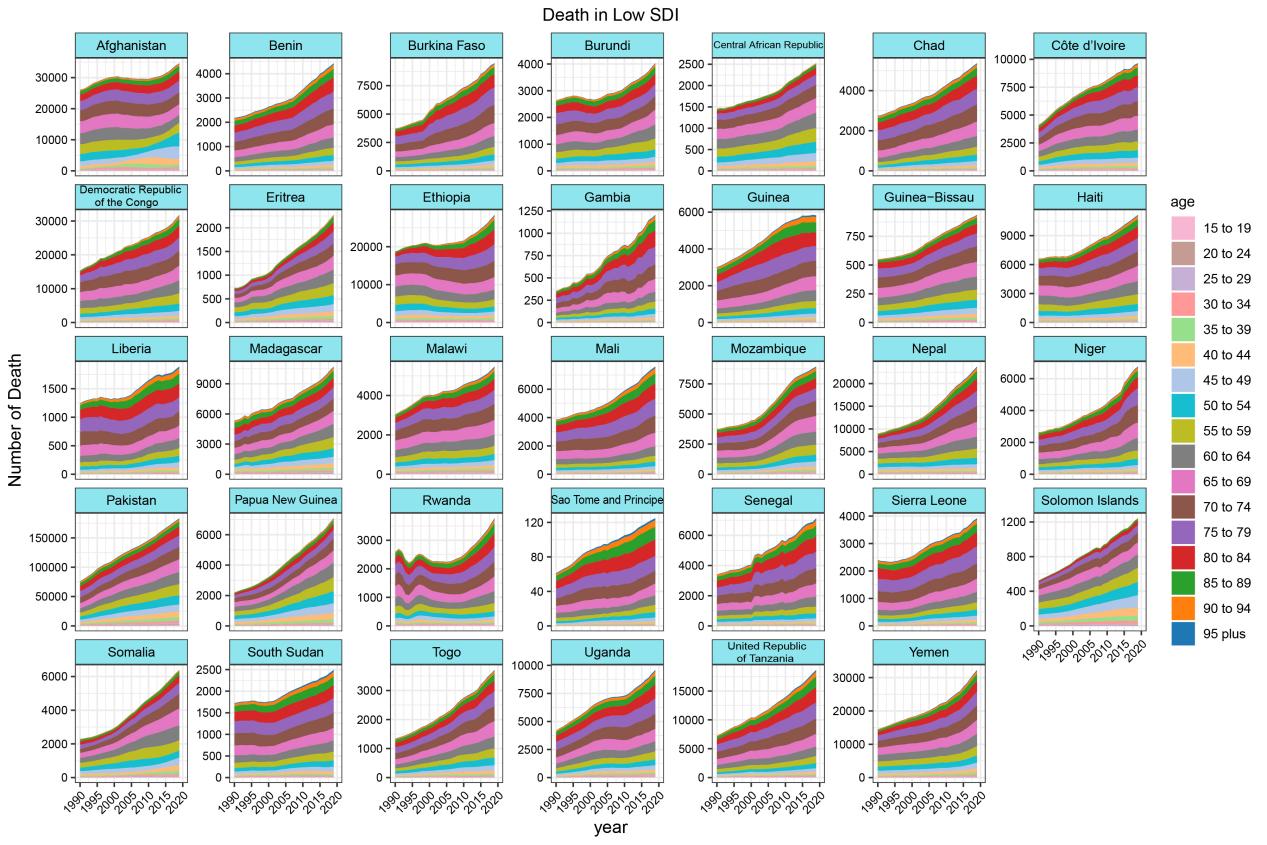


**Figure S5A** Age distribution of relative proportion of ischemic heart disease mortality in 21 GBD regions, 1990-2019


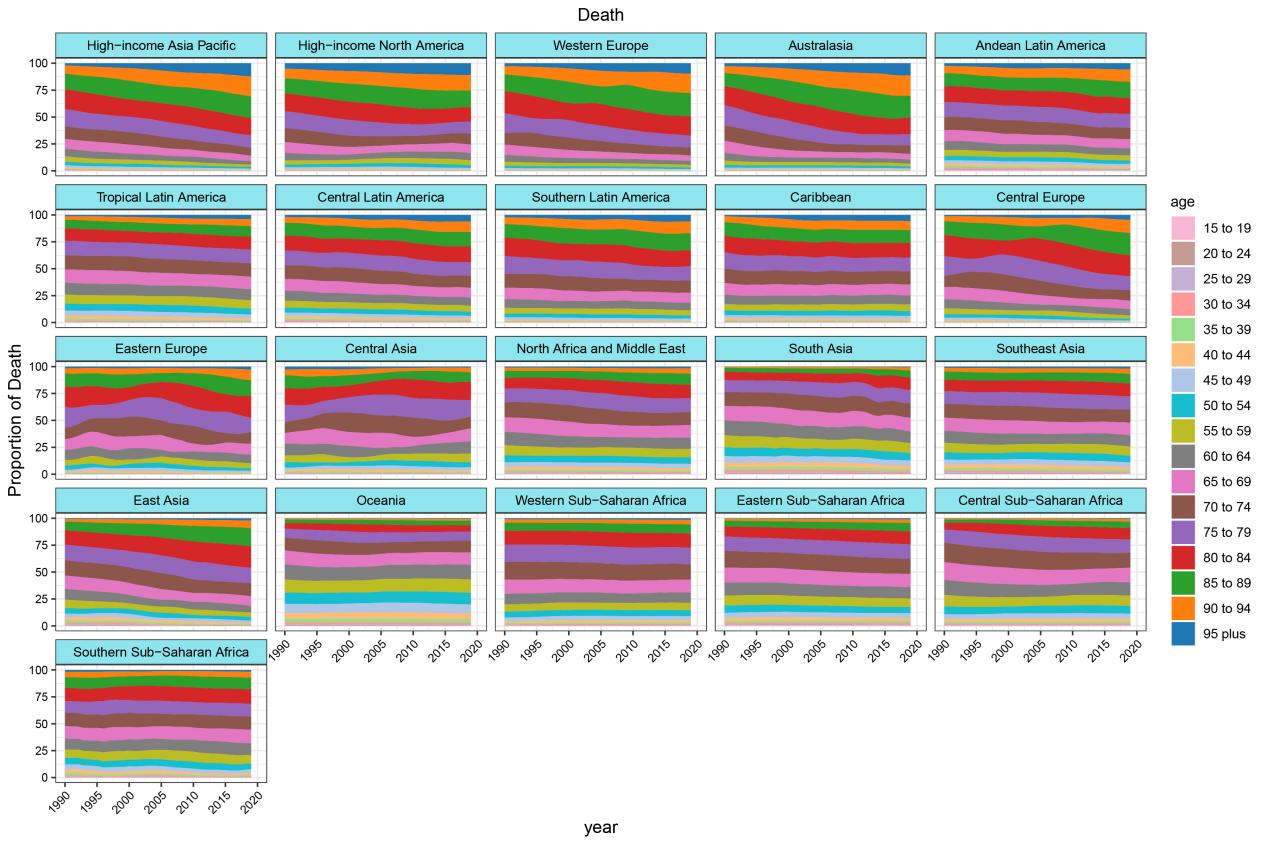


**Figure S5B** Age distribution of relative proportion of ischemic heart disease mortality in high SDI countries, 1990-2019


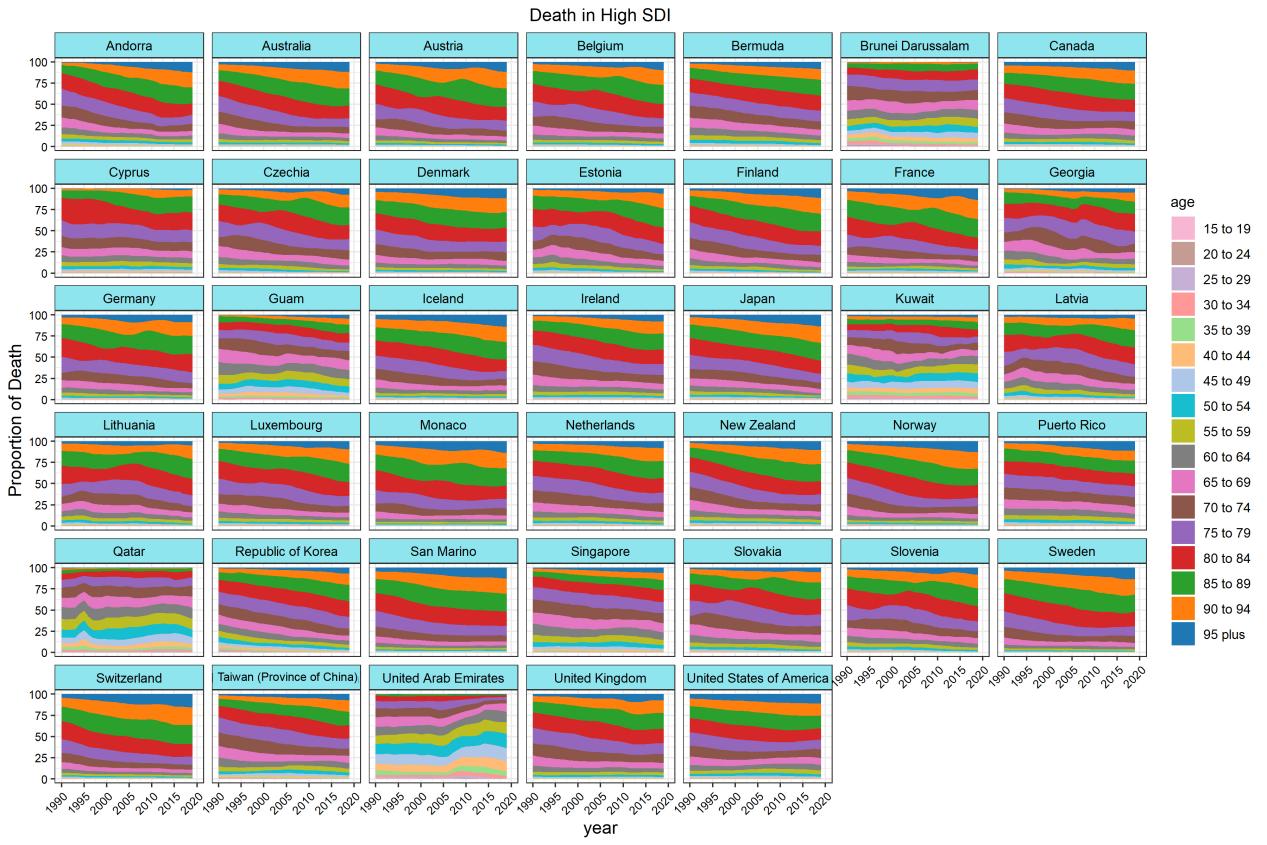


**Figure S5C** Age distribution of relative proportion of ischemic heart disease mortality in high-middle SDI countries, 1990-2019


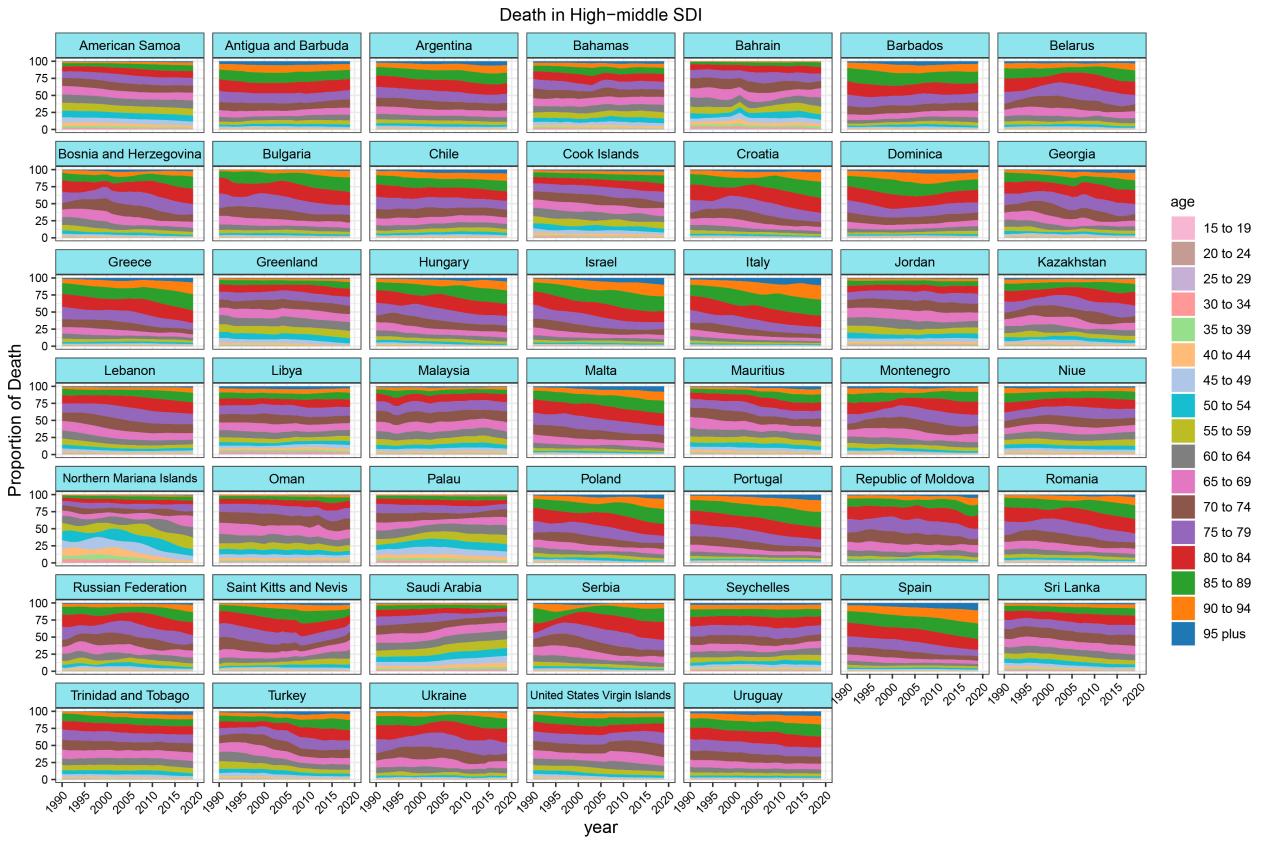


**Figure S5D** Age distribution of relative proportion of ischemic heart disease mortality in middle SDI countries, 1990-2019


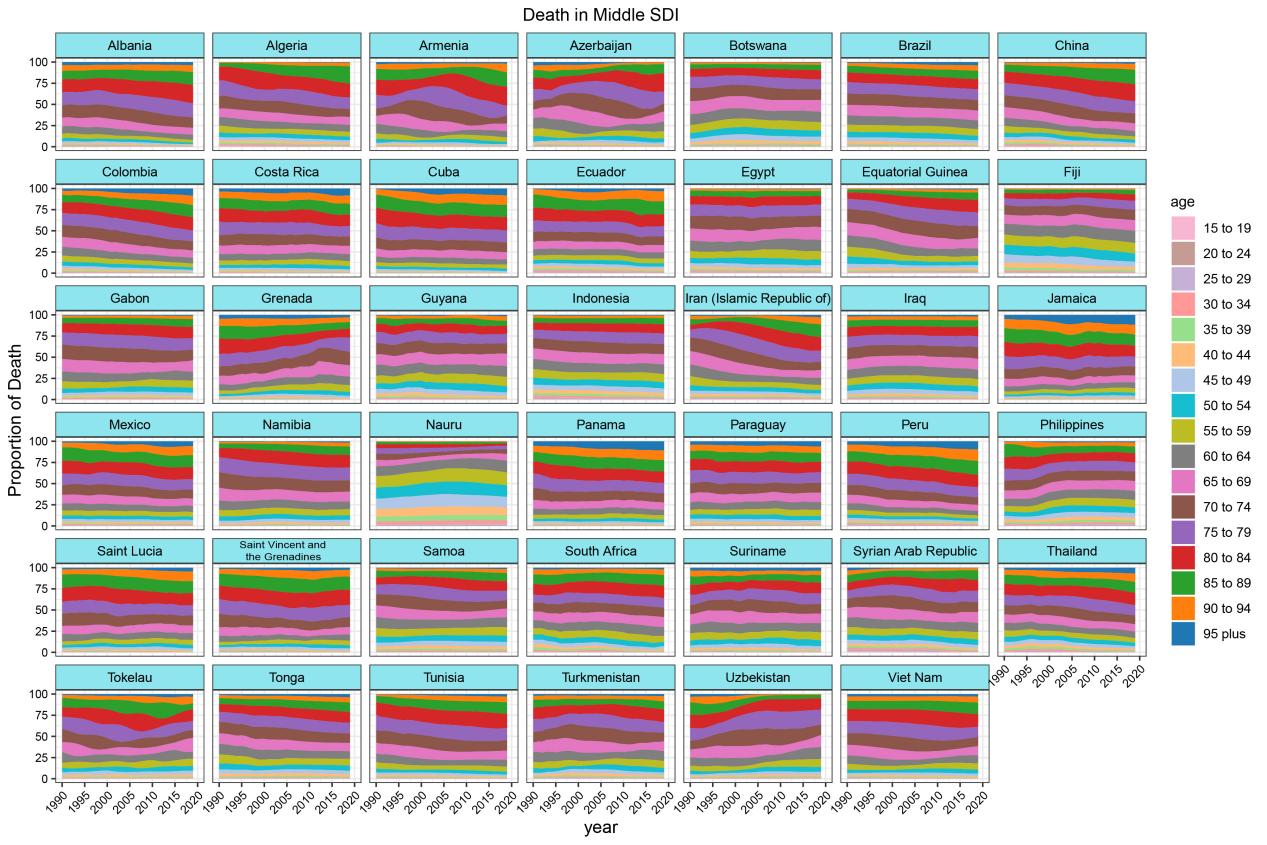


**Figure S5E** Age distribution of relative proportion of ischemic heart disease mortality in low-middle SDI countries, 1990-2019


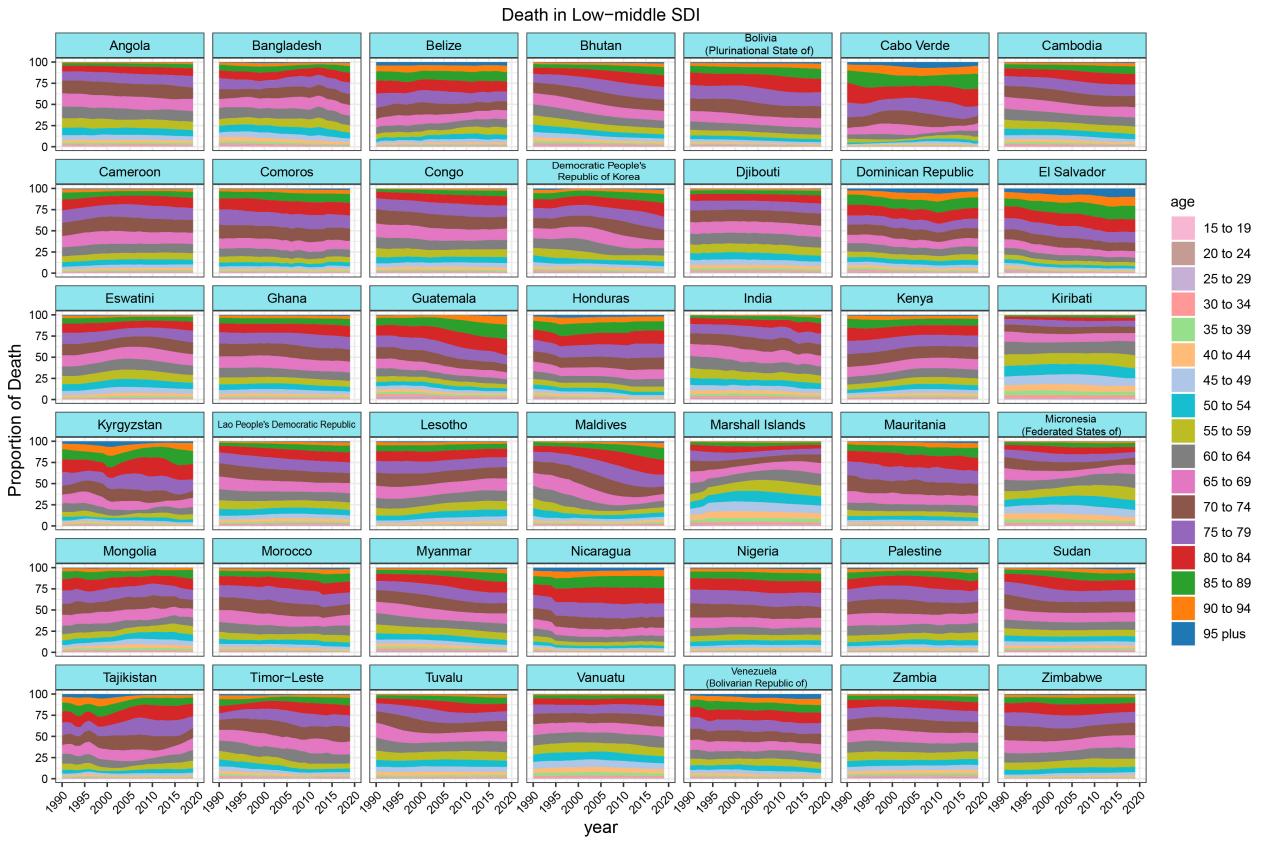


**Figure S5F** Age distribution of relative proportion of ischemic heart disease mortality in low SDI countries, 1990-2019


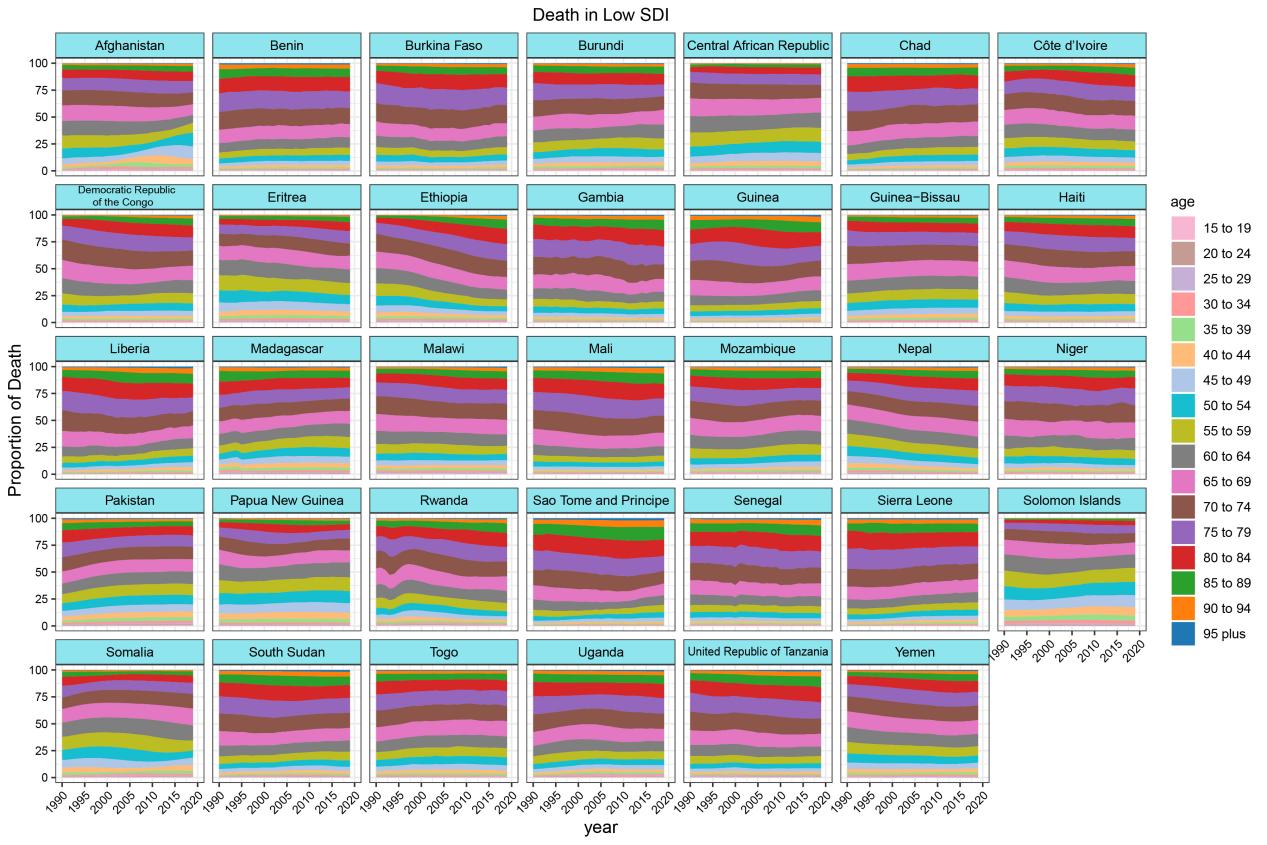


**Figure S6A** Age effects on ischemic heart disease mortality in 21 GBD regions. Age effects are shown by the fitted longitudinal age curves of mortality (per 100,000 person-years) adjusted for period deviations. (B) Period effects are shown by the relative risk of mortality (mortality rate ratio) and computed as the ratio of age-specific rates from 1990−1994 to 2015−2019 (2000−2005 as the referent period).


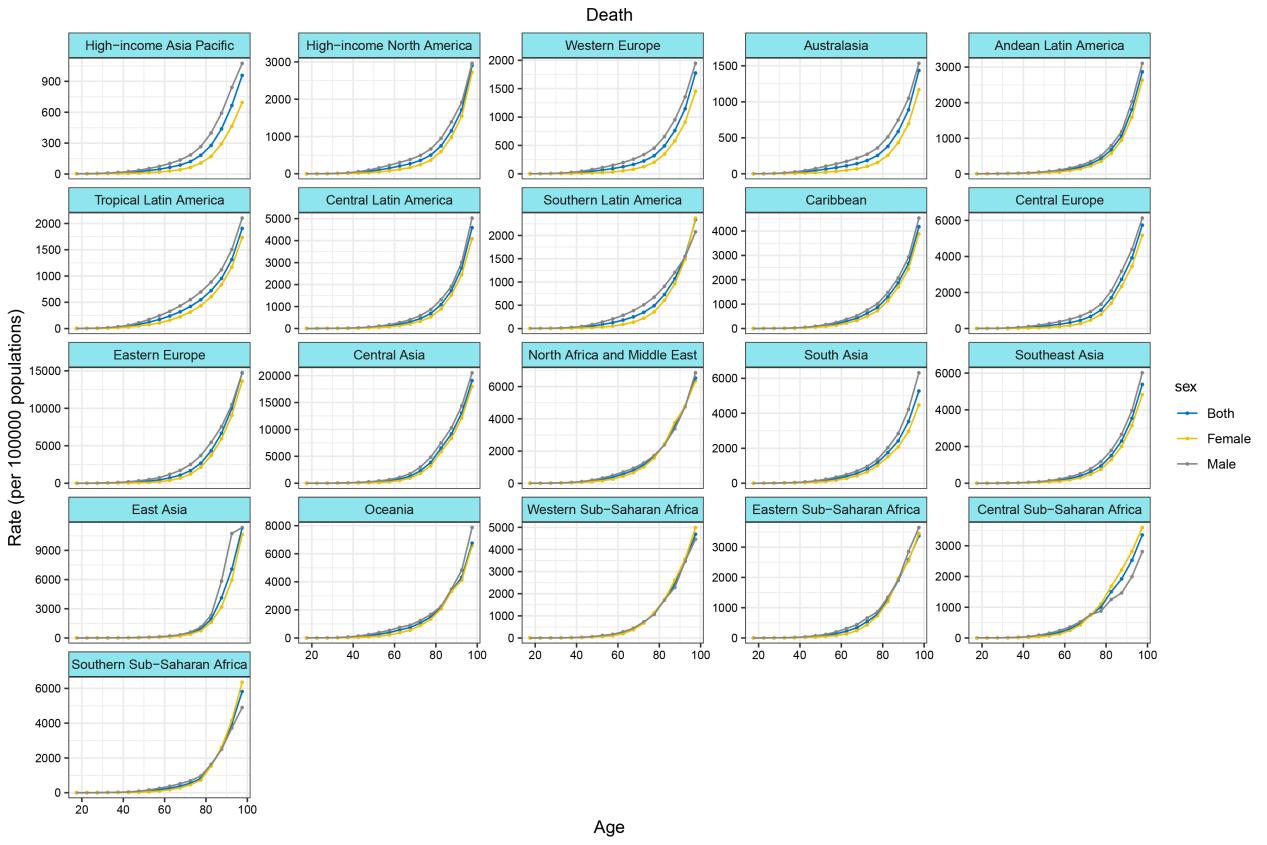


**Figure S6B** Age effects on ischemic heart disease mortality in high SDI countries.


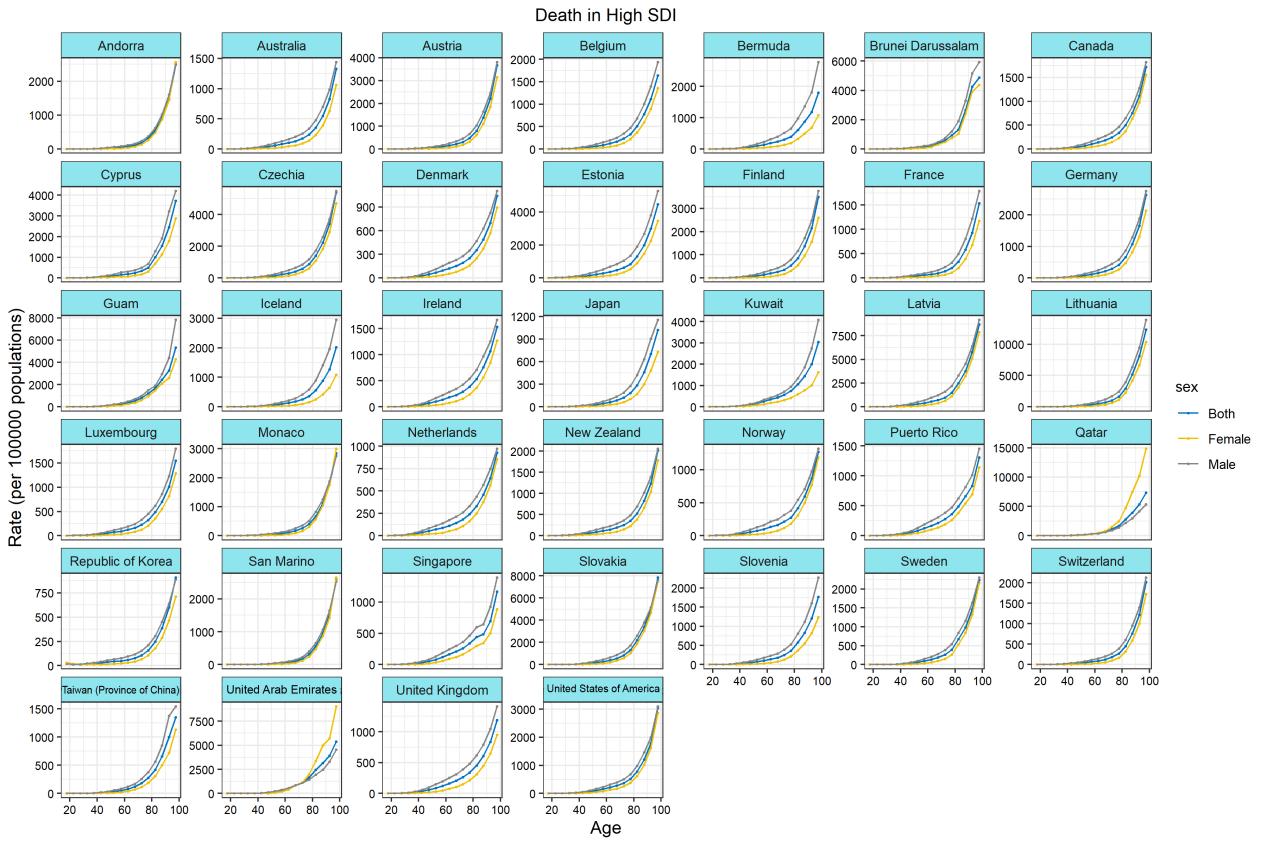


**Figure S6C** Age effects on ischemic heart disease mortality in high-middle SDI countries.


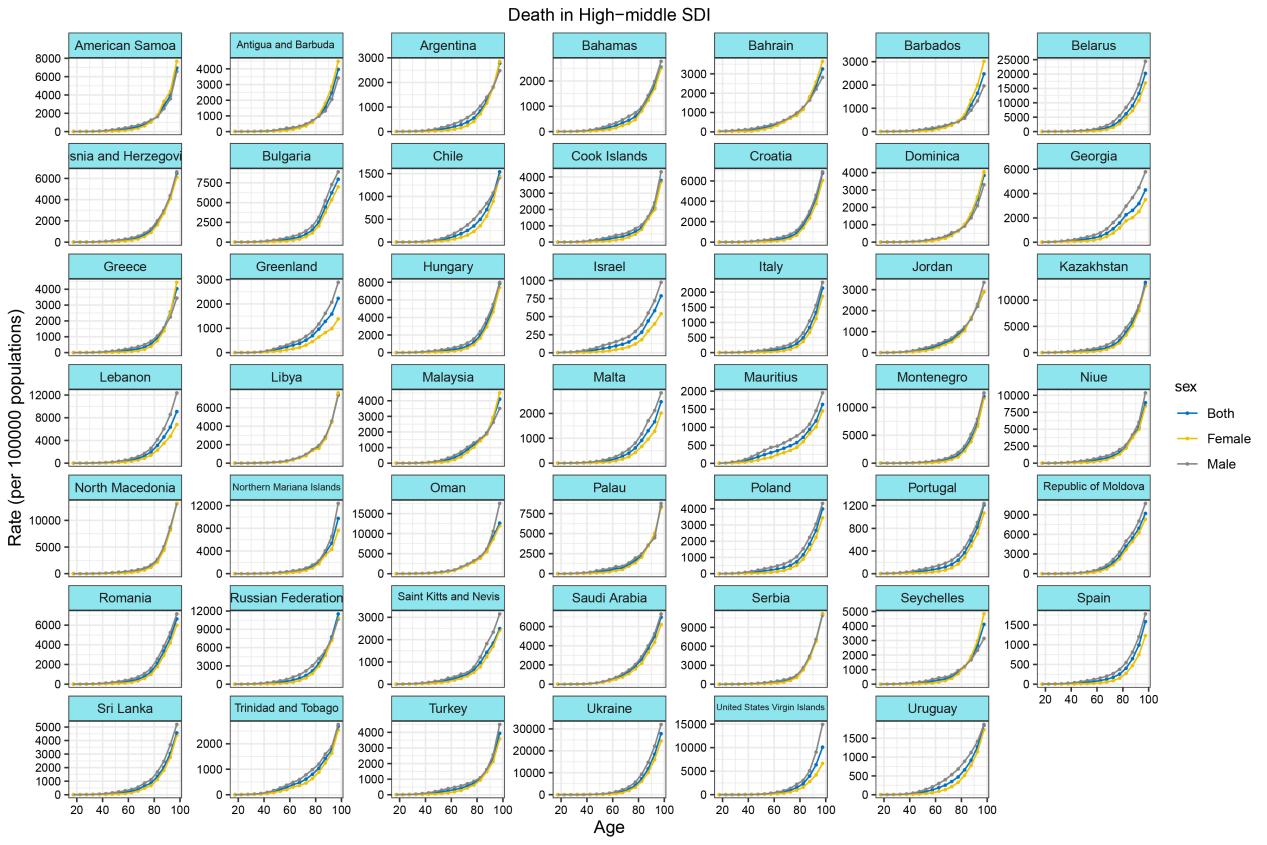


**Figure S6D** Age effects on ischemic heart disease mortality in middle SDI countries.


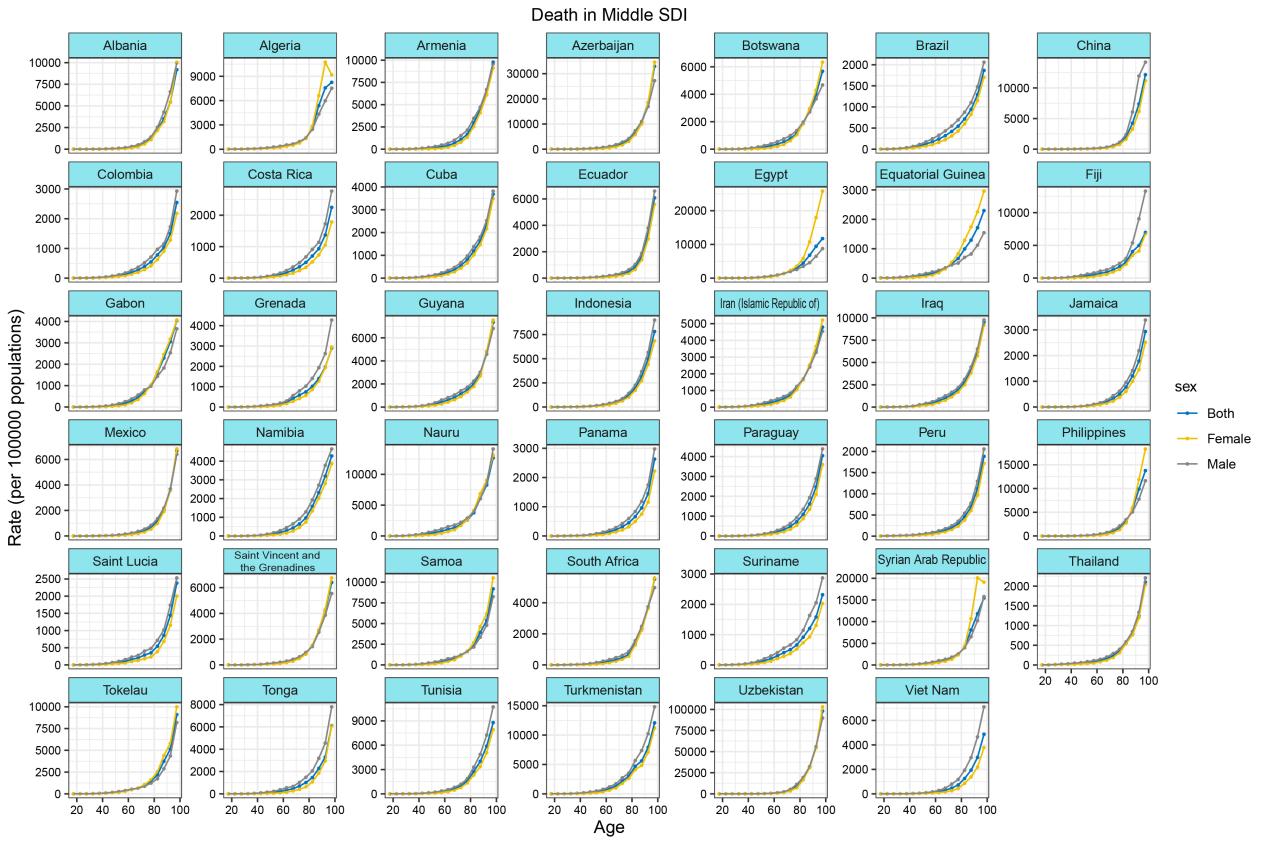


**Figure S6E** Age effects on ischemic heart disease mortality in low-middle SDI countries.


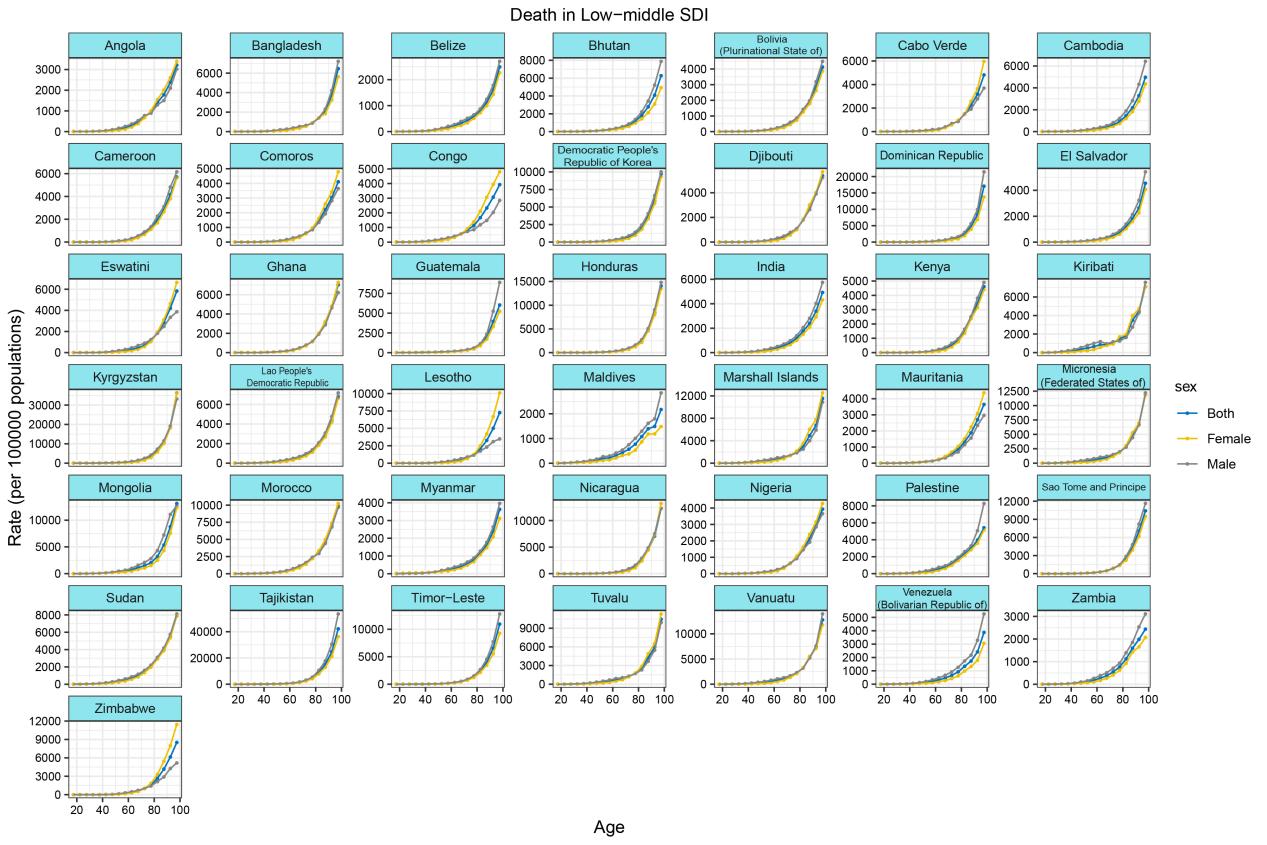


**Figure S6F** Age effects on ischemic heart disease mortality in low SDI countries.


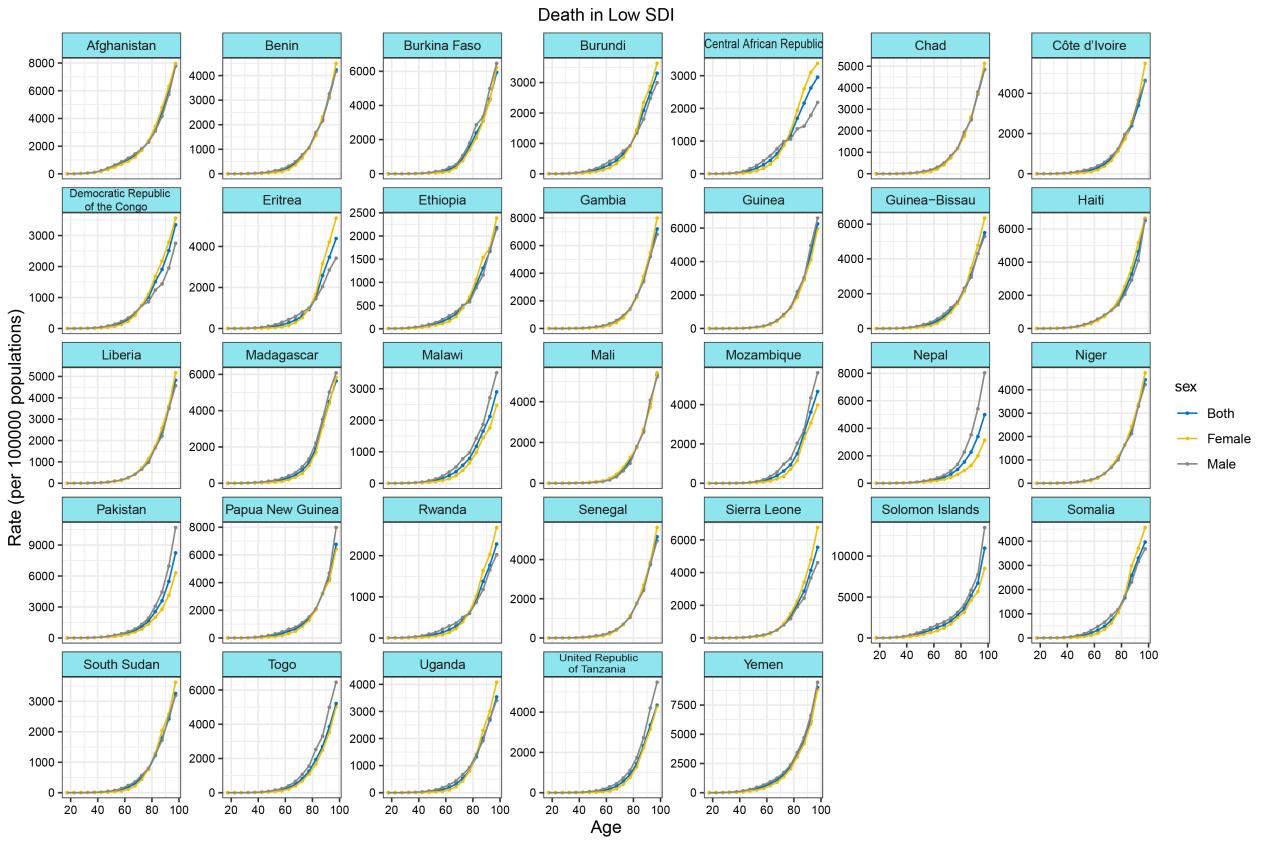


**Figure S7A** Period effects on ischemic heart disease mortality in 21 GBD regions. Period effects are shown by the relative risk of mortality (mortality rate ratio) and computed as the ratio of age-specific rates from 1990−1994 to 2015−2019 (2000−2005 as the referent period). The dots and shaded areas denote mortality rates or rate ratios and their corresponding 95% CIs.


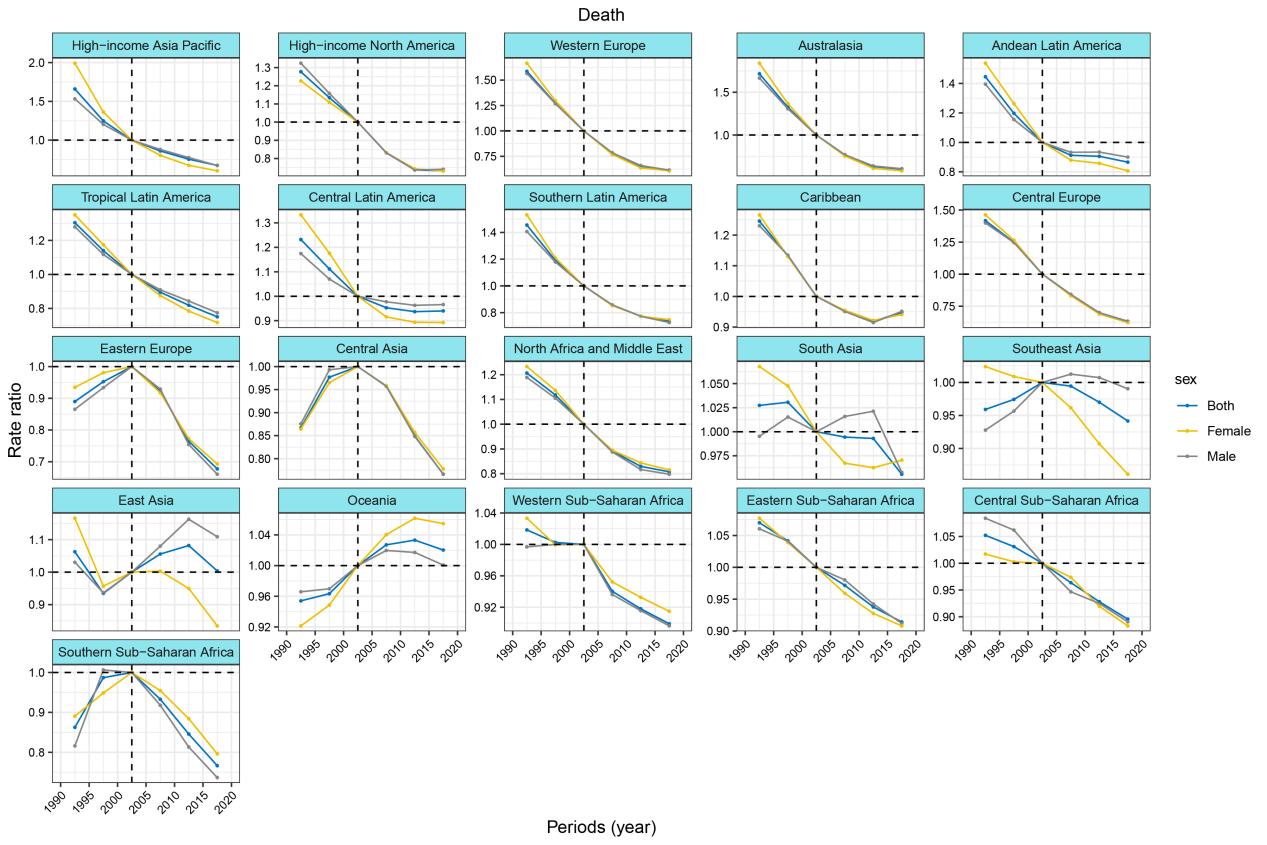


**Figure S7B** Period effects on ischemic heart disease mortality in high SDI countries.


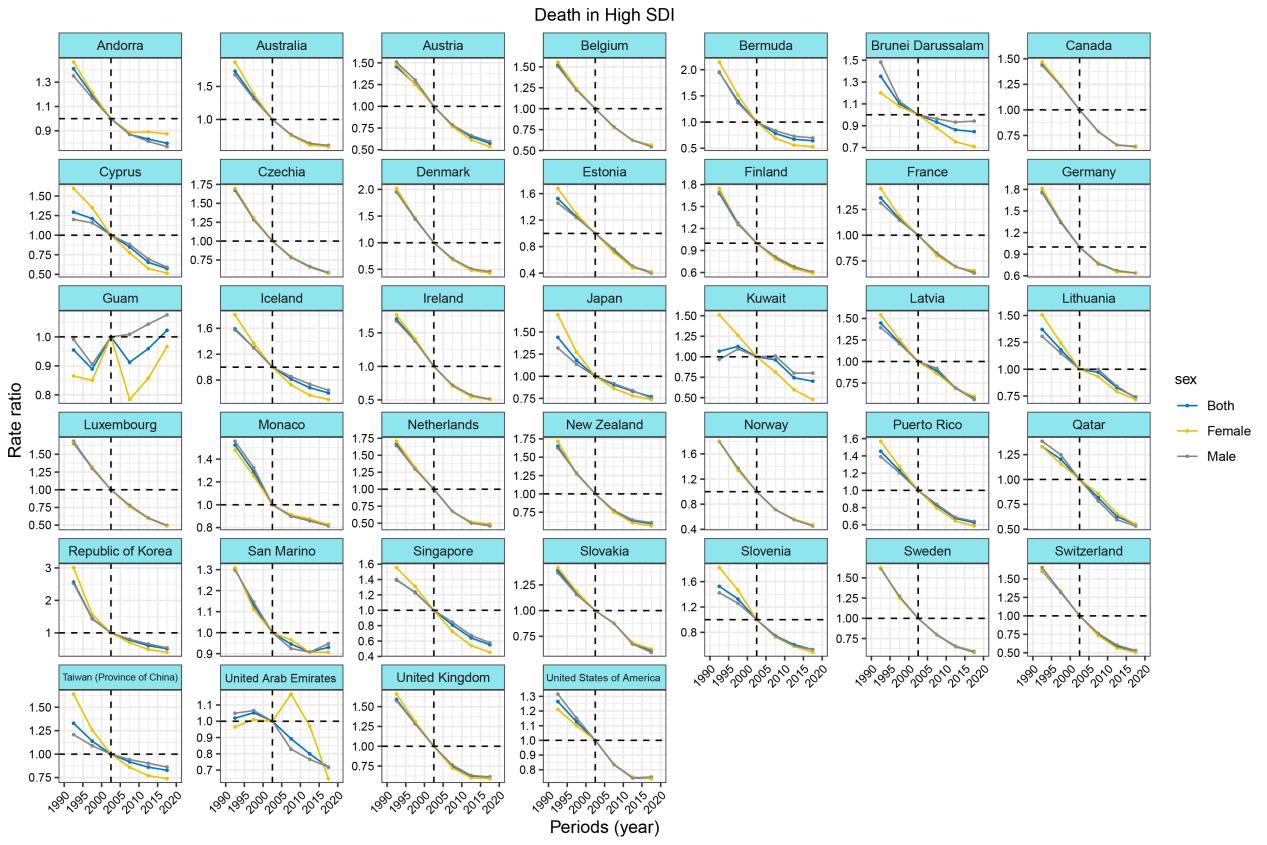


**Figure S7C** Period effects on ischemic heart disease mortality in high-middle SDI countries.


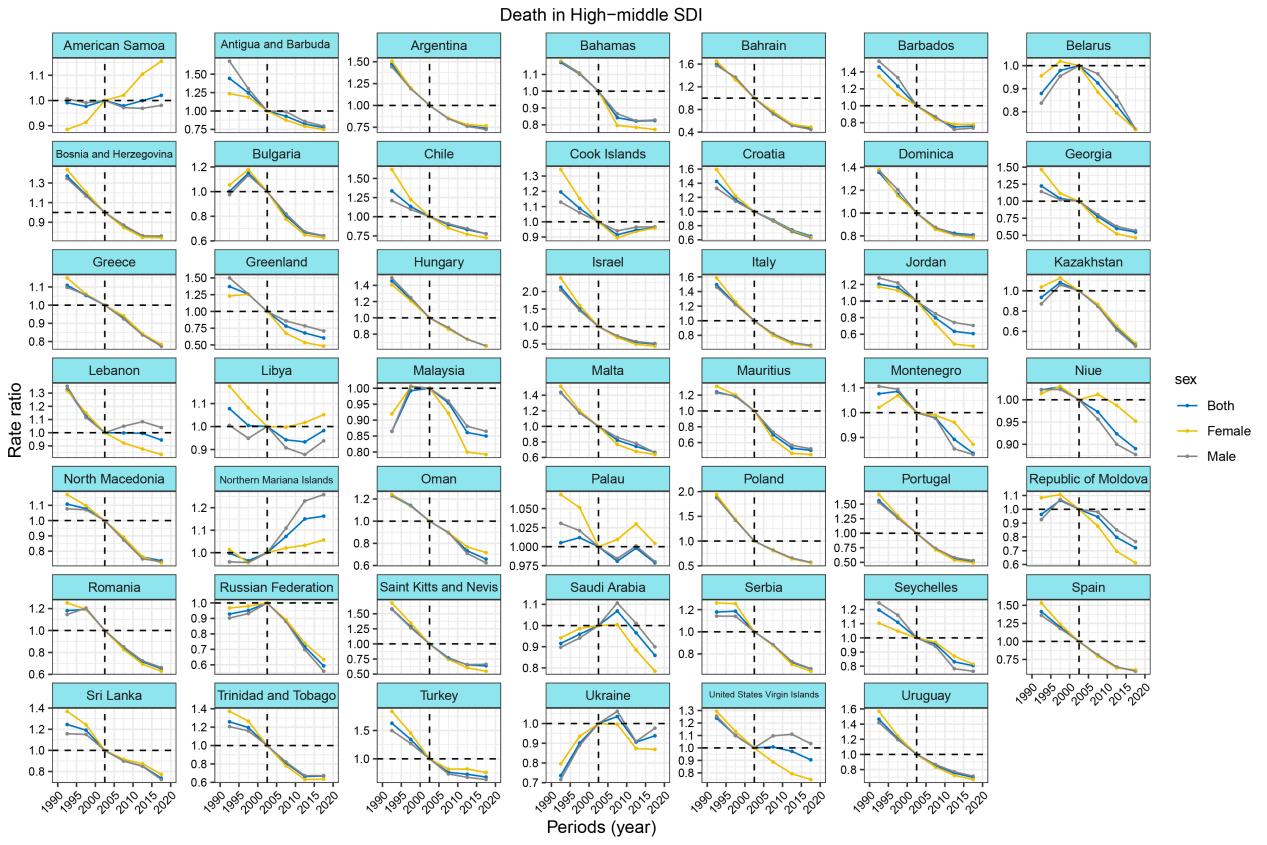


**Figure S7D** Period effects on ischemic heart disease mortality in middle SDI countries.


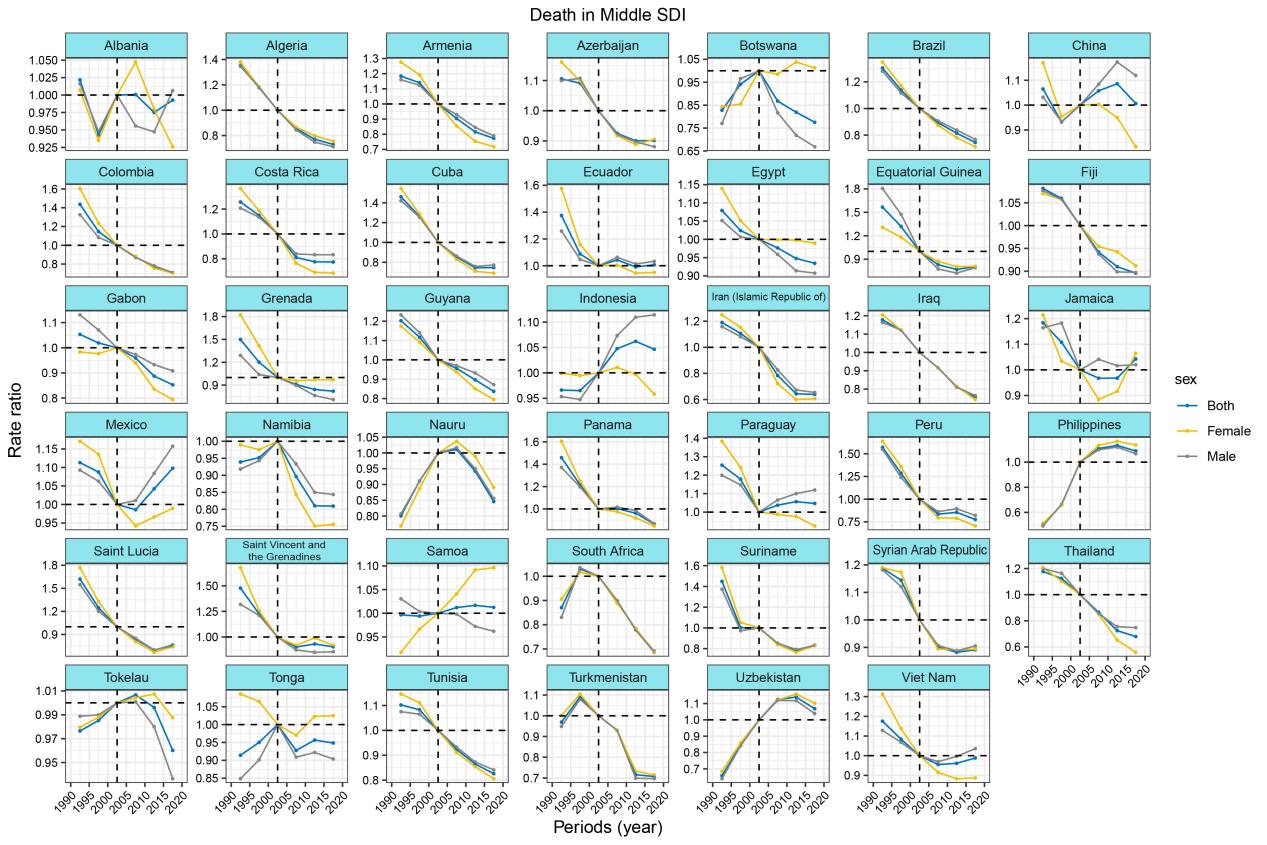


**Figure S7E** Period effects on ischemic heart disease mortality in low-middle SDI countries.


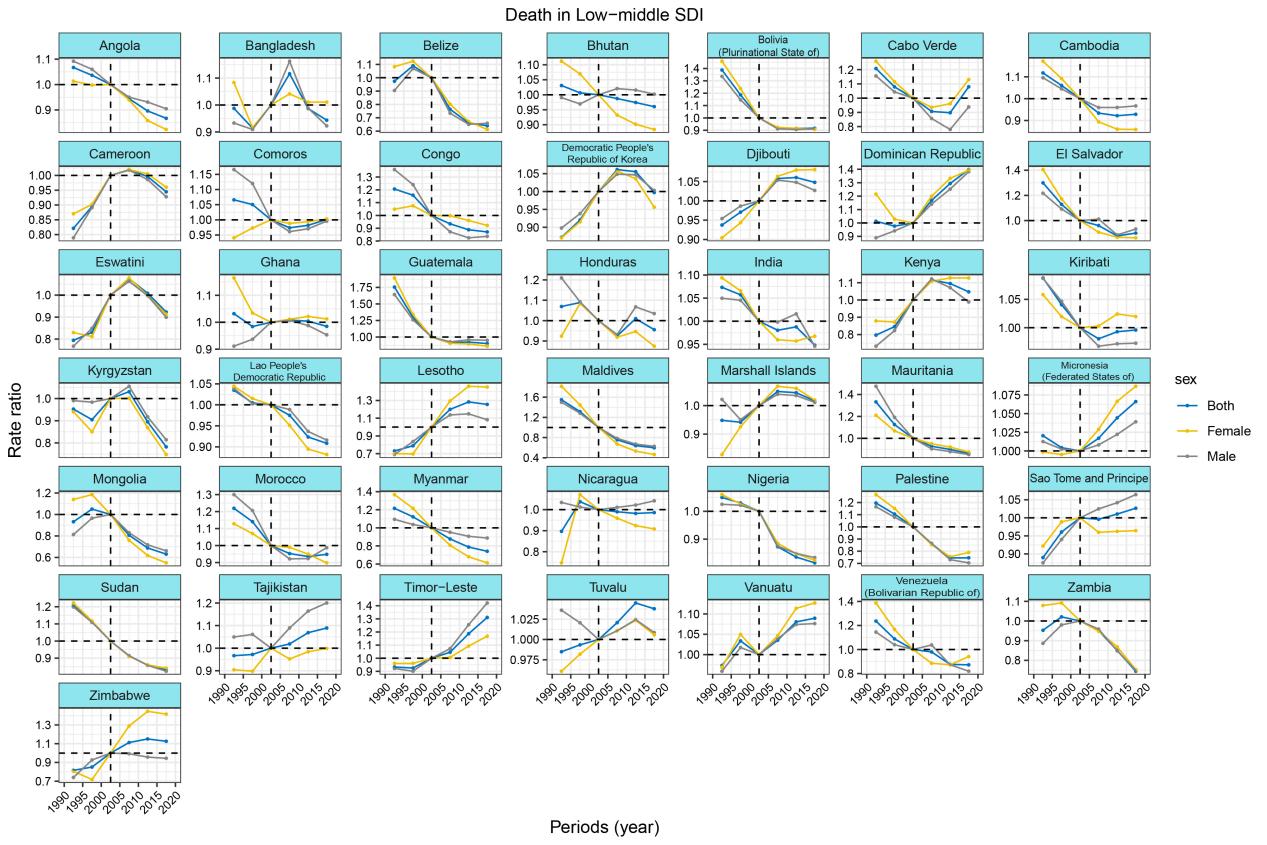


**Figure S7F** Period effects on ischemic heart disease mortality in low SDI countries.


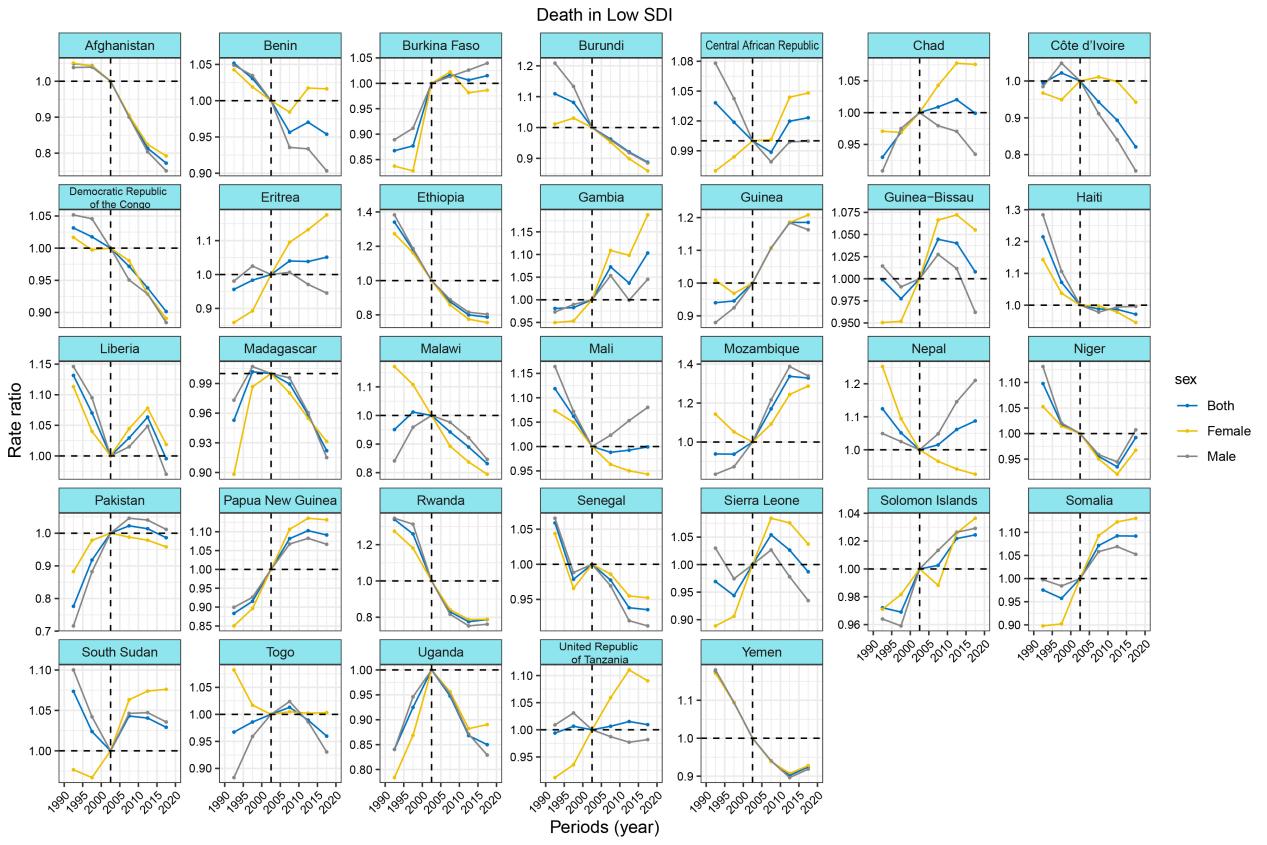


**Figure S8A** Cohort effects on ischemic heart disease mortality in 21 GBD regions. Cohort effects are shown by the relative risk of mortality and computed as the ratio of age-specific rates from the 1895 cohort to the 2000 cohort, with the referent cohort set at 1945. The dots and shaded areas denote mortality rates or rate ratios and their corresponding 95% CIs.


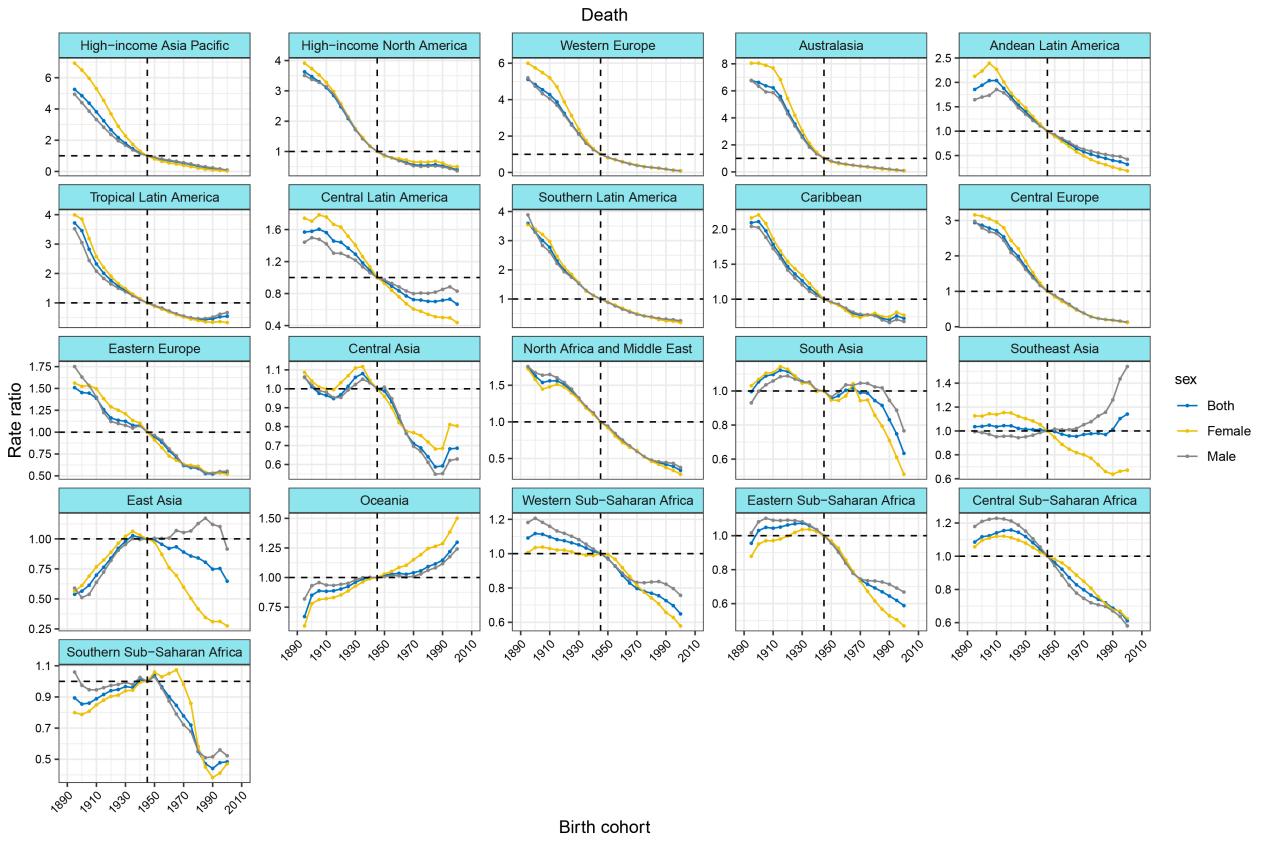


**Figure S8B** Cohort effects on ischemic heart disease mortality in high SDI countries.


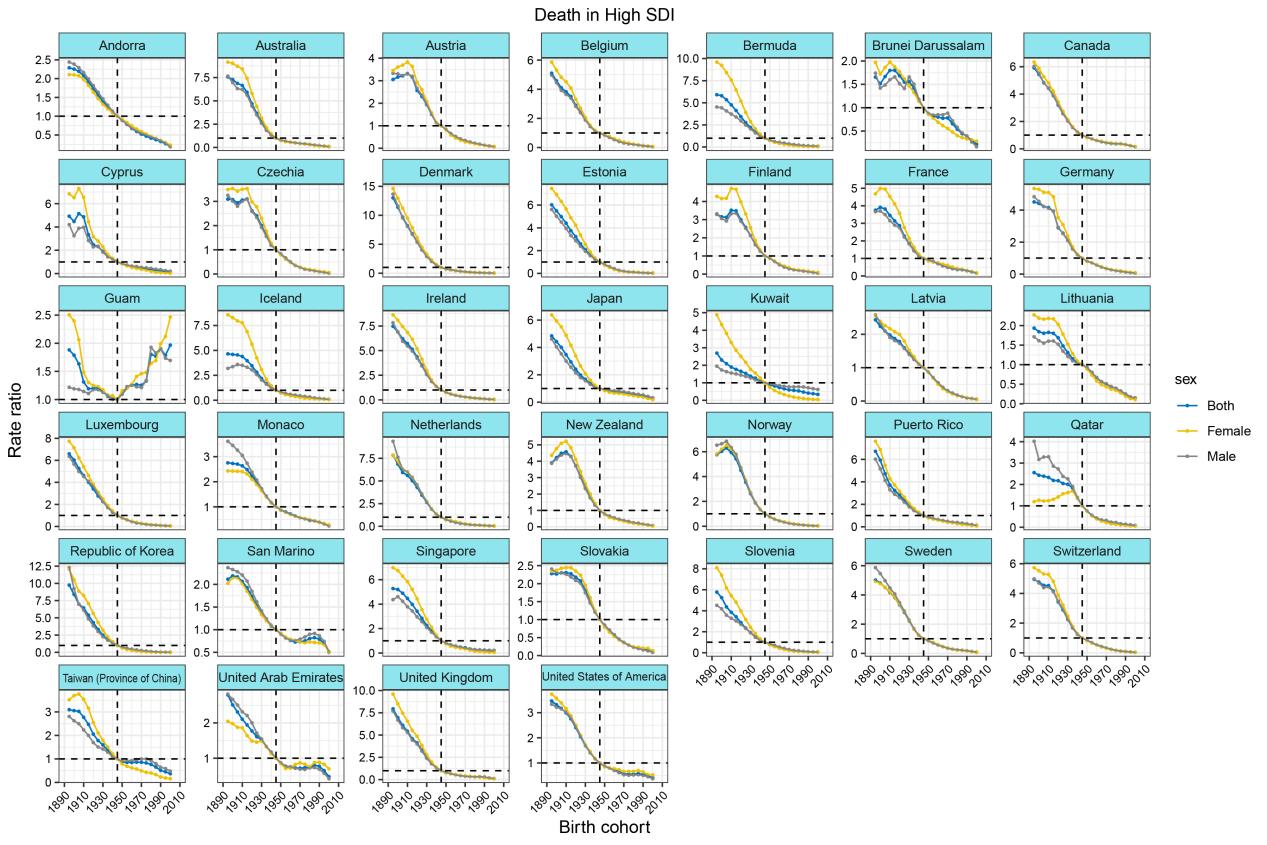


**Figure S8C** Cohort effects on ischemic heart disease mortality in high-middle SDI countries.


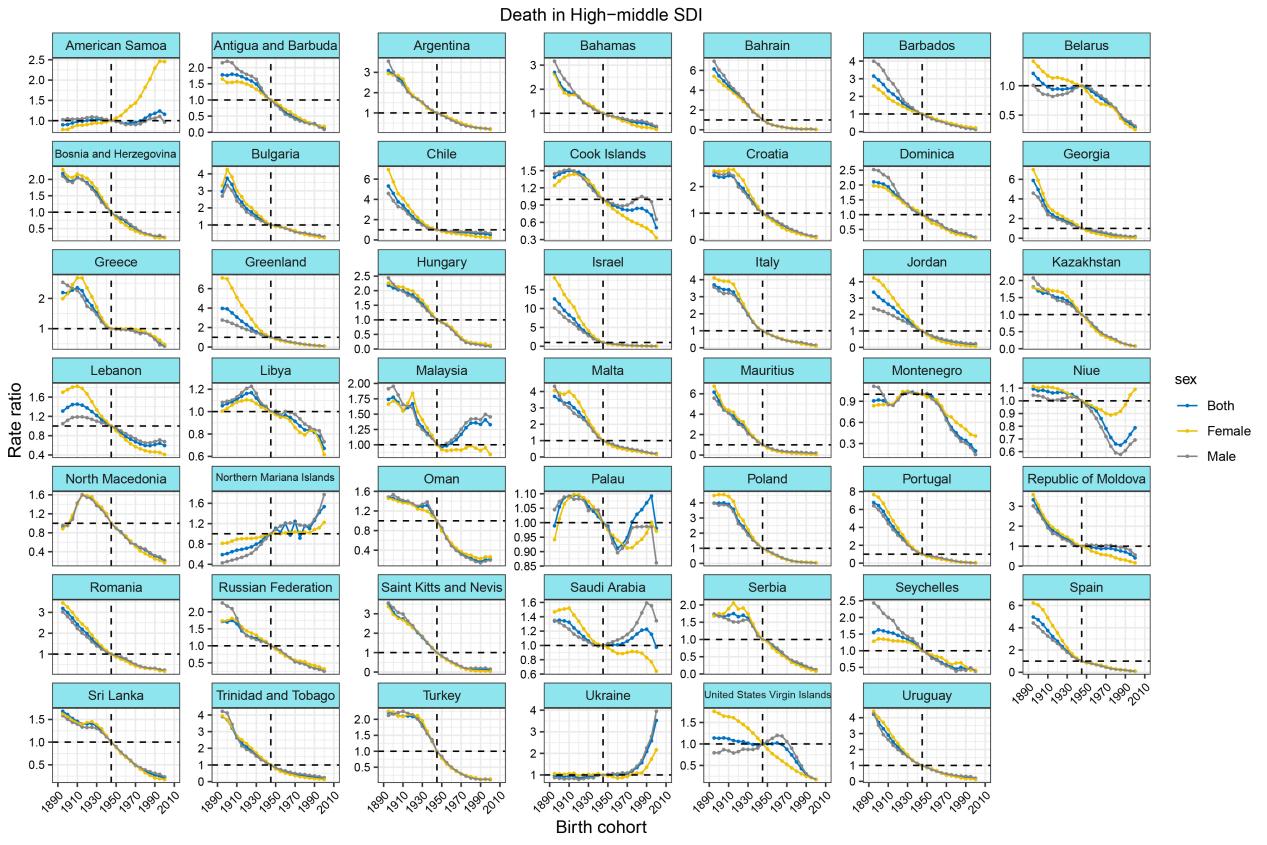


**Figure S8D** Cohort effects on ischemic heart disease mortality in middle SDI countries.


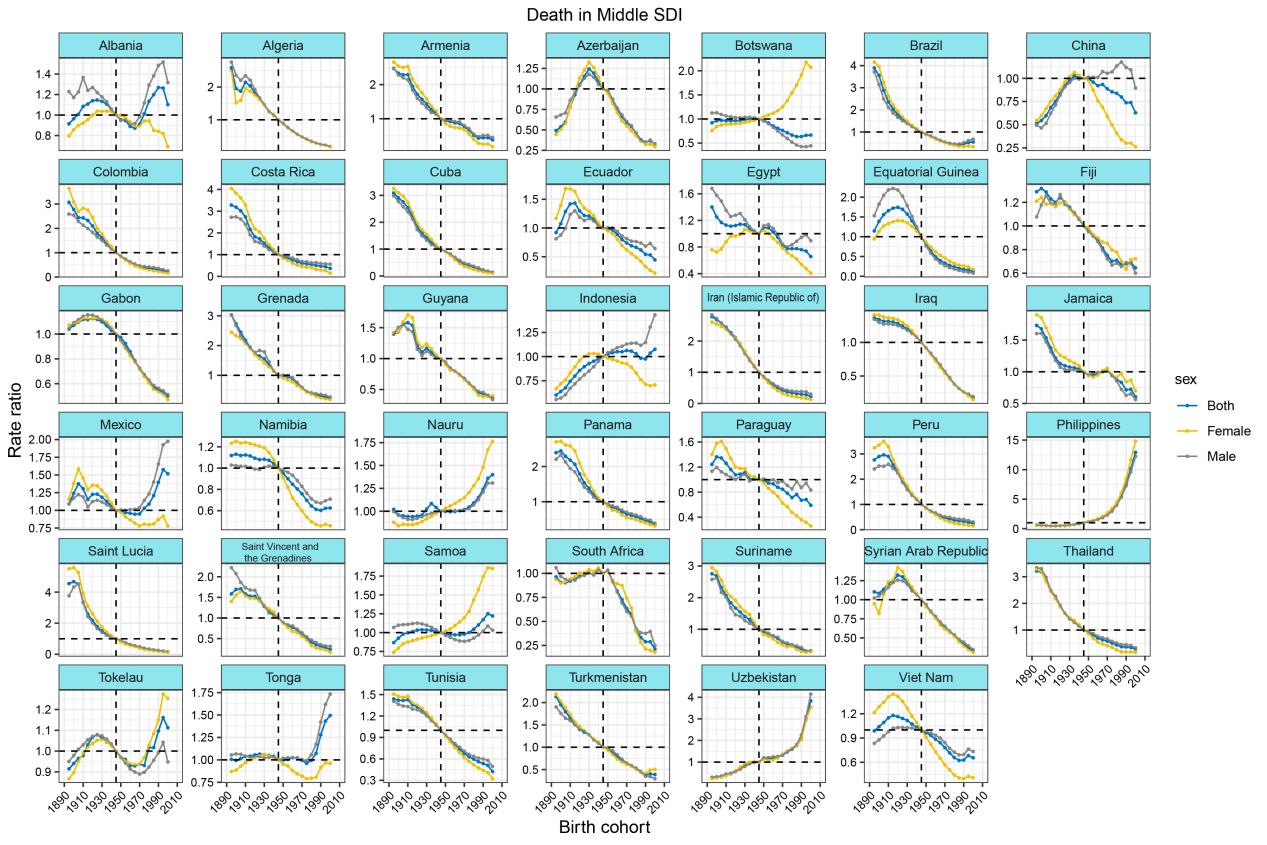


**Figure S8E** Cohort effects on ischemic heart disease mortality in low-middle SDI countries.


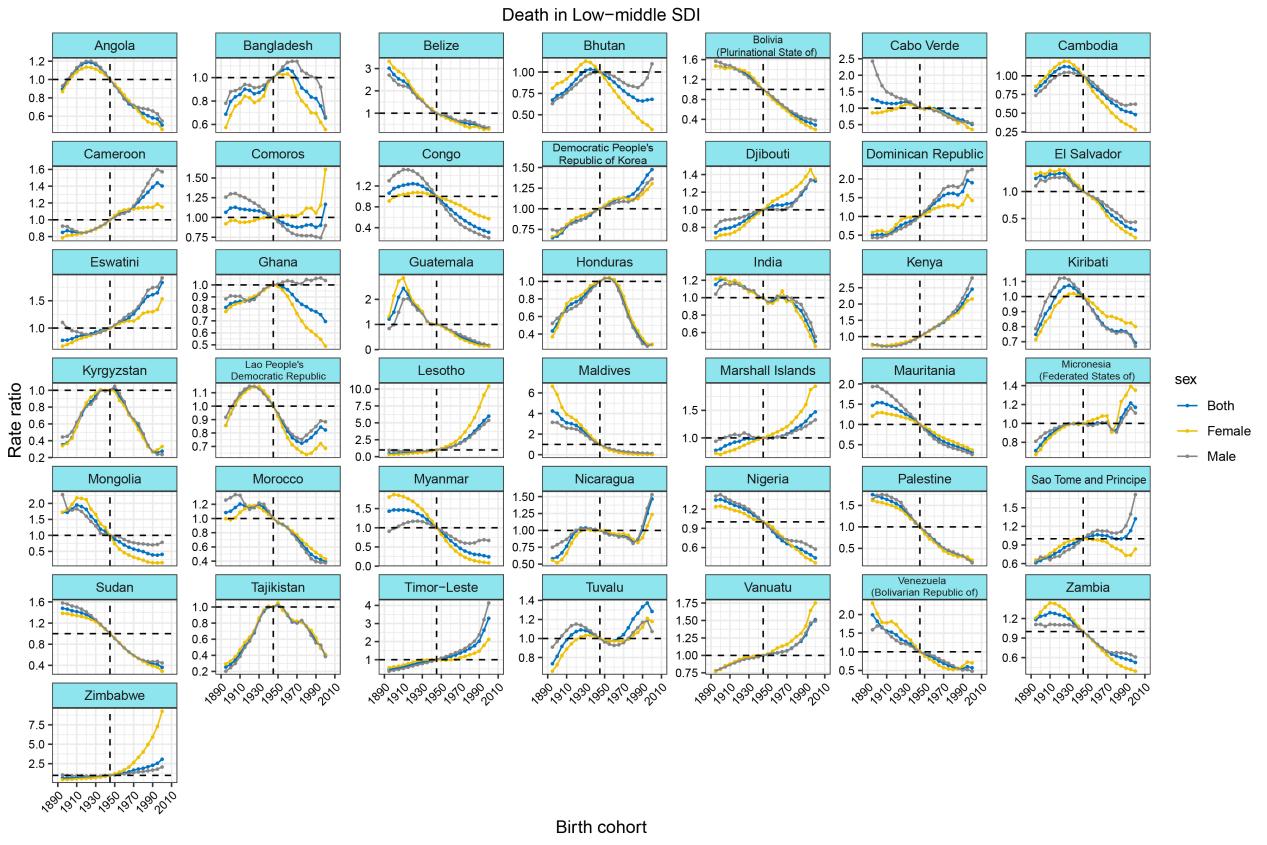


**Figure S8F** Cohort effects on ischemic heart disease mortality in low SDI countries.


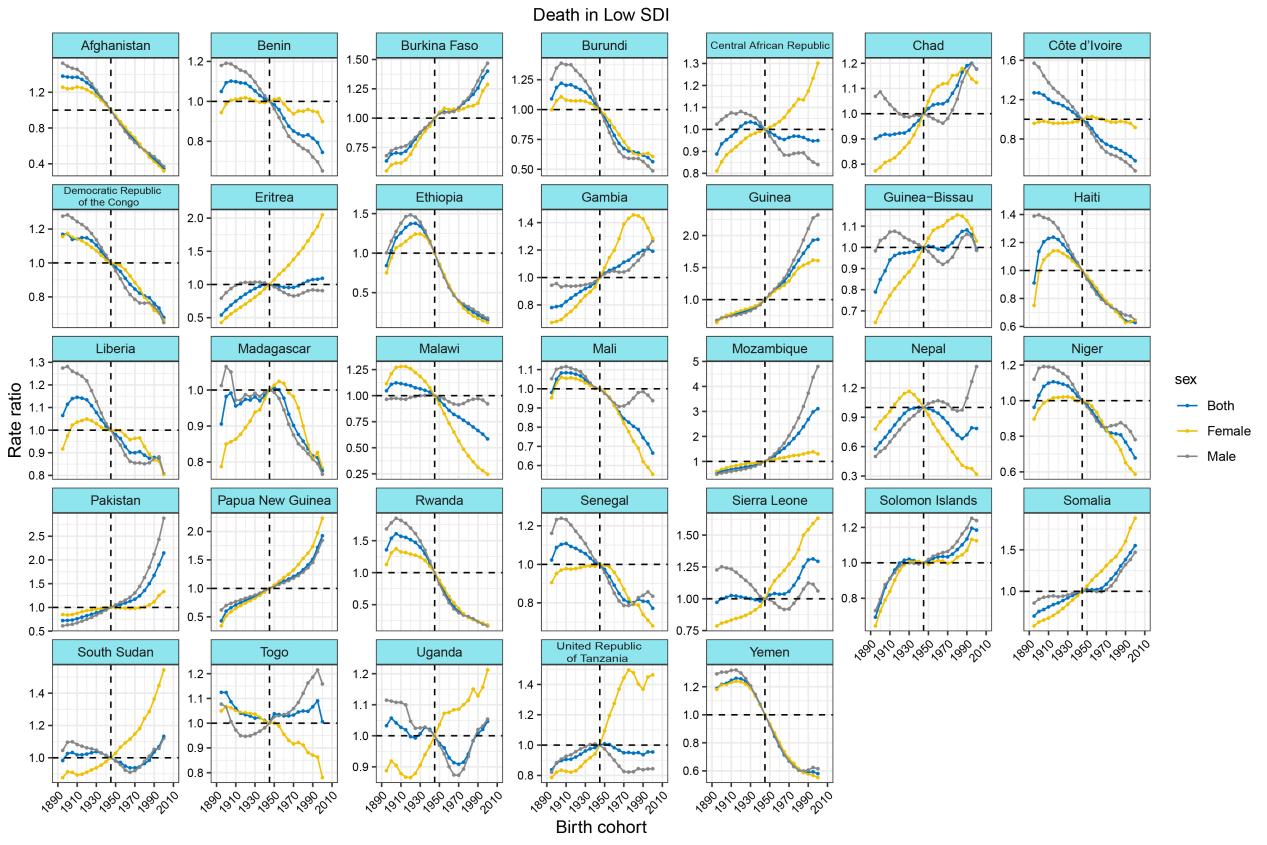


**Figure S9A** Ranked contribution of top 26 risk factors to the age-standardized death rate of ischemic heart disease by 21 GBD regions, 2019, for both sexes combined, females, and males.

Risk factors are ranked with colors (leading risk factor for age-standardized death, dark red; lowest risk factor for age-standardized death, dark blue). LDL, low density lipoprotein; SDI, Socio-demographic Index.


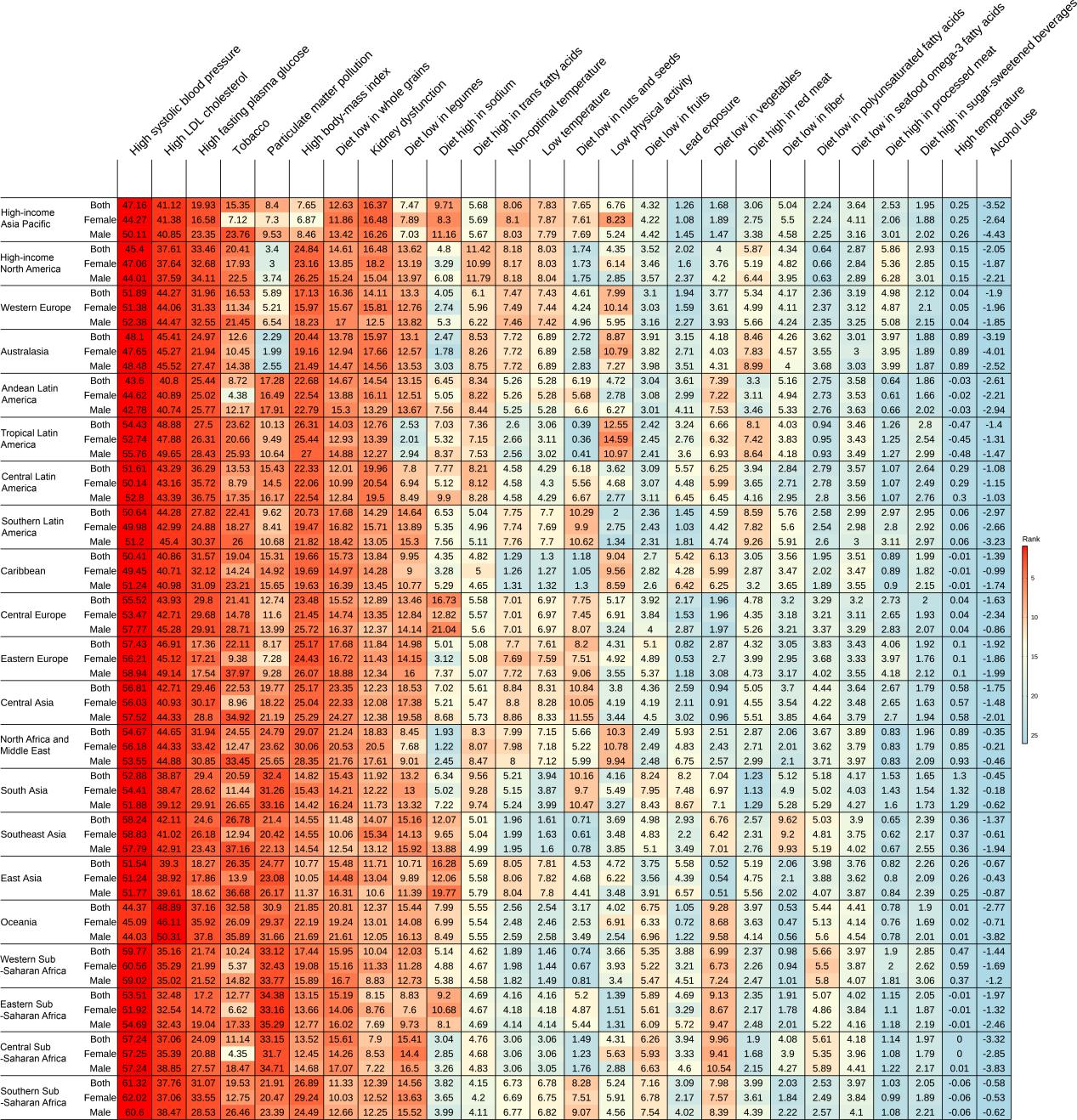


**Figure S9B to S9H** Ranked contribution of top 26 risk factors to the age-standardized death rate of ischemic heart disease across 204 countries and territories, 2019, for both sexes combined, females, and males.

**Figure S9B**

**
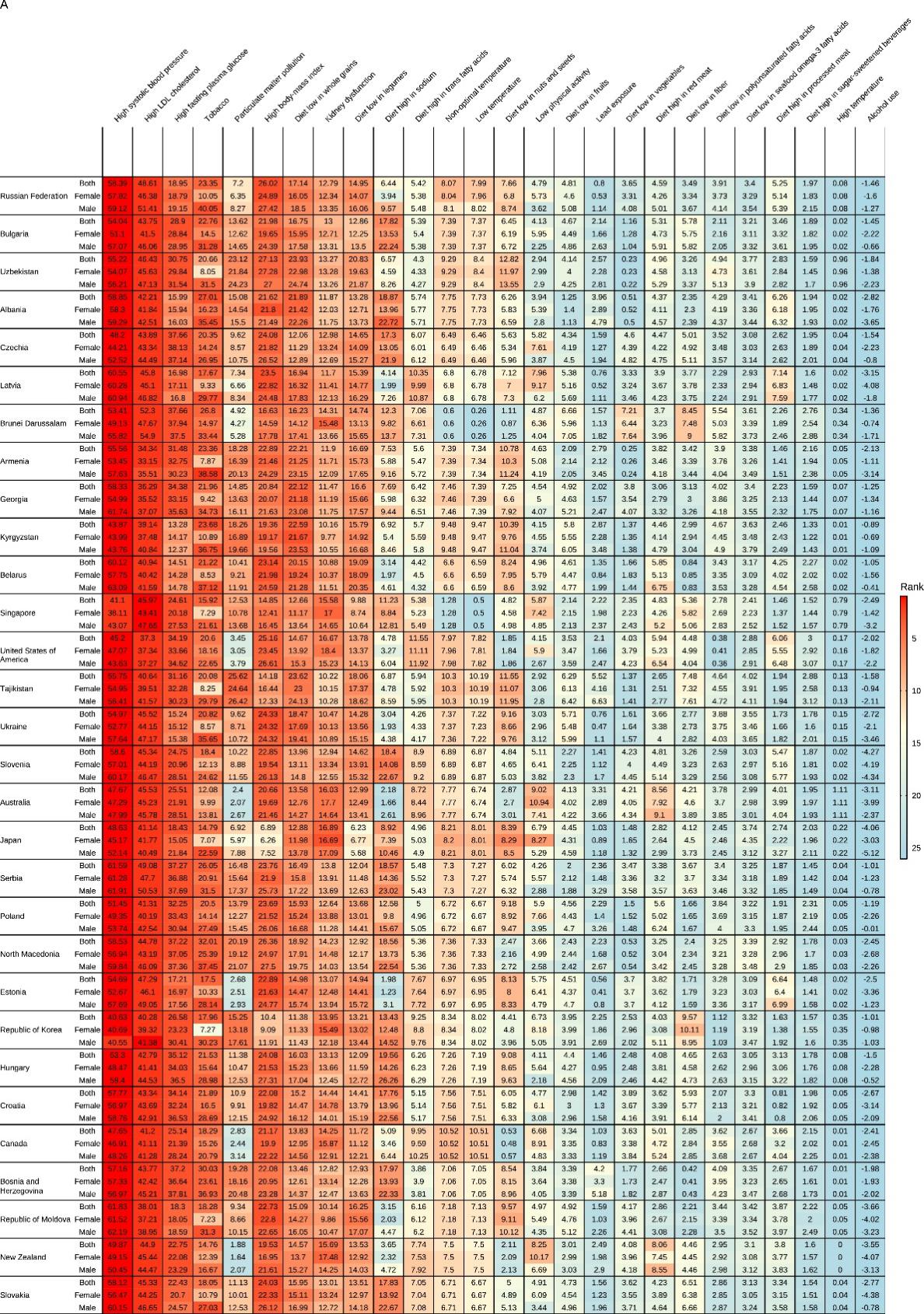
**

**Figure S9C**

**
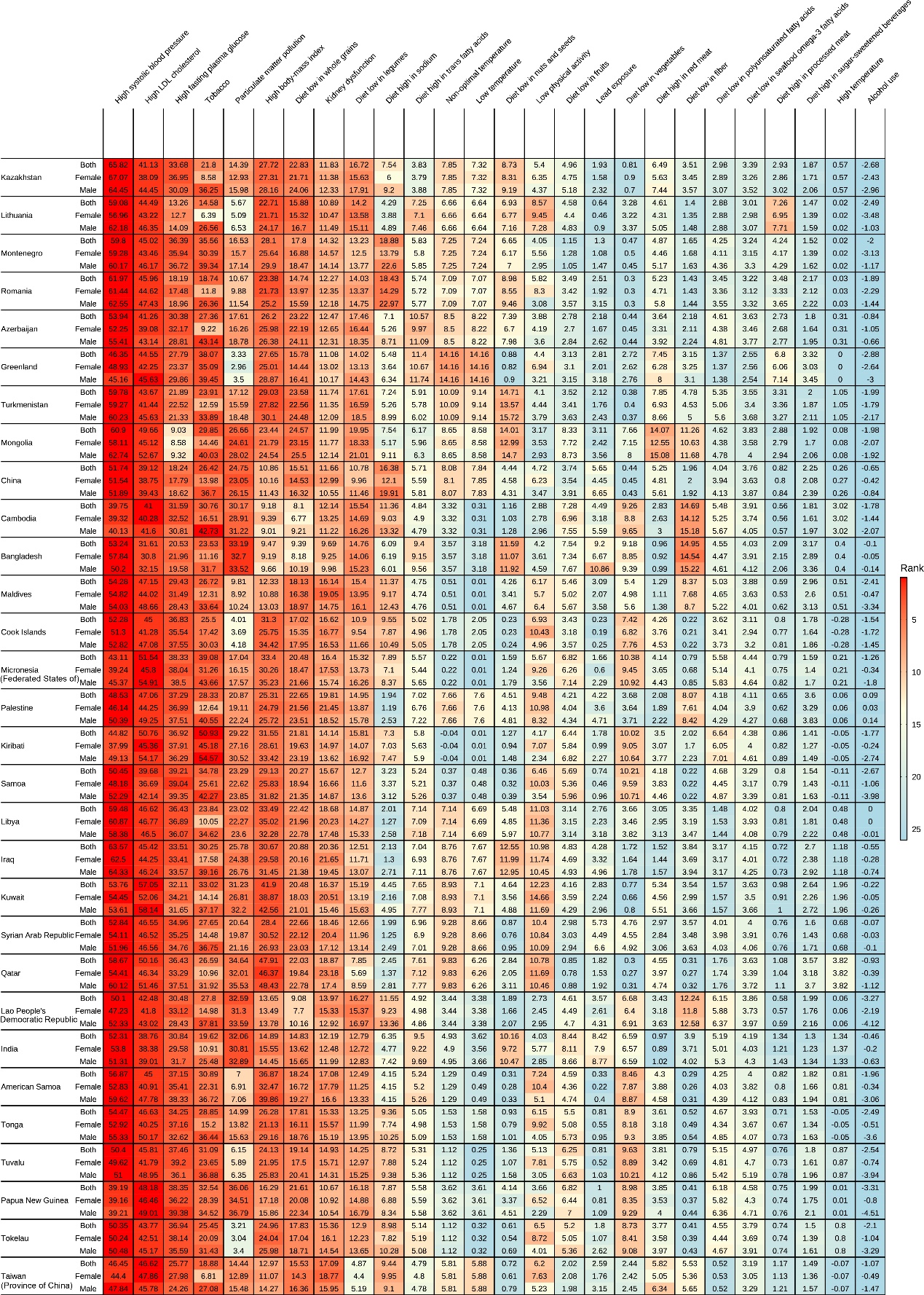
**

**Figure S9D**

**
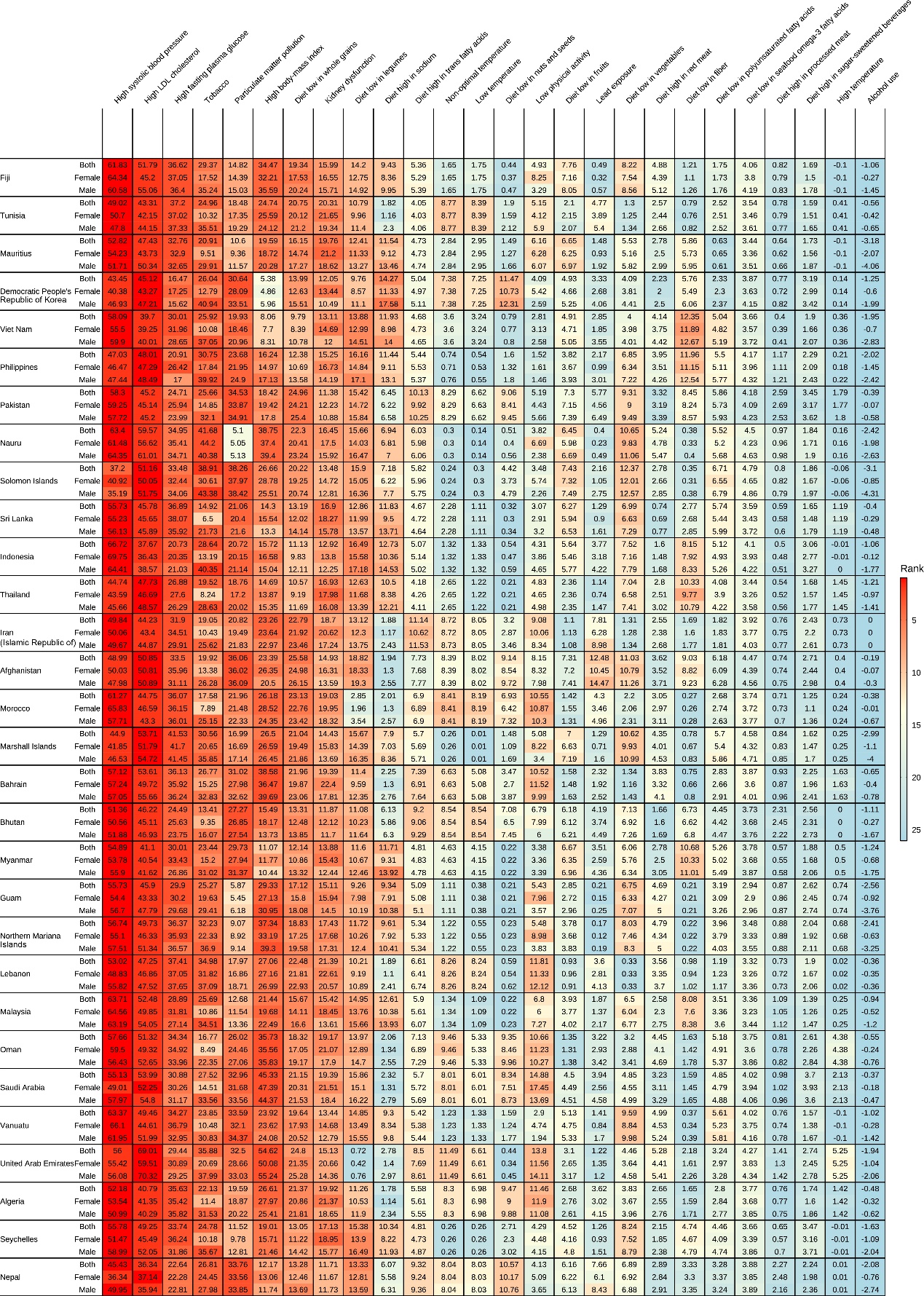
**

**Figure S9E**

**
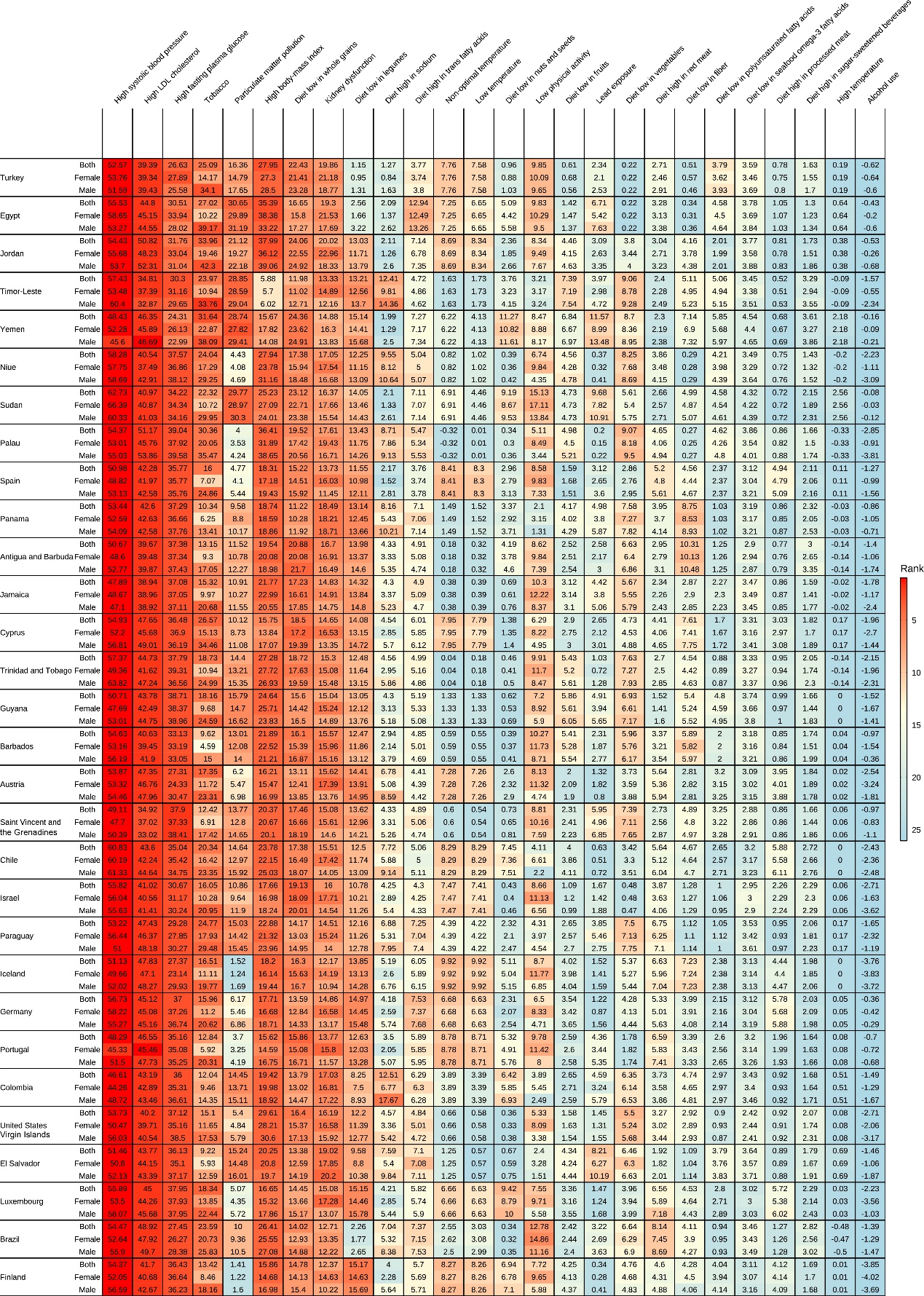
**

**Figure S9F**

**
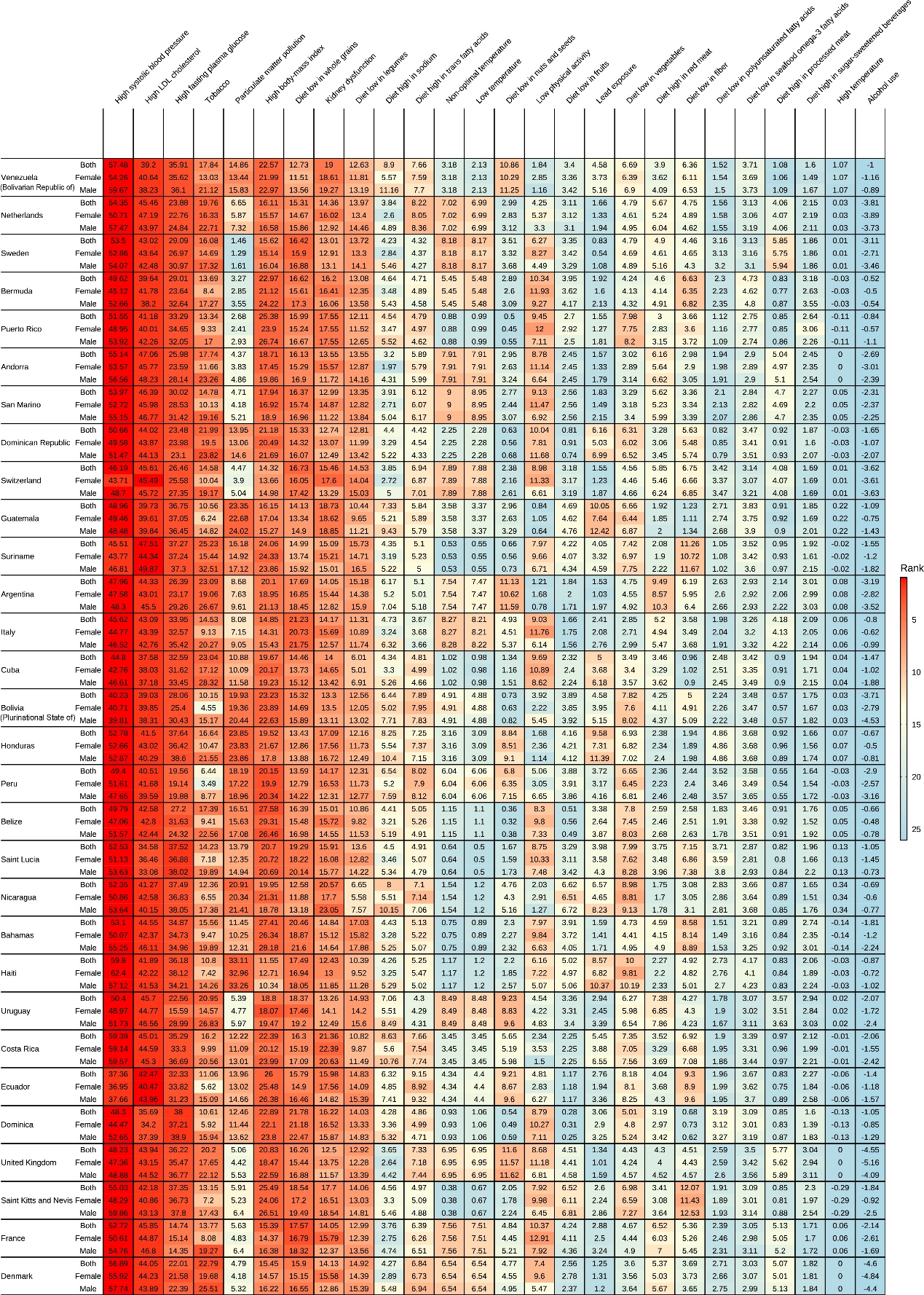
**

**Figure S9G**

**
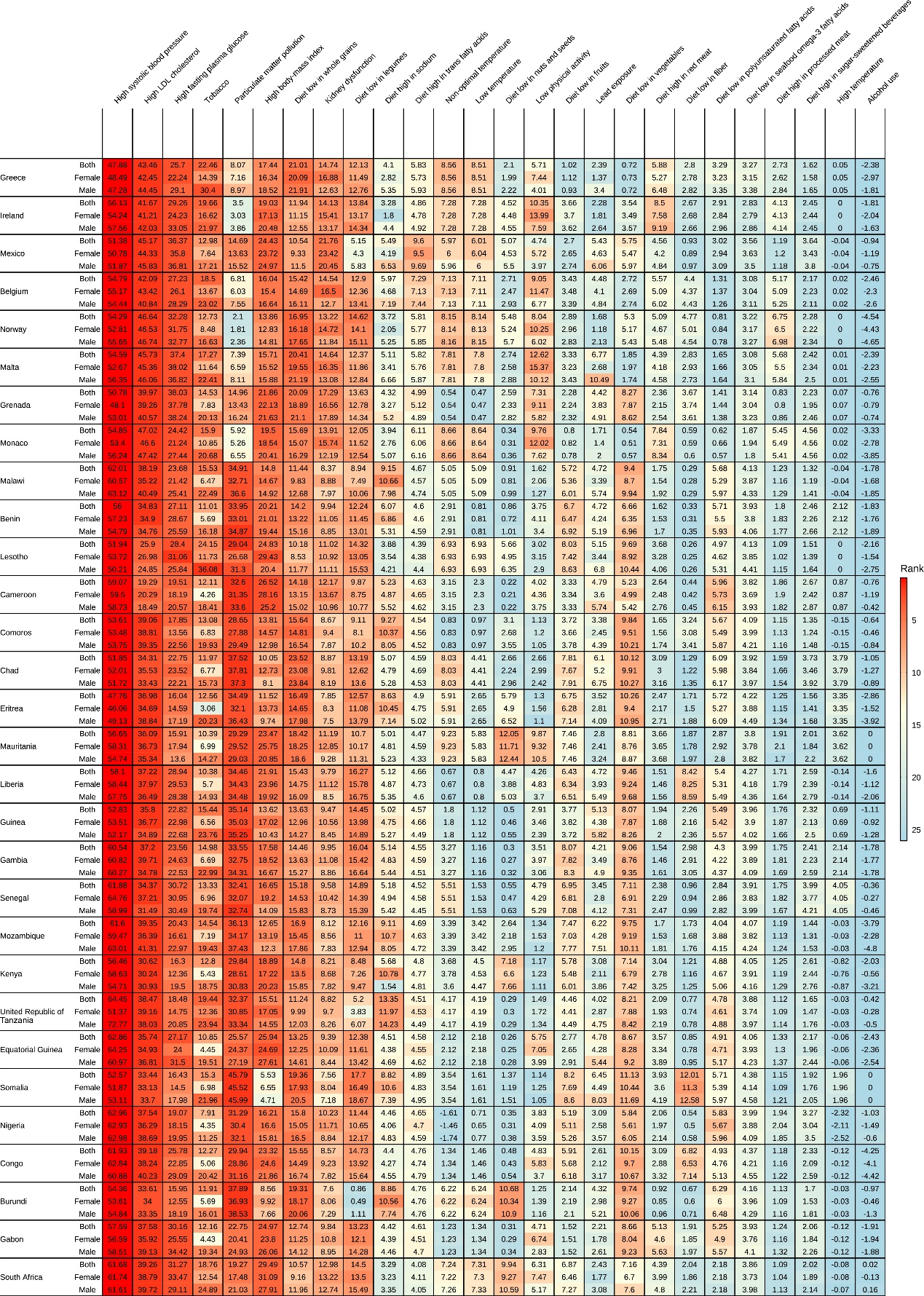
**

**Figure S9H**

**
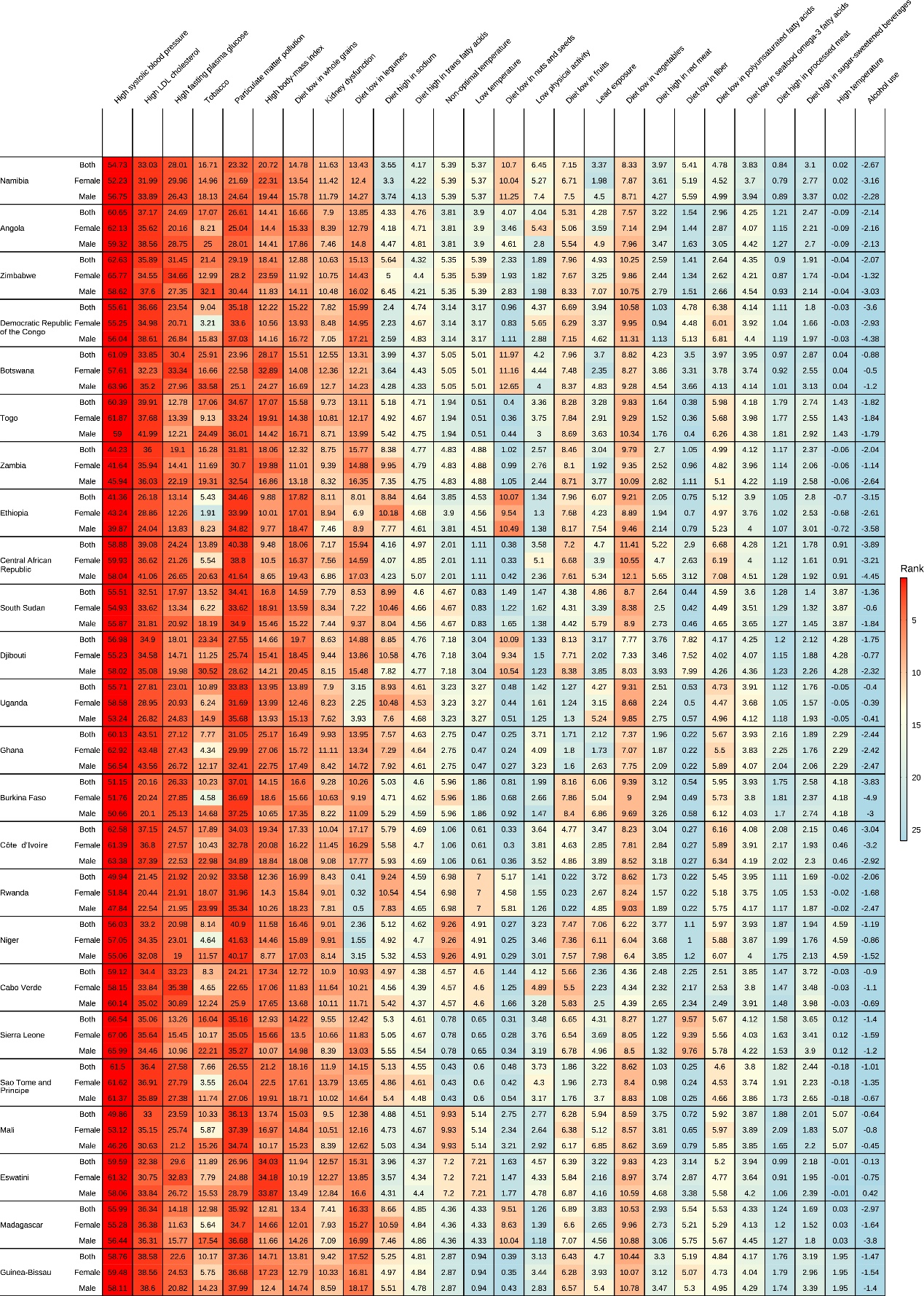
**

**Figure S10A to 10D** Time trends of top 10 risk factors to the age-standardized death rate of ischemic heart disease across 204 countries and territories, 1990 to 2019, for both sexes combined, females, and males.

LDL, low density lipoprotein; SDI, Socio-demographic Index.

**Figure S10A**


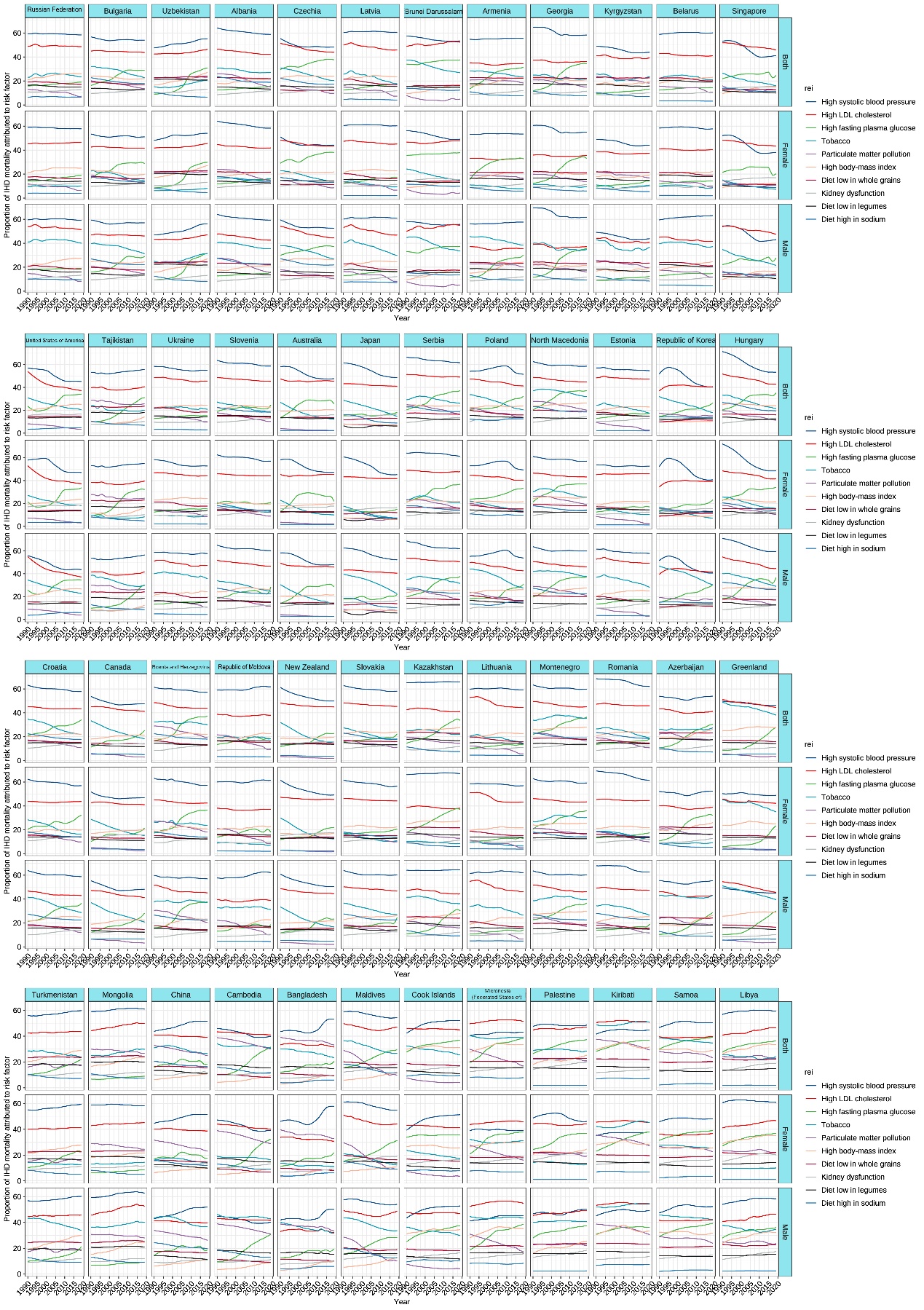


**Figure S10B**

**
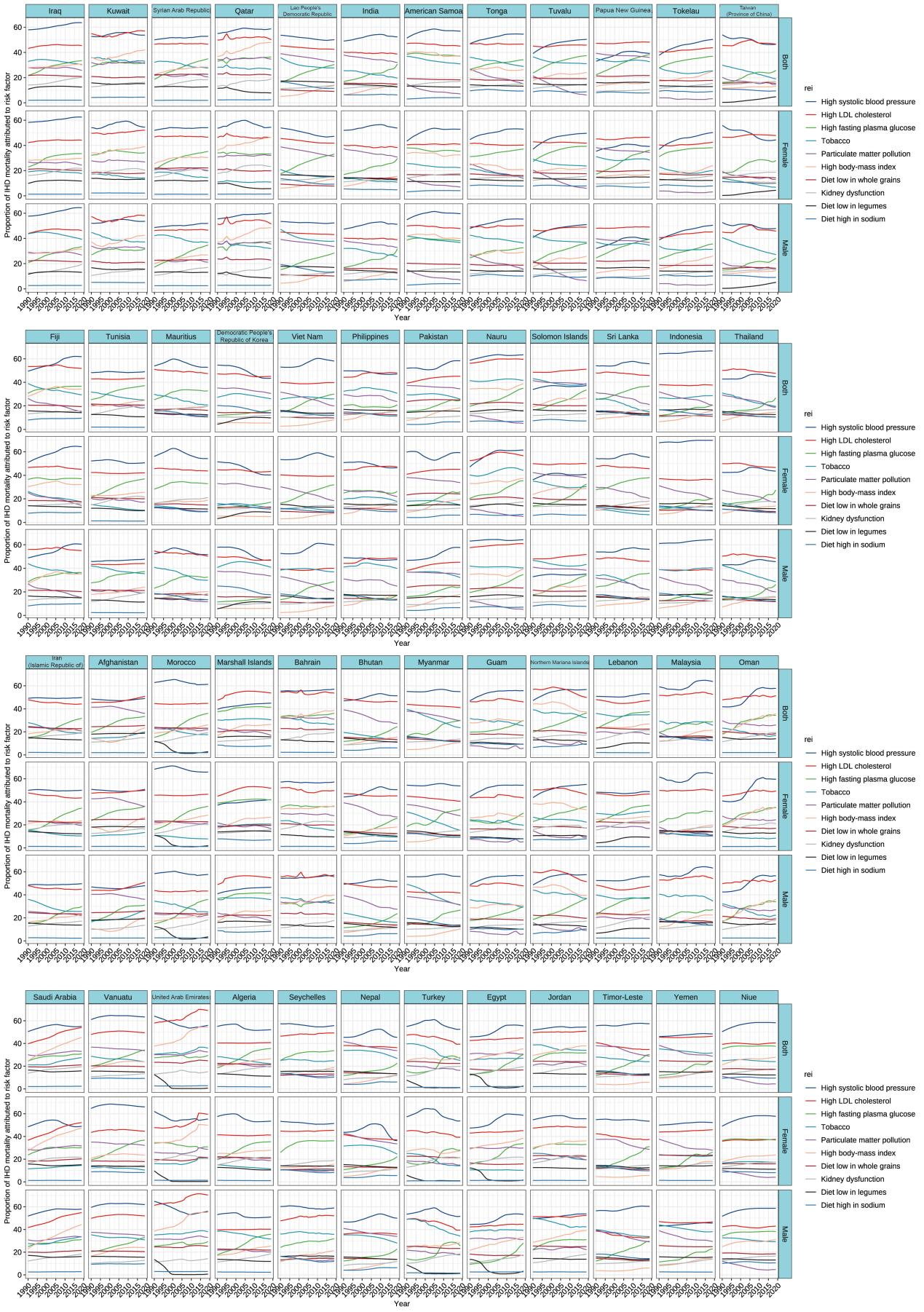
**

**Figure S10C**

**
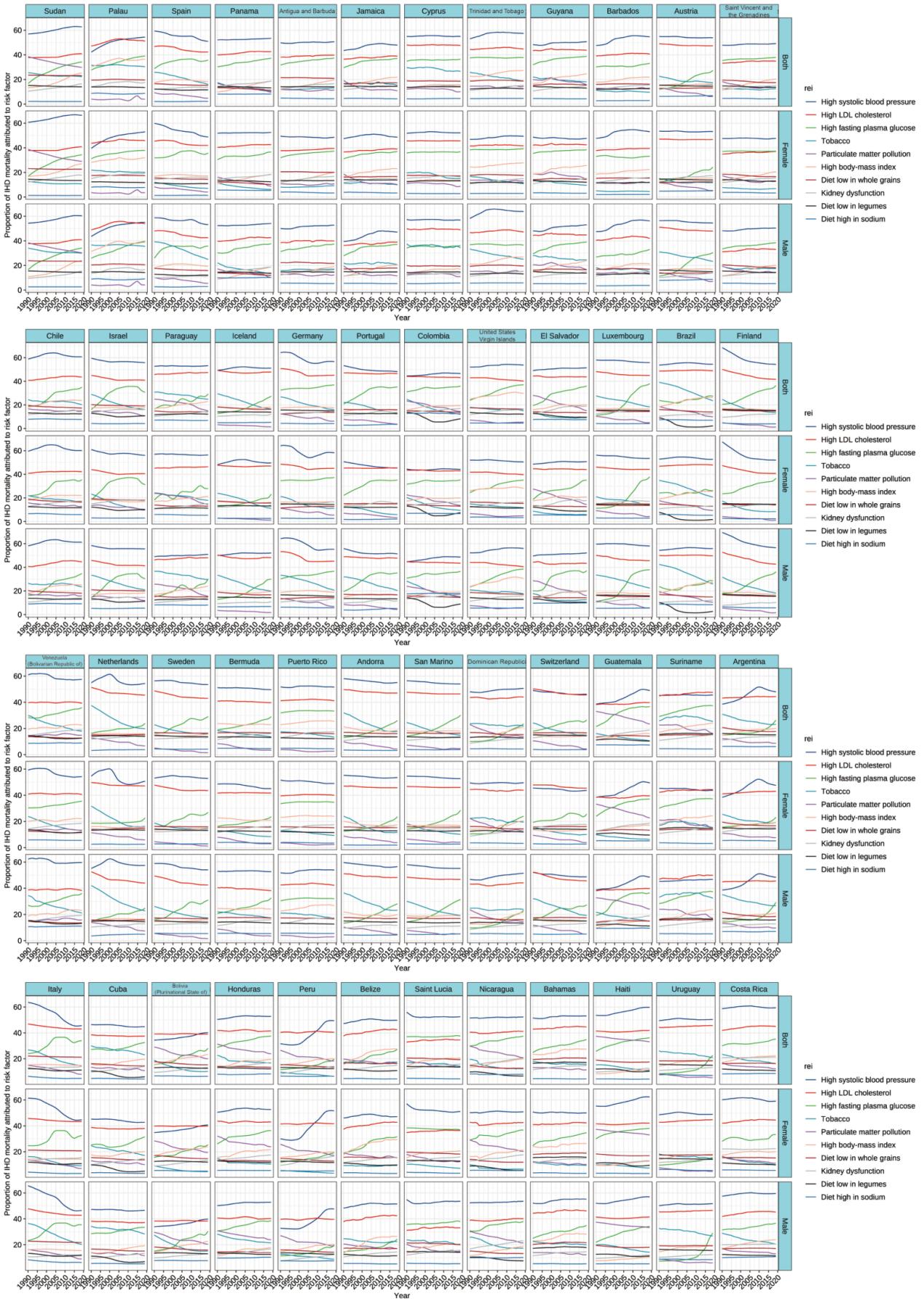
**

**Figure S10D**

**
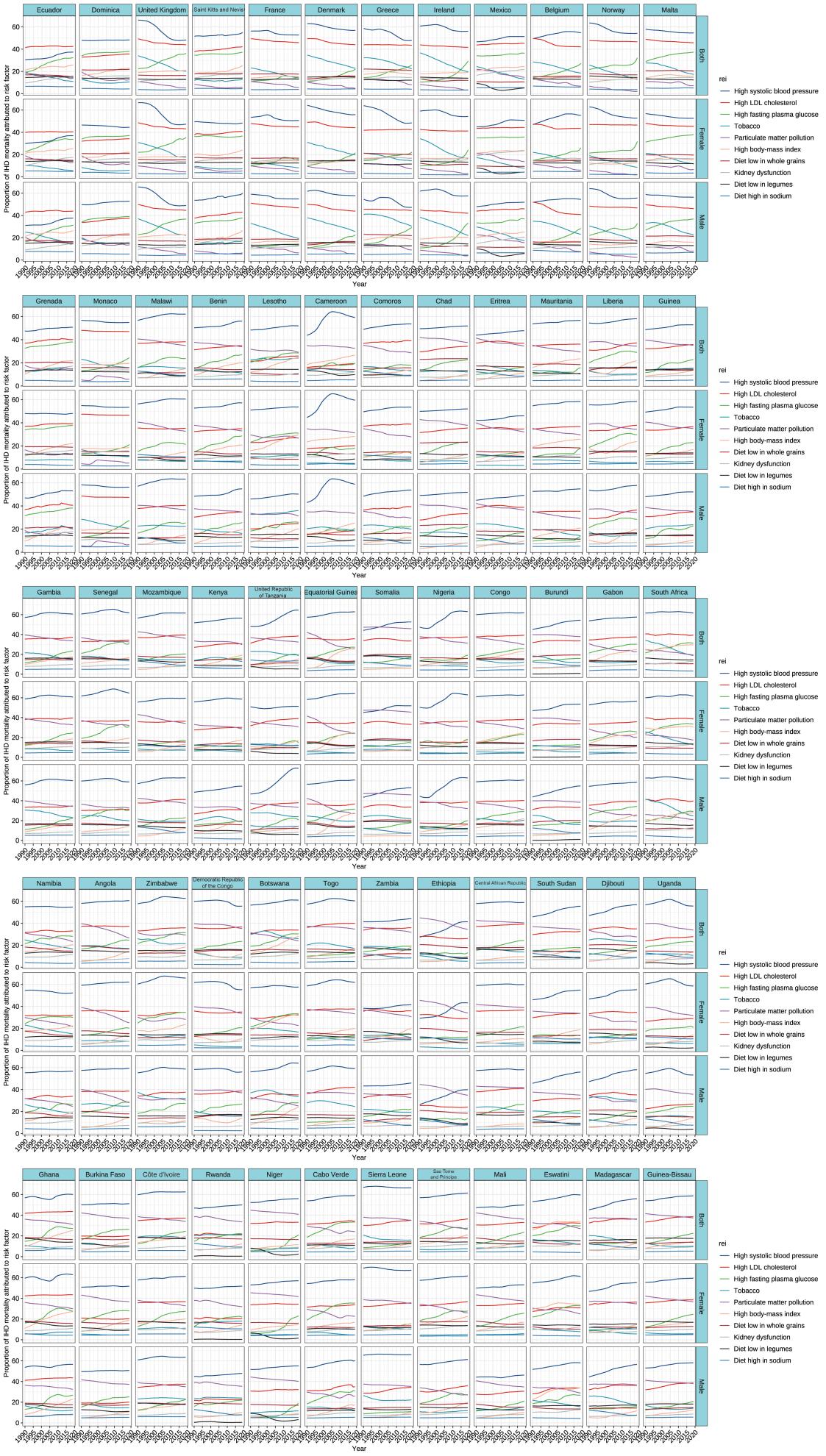
**

**Figure S11A to S11D** Ratio of male to female IHD mortality attributable to top 10 risk factors.

LDL, low density lipoprotein; SDI, Socio-demographic Index.

**Figure S11A**


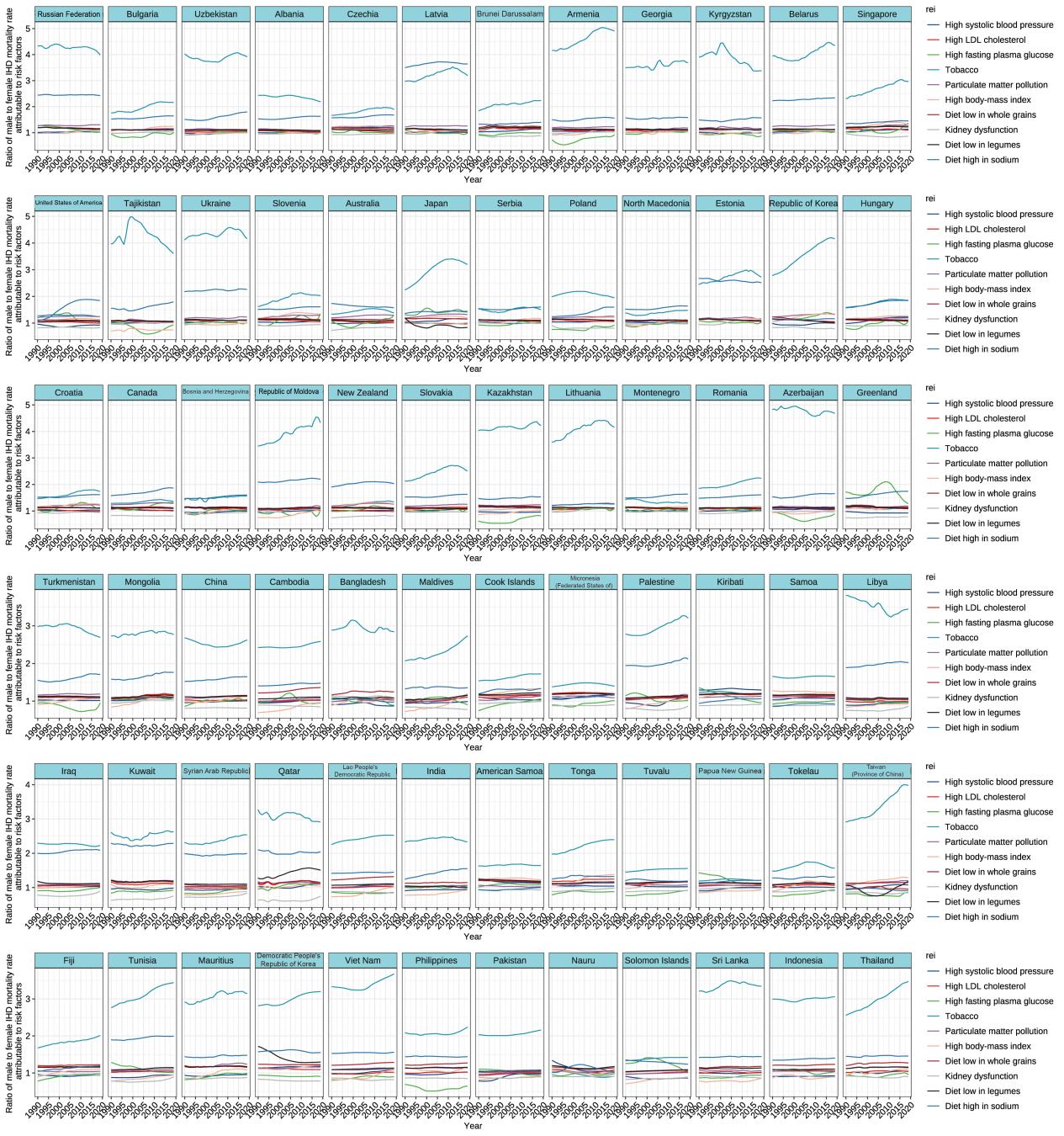


**Figure S11B**


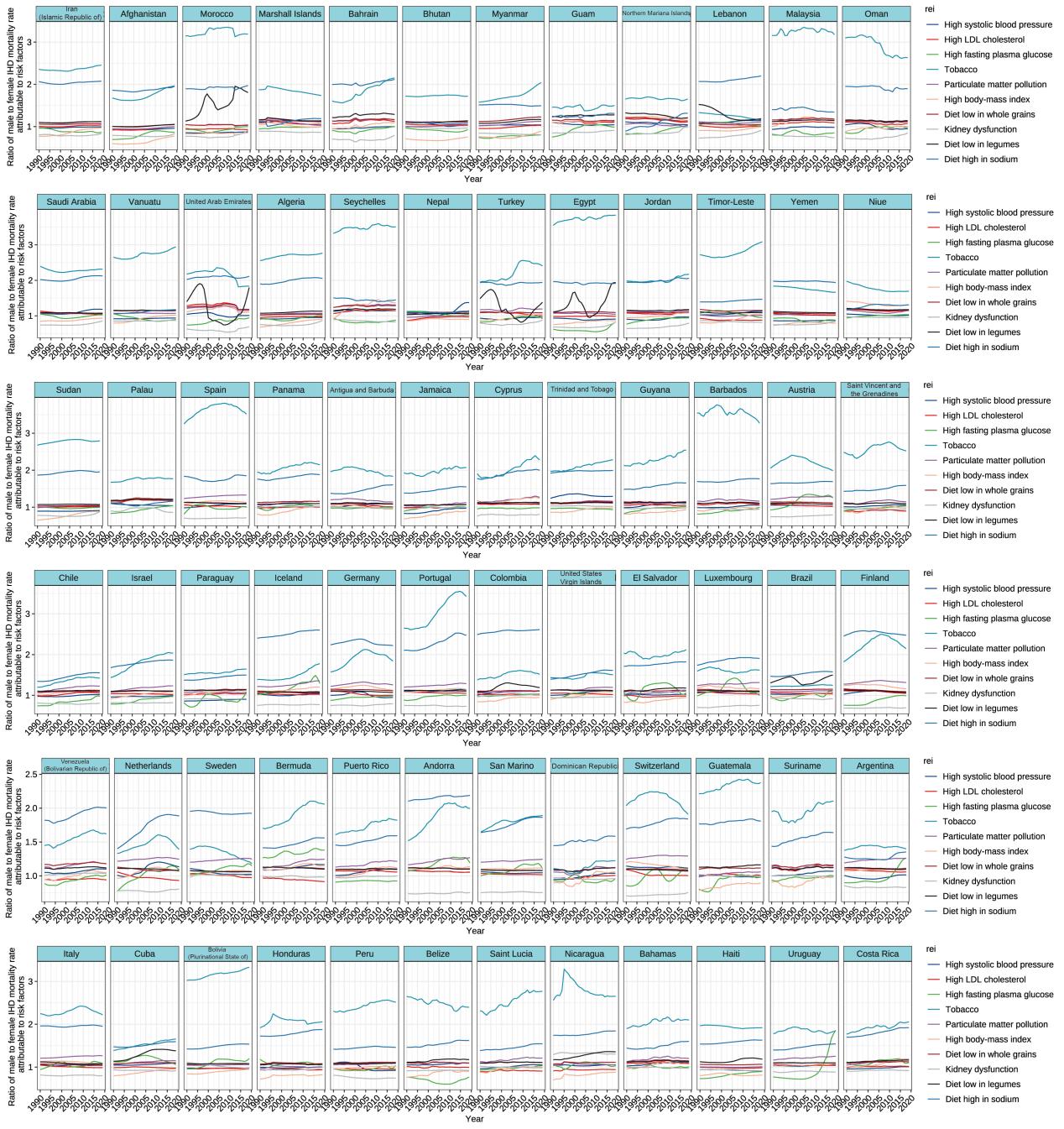


**Figure S11C**


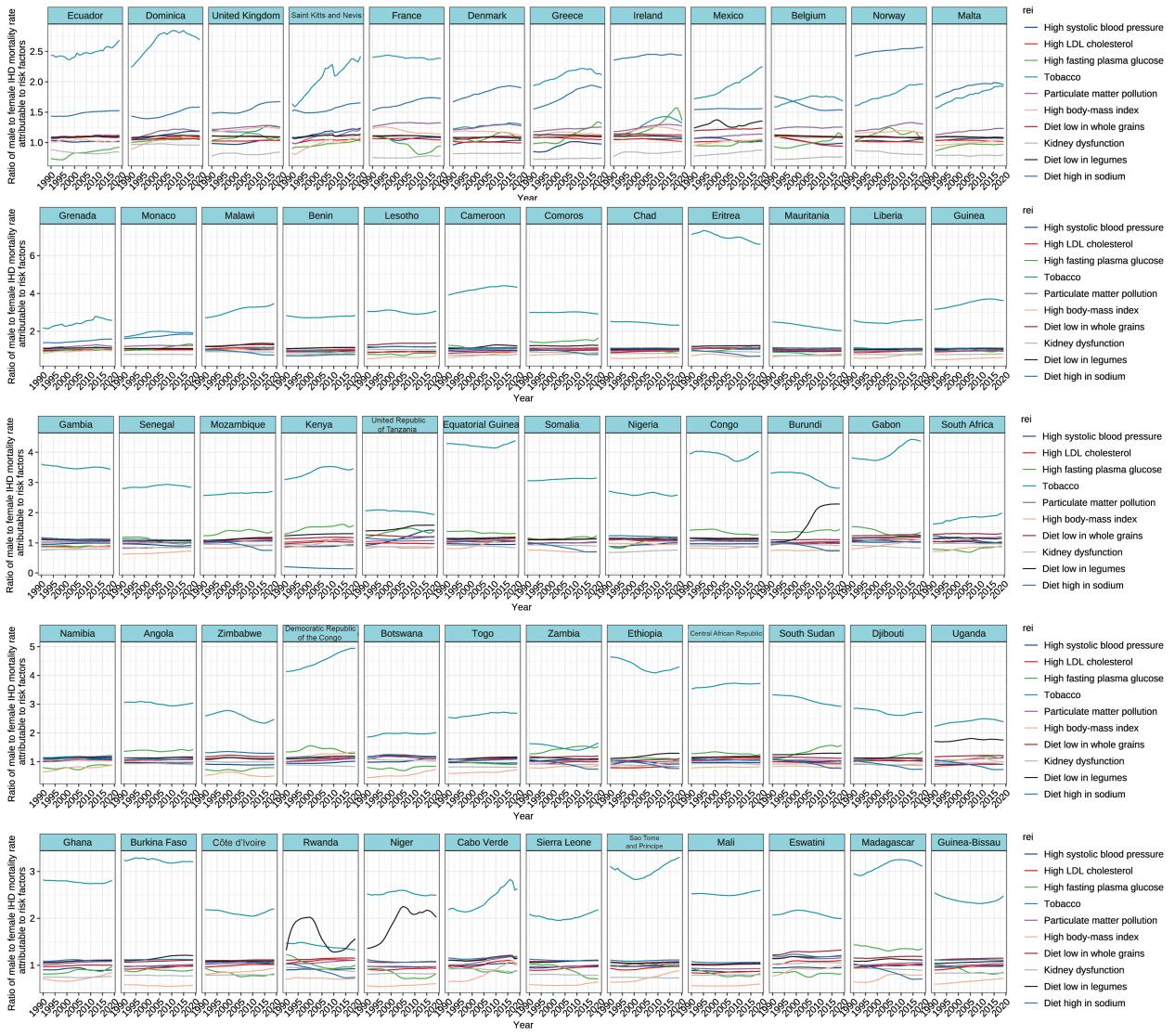


**Figure S12A** The trends of population attributable fractions for 26 factors with age by global and SDI quintiles, 2019, for females.

LDL, low density lipoprotein; SDI, Socio-demographic Index.


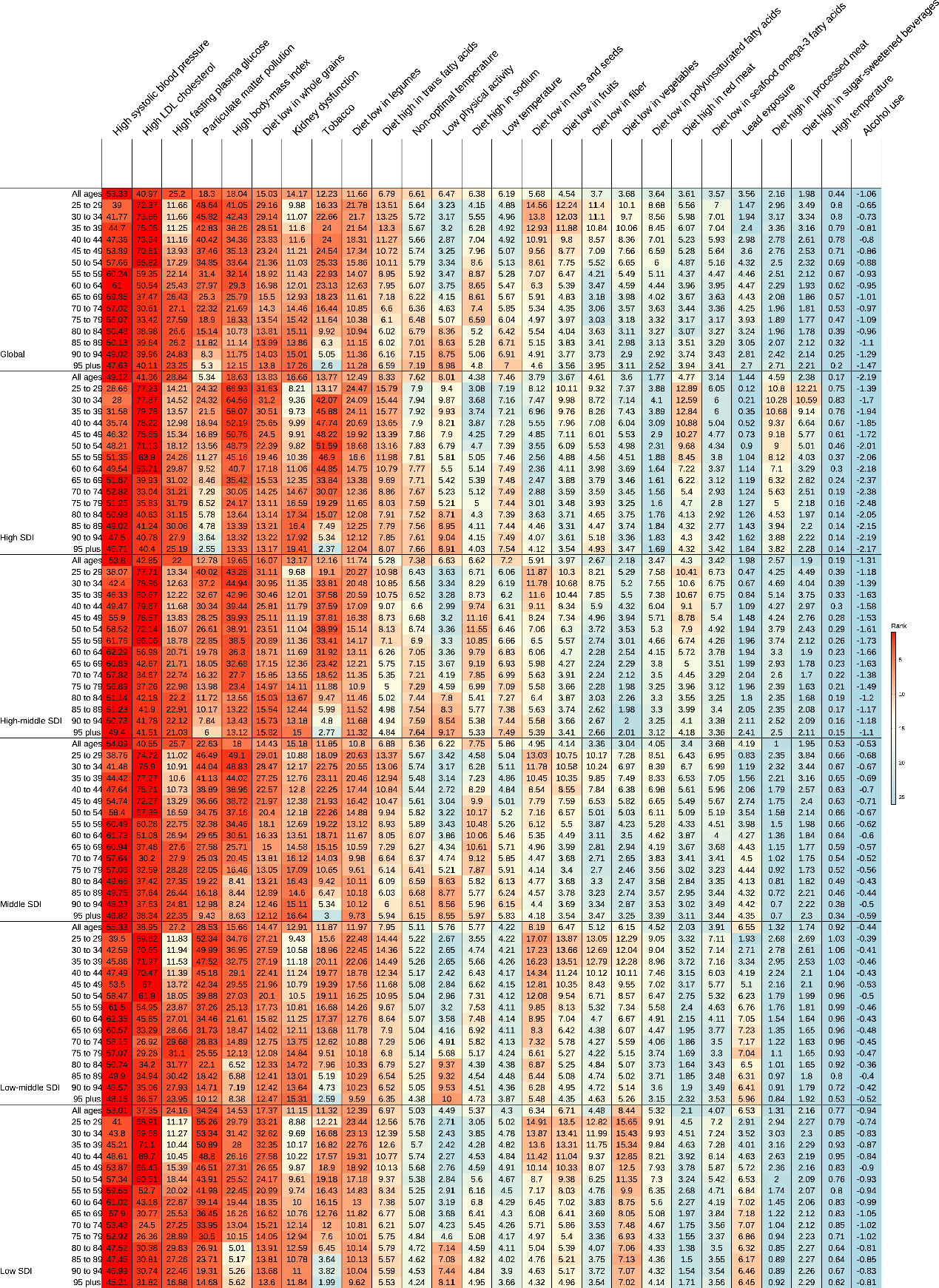


**Figure S12B** The trends of population attributable fractions for 26 factors with age by global and SDI quintiles, 2019, for males.


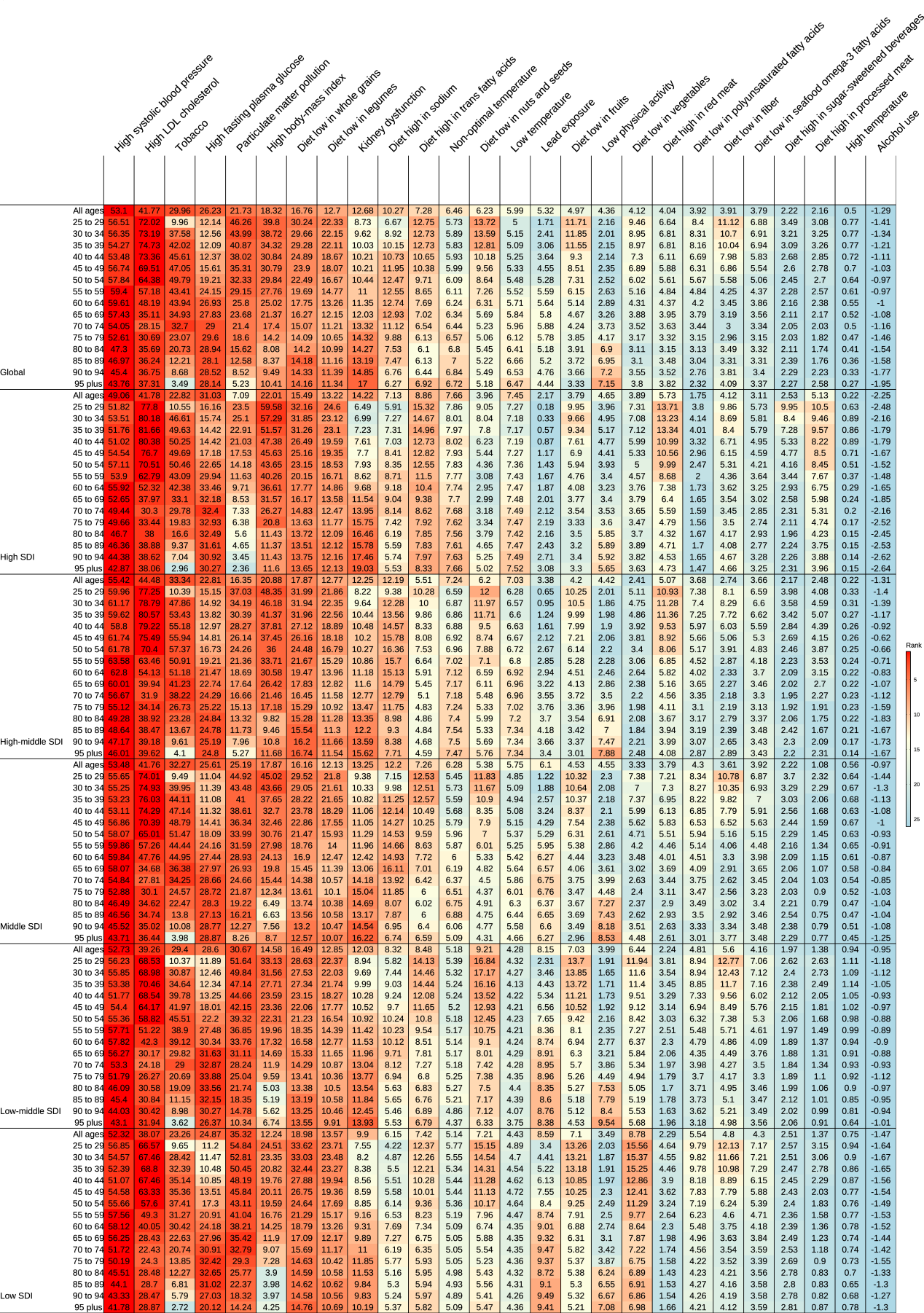

Supplement: Multimedia Appendix 1 [file publichealth_v10i1e46821_app1.docx]
