# Supplementary material for: Assessing Global, Regional, and National Time Trends and Associated Risk Factors of the Mortality in Ischemic Heart Disease Through Global Burden of Disease 2019 Study: Population-Based Study
Source: JMIR Public Health Surveill. 2024 Jan 24;10:e46821. doi: 10.2196/46821 (PMC10851120; doi:10.2196/46821)
Supplement: Multimedia Appendix 2 [file publichealth_v10i1e46821_app2.docx]

**Supplemental materials Table S1 to S3**

**Table S1. Trends in ischemic heart disease mortality across 204 countries and territories from 1990 to 2019.**

| Location | Case in 1990 | Cases in 2019 | Percentage change of cases | AAMR in 1990 | AAMR in 2019 | Percentage change of AAMR | ASMR in 1990 | ASMR in 2019 | Percentage change of ASR | AAPC | Net drift |
| --- | --- | --- | --- | --- | --- | --- | --- | --- | --- | --- | --- |
| Afghanistan | 26110 (21043, 31908) | 34628 (26989, 42665) | 32.62 (0.41, 68.89) | 228.66 (184.29, 279.43) | 90.47 (70.51, 111.46) | -60.44 (-70.05, -49.62) | 410.08 (337.36, 493.65) | 320.87 (253.97, 385.29) | -21.75 (-39.03, -4.26) | -0.81 (-0.86, -0.75) | -1.35 (-1.43, -1.27) |
| Albania | 2829 (2639, 2999) | 6008 (4726, 7562) | 112.37 (66.27, 168.26) | 85.47 (79.72, 90.62) | 220.86 (173.71, 277.99) | 158.39 (102.31, 226.4) | 163.08 (150.45, 173.2) | 145.82 (115.45, 183.28) | -10.58 (-29.92, 12.6) | -0.54 (-1.01, -0.06) | -0.02 (-0.32, 0.27) |
| Algeria | 32059 (26645, 38232) | 58692 (47673, 71563) | 83.07 (43.09, 131.01) | 126.8 (105.39, 151.22) | 140.25 (113.92, 171.01) | 10.61 (-13.55, 39.57) | 395.48 (337.77, 457.93) | 237.25 (197.13, 282.69) | -40.01 (-51.65, -26.49) | -1.73 (-1.83, -1.63) | -2.55 (-2.87, -2.22) |
| American Samoa | 30 (27, 33) | 64 (55, 74) | 118.33 (82.84, 159.71) | 60.98 (54.93, 67.49) | 116.16 (99.87, 133.99) | 90.51 (59.54, 126.61) | 152.48 (136.82, 169.13) | 151.2 (131.24, 173.21) | -0.84 (-15.75, 17.73) | 0.03 (-0.04, 0.1) | 0.11 (-2.17, 2.45) |
| Andorra | 34 (26, 45) | 76 (59, 94) | 125.7 (56.72, 203.18) | 62.05 (48.85, 83.36) | 91.2 (71.26, 113.45) | 46.98 (2.06, 97.43) | 81.21 (65.21, 106.01) | 47.77 (37.09, 59.62) | -41.17 (-57.86, -22.59) | -1.8 (-1.91, -1.7) | -2.3 (-7.04, 2.68) |
| Angola | 3798 (3021, 4677) | 9300 (7170, 11821) | 144.86 (78.51, 239.31) | 36.81 (29.28, 45.33) | 30.86 (23.79, 39.22) | -16.17 (-38.89, 16.17) | 122.67 (96.55, 150.24) | 112.72 (86.2, 140.65) | -8.11 (-32.59, 23.28) | -0.33 (-0.51, -0.16) | -0.87 (-1.05, -0.7) |
| Antigua and Barbuda | 75 (68, 80) | 78 (68, 88) | 4.78 (-8.72, 17.58) | 122.89 (112.72, 131.53) | 88.36 (77.06, 99.38) | -28.1 (-37.36, -19.32) | 133 (122.47, 141.89) | 89.27 (78.21, 99.86) | -32.88 (-41.19, -25.07) | -1.36 (-1.85, -0.87) | -2.5 (-5.29, 0.37) |
| Argentina | 49576 (46648, 51385) | 45371 (41257, 48542) | -8.48 (-13.69, -2.83) | 149.68 (140.84, 155.14) | 100.57 (91.45, 107.59) | -32.81 (-36.64, -28.66) | 170.5 (158.5, 177.48) | 82.07 (74.77, 87.71) | -51.87 (-54.39, -49.08) | -2.52 (-2.76, -2.27) | -2.79 (-3, -2.59) |
| Armenia | 7031 (6661, 7311) | 9255 (7880, 10688) | 31.63 (13.71, 50.56) | 205.92 (195.07, 214.11) | 306.5 (260.96, 353.93) | 48.85 (28.58, 70.25) | 328.88 (307.09, 344.96) | 237.45 (201.67, 272.91) | -27.8 (-37.22, -17.97) | -1.17 (-1.37, -0.96) | -1.83 (-2.14, -1.53) |
| Australia | 33094 (30907, 34279) | 26633 (22749, 28855) | -19.52 (-26.16, -14.79) | 196.28 (183.31, 203.31) | 108.41 (92.6, 117.45) | -44.77 (-49.32, -41.52) | 177.23 (163.8, 184.39) | 56.09 (48.62, 60.44) | -68.35 (-70.41, -66.72) | -3.93 (-4.11, -3.74) | -4.34 (-4.7, -3.99) |
| Austria | 20316 (18896, 22295) | 18087 (15759, 19911) | -10.97 (-18.68, -5.58) | 261.52 (243.24, 286.99) | 202.86 (176.75, 223.32) | -22.43 (-29.15, -17.73) | 166.94 (154.34, 183.57) | 83.31 (73.55, 90.96) | -50.1 (-53.82, -47.4) | -2.49 (-2.63, -2.36) | -3.86 (-4.41, -3.31) |
| Azerbaijan | 14981 (13781, 15973) | 27209 (23829, 31097) | 81.62 (57.56, 110.25) | 204.35 (187.98, 217.88) | 264.71 (231.83, 302.54) | 29.53 (12.38, 49.96) | 333.37 (306.25, 356) | 452.58 (399.78, 506.77) | 35.76 (19.27, 54.74) | 1.34 (0.91, 1.78) | -0.95 (-1.23, -0.68) |
| Bahamas | 193 (178, 208) | 326 (272, 398) | 69.17 (39.55, 104.19) | 75.2 (69.43, 80.93) | 86.56 (72.15, 105.49) | 15.1 (-5.05, 38.93) | 141.08 (129.55, 151.68) | 92.4 (76.87, 111.92) | -34.51 (-45.38, -21.26) | -1.42 (-1.72, -1.11) | -1.59 (-2.64, -0.53) |
| Bahrain | 561 (495, 630) | 863 (698, 1060) | 53.98 (20.93, 99.34) | 110.36 (97.36, 124.09) | 59.84 (48.36, 73.46) | -45.78 (-57.42, -29.8) | 442.46 (394.45, 490.13) | 155.09 (129.29, 186.67) | -64.95 (-71.4, -55.9) | -3.91 (-4.21, -3.61) | -5.14 (-5.66, -4.61) |
| Bangladesh | 47515 (40195, 54201) | 131010 (102869, 160334) | 175.72 (114.66, 253.89) | 43.57 (36.86, 49.7) | 82.26 (64.59, 100.67) | 88.8 (46.99, 142.33) | 114.28 (97.08, 129.93) | 111.23 (86.84, 135.23) | -2.67 (-22.99, 23.13) | -0.23 (-0.77, 0.31) | 0.06 (-0.13, 0.26) |
| Barbados | 382 (353, 403) | 342 (291, 395) | -10.35 (-23.33, 2.37) | 150.41 (138.89, 158.62) | 114.97 (97.81, 132.49) | -23.56 (-34.63, -12.71) | 127.28 (117.37, 134.09) | 69.84 (59.53, 80.56) | -45.13 (-53.08, -37.52) | -2.16 (-2.45, -1.87) | -2.77 (-4.42, -1.09) |
| Belarus | 40174 (37727, 41743) | 54618 (44897, 66663) | 35.95 (12.24, 65.17) | 383.69 (360.32, 398.68) | 574.88 (472.56, 701.66) | 49.83 (23.69, 82.03) | 332.34 (309.73, 346.31) | 334.16 (274.57, 408.41) | 0.55 (-17.19, 22.52) | 0.14 (-0.29, 0.57) | -0.89 (-1.56, -0.21) |
| Belgium | 22290 (20738, 23254) | 15146 (13337, 16362) | -32.05 (-36.67, -27.94) | 223.36 (207.8, 233.02) | 132.63 (116.8, 143.29) | -40.62 (-44.65, -37.02) | 143.69 (133.13, 150.04) | 54.8 (48.83, 58.85) | -61.86 (-63.85, -59.84) | -3.26 (-3.45, -3.07) | -4.11 (-4.45, -3.78) |
| Belize | 125 (114, 134) | 210 (184, 237) | 67.95 (47.06, 91.72) | 67.29 (61.35, 72.1) | 51.23 (44.94, 57.87) | -23.87 (-33.34, -13.1) | 141.08 (128.36, 151.3) | 84.86 (73.99, 95.55) | -39.85 (-46.92, -31.58) | -1.57 (-2.12, -1.02) | -2.18 (-3.43, -0.93) |
| Benin | 2176 (1870, 2531) | 4432 (3575, 5561) | 103.66 (65.2, 157.75) | 44.85 (38.54, 52.15) | 34.99 (28.23, 43.91) | -21.97 (-36.71, -1.25) | 125.48 (107.4, 146.15) | 113.1 (92.68, 137.52) | -9.86 (-24.99, 11.7) | -0.34 (-0.53, -0.16) | -0.41 (-0.68, -0.13) |
| Bermuda | 138 (129, 146) | 110 (91, 130) | -20.66 (-31.79, -7.07) | 232.49 (217.78, 245.14) | 171.27 (142.87, 202.57) | -26.33 (-36.66, -13.71) | 243.75 (227.25, 256.98) | 78.13 (65.46, 92.38) | -67.94 (-72.25, -62.45) | -3.87 (-4.18, -3.56) | -4.47 (-8.08, -0.71) |
| Bhutan | 250 (176, 326) | 663 (517, 811) | 165.57 (94.15, 265.59) | 40.79 (28.7, 53.26) | 87.91 (68.57, 107.48) | 115.54 (57.58, 196.72) | 121.35 (90.57, 156.5) | 134.54 (105.85, 162.82) | 10.87 (-17.64, 48.37) | 0.33 (0.3, 0.37) | -0.26 (-0.91, 0.38) |
| Bolivia (Plurinational State of) | 3658 (2697, 4821) | 7714 (5470, 10112) | 110.86 (63.52, 171.9) | 56.98 (42, 75.07) | 64.22 (45.54, 84.19) | 12.72 (-12.59, 45.35) | 140.42 (104.32, 182.06) | 105.91 (76.21, 136.33) | -24.58 (-40.65, -3.46) | -0.95 (-1.04, -0.87) | -1.66 (-1.88, -1.45) |
| Bosnia and Herzegovina | 7298 (6859, 7648) | 8817 (7237, 10660) | 20.82 (-0.04, 45.5) | 160.82 (151.15, 168.53) | 267.2 (219.3, 323.02) | 66.14 (37.46, 100.08) | 235.58 (217.74, 248.52) | 162.94 (133.68, 195.74) | -30.83 (-42.3, -17.43) | -1.32 (-2.38, -0.26) | -2.49 (-2.82, -2.16) |
| Botswana | 559 (440, 708) | 1409 (1039, 1875) | 152.18 (83.24, 242.85) | 42.92 (33.84, 54.4) | 60.24 (44.42, 80.18) | 40.36 (1.99, 90.82) | 122.56 (98.52, 153.4) | 130.96 (98.83, 170.58) | 6.86 (-19.13, 42.84) | 0.22 (0.04, 0.41) | -0.51 (-0.99, -0.02) |
| Brazil | 117247 (111650, 121247) | 171246 (156180, 180511) | 46.06 (37.65, 53.13) | 78.78 (75.02, 81.46) | 79.04 (72.08, 83.31) | 0.33 (-5.44, 5.19) | 157.85 (146.86, 163.95) | 74.9 (67.93, 79.12) | -52.55 (-54.9, -50.31) | -2.51 (-2.67, -2.36) | -2.22 (-2.3, -2.13) |
| Brunei Darussalam | 149 (134, 165) | 262 (234, 294) | 75.54 (50.88, 105.33) | 57.69 (51.65, 64.01) | 59.9 (53.48, 67.35) | 3.83 (-10.76, 21.45) | 204.02 (185.1, 224.66) | 132.31 (117.58, 147.23) | -35.15 (-43.42, -25.22) | -1.56 (-1.95, -1.17) | -1.79 (-2.66, -0.91) |
| Bulgaria | 35794 (33425, 37383) | 34450 (28595, 40549) | -3.75 (-19.79, 14.65) | 412.32 (385.03, 430.63) | 496.79 (412.35, 584.73) | 20.49 (0.41, 43.52) | 380 (351.23, 396.5) | 239.07 (197.94, 281.15) | -37.09 (-47.4, -25.13) | -1.62 (-2.04, -1.19) | -2.31 (-2.69, -1.93) |
| Burkina Faso | 3733 (3049, 4462) | 9474 (7865, 11196) | 153.79 (105.73, 214.03) | 39.06 (31.91, 46.68) | 41.75 (34.66, 49.34) | 6.9 (-13.35, 32.27) | 108.83 (90.43, 127.51) | 130.19 (110.76, 152.07) | 19.63 (-0.93, 46.13) | 0.72 (0.5, 0.94) | 0.7 (0.5, 0.9) |
| Burundi | 2642 (2047, 3433) | 4029 (3026, 5300) | 52.5 (9.11, 114.02) | 47.42 (36.74, 61.62) | 33.76 (25.35, 44.41) | -28.81 (-49.07, -0.1) | 132.83 (103.43, 170.47) | 113.58 (86.32, 147.64) | -14.5 (-37.79, 16.34) | -0.57 (-0.73, -0.42) | -0.93 (-1.16, -0.7) |
| Cabo Verde | 306 (268, 336) | 569 (484, 635) | 85.82 (60.56, 113.51) | 87.07 (76.33, 95.67) | 100.94 (85.93, 112.73) | 15.93 (0.17, 33.21) | 127.93 (112.31, 140.51) | 138.83 (118.43, 155.21) | 8.52 (-6.03, 24.31) | 0.33 (-0.06, 0.73) | -0.69 (-1.74, 0.37) |
| Cambodia | 4628 (3820, 5765) | 11456 (9346, 13757) | 147.54 (87.78, 214.1) | 44.6 (36.82, 55.56) | 69 (56.29, 82.86) | 54.69 (17.35, 96.28) | 122.76 (102.06, 151.51) | 118.57 (96.93, 139.99) | -3.41 (-26.25, 19.68) | -0.11 (-0.15, -0.07) | -0.81 (-0.97, -0.64) |
| Cameroon | 3547 (2824, 4355) | 10619 (8306, 13691) | 199.34 (121.48, 310.18) | 34.14 (27.18, 41.91) | 36.49 (28.54, 47.04) | 6.88 (-20.92, 46.46) | 106.31 (85.48, 129.74) | 115.52 (93.18, 145.7) | 8.66 (-17.62, 44.19) | 0.36 (0.23, 0.48) | 0.6 (0.41, 0.78) |
| Canada | 51323 (47469, 53322) | 48774 (42889, 52709) | -4.97 (-10.61, 0.84) | 188.29 (174.16, 195.63) | 133.55 (117.44, 144.33) | -29.07 (-33.29, -24.74) | 162.47 (149.15, 169.25) | 63.95 (56.89, 68.7) | -60.64 (-62.43, -58.54) | -3.19 (-3.32, -3.06) | -3.47 (-3.74, -3.2) |
| Central African Republic | 1456 (1173, 1804) | 2525 (1888, 3318) | 73.38 (31.01, 129.64) | 53.07 (42.75, 65.74) | 47.64 (35.63, 62.61) | -10.23 (-32.17, 18.9) | 151.67 (119.71, 188.99) | 146.61 (109.7, 194.31) | -3.33 (-25.26, 25.09) | -0.06 (-0.18, 0.06) | -0.05 (-0.36, 0.27) |
| Chad | 2752 (2274, 3322) | 5368 (4389, 6613) | 95.07 (56.82, 144.52) | 45.69 (37.77, 55.16) | 32.73 (26.76, 40.33) | -28.37 (-42.41, -10.21) | 114.69 (95.01, 138.33) | 120.14 (99.28, 145.38) | 4.75 (-13.72, 29.48) | 0.19 (0.13, 0.25) | 0.3 (0.04, 0.56) |
| Chile | 11006 (10440, 11388) | 11986 (10802, 12844) | 8.91 (1.87, 15.53) | 82.87 (78.62, 85.75) | 65.87 (59.35, 70.58) | -20.52 (-25.66, -15.69) | 134.43 (125.37, 139.69) | 50.38 (45.32, 54.01) | -62.52 (-64.67, -60.34) | -3.38 (-3.57, -3.18) | -2.12 (-2.52, -1.73) |
| China | 607756 (536349, 678753) | 1874007 (1612111, 2131822) | 208.35 (159.08, 267.32) | 51.34 (45.31, 57.34) | 131.75 (113.34, 149.88) | 156.61 (115.61, 205.68) | 98.98 (88.28, 110.05) | 116.41 (100.29, 131.52) | 17.61 (0.22, 38.94) | 0.58 (0.22, 0.94) | 0.13 (-0.03, 0.3) |
| Colombia | 22551 (21178, 23832) | 41709 (32539, 51907) | 84.95 (47.21, 128.61) | 69.29 (65.07, 73.22) | 87.3 (68.11, 108.65) | 26 (0.29, 55.74) | 150.98 (139.43, 160.4) | 75.34 (59.01, 93.92) | -50.1 (-60.13, -38.27) | -2.46 (-2.97, -1.96) | -2.73 (-2.83, -2.63) |
| Comoros | 208 (138, 263) | 443 (336, 580) | 113.12 (55.31, 225.73) | 44.65 (29.68, 56.4) | 62.04 (47.03, 81.16) | 38.95 (1.26, 112.37) | 113.29 (81.31, 141.11) | 105.59 (80.15, 137.2) | -6.8 (-29.91, 33.15) | -0.19 (-0.35, -0.04) | -0.31 (-1.19, 0.58) |
| Congo | 1484 (1202, 1832) | 2797 (2094, 3721) | 88.49 (42.83, 143.91) | 60.69 (49.18, 74.94) | 53.11 (39.77, 70.66) | -12.49 (-33.69, 13.24) | 170.89 (136.88, 213.66) | 140.89 (105.16, 183.06) | -17.56 (-35.3, 4.07) | -0.63 (-0.79, -0.47) | -1.41 (-1.75, -1.08) |
| Cook Islands | 16 (14, 19) | 24 (21, 29) | 53.32 (20.96, 94.13) | 83.37 (72.1, 98.49) | 135.18 (114.05, 159.82) | 62.14 (27.92, 105.29) | 138.73 (120.55, 163.12) | 103.41 (87.32, 122.36) | -25.46 (-40.29, -6.08) | -1 (-1.15, -0.84) | -0.91 (-5.16, 3.52) |
| Costa Rica | 2108 (1938, 2230) | 3708 (2908, 4606) | 75.94 (43.27, 117.27) | 69.33 (63.74, 73.37) | 78.62 (61.66, 97.65) | 13.39 (-7.67, 40.03) | 131.23 (119.56, 139.21) | 71.47 (55.99, 88.87) | -45.53 (-55.71, -32.56) | -2.1 (-2.53, -1.67) | -2.15 (-2.53, -1.77) |
| Croatia | 14858 (13875, 15668) | 13197 (10664, 15822) | -11.17 (-27.08, 6.91) | 303.18 (283.12, 319.71) | 310.68 (251.03, 372.46) | 2.47 (-15.88, 23.33) | 274.03 (251.89, 289.74) | 143.78 (116.19, 172.7) | -47.53 (-56.93, -37.16) | -2.29 (-2.55, -2.03) | -3.04 (-3.86, -2.22) |
| Cuba | 19951 (18686, 20653) | 22272 (18670, 26462) | 11.63 (-4.68, 32.07) | 184.18 (172.5, 190.66) | 196.08 (164.37, 232.97) | 6.46 (-9.1, 25.96) | 205.84 (191.09, 213.78) | 109.85 (92.08, 130.99) | -46.64 (-54.8, -36.8) | -1.98 (-2.44, -1.53) | -2.87 (-3.11, -2.64) |
| Cyprus | 1382 (1281, 1532) | 1596 (1394, 1895) | 15.42 (-3.95, 36.64) | 177.7 (164.7, 196.88) | 121.49 (106.12, 144.26) | -31.63 (-43.11, -19.07) | 228.2 (209.95, 252.6) | 95.5 (82.8, 114) | -58.15 (-65.04, -50.65) | -3.21 (-3.54, -2.88) | -3.42 (-4.11, -2.72) |
| Czechia | 42797 (40685, 43965) | 32960 (27314, 38620) | -22.99 (-34.94, -10.7) | 415.61 (395.09, 426.95) | 309.67 (256.63, 362.85) | -25.49 (-37.06, -13.6) | 327.31 (308.75, 337.15) | 148.97 (123.54, 174.69) | -54.49 (-61.49, -47.08) | -2.63 (-3.03, -2.23) | -4.2 (-4.72, -3.66) |
| Côte d'Ivoire | 4116 (3432, 4940) | 9650 (7569, 12074) | 134.46 (85.25, 196.14) | 33.66 (28.07, 40.4) | 36.87 (28.92, 46.13) | 9.53 (-13.46, 38.35) | 145.96 (124.73, 170.32) | 121.97 (99.42, 146.76) | -16.44 (-30.91, 1.28) | -0.66 (-0.76, -0.55) | -0.81 (-0.97, -0.65) |
| Democratic People's Republic of Korea | 13309 (10352, 16540) | 35798 (30116, 42401) | 168.98 (113.14, 244.21) | 63.21 (49.17, 78.55) | 136.46 (114.8, 161.63) | 115.89 (71.07, 176.27) | 107.69 (84.02, 133.16) | 126.54 (106.78, 150.01) | 17.51 (-6.46, 49.68) | 0.6 (0.46, 0.73) | 0.65 (0.56, 0.75) |
| Democratic Republic of the Congo | 15377 (12279, 19958) | 31795 (23189, 42849) | 106.76 (58.63, 169.05) | 39.85 (31.82, 51.72) | 36.27 (26.45, 48.87) | -8.99 (-30.17, 18.43) | 127.83 (102.97, 164.05) | 114.72 (83.9, 153.66) | -10.25 (-29.48, 13.51) | -0.37 (-0.55, -0.2) | -0.54 (-0.64, -0.44) |
| Denmark | 17890 (16790, 18513) | 7170 (6278, 7763) | -59.92 (-62.73, -57.23) | 347.78 (326.39, 359.88) | 123.56 (108.19, 133.78) | -64.47 (-66.96, -62.08) | 209.7 (196.62, 216.93) | 55.55 (49.11, 59.99) | -73.51 (-75.18, -71.75) | -4.45 (-4.67, -4.22) | -6 (-6.82, -5.18) |
| Djibouti | 96 (75, 124) | 496 (346, 686) | 415.54 (251.4, 621.28) | 19.78 (15.36, 25.61) | 41.21 (28.78, 57.05) | 108.31 (41.99, 191.44) | 99.93 (80.38, 124.65) | 115.99 (84.39, 154.53) | 16.07 (-16.39, 54.26) | 0.47 (0.37, 0.57) | 0.5 (-0.38, 1.4) |
| Dominica | 108 (98, 117) | 81 (69, 96) | -24.94 (-36.79, -11.32) | 146.13 (132.67, 158.15) | 118.18 (99.82, 140) | -19.12 (-31.89, -4.44) | 146.78 (133.77, 158.86) | 89.38 (75.54, 105.94) | -39.1 (-48.72, -27.91) | -1.72 (-1.86, -1.57) | -2.13 (-5.08, 0.93) |
| Dominican Republic | 4386 (3887, 4862) | 15306 (12105, 19102) | 248.99 (170.48, 341.18) | 60.89 (53.96, 67.5) | 140.66 (111.24, 175.54) | 131.01 (79.04, 192.03) | 139.46 (123.04, 154.44) | 175.57 (139.73, 217.55) | 25.89 (-0.99, 57.93) | 1.31 (0.76, 1.86) | 1.51 (1.33, 1.68) |
| Ecuador | 4709 (4384, 4951) | 10684 (8641, 13329) | 126.88 (85.03, 181.34) | 46.97 (43.72, 49.38) | 60.74 (49.13, 75.78) | 29.33 (5.47, 60.37) | 103.97 (95.51, 109.69) | 81.81 (66.65, 101.43) | -21.31 (-35.19, -3.05) | -1 (-1.56, -0.43) | -1.02 (-1.25, -0.79) |
| Egypt | 96748 (89583, 106510) | 181885 (138959, 233632) | 88 (43.65, 138.61) | 173.7 (160.84, 191.23) | 183.59 (140.26, 235.83) | 5.69 (-19.24, 34.15) | 410.76 (377.9, 449.55) | 359.27 (281.82, 447.03) | -12.54 (-32.42, 8.77) | -0.45 (-0.96, 0.07) | -0.56 (-0.69, -0.42) |
| El Salvador | 3514 (3208, 3728) | 6482 (5055, 8121) | 84.45 (45.29, 129.3) | 66.71 (60.9, 70.78) | 103.61 (80.8, 129.81) | 55.3 (22.33, 93.06) | 123.44 (111.73, 131.21) | 100.37 (78.13, 125.94) | -18.69 (-36.45, 2.47) | -0.55 (-1.05, -0.04) | -1.49 (-1.71, -1.27) |
| Equatorial Guinea | 236 (175, 309) | 349 (249, 488) | 47.95 (-1.27, 117.32) | 54.84 (40.73, 71.76) | 24.59 (17.52, 34.39) | -55.16 (-70.08, -34.13) | 141.58 (104, 188.98) | 97.07 (69.42, 129.45) | -31.44 (-54.59, -0.99) | -1.31 (-1.53, -1.09) | -2.9 (-3.75, -2.03) |
| Eritrea | 733 (525, 1018) | 2273 (1745, 2939) | 210.07 (121.55, 343.4) | 24.42 (17.5, 33.92) | 33.86 (26.01, 43.79) | 38.67 (-0.92, 98.3) | 94.51 (67.96, 129.84) | 113.46 (89.36, 142.45) | 20.05 (-14.16, 64.86) | 0.63 (0.56, 0.71) | 0.39 (0.03, 0.75) |
| Estonia | 6984 (6628, 7230) | 4434 (3512, 5893) | -36.51 (-48.87, -15.05) | 445.22 (422.54, 460.89) | 337.88 (267.63, 449.01) | -24.11 (-38.88, 1.55) | 367.19 (345.24, 381.25) | 144.16 (114.15, 191.36) | -60.74 (-68.56, -47.91) | -3.71 (-4.23, -3.18) | -5.31 (-6.24, -4.37) |
| Eswatini | 265 (219, 315) | 549 (405, 734) | 106.83 (50.25, 182.74) | 32.89 (27.09, 39) | 48.05 (35.48, 64.29) | 46.09 (6.12, 99.7) | 113.1 (93.79, 132.6) | 121.58 (91.33, 158.57) | 7.49 (-21.07, 42.36) | 0.21 (0.03, 0.39) | 0.81 (0.07, 1.55) |
| Ethiopia | 18776 (14705, 24271) | 28409 (21139, 35645) | 51.3 (-9.95, 118.46) | 36.54 (28.61, 47.23) | 26.4 (19.65, 33.13) | -27.73 (-56.99, 4.35) | 110.26 (84.68, 140.93) | 84.24 (62.56, 105.75) | -23.6 (-54.12, 8.08) | -0.98 (-1.07, -0.89) | -2.23 (-2.34, -2.13) |
| Fiji | 932 (792, 1098) | 1575 (1256, 1939) | 69.04 (29.26, 124.63) | 122.68 (104.31, 144.64) | 172.8 (137.81, 212.8) | 40.86 (7.71, 87.18) | 290.32 (250.29, 334.86) | 249.94 (205.02, 302.15) | -13.91 (-32.42, 10.31) | -0.53 (-0.86, -0.19) | -0.83 (-1.24, -0.42) |
| Finland | 16022 (15165, 16565) | 14812 (12840, 16036) | -7.55 (-15.29, -0.45) | 319.79 (302.68, 330.62) | 267.66 (232.02, 289.76) | -16.3 (-23.31, -9.88) | 226.02 (212.49, 234.15) | 100.57 (88.89, 108.53) | -55.5 (-58.56, -52.17) | -2.72 (-2.94, -2.49) | -4.06 (-4.68, -3.43) |
| France | 76451 (69917, 81025) | 68325 (57932, 75790) | -10.63 (-18.08, -3.98) | 132.34 (121.03, 140.25) | 103.2 (87.5, 114.48) | -22.02 (-28.52, -16.21) | 87.54 (80.21, 92.65) | 38.41 (33.46, 41.9) | -56.13 (-58.77, -53.6) | -2.78 (-2.98, -2.57) | -3.11 (-3.49, -2.74) |
| Gabon | 625 (510, 757) | 987 (771, 1222) | 57.83 (23.42, 97.02) | 63.05 (51.39, 76.38) | 56.38 (44.04, 69.83) | -10.58 (-30.07, 11.63) | 131.98 (106.22, 158.46) | 117.37 (91.71, 146.47) | -11.07 (-28.47, 9.75) | -0.34 (-0.49, -0.18) | -0.86 (-1.42, -0.3) |
| Gambia | 353 (282, 440) | 1206 (978, 1458) | 241.14 (154.02, 343.85) | 35.63 (28.43, 44.39) | 53.68 (43.53, 64.92) | 50.68 (12.19, 96.04) | 133.3 (109.84, 161.37) | 151.23 (125.37, 180.37) | 13.45 (-12.13, 42.75) | 0.39 (-0.26, 1.05) | 0.47 (-0.14, 1.09) |
| Georgia | 21989 (20455, 23532) | 13917 (11826, 16059) | -36.71 (-45.97, -24.46) | 399.17 (371.32, 427.18) | 379.75 (322.69, 438.19) | -4.87 (-18.78, 13.55) | 429.78 (398.28, 458.58) | 212.6 (181.64, 245.68) | -50.53 (-57.55, -41.15) | -2.38 (-3.23, -1.51) | -3.35 (-3.79, -2.91) |
| Germany | 271491 (251300, 283223) | 184323 (164517, 197583) | -32.11 (-36.04, -27.03) | 339.61 (314.36, 354.29) | 217.07 (193.75, 232.69) | -36.08 (-39.79, -31.31) | 208.33 (192.37, 217.72) | 81.42 (73.29, 86.75) | -60.92 (-62.92, -58.14) | -3.2 (-3.37, -3.04) | -4.13 (-4.67, -3.6) |
| Ghana | 5877 (4874, 6952) | 15888 (13341, 18985) | 170.33 (112.68, 239.04) | 39.14 (32.46, 46.29) | 50.38 (42.3, 60.2) | 28.72 (1.27, 61.44) | 124.79 (105.22, 145.29) | 128.55 (110.06, 151.39) | 3.02 (-16.55, 26.71) | 0.08 (0, 0.16) | -0.09 (-0.24, 0.05) |
| Greece | 20671 (19505, 21487) | 26142 (22942, 28068) | 26.47 (17.21, 34.62) | 198.96 (187.73, 206.81) | 252.9 (221.94, 271.53) | 27.11 (17.8, 35.31) | 143.33 (134.33, 149.32) | 91.92 (82.51, 97.66) | -35.87 (-39.09, -32.44) | -1.51 (-1.83, -1.19) | -1.45 (-1.77, -1.13) |
| Greenland | 54 (49, 61) | 56 (47, 66) | 3.15 (-17.59, 24.84) | 97.8 (88.02, 109.68) | 99.79 (82.84, 117.26) | 2.03 (-18.49, 23.48) | 203.09 (184.28, 225.04) | 96.19 (81.2, 111.86) | -52.63 (-61.16, -43.72) | -2.63 (-2.91, -2.35) | -3.48 (-8.06, 1.33) |
| Grenada | 151 (139, 162) | 105 (96, 114) | -30.35 (-36.63, -23.31) | 176.3 (162, 189.12) | 101.92 (93.08, 110.62) | -42.19 (-47.4, -36.34) | 191.33 (176.19, 205.08) | 107.05 (97.17, 116.31) | -44.05 (-49.19, -38.74) | -2.04 (-2.52, -1.55) | -2.36 (-4.57, -0.1) |
| Guam | 112 (100, 126) | 320 (273, 374) | 186.22 (136.36, 244.61) | 81.85 (73.16, 91.93) | 187.77 (160.24, 219.43) | 129.41 (89.44, 176.21) | 200.48 (177.99, 225.91) | 171.76 (146.81, 200.85) | -14.32 (-29.31, 2.29) | -0.35 (-0.91, 0.21) | 0.27 (-0.88, 1.44) |
| Guatemala | 4539 (4073, 5000) | 9408 (7651, 11328) | 107.27 (67.71, 156.74) | 56.98 (51.13, 62.76) | 52.93 (43.04, 63.73) | -7.11 (-24.84, 15.06) | 164.51 (148.91, 179.73) | 106.32 (87.43, 126.24) | -35.37 (-46.47, -22.26) | -1.47 (-1.91, -1.02) | -2.5 (-2.66, -2.35) |
| Guinea | 3010 (2446, 3678) | 5826 (4758, 7227) | 93.59 (51.24, 149.02) | 48.65 (39.53, 59.45) | 46.08 (37.64, 57.16) | -5.28 (-26, 21.85) | 108.92 (88.54, 132.73) | 123.67 (101.51, 152.11) | 13.54 (-10.17, 43.13) | 0.51 (0.42, 0.6) | 1.11 (0.86, 1.37) |
| Guinea-Bissau | 550 (429, 712) | 936 (735, 1173) | 70.19 (23.2, 138.71) | 54.62 (42.55, 70.67) | 49.25 (38.67, 61.72) | -9.83 (-34.72, 26.48) | 166.36 (132.3, 209.29) | 164.54 (131.86, 202.57) | -1.09 (-26.15, 34.31) | 0.01 (-0.05, 0.07) | 0.16 (-0.36, 0.67) |
| Guyana | 912 (809, 1012) | 1047 (837, 1294) | 14.81 (-10.55, 46.1) | 118.44 (105.09, 131.37) | 135.86 (108.54, 167.84) | 14.71 (-10.63, 45.98) | 274.3 (245.76, 301.72) | 192.95 (155.81, 233.9) | -29.66 (-44.01, -12.64) | -1.22 (-1.42, -1.01) | -1.43 (-1.95, -0.9) |
| Haiti | 6649 (5510, 8100) | 11149 (7990, 15413) | 67.68 (26.85, 120.71) | 104.61 (86.7, 127.45) | 89.89 (64.42, 124.28) | -14.07 (-34.99, 13.11) | 244.73 (201.12, 294.91) | 196.02 (142.67, 265.82) | -19.9 (-37.45, 2.89) | -0.71 (-0.8, -0.62) | -0.79 (-0.95, -0.62) |
| Honduras | 2190 (1812, 2875) | 7606 (6222, 9374) | 247.39 (188.84, 327.79) | 46.5 (38.48, 61.06) | 77.5 (63.39, 95.52) | 66.66 (38.57, 105.24) | 121.34 (99.9, 161.48) | 154.84 (126.87, 189.4) | 27.61 (7.1, 56.65) | 0.72 (-0.4, 1.85) | -0.49 (-0.74, -0.25) |
| Hungary | 38088 (36469, 39244) | 35931 (30178, 41877) | -5.67 (-19.94, 10.17) | 366.49 (350.91, 377.62) | 371.4 (311.94, 432.86) | 1.34 (-14, 18.35) | 285.59 (270.35, 295.23) | 174.62 (146.49, 203.95) | -38.86 (-47.65, -28.68) | -1.73 (-1.93, -1.52) | -3.19 (-3.5, -2.87) |
| Iceland | 512 (467, 540) | 436 (368, 483) | -14.72 (-23.24, -7.02) | 201.45 (183.99, 212.46) | 126.51 (106.75, 139.98) | -37.2 (-43.48, -31.53) | 170.77 (156.49, 179.98) | 66.24 (56.82, 72.98) | -61.21 (-64.78, -57.65) | -3.25 (-3.47, -3.02) | -3.93 (-5.53, -2.3) |
| India | 621764 (550975, 691893) | 1519124 (1311372, 1745629) | 144.32 (100.55, 185.66) | 72.67 (64.4, 80.87) | 109.23 (94.3, 125.52) | 50.31 (23.38, 75.74) | 170.52 (150.69, 190.21) | 150.51 (129.37, 172.58) | -11.74 (-26.57, 2.47) | -0.44 (-0.56, -0.32) | -0.48 (-0.63, -0.33) |
| Indonesia | 97860 (86022, 109594) | 245344 (207414, 275666) | 150.71 (104.72, 198.88) | 52.79 (46.4, 59.12) | 94.56 (79.94, 106.24) | 79.12 (46.26, 113.54) | 118.05 (103.39, 132.74) | 140.33 (119.78, 154.99) | 18.87 (-1.51, 41.46) | 0.63 (0.58, 0.67) | 0.42 (0.37, 0.47) |
| Iran (Islamic Republic of) | 55668 (51802, 60039) | 102799 (94455, 111215) | 84.66 (65.27, 100.03) | 95.09 (88.49, 102.56) | 121.95 (112.05, 131.93) | 28.24 (14.77, 38.91) | 285.68 (260.83, 307.09) | 163.56 (148.98, 176.17) | -42.75 (-47.92, -38.07) | -1.86 (-1.94, -1.78) | -2.8 (-2.89, -2.71) |
| Iraq | 21556 (18422, 24921) | 46848 (38263, 55511) | 117.33 (71.65, 167.11) | 122.5 (104.69, 141.62) | 111.23 (90.84, 131.79) | -9.2 (-28.29, 11.59) | 311.21 (265.52, 357.49) | 255.4 (214.11, 292.61) | -17.93 (-33.55, -2.03) | -0.69 (-1, -0.38) | -1.85 (-1.97, -1.74) |
| Ireland | 9100 (8650, 9404) | 5810 (5084, 6283) | -36.16 (-42.08, -31.84) | 252.69 (240.21, 261.13) | 118.32 (103.54, 127.95) | -53.18 (-57.52, -50.01) | 231.3 (217.7, 239.56) | 74.7 (65.6, 80.72) | -67.7 (-70.39, -65.68) | -3.91 (-4.07, -3.74) | -5.05 (-5.67, -4.41) |
| Israel | 8133 (7698, 8403) | 6166 (5376, 6664) | -24.18 (-30.75, -19.33) | 163.91 (155.15, 169.34) | 66.23 (57.75, 71.59) | -59.59 (-63.09, -57.01) | 179.16 (167.27, 185.73) | 48.13 (42.38, 51.86) | -73.13 (-75.08, -71.63) | -4.51 (-4.83, -4.19) | -5.88 (-6.29, -5.46) |
| Italy | 103619 (97061, 112129) | 101159 (85415, 111561) | -2.37 (-12.92, 3.1) | 182.43 (170.89, 197.42) | 167.72 (141.62, 184.97) | -8.06 (-17.99, -2.91) | 120.45 (111.29, 131.34) | 55.27 (47.69, 60.39) | -54.11 (-57.59, -52.14) | -2.68 (-2.83, -2.52) | -3.36 (-3.66, -3.05) |
| Jamaica | 1518 (1374, 1665) | 2109 (1738, 2520) | 38.92 (14.98, 66.85) | 64.24 (58.13, 70.45) | 75.05 (61.82, 89.67) | 16.82 (-3.31, 40.31) | 83.88 (75.63, 91.89) | 64.28 (52.47, 77.27) | -23.37 (-37.08, -7.3) | -0.82 (-1.36, -0.28) | -0.61 (-1.19, -0.03) |
| Japan | 115054 (105827, 121083) | 140588 (111886, 156600) | 22.19 (5.53, 31.76) | 91.41 (84.08, 96.2) | 110.02 (87.56, 122.55) | 20.36 (3.94, 29.78) | 76.16 (69.04, 80.48) | 29.87 (25.15, 32.5) | -60.78 (-63.8, -58.87) | -3.2 (-3.45, -2.94) | -2.42 (-2.84, -2) |
| Jordan | 2367 (2076, 2692) | 6111 (5196, 7286) | 158.16 (111.47, 221.95) | 62.74 (55.01, 71.34) | 52.51 (44.65, 62.61) | -16.29 (-31.43, 4.39) | 225.39 (197.81, 254.57) | 121.92 (103.15, 144.05) | -45.91 (-55.02, -34.59) | -2.15 (-2.65, -1.66) | -3.08 (-3.31, -2.84) |
| Kazakhstan | 32727 (30895, 34133) | 33047 (28771, 37362) | 0.98 (-11.2, 13.68) | 199.95 (188.75, 208.54) | 179.68 (156.43, 203.14) | -10.14 (-20.97, 1.17) | 303.23 (284.47, 316.96) | 251.41 (219.73, 281.88) | -17.09 (-26.29, -7.71) | -0.79 (-1.05, -0.52) | -2.9 (-3.31, -2.48) |
| Kenya | 4171 (3453, 5051) | 13717 (10678, 17133) | 228.87 (180.57, 283.66) | 17.98 (14.89, 21.78) | 27.31 (21.26, 34.11) | 51.86 (29.56, 77.16) | 66.79 (55.37, 80.64) | 82.59 (63.74, 103.66) | 23.65 (6.18, 42.46) | 0.7 (0.62, 0.77) | 1.3 (1.1, 1.49) |
| Kiribati | 96 (79, 112) | 158 (125, 196) | 64.92 (27.12, 116.24) | 129.03 (106.23, 151.76) | 132.86 (105.71, 165.47) | 2.97 (-20.63, 35.01) | 265.16 (219.66, 310.63) | 249.8 (202.36, 300.91) | -5.79 (-24.76, 19.79) | -0.27 (-0.44, -0.09) | -0.34 (-1.59, 0.92) |
| Kuwait | 1020 (948, 1098) | 2599 (2165, 3105) | 154.7 (113.73, 204.2) | 58 (53.86, 62.39) | 58.72 (48.92, 70.15) | 1.24 (-15.05, 20.91) | 194.57 (175.56, 208.87) | 108.53 (90.73, 129.2) | -44.22 (-52.68, -34.04) | -2.28 (-3.74, -0.79) | -1.91 (-2.22, -1.61) |
| Kyrgyzstan | 6686 (6219, 7091) | 11483 (10202, 12763) | 71.76 (53.32, 90.83) | 149.83 (139.38, 158.92) | 175.7 (156.1, 195.3) | 17.27 (4.69, 30.29) | 235.38 (218.42, 249.91) | 320.39 (283.02, 354.78) | 36.12 (22.12, 50.21) | 1.07 (0.62, 1.53) | -0.56 (-0.99, -0.13) |
| Lao People's Democratic Republic | 2953 (2349, 3729) | 5675 (4637, 6809) | 92.19 (43.75, 149.33) | 71.12 (56.59, 89.82) | 79.28 (64.78, 95.12) | 11.47 (-16.63, 44.61) | 171.56 (137.42, 217.5) | 162.08 (134.69, 189.79) | -5.52 (-26.93, 19.87) | -0.21 (-0.26, -0.16) | -0.53 (-0.74, -0.32) |
| Latvia | 11446 (10869, 11802) | 9021 (7678, 10554) | -21.19 (-31.79, -8.5) | 430.51 (408.8, 443.91) | 470.99 (400.9, 551.03) | 9.4 (-5.31, 27.01) | 333.2 (314.85, 344.38) | 200.43 (171.64, 234.57) | -39.85 (-47.71, -30.17) | -1.49 (-2.45, -0.51) | -3.56 (-4.4, -2.71) |
| Lebanon | 6766 (5873, 7839) | 12251 (8866, 14092) | 81.06 (35.06, 111.96) | 206.6 (179.31, 239.35) | 236.65 (171.25, 272.21) | 14.54 (-14.56, 34.09) | 352.64 (307.84, 405.86) | 241.23 (174.11, 277.12) | -31.59 (-48.89, -20.45) | -1.29 (-1.36, -1.22) | -1.18 (-1.35, -1) |
| Lesotho | 697 (540, 850) | 1270 (934, 1653) | 82.22 (35.53, 145.94) | 38.58 (29.86, 47.02) | 60.74 (44.67, 79.02) | 57.45 (17.11, 112.5) | 84.65 (65.14, 103.19) | 123.63 (91.41, 157.92) | 46.06 (10.09, 92.3) | 1.45 (1.21, 1.68) | 2.52 (1.95, 3.08) |
| Liberia | 1244 (1042, 1485) | 1888 (1473, 2448) | 51.82 (15.82, 99.05) | 63.32 (53.07, 75.61) | 39.42 (30.76, 51.11) | -37.74 (-52.51, -18.37) | 136.62 (116.33, 161.16) | 117.44 (93.65, 148.15) | -14.04 (-33.47, 10.41) | -0.62 (-0.77, -0.48) | -0.36 (-0.77, 0.05) |
| Libya | 3204 (2594, 4006) | 7827 (6177, 10363) | 144.29 (90.31, 215.04) | 75.62 (61.22, 94.55) | 116.2 (91.71, 153.85) | 53.67 (19.71, 98.17) | 189.88 (153.37, 236.74) | 171.3 (135.45, 226.05) | -9.78 (-29.12, 14.75) | -0.17 (-0.56, 0.23) | -0.42 (-0.6, -0.25) |
| Lithuania | 15400 (14407, 15936) | 14638 (12214, 17200) | -4.95 (-18.52, 10.6) | 419.21 (392.17, 433.81) | 523.86 (437.13, 615.56) | 24.96 (7.13, 45.41) | 352.18 (327.79, 365.01) | 222.93 (186.66, 262.51) | -36.7 (-45.96, -25.98) | -1.28 (-2.41, -0.15) | -2.36 (-3.07, -1.64) |
| Luxembourg | 861 (808, 899) | 617 (518, 702) | -28.36 (-37.7, -19.34) | 225.73 (211.87, 235.67) | 99.68 (83.7, 113.52) | -55.84 (-61.6, -50.29) | 162.85 (151.84, 170.13) | 54.17 (45.97, 61.62) | -66.74 (-70.82, -62.62) | -3.73 (-4.04, -3.42) | -4.81 (-6.24, -3.36) |
| Madagascar | 5331 (4555, 6141) | 10714 (7666, 14409) | 100.97 (47.96, 167.53) | 44.61 (38.12, 51.38) | 40.14 (28.72, 53.98) | -10.01 (-33.75, 19.8) | 128.65 (109.88, 149.41) | 132.35 (98.19, 172.78) | 2.87 (-22.65, 33.79) | 0.09 (-0.1, 0.28) | -0.18 (-0.31, -0.04) |
| Malawi | 3047 (2537, 3566) | 5471 (4286, 6783) | 79.53 (35.54, 135.03) | 31.89 (26.55, 37.32) | 29.66 (23.24, 36.78) | -6.98 (-29.77, 21.78) | 97.25 (82.29, 112.98) | 90.11 (71.03, 111.3) | -7.34 (-29.13, 18.19) | -0.28 (-0.39, -0.16) | -0.63 (-0.83, -0.44) |
| Malaysia | 13175 (12279, 14043) | 34010 (27126, 41375) | 158.15 (104.13, 217.73) | 74.62 (69.55, 79.54) | 108.65 (86.66, 132.18) | 45.6 (15.14, 79.21) | 165.58 (152.67, 176.84) | 145.71 (117.74, 175.96) | -12 (-28.98, 6.88) | -0.83 (-1.33, -0.33) | -0.32 (-0.52, -0.12) |
| Maldives | 185 (165, 212) | 308 (260, 360) | 66.39 (34.54, 103.78) | 83.37 (74.57, 95.61) | 61.76 (52.09, 72.15) | -25.91 (-40.09, -9.27) | 265.31 (238.41, 300.3) | 120.91 (101.79, 141.05) | -54.43 (-62.96, -44.77) | -2.83 (-3.16, -2.49) | -4.03 (-4.98, -3.06) |
| Mali | 3863 (3192, 4607) | 7596 (6053, 9386) | 96.63 (54.49, 145.72) | 44.54 (36.8, 53.12) | 34.66 (27.62, 42.82) | -22.19 (-38.87, -2.77) | 127.74 (106.37, 151.15) | 116.05 (93.53, 140.92) | -9.15 (-28.53, 10.97) | -0.31 (-0.41, -0.21) | -0.44 (-0.66, -0.23) |
| Malta | 850 (803, 890) | 920 (795, 1017) | 8.22 (-3.17, 19.18) | 229.33 (216.65, 240.08) | 209.45 (180.96, 231.59) | -8.67 (-18.28, 0.59) | 214.12 (200.55, 224.69) | 90.83 (78.79, 100.58) | -57.58 (-61.64, -53.3) | -3.04 (-3.23, -2.84) | -3.05 (-4.26, -1.82) |
| Marshall Islands | 38 (30, 46) | 80 (60, 107) | 111.8 (67.43, 171.08) | 82.93 (66.31, 100.57) | 141.3 (105.93, 188.22) | 70.39 (34.69, 118.08) | 263.15 (210.09, 321.66) | 277.78 (214.38, 356.28) | 5.56 (-15.27, 30.73) | 0.11 (-0.08, 0.29) | 0.41 (-1.37, 2.21) |
| Mauritania | 1273 (1075, 1490) | 1827 (1486, 2256) | 43.44 (12.44, 78.64) | 61.63 (52.02, 72.11) | 45.5 (37.01, 56.2) | -26.16 (-42.12, -8.04) | 152.39 (129.91, 178.25) | 106.02 (87.31, 127.8) | -30.42 (-44.31, -14.21) | -1.25 (-1.38, -1.12) | -1.66 (-2.12, -1.2) |
| Mauritius | 1724 (1650, 1784) | 1663 (1382, 1977) | -3.52 (-19.33, 14.44) | 156.73 (150.02, 162.19) | 130.3 (108.22, 154.88) | -16.87 (-30.49, -1.39) | 264.42 (250.03, 275.23) | 103.82 (86.23, 123.12) | -60.74 (-66.98, -53.57) | -3.31 (-3.71, -2.91) | -4.08 (-4.52, -3.64) |
| Mexico | 39678 (37394, 41037) | 106311 (91980, 120365) | 167.93 (134.78, 202.77) | 46.41 (43.74, 48) | 85.09 (73.62, 96.34) | 83.33 (60.65, 107.16) | 114.42 (105.95, 118.92) | 100 (85.98, 112.92) | -12.6 (-23.12, -1.77) | -0.35 (-0.55, -0.15) | -0.12 (-0.28, 0.04) |
| Micronesia (Federated States of) | 111 (83, 144) | 173 (123, 227) | 56.44 (8.5, 113.53) | 106.17 (79.46, 138.46) | 169.61 (120.79, 222.52) | 59.76 (10.8, 118.06) | 266.89 (203.34, 343.21) | 287.23 (216.68, 365.42) | 7.62 (-20, 41.86) | 0.24 (0.21, 0.28) | 0.2 (-0.94, 1.36) |
| Monaco | 103 (84, 120) | 80 (64, 92) | -22.78 (-37.45, -4.13) | 339 (275.83, 394.08) | 212.09 (170.69, 245.27) | -37.44 (-49.32, -22.33) | 127.91 (103.95, 149.26) | 67.21 (53.8, 77.91) | -47.46 (-58.26, -34.05) | -2.16 (-2.32, -2.01) | -2.44 (-8.03, 3.48) |
| Mongolia | 3460 (2949, 3938) | 4906 (3934, 6128) | 41.79 (11.35, 84.98) | 160.65 (136.94, 182.86) | 144.81 (116.14, 180.89) | -9.86 (-29.21, 17.6) | 405.25 (349.3, 458.09) | 311.63 (260.94, 376.5) | -23.1 (-36.67, -4.23) | -0.83 (-1.22, -0.44) | -1.94 (-2.18, -1.71) |
| Montenegro | 917 (829, 1003) | 1488 (1253, 1726) | 62.25 (35.54, 92.75) | 146.59 (132.53, 160.28) | 239.91 (201.91, 278.18) | 63.66 (36.72, 94.43) | 162.45 (145.69, 177.52) | 164.06 (138.59, 189.24) | 0.99 (-14.87, 18.82) | 0.41 (0.09, 0.72) | -1.06 (-2, -0.12) |
| Morocco | 35296 (31031, 39850) | 72012 (56906, 84501) | 104.02 (63.76, 138.82) | 139.53 (122.67, 157.53) | 200.3 (158.28, 235.04) | 43.55 (15.23, 68.04) | 306.23 (264.97, 346.06) | 278.54 (224.66, 321.41) | -9.04 (-25.23, 4.81) | -0.36 (-0.92, 0.21) | -1.08 (-1.25, -0.91) |
| Mozambique | 3757 (3056, 4495) | 8936 (6994, 11428) | 137.84 (79.44, 217.92) | 28.75 (23.38, 34.39) | 30.26 (23.69, 38.7) | 5.28 (-20.57, 40.73) | 80.96 (65.89, 96.86) | 102.04 (80.3, 129.34) | 26.03 (-5.4, 63.45) | 0.85 (0.75, 0.94) | 1.71 (1.51, 1.9) |
| Myanmar | 28988 (22806, 36367) | 40583 (35581, 46986) | 40 (8.12, 82.69) | 70.52 (55.48, 88.48) | 74.22 (65.08, 85.93) | 5.25 (-18.72, 37.34) | 145.85 (117.25, 178.55) | 104.1 (91.37, 119.61) | -28.63 (-43.12, -9.36) | -1.19 (-1.24, -1.14) | -2.08 (-2.16, -1.99) |
| Namibia | 734 (615, 860) | 1374 (1098, 1670) | 87.21 (44.17, 142.38) | 52.06 (43.62, 61.01) | 57.17 (45.68, 69.51) | 9.82 (-15.43, 42.18) | 122.89 (103.51, 142.44) | 115.08 (93.03, 138.54) | -6.35 (-27.06, 18.64) | -0.1 (-0.31, 0.1) | -0.76 (-1.35, -0.17) |
| Nauru | 11 (9, 14) | 13 (10, 16) | 13.41 (-8.31, 40.98) | 111.58 (89.31, 139.51) | 122.98 (98.29, 152.44) | 10.22 (-10.89, 37.01) | 333.59 (277.44, 396.04) | 356.23 (294.34, 424.75) | 6.79 (-11.51, 27.78) | 0.22 (0.13, 0.3) | 0.22 (-3.17, 3.73) |
| Nepal | 8956 (7173, 11377) | 23743 (18714, 28811) | 165.1 (99.94, 239.34) | 45.84 (36.72, 58.23) | 78.06 (61.53, 94.72) | 70.28 (28.42, 117.96) | 109.38 (88.03, 138.31) | 123.99 (98.62, 148.27) | 13.36 (-13.74, 43.35) | 0.42 (0.37, 0.47) | -0.07 (-0.18, 0.04) |
| Netherlands | 29510 (27503, 30770) | 17642 (15552, 19086) | -40.22 (-44.03, -36.66) | 197.75 (184.3, 206.19) | 102.83 (90.64, 111.25) | -48 (-51.32, -44.91) | 146 (135.65, 152.27) | 47.4 (42.06, 51.13) | -67.54 (-69.47, -65.74) | -3.9 (-4.14, -3.67) | -5.27 (-5.81, -4.73) |
| New Zealand | 7177 (6762, 7431) | 6492 (5672, 6995) | -9.55 (-16.46, -4.44) | 210.03 (197.89, 217.44) | 144.4 (126.16, 155.59) | -31.25 (-36.5, -27.36) | 186.4 (174.33, 193.56) | 75 (66.21, 80.44) | -59.77 (-62.19, -57.71) | -3.17 (-3.41, -2.93) | -4.18 (-4.74, -3.62) |
| Nicaragua | 1396 (1268, 1491) | 5091 (4369, 5800) | 264.77 (214.25, 315.34) | 35.91 (32.62, 38.35) | 78.2 (67.11, 89.08) | 117.79 (87.63, 147.98) | 106.9 (95.67, 114.73) | 148.33 (126.91, 166.41) | 38.76 (20.92, 56.23) | 1.13 (0.15, 2.12) | 0.17 (-0.15, 0.49) |
| Niger | 2644 (2082, 3269) | 6756 (5242, 8649) | 155.51 (99.53, 233.22) | 32.96 (25.94, 40.75) | 29 (22.5, 37.13) | -12 (-31.28, 14.76) | 130.48 (103.25, 161.07) | 118.13 (93.96, 146.38) | -9.46 (-27.71, 13.77) | -0.27 (-0.43, -0.11) | -0.46 (-0.69, -0.23) |
| Nigeria | 44673 (32829, 61766) | 69581 (50603, 89625) | 55.76 (-1.77, 110.74) | 49.53 (36.4, 68.49) | 32.39 (23.56, 41.72) | -34.61 (-58.76, -11.53) | 127.88 (96.26, 173.37) | 105.21 (76.6, 132.53) | -17.73 (-48.96, 8.89) | -0.61 (-0.76, -0.47) | -1.15 (-1.24, -1.07) |
| Niue | 5 (4, 6) | 4 (4, 5) | -12.61 (-29.11, 6.7) | 217.42 (185.76, 257.41) | 264.47 (212.98, 312.12) | 21.64 (-1.34, 48.52) | 221.59 (189.88, 261.41) | 208.81 (167.88, 247.91) | -5.77 (-23.81, 15.91) | -0.24 (-0.31, -0.17) | -0.59 (-9.47, 9.15) |
| North Macedonia | 3631 (3396, 3831) | 5156 (4224, 6228) | 42 (15.82, 72.26) | 180.15 (168.5, 190.1) | 239.5 (196.23, 289.29) | 32.94 (8.43, 61.27) | 230.25 (212.91, 243.48) | 209.93 (176.09, 247.29) | -8.83 (-23.64, 9.07) | -0.44 (-0.73, -0.14) | -1.81 (-2.16, -1.46) |
| Northern Mariana Islands | 19 (15, 23) | 64 (55, 74) | 243.61 (171.36, 328.15) | 41.32 (33.79, 50.37) | 151.67 (129.76, 174.1) | 267.09 (189.9, 357.4) | 124.58 (105.28, 147.8) | 148.79 (128.85, 170.16) | 19.44 (-1.93, 44.49) | 0.63 (0.46, 0.79) | 0.78 (-2.14, 3.79) |
| Norway | 13042 (12154, 13497) | 6271 (5449, 6831) | -51.92 (-55.65, -48.26) | 307.1 (286.18, 317.8) | 117.23 (101.88, 127.72) | -61.83 (-64.79, -58.92) | 179.06 (167.38, 185.14) | 55.47 (48.96, 59.95) | -69.02 (-71.04, -66.69) | -4.01 (-4.29, -3.72) | -5.41 (-6.05, -4.78) |
| Oman | 2365 (1859, 2911) | 3412 (3046, 3828) | 44.28 (16.12, 85.11) | 121.69 (95.65, 149.82) | 74.43 (66.44, 83.52) | -38.84 (-50.78, -21.53) | 470.15 (382.97, 562.72) | 329.85 (296.03, 364.09) | -29.84 (-41.94, -13.02) | -1.51 (-2.52, -0.49) | -2.6 (-3.03, -2.17) |
| Pakistan | 75992 (63096, 87555) | 183409 (152233, 220359) | 141.35 (95.89, 197.3) | 67.35 (55.92, 77.59) | 81.86 (67.94, 98.35) | 21.54 (-1.35, 49.72) | 146 (121.02, 169.53) | 189.25 (158.03, 225.54) | 29.62 (7.05, 57.67) | 0.97 (0.88, 1.06) | 0.87 (0.8, 0.94) |
| Palau | 21 (17, 27) | 41 (33, 51) | 94.28 (43.47, 160.17) | 136.4 (107.73, 177.63) | 226.69 (181.52, 283.11) | 66.19 (22.72, 122.56) | 238.21 (189.55, 305.38) | 222.94 (180.38, 270.27) | -6.41 (-29.96, 23.92) | -0.16 (-0.32, 0) | -0.11 (-2.92, 2.78) |
| Palestine | 2216 (1798, 2715) | 3810 (3326, 4366) | 71.92 (36.83, 120.57) | 107.06 (86.84, 131.16) | 76.86 (67.1, 88.09) | -28.2 (-42.86, -7.88) | 289.88 (238.1, 349.39) | 207.16 (180.07, 236.15) | -28.54 (-42.26, -9.25) | -1.14 (-1.27, -1) | -2.09 (-2.38, -1.8) |
| Panama | 1589 (1437, 1681) | 2511 (1950, 3136) | 58.03 (26.55, 94.07) | 66.52 (60.17, 70.37) | 60.35 (46.86, 75.37) | -9.28 (-27.35, 11.41) | 114.98 (103.25, 121.99) | 58.22 (45.13, 72.81) | -49.37 (-59.49, -37.55) | -2.46 (-2.86, -2.07) | -1.88 (-2.34, -1.41) |
| Papua New Guinea | 2196 (1547, 3194) | 7084 (5169, 9722) | 222.51 (143.76, 333.11) | 53.74 (37.85, 78.13) | 71.8 (52.39, 98.54) | 33.61 (0.98, 79.43) | 136.8 (96.91, 193.73) | 171.98 (126.71, 231.3) | 25.71 (-0.93, 61.95) | 0.79 (0.72, 0.86) | 0.97 (0.75, 1.19) |
| Paraguay | 2220 (1945, 2464) | 4753 (3732, 5991) | 114.04 (64.8, 172.95) | 54.89 (48.07, 60.91) | 68.58 (53.85, 86.45) | 24.94 (-3.8, 59.33) | 109.02 (94.77, 121.23) | 89.6 (70.25, 112.93) | -17.82 (-36.67, 4.81) | -0.45 (-0.9, -0.01) | -0.68 (-0.98, -0.38) |
| Peru | 11274 (9750, 12897) | 16048 (11891, 20492) | 42.34 (4.36, 91.79) | 51.88 (44.87, 59.35) | 47.21 (34.98, 60.28) | -9.01 (-33.29, 22.59) | 105.12 (90.6, 119.84) | 48.68 (35.92, 62.26) | -53.69 (-66.04, -38.02) | -2.8 (-3.85, -1.74) | -2.78 (-2.91, -2.65) |
| Philippines | 20418 (17897, 24221) | 97412 (79700, 114687) | 377.09 (248.47, 480.86) | 32.26 (28.28, 38.27) | 86.86 (71.07, 102.27) | 169.25 (96.66, 227.81) | 103.82 (91.56, 115.69) | 148.14 (123.37, 171.56) | 42.69 (13.99, 69.24) | 1.54 (1.21, 1.88) | 3.24 (3, 3.48) |
| Poland | 128572 (122025, 131893) | 97188 (81865, 112079) | -24.41 (-34.62, -13.42) | 336.95 (319.79, 345.65) | 252.87 (213, 291.61) | -24.95 (-35.09, -14.04) | 321.93 (302.17, 331.82) | 130.31 (110.03, 150.64) | -59.52 (-65.01, -53.71) | -3.39 (-3.67, -3.11) | -4.75 (-5.14, -4.36) |
| Portugal | 15895 (15028, 16494) | 12990 (11277, 14224) | -18.28 (-25.86, -11.22) | 156.8 (148.25, 162.71) | 121.96 (105.87, 133.54) | -22.22 (-29.44, -15.51) | 127.07 (119.07, 132.39) | 45.77 (40.39, 49.87) | -63.98 (-66.56, -61.34) | -3.53 (-3.7, -3.36) | -4.59 (-4.98, -4.21) |
| Puerto Rico | 5370 (5031, 5607) | 4851 (3800, 5934) | -9.66 (-27.17, 10.73) | 148.62 (139.24, 155.18) | 137.76 (107.91, 168.51) | -7.31 (-25.27, 13.62) | 156.43 (145.25, 163.87) | 59.58 (46.61, 73.44) | -61.91 (-69.56, -52.78) | -3.27 (-3.75, -2.78) | -3.49 (-3.95, -3.02) |
| Qatar | 274 (226, 327) | 830 (631, 1068) | 203.32 (126.08, 308.55) | 61.47 (50.73, 73.42) | 28.98 (22.01, 37.27) | -52.87 (-64.87, -36.51) | 405.98 (334.4, 473.78) | 252.99 (205.75, 305.43) | -37.68 (-50.16, -20.84) | -1.91 (-2.21, -1.61) | -3.69 (-4.28, -3.09) |
| Republic of Korea | 31169 (29041, 32641) | 28281 (23998, 32635) | -9.27 (-21.22, 7.94) | 70.3 (65.5, 73.62) | 52.96 (44.94, 61.12) | -24.66 (-34.59, -10.38) | 147.6 (132.83, 156.44) | 35.02 (29.52, 40.61) | -76.27 (-79.21, -72.04) | -4.91 (-5.27, -4.55) | -6.06 (-6.23, -5.88) |
| Republic of Moldova | 13926 (13345, 14283) | 15367 (13477, 17212) | 10.35 (-1.6, 22.95) | 313.2 (300.12, 321.23) | 416.64 (365.41, 466.69) | 33.03 (18.62, 48.23) | 411.62 (386.22, 424.51) | 265.43 (232.66, 297.19) | -35.52 (-42.51, -28.32) | -1.42 (-1.91, -0.93) | -1.35 (-1.9, -0.8) |
| Romania | 67761 (64558, 71301) | 69806 (59148, 81377) | 3.02 (-12.02, 19.05) | 289.62 (275.93, 304.75) | 362.87 (307.47, 423.02) | 25.29 (7.01, 44.8) | 301.4 (282.38, 318.78) | 177.15 (150.34, 206.81) | -41.23 (-49.9, -32.07) | -1.91 (-2.26, -1.56) | -2.63 (-2.98, -2.28) |
| Russian Federation | 491368 (471212, 503313) | 562609 (488817, 632969) | 14.5 (1.56, 27.74) | 325.36 (312.01, 333.27) | 383.46 (333.17, 431.42) | 17.86 (4.54, 31.49) | 315.49 (298.72, 324.61) | 240.6 (208.85, 270.82) | -23.74 (-32.08, -15.05) | -0.94 (-1.64, -0.23) | -1.82 (-2.19, -1.45) |
| Rwanda | 2587 (2028, 3316) | 3770 (2755, 4987) | 45.72 (-1.24, 93.78) | 36.07 (28.27, 46.23) | 29.71 (21.71, 39.31) | -17.62 (-44.17, 9.55) | 112.82 (88.76, 144.37) | 84.93 (61.74, 111.53) | -24.72 (-47.99, -2.71) | -1.07 (-1.18, -0.95) | -2.43 (-2.68, -2.17) |
| Saint Kitts and Nevis | 86 (80, 92) | 55 (48, 63) | -35.94 (-44.63, -25.22) | 208.32 (194.52, 222.02) | 92.73 (81.01, 105.68) | -55.48 (-61.53, -48.04) | 245.83 (230.05, 260.69) | 104.07 (91.83, 117.32) | -57.67 (-62.66, -51.18) | -2.68 (-3.19, -2.16) | -3.83 (-6.69, -0.88) |
| Saint Lucia | 118 (110, 125) | 134 (116, 154) | 13.12 (-1.84, 29.23) | 86.05 (80.25, 90.9) | 76.59 (66.2, 88.06) | -10.99 (-22.76, 1.68) | 158.58 (147.63, 167.42) | 66.59 (57.61, 76.35) | -58.01 (-63.3, -52.12) | -2.95 (-3.37, -2.53) | -3.21 (-5.33, -1.04) |
| Saint Vincent and the Grenadines | 126 (117, 135) | 159 (142, 178) | 26.06 (11.93, 41.67) | 114.93 (106.7, 122.42) | 140.94 (125.1, 157.55) | 22.63 (8.88, 37.81) | 192.4 (178.13, 204.55) | 132.27 (118.25, 147.2) | -31.25 (-38.61, -22.97) | -1.42 (-1.75, -1.1) | -1.91 (-3.74, -0.05) |
| Samoa | 168 (141, 202) | 286 (238, 347) | 69.95 (34.18, 118.27) | 102.83 (86.2, 123.75) | 135.3 (112.71, 164.16) | 31.58 (3.88, 68.99) | 216.98 (184.04, 258.02) | 215.98 (181.67, 258.97) | -0.46 (-20.4, 24.96) | -0.03 (-0.06, 0) | 0.09 (-0.91, 1.11) |
| San Marino | 25 (21, 29) | 37 (26, 50) | 49.03 (2.6, 103.51) | 105.5 (90.1, 121.72) | 111.89 (77.87, 149.81) | 6.06 (-26.98, 44.83) | 77.62 (66.13, 89.39) | 46.19 (31.95, 63.41) | -40.5 (-59.7, -17.33) | -1.8 (-1.93, -1.66) | -1.36 (-9.1, 7.03) |
| Sao Tome and Principe | 61 (52, 70) | 125 (101, 144) | 103.95 (65.97, 150.5) | 50.34 (42.65, 57.99) | 60.75 (49.27, 70.25) | 20.69 (-1.79, 48.23) | 119.19 (103.15, 135.47) | 153.15 (124.47, 176.56) | 28.5 (6.72, 53.5) | 0.9 (0.77, 1.03) | 0.49 (-1.37, 2.4) |
| Saudi Arabia | 11757 (9413, 14194) | 29689 (24089, 36176) | 152.51 (89.47, 235.81) | 73.28 (58.66, 88.46) | 83.09 (67.42, 101.24) | 13.39 (-14.92, 50.79) | 241.31 (196.57, 285.82) | 205.6 (172.88, 238.97) | -14.8 (-33.08, 9.61) | -0.63 (-0.75, -0.5) | -0.13 (-0.28, 0.02) |
| Senegal | 3404 (2813, 3930) | 7131 (5690, 8749) | 109.5 (67.06, 164.46) | 44.66 (36.91, 51.57) | 47.12 (37.6, 57.81) | 5.49 (-15.88, 33.17) | 131.73 (110.64, 151.17) | 117.63 (95.84, 141.94) | -10.71 (-26.84, 9.04) | -0.44 (-0.8, -0.08) | -0.44 (-0.67, -0.21) |
| Serbia | 24060 (22059, 25759) | 28365 (23576, 33585) | 17.9 (-3.12, 41.7) | 256.02 (234.73, 274.1) | 324.29 (269.54, 383.97) | 26.67 (4.08, 52.24) | 254.25 (232.1, 271.15) | 204.39 (171.27, 238.34) | -19.61 (-33.07, -4.73) | -0.86 (-1.14, -0.57) | -2.53 (-3.12, -1.93) |
| Seychelles | 84 (77, 91) | 108 (96, 120) | 28.74 (13.83, 44.17) | 114.52 (105.22, 124.98) | 105.41 (93.68, 117.71) | -7.96 (-18.62, 3.07) | 150.85 (138.57, 164.49) | 110.19 (97.6, 122.93) | -26.96 (-34.94, -18.63) | -1.12 (-1.47, -0.76) | -1.65 (-3.4, 0.14) |
| Sierra Leone | 2394 (1882, 2975) | 3920 (2947, 5057) | 63.73 (31, 108.95) | 65.56 (51.52, 81.47) | 47.32 (35.57, 61.04) | -27.83 (-42.25, -7.89) | 145.8 (114.73, 179.25) | 134.01 (103.83, 170.09) | -8.09 (-25.25, 14.69) | -0.26 (-0.36, -0.17) | 0.23 (-0.07, 0.53) |
| Singapore | 2845 (2717, 2932) | 3833 (3363, 4135) | 34.73 (22.28, 44.33) | 93.37 (89.16, 96.21) | 67.64 (59.33, 72.97) | -27.56 (-34.25, -22.4) | 150.93 (141.55, 156.3) | 52.34 (45.61, 56.67) | -65.32 (-68.21, -63.01) | -3.5 (-3.81, -3.18) | -3.82 (-4.34, -3.3) |
| Slovakia | 20064 (18860, 20794) | 17777 (14344, 21446) | -11.4 (-28.36, 6.28) | 379.8 (357, 393.61) | 326.96 (263.81, 394.44) | -13.91 (-30.39, 3.26) | 358.53 (334.41, 372.48) | 198.91 (160.25, 239.67) | -44.52 (-55.09, -33.61) | -1.9 (-2.31, -1.48) | -3.28 (-3.69, -2.86) |
| Slovenia | 3248 (2539, 4186) | 2980 (2326, 3848) | -8.26 (-33.29, 22.21) | 164.78 (128.83, 212.38) | 143.65 (112.14, 185.52) | -12.82 (-36.61, 16.13) | 142.5 (111.66, 183.17) | 59.04 (46.2, 76.19) | -58.57 (-70.01, -44.5) | -3.28 (-3.58, -2.97) | -4.45 (-5.61, -3.27) |
| Solomon Islands | 522 (408, 654) | 1243 (993, 1504) | 138.02 (80.08, 212.93) | 153.36 (119.84, 192.05) | 189.54 (151.4, 229.34) | 23.6 (-6.49, 62.49) | 404.14 (325.92, 489.95) | 430.35 (358.68, 501.14) | 6.49 (-15.93, 33.98) | 0.21 (0.17, 0.25) | 0.24 (-0.3, 0.79) |
| Somalia | 2292 (1741, 2957) | 6390 (4726, 8608) | 178.8 (95.37, 285.2) | 32.07 (24.35, 41.38) | 31.41 (23.23, 42.31) | -2.04 (-31.36, 35.34) | 116.2 (90.63, 144.35) | 124.6 (94.03, 166.56) | 7.22 (-21.7, 43.17) | 0.28 (0.2, 0.36) | 0.59 (0.38, 0.8) |
| South Africa | 15078 (13235, 16649) | 30470 (27596, 32798) | 102.09 (84.95, 121.68) | 40.94 (35.94, 45.2) | 54.81 (49.64, 59) | 33.89 (22.54, 46.87) | 80.43 (70.45, 89.11) | 81.39 (73.45, 87.67) | 1.19 (-7.3, 11.05) | 0.22 (-0.43, 0.87) | -1.19 (-1.48, -0.89) |
| South Sudan | 1721 (1329, 2130) | 2491 (1744, 3378) | 44.78 (2.97, 100.83) | 29.37 (22.69, 36.36) | 26.84 (18.78, 36.39) | -8.61 (-35, 26.76) | 89.56 (70.79, 109.57) | 84.24 (60.14, 111.51) | -5.94 (-31.08, 25.59) | -0.23 (-0.31, -0.15) | -0.07 (-0.39, 0.25) |
| Spain | 57651 (52930, 60545) | 53632 (46434, 59832) | -6.97 (-13.8, 1.28) | 148.66 (136.48, 156.12) | 116.54 (100.9, 130.01) | -21.61 (-27.36, -14.65) | 110.9 (100.91, 116.72) | 44.96 (40.03, 49.47) | -59.46 (-61.6, -56.48) | -3.04 (-3.25, -2.84) | -3.61 (-3.95, -3.27) |
| Sri Lanka | 15475 (14232, 16744) | 23969 (17942, 30757) | 54.89 (13.52, 99.43) | 89.86 (82.64, 97.23) | 109.67 (82.1, 140.73) | 22.05 (-10.55, 57.15) | 174.65 (159.54, 189.44) | 109.05 (81.57, 138.94) | -37.56 (-53.72, -20.04) | -1.15 (-1.34, -0.96) | -2.11 (-2.31, -1.91) |
| Sudan | 29384 (23723, 35303) | 43187 (33613, 54936) | 46.98 (18, 82.98) | 145.47 (117.45, 174.78) | 105.83 (82.37, 134.62) | -27.25 (-41.59, -9.43) | 361.78 (292.57, 428.25) | 271.47 (214.92, 338.38) | -24.96 (-38.27, -9.04) | -1.04 (-1.11, -0.97) | -1.55 (-1.61, -1.48) |
| Suriname | 441 (404, 467) | 608 (509, 710) | 37.64 (15.34, 61.88) | 114.17 (104.41, 120.73) | 105.5 (88.47, 123.23) | -7.6 (-22.57, 8.68) | 180.65 (165.72, 190.97) | 106.9 (89.91, 124.63) | -40.82 (-50.3, -30.61) | -2.2 (-3.88, -0.5) | -2.07 (-2.86, -1.28) |
| Sweden | 31270 (28956, 32515) | 18975 (16561, 21085) | -39.32 (-43.24, -33.15) | 364.07 (337.13, 378.57) | 185.62 (162.01, 206.26) | -49.02 (-52.31, -43.84) | 189.78 (175.84, 197.22) | 73.66 (65.51, 81.2) | -61.19 (-63.26, -57.6) | -3.23 (-3.37, -3.09) | -4.05 (-4.53, -3.56) |
| Switzerland | 15155 (14012, 15858) | 12371 (10380, 14076) | -18.37 (-26.22, -9.46) | 220.75 (204.09, 230.98) | 140.98 (118.28, 160.41) | -36.14 (-42.28, -29.16) | 136.87 (126.68, 143.08) | 55.72 (47.74, 62.59) | -59.29 (-62.49, -55.27) | -3.06 (-3.26, -2.86) | -4.64 (-5.13, -4.15) |
| Syrian Arab Republic | 18022 (14948, 21346) | 33542 (26239, 43170) | 86.11 (39.94, 152.57) | 139.76 (115.92, 165.54) | 231.46 (181.07, 297.9) | 65.61 (24.53, 124.74) | 387.72 (323.52, 455.31) | 359.72 (288.25, 449.75) | -7.22 (-28.38, 22.88) | -0.34 (-0.58, -0.1) | -1.32 (-1.45, -1.19) |
| Taiwan (Province of China) | 10016 (9518, 10486) | 16672 (13460, 20667) | 66.45 (35.28, 106.04) | 49.1 (46.66, 51.4) | 70.58 (56.99, 87.5) | 43.75 (16.83, 77.94) | 84.41 (78.36, 88.87) | 41.16 (33.2, 51.29) | -51.24 (-60.55, -39.64) | -2.45 (-2.68, -2.23) | -1.86 (-2.1, -1.63) |
| Tajikistan | 6437 (5790, 7126) | 13513 (11366, 16248) | 109.92 (73.13, 156.95) | 119.75 (107.71, 132.55) | 142.35 (119.74, 171.17) | 18.88 (-1.95, 45.51) | 241.17 (217.19, 266.68) | 439.18 (376.64, 515.79) | 82.1 (53.66, 116.8) | 2.07 (1.19, 2.96) | 0.52 (0.13, 0.9) |
| Thailand | 25730 (22859, 28841) | 51595 (39019, 65636) | 100.53 (49.1, 159.18) | 45.24 (40.19, 50.71) | 73.59 (55.65, 93.62) | 62.67 (20.96, 110.25) | 92.42 (81.07, 103.49) | 52.62 (39.87, 66.89) | -43.07 (-57.29, -26.52) | -2 (-2.43, -1.57) | -2.38 (-2.57, -2.18) |
| Timor-Leste | 226 (182, 285) | 1021 (769, 1273) | 351.56 (238.08, 483.57) | 28.87 (23.23, 36.41) | 76.48 (57.61, 95.35) | 164.87 (98.31, 242.31) | 108.1 (88.81, 133.18) | 156.94 (121.02, 192.15) | 45.18 (12.01, 80.45) | 1.34 (1.21, 1.47) | 1.43 (0.77, 2.1) |
| Togo | 1344 (1130, 1589) | 3709 (2959, 4713) | 176.07 (123.02, 252.7) | 36.68 (30.84, 43.37) | 46.82 (37.36, 59.5) | 27.66 (3.13, 63.09) | 142.57 (121.09, 165.27) | 134.88 (111.77, 166.54) | -5.4 (-21.55, 17.51) | -0.2 (-0.3, -0.11) | -0.01 (-0.3, 0.29) |
| Tokelau | 2 (2, 3) | 2 (2, 3) | -6.53 (-28.29, 21.52) | 146.39 (118.66, 181.59) | 163.64 (133.79, 202.59) | 11.78 (-14.24, 45.33) | 192.74 (156.06, 238.67) | 188.65 (154.74, 232.17) | -2.12 (-24.76, 26.63) | -0.09 (-0.11, -0.07) | -0.03 (-11.48, 12.91) |
| Tonga | 64 (54, 73) | 100 (81, 121) | 56.7 (23.46, 99.39) | 66.15 (56.14, 75.83) | 98.02 (79.58, 118.46) | 48.18 (16.75, 88.56) | 131.47 (112.36, 150.72) | 130.16 (106.02, 156.84) | -1 (-21.39, 24.9) | 0.18 (-0.44, 0.8) | 0.07 (-1.77, 1.95) |
| Trinidad and Tobago | 1692 (1618, 1771) | 2143 (1658, 2723) | 26.66 (-1.52, 61.35) | 140.66 (134.51, 147.18) | 154.48 (119.48, 196.26) | 9.82 (-14.61, 39.9) | 227.91 (215.52, 239.49) | 120.07 (93.49, 152.14) | -47.32 (-58.9, -32.98) | -2.19 (-2.83, -1.56) | -2.9 (-3.45, -2.35) |
| Tunisia | 9462 (8283, 10856) | 21457 (16189, 27281) | 126.78 (68.72, 196.98) | 112.11 (98.15, 128.64) | 185.43 (139.91, 235.75) | 65.39 (23.05, 116.59) | 238.79 (208.11, 273.08) | 193.45 (146.86, 244.04) | -18.99 (-38.51, 4.94) | -0.78 (-0.87, -0.7) | -1.25 (-1.4, -1.1) |
| Turkey | 74197 (64620, 82837) | 99046 (80454, 120867) | 33.49 (6.05, 67.24) | 124.13 (108.11, 138.59) | 121.74 (98.89, 148.56) | -1.93 (-22.09, 22.87) | 229.86 (199.98, 255.18) | 120.96 (97.99, 147.2) | -47.38 (-58.12, -34.73) | -2 (-2.37, -1.62) | -3.63 (-3.99, -3.27) |
| Turkmenistan | 6247 (5925, 6462) | 11305 (9343, 13757) | 80.98 (49.45, 118.87) | 168.6 (159.92, 174.39) | 222.41 (183.8, 270.64) | 31.92 (8.94, 59.54) | 403.25 (377.12, 418.24) | 357.47 (298.53, 428.96) | -11.35 (-25.94, 5.97) | -1.1 (-1.89, -0.3) | -1.65 (-1.84, -1.46) |
| Tuvalu | 15 (12, 18) | 23 (18, 29) | 53.14 (17.12, 106.83) | 158.39 (128.15, 194.33) | 192.15 (153.64, 247.34) | 21.31 (-7.22, 63.84) | 247.15 (200.64, 303.28) | 251.53 (201.8, 317.74) | 1.77 (-21.87, 36.01) | 0.07 (0.01, 0.13) | 0.25 (-3.29, 3.91) |
| Uganda | 4171 (3147, 5228) | 9590 (6633, 12167) | 129.93 (81.74, 188.65) | 24.09 (18.17, 30.19) | 23.32 (16.13, 29.59) | -3.17 (-23.46, 21.56) | 83.9 (63.19, 105.02) | 87.29 (59.51, 109.67) | 4.04 (-16.14, 29.85) | 0.11 (0.05, 0.18) | -0.11 (-0.27, 0.06) |
| Ukraine | 216870 (206523, 223116) | 325874 (284911, 371596) | 50.26 (32.95, 70.78) | 411.8 (392.16, 423.67) | 739.91 (646.9, 843.72) | 79.67 (58.97, 104.2) | 341.2 (320.7, 352.72) | 424.23 (369.92, 483.93) | 24.33 (10.1, 40.43) | 0.42 (-0.09, 0.92) | 0.72 (0.37, 1.08) |
| United Arab Emirates | 838 (667, 1073) | 4880 (3504, 6755) | 482.44 (302.29, 721.68) | 44.76 (35.61, 57.3) | 52.81 (37.91, 73.09) | 17.98 (-18.51, 66.45) | 297.53 (251.6, 366.43) | 175.4 (134.75, 223.39) | -41.05 (-54.12, -24.8) | -1.94 (-2.39, -1.49) | -1.53 (-1.9, -1.16) |
| United Kingdom | 183219 (173134, 188221) | 93398 (84321, 98290) | -49.02 (-51.66, -46.26) | 318.79 (301.24, 327.49) | 138.94 (125.44, 146.22) | -56.42 (-58.67, -54.05) | 197.98 (186.8, 203.59) | 66.8 (60.86, 70.03) | -66.26 (-67.68, -64.58) | -3.82 (-4, -3.63) | -4.05 (-4.45, -3.65) |
| United Republic of Tanzania | 7282 (6003, 8851) | 18652 (13646, 23772) | 156.14 (95.02, 235.22) | 28.11 (23.18, 34.17) | 32.88 (24.05, 41.9) | 16.93 (-10.97, 53.04) | 88.88 (73.09, 107.92) | 95.36 (70.75, 119.6) | 7.3 (-16.1, 39) | 0.2 (0.08, 0.32) | 0.06 (-0.07, 0.2) |
| United States Virgin Islands | 140 (122, 158) | 296 (263, 326) | 111.16 (80.96, 147.88) | 132.15 (115.27, 149.14) | 284.5 (252.9, 313.77) | 115.28 (84.49, 152.71) | 205.24 (179.36, 229.44) | 174.5 (155.35, 192.7) | -14.98 (-26.25, -1.7) | -0.52 (-0.73, -0.32) | -1.1 (-3.2, 1.05) |
| United States of America | 604095 (558113, 627316) | 557649 (496860, 594411) | -7.69 (-11.14, -3.43) | 238.2 (220.07, 247.36) | 170.03 (151.49, 181.23) | -28.62 (-31.29, -25.33) | 181.57 (168.1, 188.38) | 91.03 (82.44, 96.54) | -49.86 (-51.39, -47.6) | -2.38 (-2.5, -2.27) | -2.32 (-2.48, -2.15) |
| Uruguay | 5904 (5522, 6144) | 4091 (3664, 4374) | -30.71 (-34.96, -26.61) | 188.07 (175.89, 195.71) | 119.06 (106.64, 127.28) | -36.7 (-40.58, -32.95) | 155.02 (144.33, 161.63) | 67.02 (60.9, 71.32) | -56.77 (-58.98, -54.36) | -2.86 (-3.04, -2.68) | -2.97 (-3.49, -2.45) |
| Uzbekistan | 32098 (29838, 33413) | 75501 (65706, 86057) | 135.22 (103.22, 170.45) | 153.23 (142.44, 159.51) | 224.19 (195.11, 255.53) | 46.31 (26.41, 68.23) | 323.05 (297.5, 337.43) | 707.51 (638.23, 780.68) | 119.01 (96.32, 143.33) | 2.6 (2.05, 3.15) | 1.98 (1.8, 2.17) |
| Vanuatu | 150 (114, 195) | 460 (358, 605) | 207.33 (133.93, 311.46) | 98.9 (75.6, 128.97) | 156.22 (121.67, 205.48) | 57.95 (20.23, 111.47) | 263.3 (208.02, 335.02) | 304.91 (243.14, 388.28) | 15.81 (-9.13, 50.66) | 0.52 (0.3, 0.74) | 0.42 (-0.4, 1.24) |
| Venezuela (Bolivarian Republic of) | 15042 (14132, 15707) | 36194 (28430, 46067) | 140.62 (90.59, 203.76) | 79.88 (75.05, 83.41) | 128.95 (101.29, 164.12) | 61.42 (27.86, 103.77) | 174.42 (161.95, 182.65) | 130.01 (102.56, 164.86) | -25.46 (-41.11, -6.4) | -1.31 (-2.3, -0.31) | -1.37 (-1.55, -1.19) |
| Viet Nam | 36842 (30303, 43520) | 74640 (61450, 88926) | 102.59 (54.85, 157.7) | 54.23 (44.6, 64.06) | 77.45 (63.76, 92.27) | 42.83 (9.17, 81.67) | 103.93 (85.32, 122.44) | 95.63 (79.48, 112.13) | -7.99 (-28.56, 15.6) | -0.3 (-0.36, -0.24) | -0.72 (-0.81, -0.64) |
| Yemen | 14521 (11429, 18316) | 32305 (26020, 42118) | 122.47 (75.47, 194.46) | 105.77 (83.24, 133.41) | 102.55 (82.59, 133.7) | -3.05 (-23.53, 28.33) | 353.65 (286.77, 434.72) | 294.76 (243.73, 374.52) | -16.65 (-32.34, 5.21) | -0.67 (-0.79, -0.54) | -1.05 (-1.14, -0.95) |
| Zambia | 2470 (2057, 2953) | 4833 (3824, 6056) | 95.67 (42.89, 159.7) | 31.1 (25.89, 37.18) | 26.5 (20.97, 33.21) | -14.78 (-37.77, 13.11) | 104.69 (87.69, 124.07) | 90.1 (72.33, 111.29) | -13.94 (-36.28, 11.58) | -0.7 (-0.89, -0.5) | -1.04 (-1.24, -0.84) |
| Zimbabwe | 4090 (3615, 4590) | 8779 (7037, 10756) | 114.65 (69.05, 169.31) | 39.56 (34.97, 44.4) | 58.48 (46.88, 71.65) | 47.83 (16.43, 85.47) | 132.66 (117.21, 147.5) | 162.28 (131.51, 197.63) | 22.33 (-2.68, 52.9) | 0.69 (0.49, 0.89) | 1.51 (1.28, 1.75) |

Parentheses for all GBD health estimate indicate 95% uncertainty intervals; parentheses for net drift indicate 95% confidence intervals.

Abbreviations: AAMR, all-age mortality rate; ASMR, age-standardized mortality rate; AAPC, average annual percentage change; SDI, socio-demographic index.

**Table S2. The p value of age-period-cohort test for IHD mortality across 5 socio-demographic index (SDI) quintiles, 21 GBD regions, and 204 countries and territories.**

| **Location** | **Gender** | **All Age Deviations = 0** | **All Period Deviations = 0** | **All Cohort Deviations = 0** | **All Period RR = 1** | **All Cohort RR = 1** | **All Local Drifts = Net Drift** | **Net Drift = 0** |
| --- | --- | --- | --- | --- | --- | --- | --- | --- |
| Global | Both | < 0.001 | 0.024 | < 0.001 | < 0.001 | < 0.001 | < 0.001 | < 0.001 |
| Global | Female | < 0.001 | < 0.001 | < 0.001 | < 0.001 | < 0.001 | < 0.001 | < 0.001 |
| Global | Male | < 0.001 | 0.716 | < 0.001 | < 0.001 | < 0.001 | < 0.001 | < 0.001 |
| High SDI | Both | < 0.001 | < 0.001 | < 0.001 | < 0.001 | < 0.001 | < 0.001 | < 0.001 |
| High SDI | Female | < 0.001 | < 0.001 | < 0.001 | < 0.001 | < 0.001 | < 0.001 | < 0.001 |
| High SDI | Male | < 0.001 | < 0.001 | < 0.001 | < 0.001 | < 0.001 | < 0.001 | < 0.001 |
| High-middle SDI | Both | < 0.001 | < 0.001 | < 0.001 | < 0.001 | < 0.001 | < 0.001 | < 0.001 |
| High-middle SDI | Female | < 0.001 | < 0.001 | < 0.001 | < 0.001 | < 0.001 | < 0.001 | < 0.001 |
| High-middle SDI | Male | < 0.001 | < 0.001 | < 0.001 | < 0.001 | < 0.001 | < 0.001 | < 0.001 |
| Middle SDI | Both | < 0.001 | < 0.001 | < 0.001 | < 0.001 | < 0.001 | < 0.001 | < 0.001 |
| Middle SDI | Female | < 0.001 | < 0.001 | < 0.001 | < 0.001 | < 0.001 | < 0.001 | < 0.001 |
| Middle SDI | Male | < 0.001 | < 0.001 | < 0.001 | < 0.001 | < 0.001 | < 0.001 | 0.274 |
| Low-middle SDI | Both | < 0.001 | 0.016 | < 0.001 | < 0.001 | < 0.001 | < 0.001 | < 0.001 |
| Low-middle SDI | Female | < 0.001 | < 0.001 | 0.004 | < 0.001 | < 0.001 | < 0.001 | < 0.001 |
| Low-middle SDI | Male | < 0.001 | < 0.001 | 0.004 | < 0.001 | 0.005 | 0.003 | 0.56 |
| Low SDI | Both | < 0.001 | < 0.001 | < 0.001 | < 0.001 | < 0.001 | < 0.001 | < 0.001 |
| Low SDI | Female | < 0.001 | 0.002 | < 0.001 | < 0.001 | < 0.001 | < 0.001 | < 0.001 |
| Low SDI | Male | < 0.001 | < 0.001 | < 0.001 | < 0.001 | < 0.001 | < 0.001 | < 0.001 |
| High-income Asia Pacific | Both | < 0.001 | < 0.001 | < 0.001 | < 0.001 | < 0.001 | < 0.001 | < 0.001 |
| High-income Asia Pacific | Female | < 0.001 | < 0.001 | < 0.001 | < 0.001 | < 0.001 | < 0.001 | < 0.001 |
| High-income Asia Pacific | Male | < 0.001 | < 0.001 | < 0.001 | < 0.001 | < 0.001 | < 0.001 | < 0.001 |
| High-income North America | Both | < 0.001 | < 0.001 | < 0.001 | < 0.001 | < 0.001 | < 0.001 | < 0.001 |
| High-income North America | Female | < 0.001 | < 0.001 | < 0.001 | < 0.001 | < 0.001 | < 0.001 | < 0.001 |
| High-income North America | Male | < 0.001 | < 0.001 | < 0.001 | < 0.001 | < 0.001 | < 0.001 | < 0.001 |
| Western Europe | Both | < 0.001 | < 0.001 | < 0.001 | < 0.001 | < 0.001 | < 0.001 | < 0.001 |
| Western Europe | Female | < 0.001 | < 0.001 | < 0.001 | < 0.001 | < 0.001 | < 0.001 | < 0.001 |
| Western Europe | Male | < 0.001 | < 0.001 | < 0.001 | < 0.001 | < 0.001 | < 0.001 | < 0.001 |
| Australasia | Both | < 0.001 | < 0.001 | < 0.001 | < 0.001 | < 0.001 | < 0.001 | < 0.001 |
| Australasia | Female | < 0.001 | < 0.001 | < 0.001 | < 0.001 | < 0.001 | < 0.001 | < 0.001 |
| Australasia | Male | < 0.001 | < 0.001 | < 0.001 | < 0.001 | < 0.001 | < 0.001 | < 0.001 |
| Andean Latin America | Both | < 0.001 | < 0.001 | < 0.001 | < 0.001 | < 0.001 | < 0.001 | < 0.001 |
| Andean Latin America | Female | < 0.001 | < 0.001 | < 0.001 | < 0.001 | < 0.001 | < 0.001 | < 0.001 |
| Andean Latin America | Male | < 0.001 | < 0.001 | < 0.001 | < 0.001 | < 0.001 | < 0.001 | < 0.001 |
| Tropical Latin America | Both | < 0.001 | < 0.001 | < 0.001 | < 0.001 | < 0.001 | < 0.001 | < 0.001 |
| Tropical Latin America | Female | < 0.001 | < 0.001 | < 0.001 | < 0.001 | < 0.001 | < 0.001 | < 0.001 |
| Tropical Latin America | Male | < 0.001 | < 0.001 | < 0.001 | < 0.001 | < 0.001 | < 0.001 | < 0.001 |
| Central Latin America | Both | < 0.001 | < 0.001 | < 0.001 | < 0.001 | < 0.001 | < 0.001 | < 0.001 |
| Central Latin America | Female | < 0.001 | < 0.001 | < 0.001 | < 0.001 | < 0.001 | < 0.001 | < 0.001 |
| Central Latin America | Male | < 0.001 | < 0.001 | < 0.001 | < 0.001 | < 0.001 | < 0.001 | < 0.001 |
| Southern Latin America | Both | < 0.001 | < 0.001 | 0.018 | < 0.001 | < 0.001 | 0.091 | < 0.001 |
| Southern Latin America | Female | < 0.001 | < 0.001 | < 0.001 | < 0.001 | < 0.001 | < 0.001 | < 0.001 |
| Southern Latin America | Male | < 0.001 | < 0.001 | 0.092 | < 0.001 | < 0.001 | 0.352 | < 0.001 |
| Caribbean | Both | < 0.001 | < 0.001 | < 0.001 | < 0.001 | < 0.001 | < 0.001 | < 0.001 |
| Caribbean | Female | < 0.001 | < 0.001 | < 0.001 | < 0.001 | < 0.001 | < 0.001 | < 0.001 |
| Caribbean | Male | < 0.001 | < 0.001 | < 0.001 | < 0.001 | < 0.001 | < 0.001 | < 0.001 |
| Central Europe | Both | < 0.001 | < 0.001 | < 0.001 | < 0.001 | < 0.001 | < 0.001 | < 0.001 |
| Central Europe | Female | < 0.001 | < 0.001 | < 0.001 | < 0.001 | < 0.001 | < 0.001 | < 0.001 |
| Central Europe | Male | < 0.001 | < 0.001 | < 0.001 | < 0.001 | < 0.001 | < 0.001 | < 0.001 |
| Eastern Europe | Both | < 0.001 | < 0.001 | < 0.001 | < 0.001 | < 0.001 | < 0.001 | < 0.001 |
| Eastern Europe | Female | < 0.001 | < 0.001 | < 0.001 | < 0.001 | < 0.001 | < 0.001 | < 0.001 |
| Eastern Europe | Male | < 0.001 | < 0.001 | < 0.001 | < 0.001 | < 0.001 | < 0.001 | < 0.001 |
| Central Asia | Both | < 0.001 | < 0.001 | < 0.001 | < 0.001 | < 0.001 | < 0.001 | < 0.001 |
| Central Asia | Female | < 0.001 | < 0.001 | < 0.001 | < 0.001 | < 0.001 | < 0.001 | < 0.001 |
| Central Asia | Male | < 0.001 | < 0.001 | < 0.001 | < 0.001 | < 0.001 | < 0.001 | < 0.001 |
| North Africa and Middle East | Both | < 0.001 | < 0.001 | < 0.001 | < 0.001 | < 0.001 | < 0.001 | < 0.001 |
| North Africa and Middle East | Female | < 0.001 | < 0.001 | < 0.001 | < 0.001 | < 0.001 | < 0.001 | < 0.001 |
| North Africa and Middle East | Male | < 0.001 | < 0.001 | < 0.001 | < 0.001 | < 0.001 | < 0.001 | < 0.001 |
| South Asia | Both | < 0.001 | 0.129 | < 0.001 | < 0.001 | < 0.001 | < 0.001 | < 0.001 |
| South Asia | Female | < 0.001 | 0.036 | 0.005 | < 0.001 | < 0.001 | 0.001 | < 0.001 |
| South Asia | Male | < 0.001 | < 0.001 | < 0.001 | < 0.001 | < 0.001 | < 0.001 | 0.134 |
| Southeast Asia | Both | < 0.001 | < 0.001 | < 0.001 | < 0.001 | < 0.001 | < 0.001 | < 0.001 |
| Southeast Asia | Female | < 0.001 | < 0.001 | < 0.001 | < 0.001 | < 0.001 | < 0.001 | < 0.001 |
| Southeast Asia | Male | < 0.001 | < 0.001 | < 0.001 | < 0.001 | < 0.001 | < 0.001 | < 0.001 |
| East Asia | Both | < 0.001 | < 0.001 | < 0.001 | < 0.001 | < 0.001 | < 0.001 | 0.135 |
| East Asia | Female | < 0.001 | < 0.001 | < 0.001 | < 0.001 | < 0.001 | < 0.001 | < 0.001 |
| East Asia | Male | < 0.001 | < 0.001 | < 0.001 | < 0.001 | < 0.001 | < 0.001 | < 0.001 |
| Oceania | Both | < 0.001 | 0.053 | 0.974 | < 0.001 | 0.016 | 0.943 | < 0.001 |
| Oceania | Female | < 0.001 | 0.349 | 1 | 0.001 | 0.016 | 1 | < 0.001 |
| Oceania | Male | < 0.001 | 0.219 | 1 | 0.137 | 0.989 | 1 | 0.048 |
| Western Sub-Saharan Africa | Both | < 0.001 | < 0.001 | < 0.001 | < 0.001 | < 0.001 | < 0.001 | < 0.001 |
| Western Sub-Saharan Africa | Female | < 0.001 | < 0.001 | < 0.001 | < 0.001 | < 0.001 | < 0.001 | < 0.001 |
| Western Sub-Saharan Africa | Male | < 0.001 | < 0.001 | < 0.001 | < 0.001 | < 0.001 | < 0.001 | < 0.001 |
| Eastern Sub-Saharan Africa | Both | < 0.001 | 0.284 | < 0.001 | < 0.001 | < 0.001 | < 0.001 | < 0.001 |
| Eastern Sub-Saharan Africa | Female | < 0.001 | 0.174 | < 0.001 | < 0.001 | < 0.001 | < 0.001 | < 0.001 |
| Eastern Sub-Saharan Africa | Male | < 0.001 | 0.142 | < 0.001 | < 0.001 | < 0.001 | < 0.001 | < 0.001 |
| Central Sub-Saharan Africa | Both | < 0.001 | 0.335 | < 0.001 | < 0.001 | < 0.001 | < 0.001 | < 0.001 |
| Central Sub-Saharan Africa | Female | < 0.001 | < 0.001 | 0.402 | < 0.001 | < 0.001 | 0.238 | < 0.001 |
| Central Sub-Saharan Africa | Male | < 0.001 | 0.061 | < 0.001 | < 0.001 | < 0.001 | < 0.001 | < 0.001 |
| Southern Sub-Saharan Africa | Both | < 0.001 | < 0.001 | < 0.001 | < 0.001 | < 0.001 | < 0.001 | < 0.001 |
| Southern Sub-Saharan Africa | Female | < 0.001 | < 0.001 | < 0.001 | < 0.001 | < 0.001 | < 0.001 | 0.008 |
| Southern Sub-Saharan Africa | Male | < 0.001 | < 0.001 | < 0.001 | < 0.001 | < 0.001 | < 0.001 | < 0.001 |
| Afghanistan | Both | < 0.001 | < 0.001 | < 0.001 | < 0.001 | < 0.001 | < 0.001 | < 0.001 |
| Afghanistan | Female | < 0.001 | < 0.001 | < 0.001 | < 0.001 | < 0.001 | < 0.001 | < 0.001 |
| Afghanistan | Male | < 0.001 | < 0.001 | < 0.001 | < 0.001 | < 0.001 | < 0.001 | < 0.001 |
| Albania | Both | < 0.001 | 0.02 | < 0.001 | 0.04 | < 0.001 | < 0.001 | 0.874 |
| Albania | Female | < 0.001 | 0.002 | 0.916 | 0.003 | 0.6 | 0.818 | 0.657 |
| Albania | Male | 0.402 | 0.022 | 0.072 | 0.044 | < 0.001 | 0.04 | 0.762 |
| Algeria | Both | < 0.001 | 0.007 | 0.255 | < 0.001 | < 0.001 | 0.158 | < 0.001 |
| Algeria | Female | < 0.001 | 0.037 | 0.251 | < 0.001 | < 0.001 | 0.16 | < 0.001 |
| Algeria | Male | < 0.001 | < 0.001 | 0.309 | < 0.001 | < 0.001 | 0.209 | < 0.001 |
| American Samoa | Both | 0.966 | 1 | 1 | 1 | 1 | 1 | 0.925 |
| American Samoa | Female | 1 | 1 | 1 | 0.999 | 1 | 1 | 0.641 |
| American Samoa | Male | 0.97 | 1 | 1 | 1 | 1 | 1 | 0.928 |
| Andorra | Both | 1 | 0.924 | 1 | 0.897 | 0.986 | 1 | 0.358 |
| Andorra | Female | 1 | 0.948 | 1 | 0.973 | 1 | 1 | 0.661 |
| Andorra | Male | 1 | 0.992 | 1 | 0.978 | 0.999 | 1 | 0.454 |
| Angola | Both | < 0.001 | 0.74 | < 0.001 | < 0.001 | < 0.001 | < 0.001 | < 0.001 |
| Angola | Female | < 0.001 | 0.046 | 0.089 | < 0.001 | < 0.001 | 0.035 | < 0.001 |
| Angola | Male | < 0.001 | 0.711 | 0.025 | < 0.001 | < 0.001 | 0.008 | < 0.001 |
| Antigua and Barbuda | Both | 1 | 0.951 | 1 | 0.633 | 0.949 | 1 | 0.088 |
| Antigua and Barbuda | Female | 1 | 0.995 | 1 | 0.973 | 1 | 1 | 0.413 |
| Antigua and Barbuda | Male | 1 | 0.909 | 1 | 0.649 | 0.983 | 1 | 0.115 |
| Argentina | Both | < 0.001 | < 0.001 | < 0.001 | < 0.001 | < 0.001 | < 0.001 | < 0.001 |
| Argentina | Female | < 0.001 | < 0.001 | < 0.001 | < 0.001 | < 0.001 | < 0.001 | < 0.001 |
| Argentina | Male | < 0.001 | < 0.001 | < 0.001 | < 0.001 | < 0.001 | < 0.001 | < 0.001 |
| Armenia | Both | < 0.001 | < 0.001 | < 0.001 | < 0.001 | < 0.001 | < 0.001 | < 0.001 |
| Armenia | Female | < 0.001 | < 0.001 | 0.001 | < 0.001 | < 0.001 | < 0.001 | < 0.001 |
| Armenia | Male | < 0.001 | 0.219 | < 0.001 | < 0.001 | < 0.001 | < 0.001 | < 0.001 |
| Australia | Both | < 0.001 | < 0.001 | < 0.001 | < 0.001 | < 0.001 | < 0.001 | < 0.001 |
| Australia | Female | < 0.001 | < 0.001 | < 0.001 | < 0.001 | < 0.001 | < 0.001 | < 0.001 |
| Australia | Male | < 0.001 | < 0.001 | < 0.001 | < 0.001 | < 0.001 | < 0.001 | < 0.001 |
| Austria | Both | < 0.001 | < 0.001 | < 0.001 | < 0.001 | < 0.001 | < 0.001 | < 0.001 |
| Austria | Female | < 0.001 | < 0.001 | < 0.001 | < 0.001 | < 0.001 | < 0.001 | < 0.001 |
| Austria | Male | < 0.001 | < 0.001 | < 0.001 | < 0.001 | < 0.001 | < 0.001 | < 0.001 |
| Azerbaijan | Both | 0.029 | 0.003 | < 0.001 | < 0.001 | < 0.001 | < 0.001 | < 0.001 |
| Azerbaijan | Female | < 0.001 | < 0.001 | < 0.001 | < 0.001 | < 0.001 | < 0.001 | < 0.001 |
| Azerbaijan | Male | < 0.001 | < 0.001 | < 0.001 | < 0.001 | < 0.001 | < 0.001 | < 0.001 |
| Bahamas | Both | 0.142 | 0.597 | 1 | 0.064 | 0.057 | 1 | 0.003 |
| Bahamas | Female | 0.999 | 0.802 | 1 | 0.433 | 0.875 | 1 | 0.06 |
| Bahamas | Male | 0.088 | 0.881 | 1 | 0.266 | 0.43 | 1 | 0.016 |
| Bahrain | Both | 0.957 | 0.042 | 0.001 | < 0.001 | < 0.001 | < 0.001 | < 0.001 |
| Bahrain | Female | 0.117 | 0.485 | 0.324 | < 0.001 | < 0.001 | 0.182 | < 0.001 |
| Bahrain | Male | 0.998 | 0.108 | 0.224 | < 0.001 | < 0.001 | 0.134 | < 0.001 |
| Bangladesh | Both | < 0.001 | < 0.001 | < 0.001 | < 0.001 | < 0.001 | < 0.001 | 0.513 |
| Bangladesh | Female | < 0.001 | < 0.001 | < 0.001 | 0.001 | < 0.001 | < 0.001 | 0.96 |
| Bangladesh | Male | < 0.001 | < 0.001 | 0.291 | < 0.001 | 0.002 | 0.164 | 0.102 |
| Barbados | Both | 0.794 | 0.192 | 1 | 0.009 | < 0.001 | 1 | 0.001 |
| Barbados | Female | 0.995 | 0.662 | 1 | 0.49 | 0.085 | 1 | 0.129 |
| Barbados | Male | 0.712 | 0.432 | 1 | 0.035 | < 0.001 | 1 | 0.003 |
| Belarus | Both | < 0.001 | < 0.001 | < 0.001 | < 0.001 | < 0.001 | < 0.001 | 0.011 |
| Belarus | Female | < 0.001 | < 0.001 | 0.152 | < 0.001 | < 0.001 | 0.092 | 0.022 |
| Belarus | Male | < 0.001 | < 0.001 | < 0.001 | < 0.001 | < 0.001 | < 0.001 | 0.004 |
| Belgium | Both | < 0.001 | < 0.001 | < 0.001 | < 0.001 | < 0.001 | < 0.001 | < 0.001 |
| Belgium | Female | < 0.001 | < 0.001 | < 0.001 | < 0.001 | < 0.001 | < 0.001 | < 0.001 |
| Belgium | Male | < 0.001 | < 0.001 | < 0.001 | < 0.001 | < 0.001 | < 0.001 | < 0.001 |
| Belize | Both | 0.7 | 0.159 | 1 | 0.001 | 0.017 | 1 | < 0.001 |
| Belize | Female | 1 | 0.831 | 1 | 0.205 | 0.502 | 1 | 0.024 |
| Belize | Male | 0.829 | 0.23 | 1 | 0.026 | 0.661 | 1 | 0.015 |
| Benin | Both | < 0.001 | 0.563 | 1 | 0.068 | 0.626 | 1 | 0.004 |
| Benin | Female | < 0.001 | 0.741 | 1 | 0.852 | 1 | 1 | 0.726 |
| Benin | Male | < 0.001 | 0.837 | 1 | 0.012 | 0.198 | 1 | < 0.001 |
| Bermuda | Both | 0.995 | 0.223 | 1 | 0.055 | < 0.001 | 1 | 0.02 |
| Bermuda | Female | 1 | 0.574 | 1 | 0.457 | < 0.001 | 1 | 0.175 |
| Bermuda | Male | 0.999 | 0.456 | 1 | 0.222 | 0.003 | 1 | 0.062 |
| Bhutan | Both | 0.847 | 1 | 0.95 | 0.984 | 0.964 | 0.864 | 0.42 |
| Bhutan | Female | 1 | 0.992 | 0.992 | 0.603 | 0.989 | 0.968 | 0.061 |
| Bhutan | Male | 0.913 | 0.993 | 1 | 0.997 | 1 | 1 | 0.775 |
| Bolivia (Plurinational State of) | Both | < 0.001 | < 0.001 | < 0.001 | < 0.001 | < 0.001 | < 0.001 | < 0.001 |
| Bolivia (Plurinational State of) | Female | < 0.001 | < 0.001 | < 0.001 | < 0.001 | < 0.001 | < 0.001 | < 0.001 |
| Bolivia (Plurinational State of) | Male | < 0.001 | < 0.001 | 0.815 | < 0.001 | < 0.001 | 0.651 | < 0.001 |
| Bosnia and Herzegovina | Both | < 0.001 | < 0.001 | < 0.001 | < 0.001 | < 0.001 | < 0.001 | < 0.001 |
| Bosnia and Herzegovina | Female | < 0.001 | < 0.001 | < 0.001 | < 0.001 | < 0.001 | < 0.001 | < 0.001 |
| Bosnia and Herzegovina | Male | < 0.001 | < 0.001 | < 0.001 | < 0.001 | < 0.001 | < 0.001 | < 0.001 |
| Botswana | Both | < 0.001 | < 0.001 | 0.939 | < 0.001 | 0.901 | 0.843 | 0.042 |
| Botswana | Female | 0.017 | 0.427 | 1 | 0.266 | 1 | 1 | 0.055 |
| Botswana | Male | < 0.001 | < 0.001 | 0.951 | < 0.001 | 0.474 | 0.867 | 0.002 |
| Brazil | Both | < 0.001 | < 0.001 | < 0.001 | < 0.001 | < 0.001 | < 0.001 | < 0.001 |
| Brazil | Female | < 0.001 | < 0.001 | < 0.001 | < 0.001 | < 0.001 | < 0.001 | < 0.001 |
| Brazil | Male | < 0.001 | < 0.001 | < 0.001 | < 0.001 | < 0.001 | < 0.001 | < 0.001 |
| Brunei Darussalam | Both | 0.997 | 0.7 | 0.996 | 0.005 | 0.085 | 0.986 | < 0.001 |
| Brunei Darussalam | Female | 0.959 | 0.997 | 1 | 0.288 | 0.917 | 1 | 0.015 |
| Brunei Darussalam | Male | 1 | 0.387 | 0.983 | 0.115 | 0.532 | 0.958 | 0.022 |
| Bulgaria | Both | < 0.001 | < 0.001 | < 0.001 | < 0.001 | < 0.001 | < 0.001 | < 0.001 |
| Bulgaria | Female | < 0.001 | < 0.001 | < 0.001 | < 0.001 | < 0.001 | < 0.001 | < 0.001 |
| Bulgaria | Male | < 0.001 | < 0.001 | < 0.001 | < 0.001 | < 0.001 | < 0.001 | < 0.001 |
| Burkina Faso | Both | < 0.001 | < 0.001 | 0.175 | < 0.001 | < 0.001 | 0.107 | < 0.001 |
| Burkina Faso | Female | < 0.001 | < 0.001 | 0.392 | < 0.001 | < 0.001 | 0.265 | < 0.001 |
| Burkina Faso | Male | < 0.001 | 0.053 | 0.99 | < 0.001 | < 0.001 | 0.967 | < 0.001 |
| Burundi | Both | < 0.001 | 0.793 | 0.085 | < 0.001 | < 0.001 | 0.034 | < 0.001 |
| Burundi | Female | < 0.001 | 0.542 | 0.978 | 0.003 | 0.525 | 0.929 | < 0.001 |
| Burundi | Male | < 0.001 | 0.213 | 0.181 | < 0.001 | < 0.001 | 0.083 | < 0.001 |
| Cabo Verde | Both | 0.23 | 0.002 | 1 | 0.005 | 1 | 1 | 0.201 |
| Cabo Verde | Female | 0.52 | 0.049 | 1 | 0.089 | 0.999 | 1 | 0.551 |
| Cabo Verde | Male | 0.59 | 0.173 | 1 | 0.139 | 0.963 | 1 | 0.071 |
| Cambodia | Both | < 0.001 | < 0.001 | < 0.001 | < 0.001 | < 0.001 | < 0.001 | < 0.001 |
| Cambodia | Female | 0.275 | < 0.001 | < 0.001 | < 0.001 | < 0.001 | < 0.001 | < 0.001 |
| Cambodia | Male | < 0.001 | 0.039 | < 0.001 | < 0.001 | < 0.001 | < 0.001 | < 0.001 |
| Cameroon | Both | < 0.001 | < 0.001 | 0.808 | < 0.001 | < 0.001 | 0.661 | < 0.001 |
| Cameroon | Female | < 0.001 | < 0.001 | 0.999 | < 0.001 | 0.008 | 0.996 | 0.002 |
| Cameroon | Male | < 0.001 | < 0.001 | 0.961 | < 0.001 | < 0.001 | 0.896 | < 0.001 |
| Canada | Both | < 0.001 | < 0.001 | < 0.001 | < 0.001 | < 0.001 | < 0.001 | < 0.001 |
| Canada | Female | < 0.001 | < 0.001 | < 0.001 | < 0.001 | < 0.001 | < 0.001 | < 0.001 |
| Canada | Male | < 0.001 | < 0.001 | < 0.001 | < 0.001 | < 0.001 | < 0.001 | < 0.001 |
| Central African Republic | Both | < 0.001 | 0.658 | 1 | 0.786 | 1 | 1 | 0.773 |
| Central African Republic | Female | < 0.001 | 0.991 | 1 | 0.884 | 1 | 1 | 0.245 |
| Central African Republic | Male | < 0.001 | 0.692 | 1 | 0.621 | 0.98 | 1 | 0.216 |
| Chad | Both | < 0.001 | 0.161 | 1 | 0.085 | 0.848 | 1 | 0.024 |
| Chad | Female | < 0.001 | 0.841 | 1 | 0.245 | 0.179 | 1 | 0.021 |
| Chad | Male | < 0.001 | 0.03 | 1 | 0.056 | 1 | 1 | 0.715 |
| Chile | Both | < 0.001 | < 0.001 | < 0.001 | < 0.001 | < 0.001 | < 0.001 | < 0.001 |
| Chile | Female | < 0.001 | < 0.001 | < 0.001 | < 0.001 | < 0.001 | < 0.001 | < 0.001 |
| Chile | Male | < 0.001 | 0.758 | < 0.001 | < 0.001 | < 0.001 | < 0.001 | < 0.001 |
| China | Both | < 0.001 | < 0.001 | < 0.001 | < 0.001 | < 0.001 | < 0.001 | 0.109 |
| China | Female | < 0.001 | < 0.001 | < 0.001 | < 0.001 | < 0.001 | < 0.001 | < 0.001 |
| China | Male | < 0.001 | < 0.001 | < 0.001 | < 0.001 | < 0.001 | < 0.001 | < 0.001 |
| Colombia | Both | < 0.001 | < 0.001 | < 0.001 | < 0.001 | < 0.001 | < 0.001 | < 0.001 |
| Colombia | Female | < 0.001 | < 0.001 | < 0.001 | < 0.001 | < 0.001 | < 0.001 | < 0.001 |
| Colombia | Male | < 0.001 | < 0.001 | < 0.001 | < 0.001 | < 0.001 | < 0.001 | < 0.001 |
| Comoros | Both | 0.535 | 0.942 | 1 | 0.958 | 1 | 1 | 0.496 |
| Comoros | Female | 0.863 | 0.999 | 1 | 0.999 | 1 | 1 | 0.77 |
| Comoros | Male | 0.772 | 0.831 | 1 | 0.773 | 1 | 1 | 0.228 |
| Congo | Both | < 0.001 | 0.026 | 0.01 | < 0.001 | < 0.001 | 0.003 | < 0.001 |
| Congo | Female | < 0.001 | 0.799 | 1 | 0.255 | 0.992 | 0.999 | 0.039 |
| Congo | Male | < 0.001 | < 0.001 | 0.187 | < 0.001 | < 0.001 | 0.086 | < 0.001 |
| Cook Islands | Both | 1 | 0.993 | 1 | 0.996 | 1 | 1 | 0.681 |
| Cook Islands | Female | 1 | 0.996 | 1 | 0.998 | 1 | 1 | 0.773 |
| Cook Islands | Male | 1 | 0.999 | 1 | 1 | 1 | 1 | 0.806 |
| Costa Rica | Both | < 0.001 | < 0.001 | 0.167 | < 0.001 | < 0.001 | 0.144 | < 0.001 |
| Costa Rica | Female | 0.023 | < 0.001 | 0.903 | < 0.001 | < 0.001 | 0.89 | < 0.001 |
| Costa Rica | Male | < 0.001 | < 0.001 | 0.359 | < 0.001 | < 0.001 | 0.239 | < 0.001 |
| Croatia | Both | < 0.001 | 0.179 | < 0.001 | < 0.001 | < 0.001 | < 0.001 | < 0.001 |
| Croatia | Female | < 0.001 | < 0.001 | < 0.001 | < 0.001 | < 0.001 | < 0.001 | < 0.001 |
| Croatia | Male | < 0.001 | 0.321 | < 0.001 | < 0.001 | < 0.001 | < 0.001 | < 0.001 |
| Cuba | Both | < 0.001 | < 0.001 | < 0.001 | < 0.001 | < 0.001 | < 0.001 | < 0.001 |
| Cuba | Female | < 0.001 | < 0.001 | < 0.001 | < 0.001 | < 0.001 | < 0.001 | < 0.001 |
| Cuba | Male | < 0.001 | < 0.001 | < 0.001 | < 0.001 | < 0.001 | < 0.001 | < 0.001 |
| Cyprus | Both | < 0.001 | 0.008 | 0.005 | < 0.001 | < 0.001 | 0.009 | < 0.001 |
| Cyprus | Female | < 0.001 | 0.105 | 0.646 | < 0.001 | < 0.001 | 0.739 | < 0.001 |
| Cyprus | Male | < 0.001 | 0.018 | 0.107 | < 0.001 | < 0.001 | 0.218 | < 0.001 |
| Czechia | Both | < 0.001 | < 0.001 | < 0.001 | < 0.001 | < 0.001 | < 0.001 | < 0.001 |
| Czechia | Female | < 0.001 | < 0.001 | < 0.001 | < 0.001 | < 0.001 | < 0.001 | < 0.001 |
| Czechia | Male | < 0.001 | < 0.001 | < 0.001 | < 0.001 | < 0.001 | < 0.001 | < 0.001 |
| Côte d'Ivoire | Both | < 0.001 | < 0.001 | 0.308 | < 0.001 | < 0.001 | 0.182 | < 0.001 |
| Côte d'Ivoire | Female | < 0.001 | 0.018 | 1 | 0.032 | 1 | 1 | 0.896 |
| Côte d'Ivoire | Male | < 0.001 | < 0.001 | 0.519 | < 0.001 | < 0.001 | 0.331 | < 0.001 |
| Democratic People's Republic of Korea | Both | < 0.001 | < 0.001 | 0.039 | < 0.001 | < 0.001 | 0.029 | < 0.001 |
| Democratic People's Republic of Korea | Female | < 0.001 | < 0.001 | 0.038 | < 0.001 | < 0.001 | 0.013 | < 0.001 |
| Democratic People's Republic of Korea | Male | < 0.001 | < 0.001 | 0.807 | < 0.001 | < 0.001 | 0.669 | < 0.001 |
| Democratic Republic of the Congo | Both | < 0.001 | 0.037 | 0.157 | < 0.001 | < 0.001 | 0.08 | < 0.001 |
| Democratic Republic of the Congo | Female | < 0.001 | < 0.001 | 0.996 | < 0.001 | < 0.001 | 0.983 | < 0.001 |
| Democratic Republic of the Congo | Male | < 0.001 | 0.19 | 0.666 | < 0.001 | < 0.001 | 0.478 | < 0.001 |
| Denmark | Both | < 0.001 | < 0.001 | < 0.001 | < 0.001 | < 0.001 | < 0.001 | < 0.001 |
| Denmark | Female | < 0.001 | < 0.001 | < 0.001 | < 0.001 | < 0.001 | < 0.001 | < 0.001 |
| Denmark | Male | < 0.001 | < 0.001 | < 0.001 | < 0.001 | < 0.001 | < 0.001 | < 0.001 |
| Djibouti | Both | 0.465 | 0.966 | 1 | 0.913 | 1 | 1 | 0.267 |
| Djibouti | Female | 0.954 | 0.994 | 1 | 0.969 | 1 | 1 | 0.358 |
| Djibouti | Male | 0.558 | 0.984 | 1 | 0.988 | 1 | 1 | 0.553 |
| Dominica | Both | 1 | 0.889 | 1 | 0.723 | 0.852 | 1 | 0.17 |
| Dominica | Female | 1 | 0.956 | 1 | 0.924 | 0.999 | 1 | 0.368 |
| Dominica | Male | 1 | 0.972 | 1 | 0.888 | 0.987 | 1 | 0.255 |
| Dominican Republic | Both | < 0.001 | < 0.001 | < 0.001 | < 0.001 | < 0.001 | < 0.001 | < 0.001 |
| Dominican Republic | Female | < 0.001 | < 0.001 | < 0.001 | < 0.001 | < 0.001 | < 0.001 | < 0.001 |
| Dominican Republic | Male | < 0.001 | 0.08 | 0.415 | < 0.001 | < 0.001 | 0.255 | < 0.001 |
| Ecuador | Both | < 0.001 | < 0.001 | < 0.001 | < 0.001 | < 0.001 | < 0.001 | < 0.001 |
| Ecuador | Female | < 0.001 | < 0.001 | < 0.001 | < 0.001 | < 0.001 | < 0.001 | < 0.001 |
| Ecuador | Male | < 0.001 | < 0.001 | < 0.001 | < 0.001 | < 0.001 | < 0.001 | < 0.001 |
| Egypt | Both | < 0.001 | 0.437 | < 0.001 | < 0.001 | < 0.001 | < 0.001 | < 0.001 |
| Egypt | Female | < 0.001 | < 0.001 | < 0.001 | < 0.001 | < 0.001 | < 0.001 | < 0.001 |
| Egypt | Male | < 0.001 | 0.165 | < 0.001 | < 0.001 | < 0.001 | < 0.001 | < 0.001 |
| El Salvador | Both | 0.001 | < 0.001 | < 0.001 | < 0.001 | < 0.001 | < 0.001 | < 0.001 |
| El Salvador | Female | < 0.001 | < 0.001 | < 0.001 | < 0.001 | < 0.001 | < 0.001 | < 0.001 |
| El Salvador | Male | 0.384 | < 0.001 | 0.004 | < 0.001 | < 0.001 | 0.001 | < 0.001 |
| Equatorial Guinea | Both | 0.952 | 0.011 | 0.218 | < 0.001 | < 0.001 | 0.105 | < 0.001 |
| Equatorial Guinea | Female | 0.818 | 0.757 | 0.993 | 0.128 | 0.63 | 0.971 | 0.006 |
| Equatorial Guinea | Male | 0.933 | 0.016 | 0.967 | < 0.001 | < 0.001 | 0.901 | < 0.001 |
| Eritrea | Both | < 0.001 | 0.95 | 0.929 | 0.448 | 0.723 | 0.825 | 0.036 |
| Eritrea | Female | 0.003 | 0.779 | 1 | 0.005 | 0.02 | 1 | < 0.001 |
| Eritrea | Male | < 0.001 | 0.801 | 1 | 0.794 | 0.979 | 0.998 | 0.492 |
| Estonia | Both | < 0.001 | < 0.001 | < 0.001 | < 0.001 | < 0.001 | < 0.001 | < 0.001 |
| Estonia | Female | 0.027 | < 0.001 | < 0.001 | < 0.001 | < 0.001 | < 0.001 | < 0.001 |
| Estonia | Male | < 0.001 | < 0.001 | < 0.001 | < 0.001 | < 0.001 | < 0.001 | < 0.001 |
| Eswatini | Both | < 0.001 | 0.002 | 1 | 0.002 | 0.997 | 1 | 0.032 |
| Eswatini | Female | 0.736 | 0.085 | 1 | 0.132 | 1 | 1 | 0.304 |
| Eswatini | Male | < 0.001 | 0.035 | 1 | 0.046 | 0.999 | 1 | 0.131 |
| Ethiopia | Both | < 0.001 | < 0.001 | < 0.001 | < 0.001 | < 0.001 | < 0.001 | < 0.001 |
| Ethiopia | Female | < 0.001 | < 0.001 | < 0.001 | < 0.001 | < 0.001 | < 0.001 | < 0.001 |
| Ethiopia | Male | < 0.001 | < 0.001 | < 0.001 | < 0.001 | < 0.001 | < 0.001 | < 0.001 |
| Fiji | Both | < 0.001 | 0.876 | 1 | 0.006 | 0.004 | 0.999 | < 0.001 |
| Fiji | Female | 0.015 | 0.994 | 1 | 0.729 | 0.997 | 1 | 0.106 |
| Fiji | Male | < 0.001 | 0.851 | 1 | 0.061 | 0.022 | 0.999 | 0.002 |
| Finland | Both | < 0.001 | < 0.001 | < 0.001 | < 0.001 | < 0.001 | < 0.001 | < 0.001 |
| Finland | Female | < 0.001 | < 0.001 | < 0.001 | < 0.001 | < 0.001 | < 0.001 | < 0.001 |
| Finland | Male | < 0.001 | < 0.001 | < 0.001 | < 0.001 | < 0.001 | < 0.001 | < 0.001 |
| France | Both | < 0.001 | < 0.001 | < 0.001 | < 0.001 | < 0.001 | < 0.001 | < 0.001 |
| France | Female | < 0.001 | < 0.001 | < 0.001 | < 0.001 | < 0.001 | < 0.001 | < 0.001 |
| France | Male | < 0.001 | < 0.001 | < 0.001 | < 0.001 | < 0.001 | < 0.001 | < 0.001 |
| Gabon | Both | < 0.001 | 0.906 | 0.999 | 0.053 | 0.8 | 0.994 | 0.003 |
| Gabon | Female | 0.003 | 0.469 | 1 | 0.238 | 1 | 1 | 0.12 |
| Gabon | Male | < 0.001 | 0.989 | 1 | 0.31 | 0.937 | 1 | 0.016 |
| Gambia | Both | < 0.001 | 0.774 | 1 | 0.493 | 0.998 | 1 | 0.134 |
| Gambia | Female | 0.007 | 0.912 | 1 | 0.477 | 0.972 | 1 | 0.078 |
| Gambia | Male | 0.013 | 0.914 | 1 | 0.931 | 1 | 1 | 0.531 |
| Georgia | Both | < 0.001 | < 0.001 | < 0.001 | < 0.001 | < 0.001 | < 0.001 | < 0.001 |
| Georgia | Female | < 0.001 | < 0.001 | < 0.001 | < 0.001 | < 0.001 | < 0.001 | < 0.001 |
| Georgia | Male | < 0.001 | < 0.001 | 0.017 | < 0.001 | < 0.001 | 0.013 | < 0.001 |
| Germany | Both | < 0.001 | < 0.001 | < 0.001 | < 0.001 | < 0.001 | < 0.001 | < 0.001 |
| Germany | Female | < 0.001 | < 0.001 | < 0.001 | < 0.001 | < 0.001 | < 0.001 | < 0.001 |
| Germany | Male | < 0.001 | < 0.001 | < 0.001 | < 0.001 | < 0.001 | < 0.001 | < 0.001 |
| Ghana | Both | < 0.001 | 0.066 | < 0.001 | 0.061 | < 0.001 | < 0.001 | 0.205 |
| Ghana | Female | < 0.001 | < 0.001 | < 0.001 | < 0.001 | < 0.001 | < 0.001 | < 0.001 |
| Ghana | Male | < 0.001 | < 0.001 | 0.734 | < 0.001 | 0.012 | 0.641 | 0.034 |
| Greece | Both | < 0.001 | < 0.001 | < 0.001 | < 0.001 | < 0.001 | < 0.001 | < 0.001 |
| Greece | Female | < 0.001 | 0.07 | < 0.001 | < 0.001 | < 0.001 | < 0.001 | < 0.001 |
| Greece | Male | < 0.001 | < 0.001 | < 0.001 | < 0.001 | < 0.001 | < 0.001 | < 0.001 |
| Greenland | Both | 0.951 | 0.968 | 1 | 0.767 | 0.207 | 1 | 0.153 |
| Greenland | Female | 1 | 0.894 | 1 | 0.835 | 0.648 | 1 | 0.318 |
| Greenland | Male | 0.97 | 0.983 | 1 | 0.93 | 0.97 | 1 | 0.319 |
| Grenada | Both | 0.892 | 0.69 | 1 | 0.299 | 0.418 | 1 | 0.041 |
| Grenada | Female | 1 | 0.381 | 1 | 0.363 | 0.988 | 1 | 0.219 |
| Grenada | Male | 0.994 | 0.982 | 1 | 0.718 | 0.903 | 1 | 0.117 |
| Guam | Both | 0.116 | 0.752 | 0.994 | 0.81 | 0.996 | 0.974 | 0.643 |
| Guam | Female | 0.881 | 0.562 | 1 | 0.692 | 1 | 0.999 | 0.867 |
| Guam | Male | 0.353 | 0.967 | 1 | 0.954 | 1 | 1 | 0.503 |
| Guatemala | Both | < 0.001 | < 0.001 | < 0.001 | < 0.001 | < 0.001 | < 0.001 | < 0.001 |
| Guatemala | Female | < 0.001 | < 0.001 | < 0.001 | < 0.001 | < 0.001 | < 0.001 | < 0.001 |
| Guatemala | Male | < 0.001 | < 0.001 | < 0.001 | < 0.001 | < 0.001 | < 0.001 | < 0.001 |
| Guinea | Both | < 0.001 | 0.001 | 0.59 | < 0.001 | < 0.001 | 0.394 | < 0.001 |
| Guinea | Female | < 0.001 | 0.011 | 1 | < 0.001 | < 0.001 | 0.999 | < 0.001 |
| Guinea | Male | < 0.001 | 0.012 | 0.82 | < 0.001 | < 0.001 | 0.655 | < 0.001 |
| Guinea-Bissau | Both | < 0.001 | 0.805 | 1 | 0.87 | 1 | 1 | 0.549 |
| Guinea-Bissau | Female | 0.005 | 0.915 | 1 | 0.788 | 0.999 | 1 | 0.188 |
| Guinea-Bissau | Male | < 0.001 | 0.925 | 1 | 0.958 | 1 | 1 | 0.776 |
| Guyana | Both | < 0.001 | 0.913 | 0.733 | < 0.001 | < 0.001 | 0.603 | < 0.001 |
| Guyana | Female | 0.009 | 0.999 | 0.986 | 0.035 | 0.004 | 0.971 | < 0.001 |
| Guyana | Male | < 0.001 | 0.764 | 0.984 | 0.004 | 0.012 | 0.953 | < 0.001 |
| Haiti | Both | < 0.001 | < 0.001 | 0.065 | < 0.001 | < 0.001 | 0.024 | < 0.001 |
| Haiti | Female | < 0.001 | 0.023 | 0.256 | < 0.001 | < 0.001 | 0.128 | < 0.001 |
| Haiti | Male | < 0.001 | < 0.001 | 0.999 | < 0.001 | < 0.001 | 0.994 | < 0.001 |
| Honduras | Both | < 0.001 | < 0.001 | < 0.001 | < 0.001 | < 0.001 | < 0.001 | < 0.001 |
| Honduras | Female | < 0.001 | < 0.001 | < 0.001 | < 0.001 | < 0.001 | < 0.001 | 0.02 |
| Honduras | Male | 0.263 | < 0.001 | < 0.001 | < 0.001 | < 0.001 | < 0.001 | 0.002 |
| Hungary | Both | < 0.001 | < 0.001 | < 0.001 | < 0.001 | < 0.001 | < 0.001 | < 0.001 |
| Hungary | Female | < 0.001 | < 0.001 | < 0.001 | < 0.001 | < 0.001 | < 0.001 | < 0.001 |
| Hungary | Male | < 0.001 | < 0.001 | < 0.001 | < 0.001 | < 0.001 | < 0.001 | < 0.001 |
| Iceland | Both | 0.932 | 0.514 | 0.029 | < 0.001 | < 0.001 | 0.01 | < 0.001 |
| Iceland | Female | 1 | 0.573 | 0.148 | 0.069 | < 0.001 | 0.073 | 0.006 |
| Iceland | Male | 0.968 | 0.723 | 0.379 | 0.007 | < 0.001 | 0.218 | < 0.001 |
| India | Both | < 0.001 | 0.056 | < 0.001 | < 0.001 | < 0.001 | < 0.001 | < 0.001 |
| India | Female | < 0.001 | 0.008 | 0.001 | < 0.001 | < 0.001 | < 0.001 | < 0.001 |
| India | Male | < 0.001 | < 0.001 | < 0.001 | < 0.001 | < 0.001 | < 0.001 | < 0.001 |
| Indonesia | Both | < 0.001 | < 0.001 | < 0.001 | < 0.001 | < 0.001 | < 0.001 | < 0.001 |
| Indonesia | Female | < 0.001 | < 0.001 | < 0.001 | < 0.001 | < 0.001 | < 0.001 | 0.002 |
| Indonesia | Male | < 0.001 | < 0.001 | < 0.001 | < 0.001 | < 0.001 | < 0.001 | < 0.001 |
| Iran (Islamic Republic of) | Both | < 0.001 | < 0.001 | < 0.001 | < 0.001 | < 0.001 | < 0.001 | < 0.001 |
| Iran (Islamic Republic of) | Female | < 0.001 | < 0.001 | < 0.001 | < 0.001 | < 0.001 | < 0.001 | < 0.001 |
| Iran (Islamic Republic of) | Male | < 0.001 | < 0.001 | < 0.001 | < 0.001 | < 0.001 | < 0.001 | < 0.001 |
| Iraq | Both | < 0.001 | < 0.001 | < 0.001 | < 0.001 | < 0.001 | < 0.001 | < 0.001 |
| Iraq | Female | < 0.001 | 0.147 | < 0.001 | < 0.001 | < 0.001 | < 0.001 | < 0.001 |
| Iraq | Male | < 0.001 | < 0.001 | < 0.001 | < 0.001 | < 0.001 | < 0.001 | < 0.001 |
| Ireland | Both | < 0.001 | < 0.001 | < 0.001 | < 0.001 | < 0.001 | < 0.001 | < 0.001 |
| Ireland | Female | 0.496 | < 0.001 | < 0.001 | < 0.001 | < 0.001 | < 0.001 | < 0.001 |
| Ireland | Male | < 0.001 | < 0.001 | < 0.001 | < 0.001 | < 0.001 | < 0.001 | < 0.001 |
| Israel | Both | < 0.001 | < 0.001 | < 0.001 | < 0.001 | < 0.001 | < 0.001 | < 0.001 |
| Israel | Female | < 0.001 | < 0.001 | < 0.001 | < 0.001 | < 0.001 | < 0.001 | < 0.001 |
| Israel | Male | < 0.001 | < 0.001 | < 0.001 | < 0.001 | < 0.001 | < 0.001 | < 0.001 |
| Italy | Both | < 0.001 | < 0.001 | < 0.001 | < 0.001 | < 0.001 | < 0.001 | < 0.001 |
| Italy | Female | < 0.001 | < 0.001 | < 0.001 | < 0.001 | < 0.001 | < 0.001 | < 0.001 |
| Italy | Male | < 0.001 | < 0.001 | < 0.001 | < 0.001 | < 0.001 | < 0.001 | < 0.001 |
| Jamaica | Both | < 0.001 | < 0.001 | 0.076 | < 0.001 | < 0.001 | 0.032 | 0.04 |
| Jamaica | Female | 0.331 | < 0.001 | 0.944 | < 0.001 | < 0.001 | 0.882 | 0.17 |
| Jamaica | Male | < 0.001 | 0.073 | 0.586 | 0.061 | 0.007 | 0.391 | 0.115 |
| Japan | Both | < 0.001 | < 0.001 | < 0.001 | < 0.001 | < 0.001 | < 0.001 | < 0.001 |
| Japan | Female | < 0.001 | < 0.001 | < 0.001 | < 0.001 | < 0.001 | < 0.001 | < 0.001 |
| Japan | Male | < 0.001 | < 0.001 | < 0.001 | < 0.001 | < 0.001 | < 0.001 | < 0.001 |
| Jordan | Both | < 0.001 | < 0.001 | 0.004 | < 0.001 | < 0.001 | 0.001 | < 0.001 |
| Jordan | Female | < 0.001 | < 0.001 | 0.01 | < 0.001 | < 0.001 | 0.003 | < 0.001 |
| Jordan | Male | < 0.001 | < 0.001 | 0.107 | < 0.001 | < 0.001 | 0.049 | < 0.001 |
| Kazakhstan | Both | < 0.001 | < 0.001 | < 0.001 | < 0.001 | < 0.001 | < 0.001 | < 0.001 |
| Kazakhstan | Female | < 0.001 | < 0.001 | < 0.001 | < 0.001 | < 0.001 | < 0.001 | < 0.001 |
| Kazakhstan | Male | < 0.001 | < 0.001 | < 0.001 | < 0.001 | < 0.001 | < 0.001 | < 0.001 |
| Kenya | Both | < 0.001 | < 0.001 | 0.006 | < 0.001 | < 0.001 | 0.002 | < 0.001 |
| Kenya | Female | < 0.001 | < 0.001 | 0.82 | < 0.001 | < 0.001 | 0.654 | < 0.001 |
| Kenya | Male | < 0.001 | < 0.001 | 0.02 | < 0.001 | < 0.001 | 0.006 | < 0.001 |
| Kiribati | Both | 0.002 | 0.991 | 1 | 0.991 | 1 | 1 | 0.592 |
| Kiribati | Female | 0.943 | 1 | 1 | 1 | 1 | 1 | 0.927 |
| Kiribati | Male | 0.001 | 0.997 | 1 | 0.996 | 1 | 1 | 0.619 |
| Kuwait | Both | < 0.001 | < 0.001 | 1 | < 0.001 | < 0.001 | 0.998 | < 0.001 |
| Kuwait | Female | 0.602 | 0.805 | 0.692 | < 0.001 | < 0.001 | 0.507 | < 0.001 |
| Kuwait | Male | < 0.001 | < 0.001 | 0.999 | < 0.001 | < 0.001 | 0.993 | < 0.001 |
| Kyrgyzstan | Both | 0.019 | < 0.001 | < 0.001 | < 0.001 | < 0.001 | < 0.001 | 0.011 |
| Kyrgyzstan | Female | 0.012 | < 0.001 | < 0.001 | < 0.001 | < 0.001 | < 0.001 | 0.072 |
| Kyrgyzstan | Male | 0.002 | < 0.001 | < 0.001 | < 0.001 | < 0.001 | < 0.001 | < 0.001 |
| Lao People's Democratic Republic | Both | < 0.001 | 0.597 | < 0.001 | < 0.001 | < 0.001 | < 0.001 | < 0.001 |
| Lao People's Democratic Republic | Female | 0.012 | 0.809 | 0.003 | < 0.001 | < 0.001 | < 0.001 | < 0.001 |
| Lao People's Democratic Republic | Male | < 0.001 | 0.756 | 0.271 | 0.012 | < 0.001 | 0.139 | < 0.001 |
| Latvia | Both | < 0.001 | < 0.001 | < 0.001 | < 0.001 | < 0.001 | < 0.001 | < 0.001 |
| Latvia | Female | < 0.001 | 0.009 | < 0.001 | < 0.001 | < 0.001 | < 0.001 | < 0.001 |
| Latvia | Male | < 0.001 | < 0.001 | < 0.001 | < 0.001 | < 0.001 | < 0.001 | < 0.001 |
| Lebanon | Both | < 0.001 | < 0.001 | < 0.001 | < 0.001 | < 0.001 | < 0.001 | < 0.001 |
| Lebanon | Female | < 0.001 | < 0.001 | 0.006 | < 0.001 | < 0.001 | 0.002 | < 0.001 |
| Lebanon | Male | < 0.001 | < 0.001 | 0.327 | < 0.001 | < 0.001 | 0.178 | < 0.001 |
| Lesotho | Both | < 0.001 | < 0.001 | 0.954 | < 0.001 | < 0.001 | 0.874 | < 0.001 |
| Lesotho | Female | < 0.001 | < 0.001 | 0.992 | < 0.001 | < 0.001 | 0.968 | < 0.001 |
| Lesotho | Male | < 0.001 | < 0.001 | 0.592 | < 0.001 | < 0.001 | 0.395 | < 0.001 |
| Liberia | Both | < 0.001 | 0.074 | 1 | 0.049 | 0.916 | 1 | 0.085 |
| Liberia | Female | < 0.001 | 0.395 | 1 | 0.508 | 1 | 1 | 0.598 |
| Liberia | Male | < 0.001 | 0.321 | 1 | 0.144 | 0.708 | 1 | 0.052 |
| Libya | Both | < 0.001 | < 0.001 | 0.073 | < 0.001 | < 0.001 | 0.045 | < 0.001 |
| Libya | Female | < 0.001 | < 0.001 | 0.988 | < 0.001 | 0.826 | 0.959 | 0.004 |
| Libya | Male | < 0.001 | < 0.001 | 0.11 | < 0.001 | 0.003 | 0.08 | < 0.001 |
| Lithuania | Both | < 0.001 | < 0.001 | < 0.001 | < 0.001 | < 0.001 | < 0.001 | < 0.001 |
| Lithuania | Female | < 0.001 | < 0.001 | < 0.001 | < 0.001 | < 0.001 | < 0.001 | < 0.001 |
| Lithuania | Male | < 0.001 | < 0.001 | < 0.001 | < 0.001 | < 0.001 | < 0.001 | < 0.001 |
| Luxembourg | Both | 0.713 | 0.874 | 0.306 | < 0.001 | < 0.001 | 0.163 | < 0.001 |
| Luxembourg | Female | 0.999 | 0.931 | 0.976 | 0.071 | < 0.001 | 0.924 | 0.002 |
| Luxembourg | Male | 0.915 | 0.963 | 0.572 | < 0.001 | < 0.001 | 0.375 | < 0.001 |
| Madagascar | Both | < 0.001 | < 0.001 | 0.302 | < 0.001 | 0.331 | 0.172 | 0.013 |
| Madagascar | Female | < 0.001 | 0.002 | 0.986 | 0.004 | 0.661 | 0.953 | 0.791 |
| Madagascar | Male | < 0.001 | 0.006 | 0.723 | < 0.001 | 0.497 | 0.557 | 0.002 |
| Malawi | Both | < 0.001 | < 0.001 | 0.826 | < 0.001 | < 0.001 | 0.664 | < 0.001 |
| Malawi | Female | < 0.001 | 0.502 | 0.02 | < 0.001 | < 0.001 | 0.006 | < 0.001 |
| Malawi | Male | < 0.001 | < 0.001 | 1 | < 0.001 | 1 | 0.999 | 0.626 |
| Malaysia | Both | < 0.001 | < 0.001 | < 0.001 | < 0.001 | < 0.001 | < 0.001 | 0.002 |
| Malaysia | Female | < 0.001 | < 0.001 | < 0.001 | < 0.001 | < 0.001 | < 0.001 | < 0.001 |
| Malaysia | Male | < 0.001 | < 0.001 | < 0.001 | < 0.001 | < 0.001 | < 0.001 | 0.031 |
| Maldives | Both | 0.873 | 0.294 | 0.813 | < 0.001 | < 0.001 | 0.658 | < 0.001 |
| Maldives | Female | 0.999 | 0.677 | 0.996 | < 0.001 | < 0.001 | 0.979 | < 0.001 |
| Maldives | Male | 0.94 | 0.654 | 0.986 | < 0.001 | < 0.001 | 0.953 | < 0.001 |
| Mali | Both | < 0.001 | 0.001 | 0.998 | < 0.001 | 0.107 | 0.99 | < 0.001 |
| Mali | Female | < 0.001 | 0.652 | 0.991 | 0.008 | 0.658 | 0.964 | < 0.001 |
| Mali | Male | < 0.001 | < 0.001 | 1 | < 0.001 | 0.825 | 1 | 0.191 |
| Malta | Both | 0.166 | 0.254 | 0.01 | < 0.001 | < 0.001 | 0.003 | < 0.001 |
| Malta | Female | 0.317 | 0.122 | 0.027 | 0.014 | < 0.001 | 0.01 | 0.006 |
| Malta | Male | 0.661 | 0.82 | 0.718 | 0.004 | < 0.001 | 0.58 | < 0.001 |
| Marshall Islands | Both | 0.703 | 0.994 | 1 | 0.996 | 1 | 1 | 0.655 |
| Marshall Islands | Female | 1 | 0.993 | 1 | 0.994 | 1 | 1 | 0.592 |
| Marshall Islands | Male | 0.748 | 0.998 | 1 | 1 | 1 | 1 | 0.91 |
| Mauritania | Both | < 0.001 | 0.004 | 0.845 | < 0.001 | < 0.001 | 0.69 | < 0.001 |
| Mauritania | Female | < 0.001 | 0.643 | 0.999 | 0.019 | 0.161 | 0.996 | < 0.001 |
| Mauritania | Male | 0.077 | 0.002 | 0.995 | < 0.001 | < 0.001 | 0.977 | < 0.001 |
| Mauritius | Both | < 0.001 | < 0.001 | < 0.001 | < 0.001 | < 0.001 | < 0.001 | < 0.001 |
| Mauritius | Female | 0.027 | < 0.001 | < 0.001 | < 0.001 | < 0.001 | < 0.001 | < 0.001 |
| Mauritius | Male | < 0.001 | < 0.001 | 0.006 | < 0.001 | < 0.001 | 0.003 | < 0.001 |
| Mexico | Both | < 0.001 | < 0.001 | < 0.001 | < 0.001 | < 0.001 | < 0.001 | 0.145 |
| Mexico | Female | < 0.001 | < 0.001 | < 0.001 | < 0.001 | < 0.001 | < 0.001 | < 0.001 |
| Mexico | Male | < 0.001 | < 0.001 | < 0.001 | < 0.001 | < 0.001 | < 0.001 | 0.002 |
| Micronesia (Federated States of) | Both | 0.044 | 0.997 | 1 | 0.998 | 1 | 1 | 0.73 |
| Micronesia (Federated States of) | Female | 0.998 | 1 | 1 | 0.999 | 1 | 1 | 0.731 |
| Micronesia (Federated States of) | Male | 0.068 | 1 | 1 | 1 | 1 | 1 | 0.882 |
| Monaco | Both | 1 | 0.688 | 1 | 0.722 | 0.378 | 1 | 0.411 |
| Monaco | Female | 1 | 0.933 | 1 | 0.966 | 0.998 | 1 | 0.71 |
| Monaco | Male | 1 | 0.856 | 1 | 0.869 | 0.661 | 1 | 0.446 |
| Mongolia | Both | < 0.001 | < 0.001 | < 0.001 | < 0.001 | < 0.001 | < 0.001 | < 0.001 |
| Mongolia | Female | < 0.001 | < 0.001 | < 0.001 | < 0.001 | < 0.001 | < 0.001 | < 0.001 |
| Mongolia | Male | < 0.001 | < 0.001 | 0.001 | < 0.001 | < 0.001 | < 0.001 | < 0.001 |
| Montenegro | Both | 0.857 | 0.452 | 0.022 | 0.107 | 0.019 | 0.013 | 0.028 |
| Montenegro | Female | 0.849 | 0.602 | 0.976 | 0.655 | 0.903 | 0.963 | 0.526 |
| Montenegro | Male | 0.666 | 0.594 | 0.175 | 0.163 | 0.216 | 0.094 | 0.027 |
| Morocco | Both | < 0.001 | < 0.001 | < 0.001 | < 0.001 | < 0.001 | < 0.001 | < 0.001 |
| Morocco | Female | < 0.001 | < 0.001 | < 0.001 | < 0.001 | < 0.001 | < 0.001 | < 0.001 |
| Morocco | Male | < 0.001 | < 0.001 | < 0.001 | < 0.001 | < 0.001 | < 0.001 | < 0.001 |
| Mozambique | Both | < 0.001 | < 0.001 | < 0.001 | < 0.001 | < 0.001 | < 0.001 | < 0.001 |
| Mozambique | Female | < 0.001 | < 0.001 | 1 | < 0.001 | < 0.001 | 0.999 | < 0.001 |
| Mozambique | Male | < 0.001 | < 0.001 | < 0.001 | < 0.001 | < 0.001 | < 0.001 | < 0.001 |
| Myanmar | Both | < 0.001 | < 0.001 | < 0.001 | < 0.001 | < 0.001 | < 0.001 | < 0.001 |
| Myanmar | Female | < 0.001 | < 0.001 | < 0.001 | < 0.001 | < 0.001 | < 0.001 | < 0.001 |
| Myanmar | Male | < 0.001 | 0.235 | < 0.001 | < 0.001 | < 0.001 | < 0.001 | < 0.001 |
| Namibia | Both | < 0.001 | 0.011 | 0.999 | < 0.001 | 0.797 | 0.995 | 0.011 |
| Namibia | Female | 0.071 | 0.13 | 0.996 | 0.02 | 0.679 | 0.981 | 0.018 |
| Namibia | Male | < 0.001 | 0.155 | 1 | 0.103 | 1 | 1 | 0.216 |
| Nauru | Both | 0.999 | 0.974 | 1 | 0.992 | 1 | 1 | 0.9 |
| Nauru | Female | 1 | 0.997 | 1 | 0.999 | 1 | 1 | 0.839 |
| Nauru | Male | 1 | 0.988 | 1 | 0.997 | 1 | 1 | 0.911 |
| Nepal | Both | < 0.001 | < 0.001 | < 0.001 | < 0.001 | < 0.001 | < 0.001 | 0.225 |
| Nepal | Female | < 0.001 | < 0.001 | < 0.001 | < 0.001 | < 0.001 | < 0.001 | < 0.001 |
| Nepal | Male | < 0.001 | < 0.001 | < 0.001 | < 0.001 | < 0.001 | < 0.001 | < 0.001 |
| Netherlands | Both | < 0.001 | < 0.001 | < 0.001 | < 0.001 | < 0.001 | < 0.001 | < 0.001 |
| Netherlands | Female | < 0.001 | < 0.001 | < 0.001 | < 0.001 | < 0.001 | < 0.001 | < 0.001 |
| Netherlands | Male | < 0.001 | < 0.001 | < 0.001 | < 0.001 | < 0.001 | < 0.001 | < 0.001 |
| New Zealand | Both | < 0.001 | < 0.001 | < 0.001 | < 0.001 | < 0.001 | < 0.001 | < 0.001 |
| New Zealand | Female | < 0.001 | < 0.001 | < 0.001 | < 0.001 | < 0.001 | < 0.001 | < 0.001 |
| New Zealand | Male | < 0.001 | < 0.001 | < 0.001 | < 0.001 | < 0.001 | < 0.001 | < 0.001 |
| Nicaragua | Both | < 0.001 | < 0.001 | < 0.001 | < 0.001 | < 0.001 | < 0.001 | 0.294 |
| Nicaragua | Female | < 0.001 | < 0.001 | < 0.001 | < 0.001 | < 0.001 | < 0.001 | 0.274 |
| Nicaragua | Male | < 0.001 | 0.827 | 0.385 | 0.887 | 0.339 | 0.227 | 0.838 |
| Niger | Both | < 0.001 | < 0.001 | 0.982 | < 0.001 | 0.021 | 0.941 | < 0.001 |
| Niger | Female | < 0.001 | 0.105 | 0.998 | 0.044 | 0.993 | 0.989 | 0.019 |
| Niger | Male | < 0.001 | < 0.001 | 1 | < 0.001 | 0.017 | 0.998 | 0.002 |
| Nigeria | Both | < 0.001 | < 0.001 | < 0.001 | < 0.001 | < 0.001 | < 0.001 | < 0.001 |
| Nigeria | Female | < 0.001 | < 0.001 | < 0.001 | < 0.001 | < 0.001 | < 0.001 | < 0.001 |
| Nigeria | Male | < 0.001 | < 0.001 | < 0.001 | < 0.001 | < 0.001 | < 0.001 | < 0.001 |
| Niue | Both | 1 | 1 | 1 | 1 | 1 | 1 | 0.901 |
| Niue | Female | 1 | 1 | 1 | 1 | 1 | 1 | 0.98 |
| Niue | Male | 1 | 1 | 1 | 1 | 1 | 1 | 0.902 |
| North Macedonia | Both | < 0.001 | < 0.001 | < 0.001 | < 0.001 | < 0.001 | < 0.001 | < 0.001 |
| North Macedonia | Female | < 0.001 | 0.184 | < 0.001 | < 0.001 | < 0.001 | < 0.001 | < 0.001 |
| North Macedonia | Male | < 0.001 | < 0.001 | < 0.001 | < 0.001 | < 0.001 | < 0.001 | < 0.001 |
| Northern Mariana Islands | Both | 0.987 | 0.998 | 1 | 0.994 | 1 | 1 | 0.603 |
| Northern Mariana Islands | Female | 1 | 1 | 1 | 1 | 1 | 1 | 0.926 |
| Northern Mariana Islands | Male | 0.996 | 0.998 | 1 | 0.987 | 1 | 1 | 0.496 |
| Norway | Both | < 0.001 | < 0.001 | < 0.001 | < 0.001 | < 0.001 | < 0.001 | < 0.001 |
| Norway | Female | 0.025 | < 0.001 | < 0.001 | < 0.001 | < 0.001 | < 0.001 | < 0.001 |
| Norway | Male | < 0.001 | < 0.001 | < 0.001 | < 0.001 | < 0.001 | < 0.001 | < 0.001 |
| Oman | Both | < 0.001 | 0.437 | < 0.001 | < 0.001 | < 0.001 | < 0.001 | < 0.001 |
| Oman | Female | < 0.001 | 0.743 | < 0.001 | < 0.001 | < 0.001 | < 0.001 | < 0.001 |
| Oman | Male | < 0.001 | 0.261 | < 0.001 | < 0.001 | < 0.001 | < 0.001 | < 0.001 |
| Pakistan | Both | < 0.001 | < 0.001 | < 0.001 | < 0.001 | < 0.001 | < 0.001 | < 0.001 |
| Pakistan | Female | < 0.001 | < 0.001 | < 0.001 | < 0.001 | < 0.001 | < 0.001 | < 0.001 |
| Pakistan | Male | < 0.001 | < 0.001 | < 0.001 | < 0.001 | < 0.001 | < 0.001 | < 0.001 |
| Palau | Both | 0.99 | 1 | 1 | 1 | 1 | 1 | 0.939 |
| Palau | Female | 1 | 1 | 1 | 1 | 1 | 1 | 0.946 |
| Palau | Male | 0.991 | 1 | 1 | 1 | 1 | 1 | 0.916 |
| Palestine | Both | < 0.001 | < 0.001 | 0.003 | < 0.001 | < 0.001 | < 0.001 | < 0.001 |
| Palestine | Female | < 0.001 | < 0.001 | 0.078 | < 0.001 | < 0.001 | 0.032 | < 0.001 |
| Palestine | Male | < 0.001 | 0.055 | 0.123 | < 0.001 | < 0.001 | 0.055 | < 0.001 |
| Panama | Both | < 0.001 | < 0.001 | 0.274 | < 0.001 | < 0.001 | 0.154 | < 0.001 |
| Panama | Female | 0.152 | < 0.001 | 0.44 | < 0.001 | < 0.001 | 0.297 | < 0.001 |
| Panama | Male | < 0.001 | 0.005 | 0.942 | < 0.001 | < 0.001 | 0.852 | < 0.001 |
| Papua New Guinea | Both | < 0.001 | < 0.001 | 1 | < 0.001 | < 0.001 | 1 | < 0.001 |
| Papua New Guinea | Female | < 0.001 | 0.033 | 1 | < 0.001 | < 0.001 | 1 | < 0.001 |
| Papua New Guinea | Male | < 0.001 | 0.012 | 1 | < 0.001 | < 0.001 | 1 | < 0.001 |
| Paraguay | Both | < 0.001 | < 0.001 | 0.416 | < 0.001 | < 0.001 | 0.591 | < 0.001 |
| Paraguay | Female | 0.311 | < 0.001 | 0.81 | < 0.001 | < 0.001 | 0.789 | < 0.001 |
| Paraguay | Male | < 0.001 | < 0.001 | 0.959 | < 0.001 | 0.883 | 0.986 | 0.213 |
| Peru | Both | < 0.001 | < 0.001 | < 0.001 | < 0.001 | < 0.001 | < 0.001 | < 0.001 |
| Peru | Female | < 0.001 | < 0.001 | < 0.001 | < 0.001 | < 0.001 | < 0.001 | < 0.001 |
| Peru | Male | < 0.001 | < 0.001 | < 0.001 | < 0.001 | < 0.001 | < 0.001 | < 0.001 |
| Philippines | Both | < 0.001 | < 0.001 | < 0.001 | < 0.001 | < 0.001 | < 0.001 | < 0.001 |
| Philippines | Female | < 0.001 | < 0.001 | < 0.001 | < 0.001 | < 0.001 | < 0.001 | < 0.001 |
| Philippines | Male | < 0.001 | < 0.001 | < 0.001 | < 0.001 | < 0.001 | < 0.001 | < 0.001 |
| Poland | Both | < 0.001 | < 0.001 | < 0.001 | < 0.001 | < 0.001 | < 0.001 | < 0.001 |
| Poland | Female | < 0.001 | < 0.001 | < 0.001 | < 0.001 | < 0.001 | < 0.001 | < 0.001 |
| Poland | Male | < 0.001 | < 0.001 | < 0.001 | < 0.001 | < 0.001 | < 0.001 | < 0.001 |
| Portugal | Both | < 0.001 | < 0.001 | < 0.001 | < 0.001 | < 0.001 | < 0.001 | < 0.001 |
| Portugal | Female | < 0.001 | < 0.001 | < 0.001 | < 0.001 | < 0.001 | < 0.001 | < 0.001 |
| Portugal | Male | < 0.001 | < 0.001 | < 0.001 | < 0.001 | < 0.001 | < 0.001 | < 0.001 |
| Puerto Rico | Both | < 0.001 | < 0.001 | 0.003 | < 0.001 | < 0.001 | 0.001 | < 0.001 |
| Puerto Rico | Female | < 0.001 | < 0.001 | 0.17 | < 0.001 | < 0.001 | 0.125 | < 0.001 |
| Puerto Rico | Male | < 0.001 | 0.017 | 0.245 | < 0.001 | < 0.001 | 0.148 | < 0.001 |
| Qatar | Both | 0.125 | 0.448 | < 0.001 | < 0.001 | < 0.001 | < 0.001 | < 0.001 |
| Qatar | Female | 0.92 | 0.912 | 0.001 | < 0.001 | < 0.001 | < 0.001 | < 0.001 |
| Qatar | Male | 0.057 | 0.405 | 0.107 | < 0.001 | < 0.001 | 0.055 | < 0.001 |
| Republic of Korea | Both | < 0.001 | < 0.001 | < 0.001 | < 0.001 | < 0.001 | < 0.001 | < 0.001 |
| Republic of Korea | Female | < 0.001 | < 0.001 | < 0.001 | < 0.001 | < 0.001 | < 0.001 | < 0.001 |
| Republic of Korea | Male | < 0.001 | < 0.001 | < 0.001 | < 0.001 | < 0.001 | < 0.001 | < 0.001 |
| Republic of Moldova | Both | < 0.001 | < 0.001 | < 0.001 | < 0.001 | < 0.001 | < 0.001 | < 0.001 |
| Republic of Moldova | Female | < 0.001 | < 0.001 | 0.699 | < 0.001 | < 0.001 | 0.763 | < 0.001 |
| Republic of Moldova | Male | < 0.001 | < 0.001 | < 0.001 | < 0.001 | < 0.001 | < 0.001 | < 0.001 |
| Romania | Both | < 0.001 | < 0.001 | < 0.001 | < 0.001 | < 0.001 | < 0.001 | < 0.001 |
| Romania | Female | < 0.001 | < 0.001 | < 0.001 | < 0.001 | < 0.001 | < 0.001 | < 0.001 |
| Romania | Male | < 0.001 | < 0.001 | < 0.001 | < 0.001 | < 0.001 | < 0.001 | < 0.001 |
| Russian Federation | Both | < 0.001 | < 0.001 | < 0.001 | < 0.001 | < 0.001 | < 0.001 | < 0.001 |
| Russian Federation | Female | < 0.001 | < 0.001 | < 0.001 | < 0.001 | < 0.001 | < 0.001 | < 0.001 |
| Russian Federation | Male | < 0.001 | < 0.001 | < 0.001 | < 0.001 | < 0.001 | < 0.001 | < 0.001 |
| Rwanda | Both | < 0.001 | < 0.001 | < 0.001 | < 0.001 | < 0.001 | < 0.001 | < 0.001 |
| Rwanda | Female | < 0.001 | < 0.001 | < 0.001 | < 0.001 | < 0.001 | < 0.001 | < 0.001 |
| Rwanda | Male | < 0.001 | < 0.001 | < 0.001 | < 0.001 | < 0.001 | < 0.001 | < 0.001 |
| Saint Kitts and Nevis | Both | 1 | 0.863 | 1 | 0.194 | 0.218 | 1 | 0.011 |
| Saint Kitts and Nevis | Female | 1 | 0.981 | 1 | 0.766 | 0.969 | 1 | 0.136 |
| Saint Kitts and Nevis | Male | 1 | 0.92 | 1 | 0.427 | 0.753 | 1 | 0.039 |
| Saint Lucia | Both | 0.931 | 0.246 | 1 | 0.028 | < 0.001 | 1 | 0.004 |
| Saint Lucia | Female | 0.996 | 0.479 | 1 | 0.292 | 0.028 | 1 | 0.078 |
| Saint Lucia | Male | 0.997 | 0.702 | 1 | 0.235 | 0.126 | 1 | 0.022 |
| Saint Vincent and the Grenadines | Both | 0.999 | 0.331 | 1 | 0.159 | 0.884 | 1 | 0.044 |
| Saint Vincent and the Grenadines | Female | 1 | 0.356 | 1 | 0.297 | 1 | 1 | 0.154 |
| Saint Vincent and the Grenadines | Male | 1 | 0.864 | 1 | 0.659 | 0.982 | 1 | 0.13 |
| Samoa | Both | 0.063 | 1 | 1 | 1 | 1 | 1 | 0.86 |
| Samoa | Female | 0.972 | 0.999 | 1 | 0.985 | 1 | 1 | 0.429 |
| Samoa | Male | 0.088 | 1 | 1 | 0.999 | 1 | 1 | 0.693 |
| San Marino | Both | 1 | 0.977 | 1 | 0.991 | 1 | 1 | 0.742 |
| San Marino | Female | 1 | 0.997 | 1 | 0.999 | 1 | 1 | 0.871 |
| San Marino | Male | 1 | 0.989 | 1 | 0.996 | 1 | 1 | 0.781 |
| Sao Tome and Principe | Both | 1 | 0.995 | 1 | 0.995 | 1 | 1 | 0.606 |
| Sao Tome and Principe | Female | 1 | 0.997 | 1 | 1 | 1 | 1 | 0.969 |
| Sao Tome and Principe | Male | 1 | 0.999 | 1 | 0.996 | 1 | 1 | 0.551 |
| Saudi Arabia | Both | < 0.001 | < 0.001 | < 0.001 | < 0.001 | < 0.001 | < 0.001 | 0.086 |
| Saudi Arabia | Female | < 0.001 | < 0.001 | 0.002 | < 0.001 | < 0.001 | < 0.001 | < 0.001 |
| Saudi Arabia | Male | < 0.001 | < 0.001 | < 0.001 | < 0.001 | < 0.001 | < 0.001 | 0.015 |
| Senegal | Both | < 0.001 | 0.087 | 0.993 | < 0.001 | 0.021 | 0.975 | < 0.001 |
| Senegal | Female | < 0.001 | 0.382 | 1 | 0.273 | 1 | 0.999 | 0.136 |
| Senegal | Male | < 0.001 | 0.443 | 1 | 0.002 | < 0.001 | 0.998 | < 0.001 |
| Serbia | Both | < 0.001 | < 0.001 | < 0.001 | < 0.001 | < 0.001 | < 0.001 | < 0.001 |
| Serbia | Female | < 0.001 | < 0.001 | < 0.001 | < 0.001 | < 0.001 | < 0.001 | < 0.001 |
| Serbia | Male | < 0.001 | < 0.001 | < 0.001 | < 0.001 | < 0.001 | < 0.001 | < 0.001 |
| Seychelles | Both | 0.998 | 0.995 | 1 | 0.624 | 0.986 | 1 | 0.07 |
| Seychelles | Female | 1 | 0.999 | 1 | 0.991 | 1 | 1 | 0.517 |
| Seychelles | Male | 0.992 | 0.991 | 1 | 0.557 | 0.966 | 1 | 0.054 |
| Sierra Leone | Both | < 0.001 | 0.003 | 0.999 | 0.004 | 1 | 0.997 | 0.14 |
| Sierra Leone | Female | < 0.001 | 0.006 | 1 | < 0.001 | 0.174 | 0.997 | < 0.001 |
| Sierra Leone | Male | < 0.001 | 0.234 | 1 | 0.153 | 0.363 | 1 | 0.21 |
| Singapore | Both | < 0.001 | 0.008 | < 0.001 | < 0.001 | < 0.001 | < 0.001 | < 0.001 |
| Singapore | Female | < 0.001 | 0.013 | < 0.001 | < 0.001 | < 0.001 | < 0.001 | < 0.001 |
| Singapore | Male | < 0.001 | 0.371 | < 0.001 | < 0.001 | < 0.001 | < 0.001 | < 0.001 |
| Slovakia | Both | < 0.001 | < 0.001 | < 0.001 | < 0.001 | < 0.001 | < 0.001 | < 0.001 |
| Slovakia | Female | < 0.001 | < 0.001 | < 0.001 | < 0.001 | < 0.001 | < 0.001 | < 0.001 |
| Slovakia | Male | < 0.001 | < 0.001 | < 0.001 | < 0.001 | < 0.001 | < 0.001 | < 0.001 |
| Slovenia | Both | < 0.001 | < 0.001 | < 0.001 | < 0.001 | < 0.001 | < 0.001 | < 0.001 |
| Slovenia | Female | 0.012 | < 0.001 | < 0.001 | < 0.001 | < 0.001 | < 0.001 | < 0.001 |
| Slovenia | Male | < 0.001 | < 0.001 | < 0.001 | < 0.001 | < 0.001 | < 0.001 | < 0.001 |
| Solomon Islands | Both | < 0.001 | 0.996 | 1 | 0.967 | 1 | 1 | 0.381 |
| Solomon Islands | Female | < 0.001 | 0.998 | 1 | 0.993 | 1 | 1 | 0.581 |
| Solomon Islands | Male | < 0.001 | 0.992 | 1 | 0.961 | 1 | 1 | 0.37 |
| Somalia | Both | < 0.001 | 0.069 | 0.994 | < 0.001 | 0.002 | 0.975 | < 0.001 |
| Somalia | Female | < 0.001 | 0.11 | 1 | < 0.001 | < 0.001 | 1 | < 0.001 |
| Somalia | Male | < 0.001 | 0.392 | 0.999 | 0.11 | 0.986 | 0.996 | 0.019 |
| South Africa | Both | < 0.001 | < 0.001 | < 0.001 | < 0.001 | < 0.001 | < 0.001 | < 0.001 |
| South Africa | Female | < 0.001 | < 0.001 | < 0.001 | < 0.001 | < 0.001 | < 0.001 | < 0.001 |
| South Africa | Male | < 0.001 | < 0.001 | < 0.001 | < 0.001 | < 0.001 | < 0.001 | < 0.001 |
| South Sudan | Both | < 0.001 | 0.345 | 1 | 0.47 | 1 | 1 | 0.674 |
| South Sudan | Female | < 0.001 | 0.887 | 1 | 0.588 | 1 | 1 | 0.099 |
| South Sudan | Male | < 0.001 | 0.372 | 1 | 0.47 | 1 | 1 | 0.491 |
| Spain | Both | < 0.001 | < 0.001 | < 0.001 | < 0.001 | < 0.001 | < 0.001 | < 0.001 |
| Spain | Female | < 0.001 | < 0.001 | < 0.001 | < 0.001 | < 0.001 | < 0.001 | < 0.001 |
| Spain | Male | < 0.001 | < 0.001 | < 0.001 | < 0.001 | < 0.001 | < 0.001 | < 0.001 |
| Sri Lanka | Both | < 0.001 | < 0.001 | < 0.001 | < 0.001 | < 0.001 | < 0.001 | < 0.001 |
| Sri Lanka | Female | < 0.001 | < 0.001 | < 0.001 | < 0.001 | < 0.001 | < 0.001 | < 0.001 |
| Sri Lanka | Male | < 0.001 | < 0.001 | < 0.001 | < 0.001 | < 0.001 | < 0.001 | < 0.001 |
| Sudan | Both | < 0.001 | < 0.001 | < 0.001 | < 0.001 | < 0.001 | < 0.001 | < 0.001 |
| Sudan | Female | < 0.001 | < 0.001 | < 0.001 | < 0.001 | < 0.001 | < 0.001 | < 0.001 |
| Sudan | Male | < 0.001 | < 0.001 | < 0.001 | < 0.001 | < 0.001 | < 0.001 | < 0.001 |
| Suriname | Both | < 0.001 | < 0.001 | 1 | < 0.001 | < 0.001 | 1 | < 0.001 |
| Suriname | Female | 0.669 | 0.008 | 1 | < 0.001 | < 0.001 | 1 | < 0.001 |
| Suriname | Male | < 0.001 | 0.024 | 1 | < 0.001 | 0.013 | 1 | < 0.001 |
| Sweden | Both | < 0.001 | < 0.001 | < 0.001 | < 0.001 | < 0.001 | < 0.001 | < 0.001 |
| Sweden | Female | < 0.001 | < 0.001 | < 0.001 | < 0.001 | < 0.001 | < 0.001 | < 0.001 |
| Sweden | Male | < 0.001 | < 0.001 | < 0.001 | < 0.001 | < 0.001 | < 0.001 | < 0.001 |
| Switzerland | Both | < 0.001 | < 0.001 | < 0.001 | < 0.001 | < 0.001 | < 0.001 | < 0.001 |
| Switzerland | Female | < 0.001 | < 0.001 | < 0.001 | < 0.001 | < 0.001 | < 0.001 | < 0.001 |
| Switzerland | Male | < 0.001 | < 0.001 | < 0.001 | < 0.001 | < 0.001 | < 0.001 | < 0.001 |
| Syrian Arab Republic | Both | < 0.001 | < 0.001 | < 0.001 | < 0.001 | < 0.001 | < 0.001 | < 0.001 |
| Syrian Arab Republic | Female | < 0.001 | < 0.001 | < 0.001 | < 0.001 | < 0.001 | < 0.001 | < 0.001 |
| Syrian Arab Republic | Male | < 0.001 | < 0.001 | < 0.001 | < 0.001 | < 0.001 | < 0.001 | < 0.001 |
| Taiwan (Province of China) | Both | < 0.001 | < 0.001 | < 0.001 | < 0.001 | < 0.001 | < 0.001 | < 0.001 |
| Taiwan (Province of China) | Female | < 0.001 | < 0.001 | < 0.001 | < 0.001 | < 0.001 | < 0.001 | < 0.001 |
| Taiwan (Province of China) | Male | < 0.001 | < 0.001 | < 0.001 | < 0.001 | < 0.001 | < 0.001 | < 0.001 |
| Tajikistan | Both | < 0.001 | 0.901 | < 0.001 | 0.116 | < 0.001 | < 0.001 | 0.008 |
| Tajikistan | Female | < 0.001 | 0.207 | < 0.001 | 0.162 | < 0.001 | < 0.001 | 0.142 |
| Tajikistan | Male | < 0.001 | < 0.001 | < 0.001 | < 0.001 | < 0.001 | < 0.001 | < 0.001 |
| Thailand | Both | < 0.001 | < 0.001 | 0.577 | < 0.001 | < 0.001 | 0.525 | < 0.001 |
| Thailand | Female | < 0.001 | < 0.001 | < 0.001 | < 0.001 | < 0.001 | < 0.001 | < 0.001 |
| Thailand | Male | < 0.001 | < 0.001 | 0.005 | < 0.001 | < 0.001 | 0.002 | < 0.001 |
| Timor-Leste | Both | 0.233 | 0.579 | 1 | < 0.001 | 0.091 | 1 | < 0.001 |
| Timor-Leste | Female | 0.807 | 0.947 | 1 | 0.637 | 0.998 | 1 | 0.164 |
| Timor-Leste | Male | 0.833 | 0.645 | 1 | < 0.001 | 0.12 | 1 | < 0.001 |
| Togo | Both | < 0.001 | 0.348 | 1 | 0.439 | 1 | 1 | 0.954 |
| Togo | Female | < 0.001 | 0.813 | 1 | 0.833 | 1 | 1 | 0.337 |
| Togo | Male | < 0.001 | 0.008 | 1 | 0.017 | 1 | 1 | 0.279 |
| Tokelau | Both | 1 | 1 | 1 | 1 | 1 | 1 | 0.997 |
| Tokelau | Female | 1 | 1 | 1 | 1 | 1 | 1 | 0.995 |
| Tokelau | Male | 1 | 1 | 1 | 1 | 1 | 1 | 0.983 |
| Tonga | Both | 0.95 | 0.985 | 1 | 0.996 | 1 | 1 | 0.937 |
| Tonga | Female | 1 | 0.997 | 1 | 1 | 1 | 1 | 0.908 |
| Tonga | Male | 0.949 | 0.949 | 1 | 0.981 | 1 | 1 | 0.878 |
| Trinidad and Tobago | Both | < 0.001 | < 0.001 | 0.878 | < 0.001 | < 0.001 | 0.797 | < 0.001 |
| Trinidad and Tobago | Female | < 0.001 | < 0.001 | 0.52 | < 0.001 | < 0.001 | 0.337 | < 0.001 |
| Trinidad and Tobago | Male | < 0.001 | 0.002 | 0.994 | < 0.001 | < 0.001 | 0.989 | < 0.001 |
| Tunisia | Both | < 0.001 | 0.001 | < 0.001 | < 0.001 | < 0.001 | < 0.001 | < 0.001 |
| Tunisia | Female | < 0.001 | 0.065 | 0.001 | < 0.001 | < 0.001 | < 0.001 | < 0.001 |
| Tunisia | Male | < 0.001 | 0.027 | < 0.001 | < 0.001 | < 0.001 | < 0.001 | < 0.001 |
| Turkey | Both | < 0.001 | < 0.001 | < 0.001 | < 0.001 | < 0.001 | < 0.001 | < 0.001 |
| Turkey | Female | 0.651 | < 0.001 | < 0.001 | < 0.001 | < 0.001 | < 0.001 | < 0.001 |
| Turkey | Male | < 0.001 | < 0.001 | < 0.001 | < 0.001 | < 0.001 | < 0.001 | < 0.001 |
| Turkmenistan | Both | < 0.001 | < 0.001 | 0.222 | < 0.001 | < 0.001 | 0.124 | < 0.001 |
| Turkmenistan | Female | < 0.001 | < 0.001 | 0.964 | < 0.001 | < 0.001 | 0.903 | < 0.001 |
| Turkmenistan | Male | < 0.001 | < 0.001 | 0.074 | < 0.001 | < 0.001 | 0.032 | < 0.001 |
| Tuvalu | Both | 1 | 1 | 1 | 1 | 1 | 1 | 0.892 |
| Tuvalu | Female | 1 | 1 | 1 | 1 | 1 | 1 | 0.95 |
| Tuvalu | Male | 1 | 1 | 1 | 1 | 1 | 1 | 0.978 |
| Uganda | Both | < 0.001 | < 0.001 | 0.69 | < 0.001 | 0.568 | 0.6 | 0.214 |
| Uganda | Female | < 0.001 | < 0.001 | 0.985 | < 0.001 | 0.313 | 0.949 | 0.03 |
| Uganda | Male | < 0.001 | < 0.001 | 0.883 | < 0.001 | 0.174 | 0.776 | 0.046 |
| Ukraine | Both | < 0.001 | < 0.001 | < 0.001 | < 0.001 | < 0.001 | < 0.001 | < 0.001 |
| Ukraine | Female | < 0.001 | < 0.001 | < 0.001 | < 0.001 | < 0.001 | < 0.001 | 0.616 |
| Ukraine | Male | < 0.001 | < 0.001 | < 0.001 | < 0.001 | < 0.001 | < 0.001 | < 0.001 |
| United Arab Emirates | Both | < 0.001 | 0.007 | < 0.001 | < 0.001 | < 0.001 | < 0.001 | < 0.001 |
| United Arab Emirates | Female | < 0.001 | < 0.001 | 0.443 | < 0.001 | < 0.001 | 0.291 | 0.003 |
| United Arab Emirates | Male | < 0.001 | 0.013 | < 0.001 | < 0.001 | < 0.001 | < 0.001 | < 0.001 |
| United Kingdom | Both | < 0.001 | < 0.001 | < 0.001 | < 0.001 | < 0.001 | < 0.001 | < 0.001 |
| United Kingdom | Female | < 0.001 | < 0.001 | < 0.001 | < 0.001 | < 0.001 | < 0.001 | < 0.001 |
| United Kingdom | Male | < 0.001 | < 0.001 | < 0.001 | < 0.001 | < 0.001 | < 0.001 | < 0.001 |
| United Republic of Tanzania | Both | < 0.001 | 0.881 | 0.613 | 0.862 | 0.006 | 0.441 | 0.358 |
| United Republic of Tanzania | Female | < 0.001 | 0.007 | 0.077 | < 0.001 | < 0.001 | 0.035 | < 0.001 |
| United Republic of Tanzania | Male | < 0.001 | 0.378 | < 0.001 | 0.129 | 0.001 | < 0.001 | 0.035 |
| United States of America | Both | < 0.001 | < 0.001 | < 0.001 | < 0.001 | < 0.001 | < 0.001 | < 0.001 |
| United States of America | Female | < 0.001 | < 0.001 | < 0.001 | < 0.001 | < 0.001 | < 0.001 | < 0.001 |
| United States of America | Male | < 0.001 | < 0.001 | < 0.001 | < 0.001 | < 0.001 | < 0.001 | < 0.001 |
| United States Virgin Islands | Both | 0.991 | 0.896 | 1 | 0.845 | 1 | 1 | 0.313 |
| United States Virgin Islands | Female | 1 | 0.994 | 1 | 0.945 | 0.987 | 1 | 0.31 |
| United States Virgin Islands | Male | 0.981 | 0.764 | 1 | 0.855 | 1 | 1 | 0.707 |
| Uruguay | Both | < 0.001 | < 0.001 | 0.988 | < 0.001 | < 0.001 | 0.984 | < 0.001 |
| Uruguay | Female | 0.188 | < 0.001 | 0.596 | < 0.001 | < 0.001 | 0.427 | < 0.001 |
| Uruguay | Male | < 0.001 | 0.007 | 1 | < 0.001 | < 0.001 | 1 | < 0.001 |
| Uzbekistan | Both | < 0.001 | < 0.001 | < 0.001 | < 0.001 | < 0.001 | < 0.001 | < 0.001 |
| Uzbekistan | Female | < 0.001 | < 0.001 | < 0.001 | < 0.001 | < 0.001 | < 0.001 | < 0.001 |
| Uzbekistan | Male | < 0.001 | < 0.001 | < 0.001 | < 0.001 | < 0.001 | < 0.001 | < 0.001 |
| Vanuatu | Both | < 0.001 | 0.982 | 1 | 0.913 | 1 | 1 | 0.316 |
| Vanuatu | Female | 0.946 | 0.992 | 1 | 0.976 | 1 | 1 | 0.489 |
| Vanuatu | Male | < 0.001 | 0.996 | 1 | 0.968 | 1 | 1 | 0.384 |
| Venezuela (Bolivarian Republic of) | Both | < 0.001 | < 0.001 | 0.358 | < 0.001 | < 0.001 | 0.486 | < 0.001 |
| Venezuela (Bolivarian Republic of) | Female | < 0.001 | < 0.001 | < 0.001 | < 0.001 | < 0.001 | < 0.001 | < 0.001 |
| Venezuela (Bolivarian Republic of) | Male | < 0.001 | < 0.001 | 0.305 | < 0.001 | < 0.001 | 0.38 | < 0.001 |
| Viet Nam | Both | < 0.001 | < 0.001 | < 0.001 | < 0.001 | < 0.001 | < 0.001 | < 0.001 |
| Viet Nam | Female | < 0.001 | < 0.001 | < 0.001 | < 0.001 | < 0.001 | < 0.001 | < 0.001 |
| Viet Nam | Male | < 0.001 | < 0.001 | < 0.001 | < 0.001 | < 0.001 | < 0.001 | < 0.001 |
| Yemen | Both | < 0.001 | < 0.001 | < 0.001 | < 0.001 | < 0.001 | < 0.001 | < 0.001 |
| Yemen | Female | < 0.001 | < 0.001 | < 0.001 | < 0.001 | < 0.001 | < 0.001 | < 0.001 |
| Yemen | Male | < 0.001 | < 0.001 | < 0.001 | < 0.001 | < 0.001 | < 0.001 | < 0.001 |
| Zambia | Both | < 0.001 | < 0.001 | 0.227 | < 0.001 | < 0.001 | 0.113 | < 0.001 |
| Zambia | Female | < 0.001 | 0.033 | 0.834 | < 0.001 | < 0.001 | 0.675 | < 0.001 |
| Zambia | Male | < 0.001 | < 0.001 | 0.183 | < 0.001 | < 0.001 | 0.086 | < 0.001 |
| Zimbabwe | Both | < 0.001 | < 0.001 | 0.001 | < 0.001 | < 0.001 | < 0.001 | < 0.001 |
| Zimbabwe | Female | < 0.001 | < 0.001 | < 0.001 | < 0.001 | < 0.001 | < 0.001 | < 0.001 |
| Zimbabwe | Male | < 0.001 | < 0.001 | 0.033 | < 0.001 | < 0.001 | 0.011 | < 0.001 |

Abbreviations: IHD, ischemic heart disease; GBD, Global Burden of Disease; SDI, socio-demographic index; RR, rate ratio.

**Table S3. Net drift values in gender groups across 5 socio-demographic index (SDI) quintiles, 21 GBD regions, and 204 countries and territories.**

| **Location** | **Gender** | **Net Drift (%/year)** | **Lower bound of 95% CI** | **Upper bound of 95% CI** |
| --- | --- | --- | --- | --- |
| Global | Both | -1.148968242 | -1.205486802 | -1.092417349 |
| Global | Male | -1.037380093 | -1.11172161 | -0.962982687 |
| Global | Female | -1.329783685 | -1.406383287 | -1.253124571 |
| High SDI | Both | -2.885454812 | -3.065484382 | -2.705090887 |
| High SDI | Male | -2.906852133 | -3.08282718 | -2.730557563 |
| High SDI | Female | -2.939474725 | -3.190596441 | -2.687701603 |
| High-middle SDI | Both | -2.000582111 | -2.169408157 | -1.831464721 |
| High-middle SDI | Male | -1.875671367 | -2.03138194 | -1.719713308 |
| High-middle SDI | Female | -2.301434561 | -2.545772312 | -2.056484204 |
| Middle SDI | Both | -0.312397609 | -0.371554947 | -0.253205146 |
| Middle SDI | Male | -0.040873302 | -0.114011995 | 0.032318944 |
| Middle SDI | Female | -0.804083362 | -0.894007359 | -0.714077773 |
| Low-middle SDI | Both | -0.240066971 | -0.318931401 | -0.161140146 |
| Low-middle SDI | Male | -0.025408528 | -0.110881164 | 0.060137245 |
| Low-middle SDI | Female | -0.504951815 | -0.619039366 | -0.390733293 |
| Low SDI | Both | -0.434896202 | -0.495070673 | -0.374685341 |
| Low SDI | Male | -0.333211574 | -0.406935729 | -0.259432844 |
| Low SDI | Female | -0.552728697 | -0.610533558 | -0.494890216 |
| High-income Asia Pacific | Both | -3.461325299 | -3.734062121 | -3.18781577 |
| High-income Asia Pacific | Male | -3.123729825 | -3.292348577 | -2.954817072 |
| High-income Asia Pacific | Female | -4.624089741 | -4.98086005 | -4.265979858 |
| High-income North America | Both | -2.404189583 | -2.565672742 | -2.242438789 |
| High-income North America | Male | -2.506185613 | -2.685047434 | -2.32699505 |
| High-income North America | Female | -2.239825996 | -2.470310784 | -2.00879652 |
| Western Europe | Both | -3.915628981 | -4.195326048 | -3.635115351 |
| Western Europe | Male | -3.85838172 | -4.11587648 | -3.600195464 |
| Western Europe | Female | -4.169360502 | -4.556199075 | -3.780954052 |
| Australasia | Both | -4.318724058 | -4.662034391 | -3.974177471 |
| Australasia | Male | -4.173942454 | -4.477409181 | -3.86951164 |
| Australasia | Female | -4.715648953 | -5.214322255 | -4.2143521 |
| Andean Latin America | Both | -1.975539194 | -2.069792766 | -1.881194908 |
| Andean Latin America | Male | -1.6424489 | -1.75688936 | -1.527875131 |
| Andean Latin America | Female | -2.547083197 | -2.704468323 | -2.389443486 |
| Tropical Latin America | Both | -2.183916222 | -2.265474535 | -2.102289849 |
| Tropical Latin America | Male | -1.953058313 | -2.011289448 | -1.894792574 |
| Tropical Latin America | Female | -2.543628075 | -2.680357324 | -2.406706728 |
| Central Latin America | Both | -1.087740108 | -1.186993922 | -0.988386598 |
| Central Latin America | Male | -0.750692314 | -0.837440938 | -0.663867801 |
| Central Latin America | Female | -1.653769494 | -1.802774554 | -1.504538333 |
| Southern Latin America | Both | -2.737984643 | -2.948494638 | -2.527018041 |
| Southern Latin America | Male | -2.665957024 | -2.833039478 | -2.498587264 |
| Southern Latin America | Female | -2.86597593 | -3.163747784 | -2.567288426 |
| Caribbean | Both | -1.158592765 | -1.24590069 | -1.07120765 |
| Caribbean | Male | -1.125823259 | -1.2325106 | -1.019020676 |
| Caribbean | Female | -1.215510417 | -1.347875962 | -1.082967272 |
| Central Europe | Both | -3.341183971 | -3.589266891 | -3.092462687 |
| Central Europe | Male | -3.306116632 | -3.510740968 | -3.10105835 |
| Central Europe | Female | -3.519596172 | -3.869285598 | -3.168634701 |
| Eastern Europe | Both | -1.193267304 | -1.489405515 | -0.896238856 |
| Eastern Europe | Male | -1.17217061 | -1.397817263 | -0.946007574 |
| Eastern Europe | Female | -1.303666297 | -1.770862691 | -0.834247828 |
| Central Asia | Both | -0.615896443 | -0.755830287 | -0.475765293 |
| Central Asia | Male | -0.674418094 | -0.812791145 | -0.535852002 |
| Central Asia | Female | -0.528180961 | -0.736627866 | -0.319296331 |
| North Africa and Middle East | Both | -1.708669657 | -1.774391523 | -1.642903817 |
| North Africa and Middle East | Male | -1.709940408 | -1.763982411 | -1.655868674 |
| North Africa and Middle East | Female | -1.739948099 | -1.841197214 | -1.638594547 |
| South Asia | Both | -0.272834561 | -0.387235505 | -0.158302231 |
| South Asia | Male | -0.090546402 | -0.208728488 | 0.027775647 |
| South Asia | Female | -0.43599636 | -0.60193892 | -0.269776763 |
| Southeast Asia | Both | -0.063471082 | -0.100301508 | -0.026627076 |
| Southeast Asia | Male | 0.282830524 | 0.242544079 | 0.32313316 |
| Southeast Asia | Female | -0.698169196 | -0.742350567 | -0.65396816 |
| East Asia | Both | 0.119607025 | -0.037366753 | 0.276827303 |
| East Asia | Male | 0.627884354 | 0.477065002 | 0.77893009 |
| East Asia | Female | -0.962525334 | -1.221791771 | -0.702578393 |
| Oceania | Both | 0.327669887 | 0.176008239 | 0.479561143 |
| Oceania | Male | 0.194491836 | 0.002083803 | 0.38727007 |
| Oceania | Female | 0.604088632 | 0.330436808 | 0.878486843 |
| Western Sub-Saharan Africa | Both | -0.540683157 | -0.60702567 | -0.474296362 |
| Western Sub-Saharan Africa | Male | -0.49093662 | -0.559493815 | -0.42233216 |
| Western Sub-Saharan Africa | Female | -0.492795539 | -0.563642307 | -0.421898295 |
| Eastern Sub-Saharan Africa | Both | -0.644075785 | -0.688972526 | -0.599158747 |
| Eastern Sub-Saharan Africa | Male | -0.610928172 | -0.667994078 | -0.553829482 |
| Eastern Sub-Saharan Africa | Female | -0.703595238 | -0.780836868 | -0.626293476 |
| Central Sub-Saharan Africa | Both | -0.659713966 | -0.737757374 | -0.581609198 |
| Central Sub-Saharan Africa | Male | -0.826535247 | -0.930841092 | -0.722119583 |
| Central Sub-Saharan Africa | Female | -0.566286669 | -0.698297921 | -0.434099921 |
| Southern Sub-Saharan Africa | Both | -0.640284619 | -0.850100218 | -0.43002502 |
| Southern Sub-Saharan Africa | Male | -0.70427657 | -0.899911997 | -0.508254935 |
| Southern Sub-Saharan Africa | Female | -0.46592941 | -0.810968715 | -0.119689851 |
| Chile | Both | -2.123462542 | -2.519623009 | -1.725692079 |
| Chile | Male | -1.760182822 | -2.04044758 | -1.47911622 |
| Chile | Female | -3.130711279 | -3.755796187 | -2.501566581 |
| Djibouti | Both | 0.503613518 | -0.383857479 | 1.398990912 |
| Djibouti | Male | 0.344510462 | -0.788570707 | 1.490532409 |
| Djibouti | Female | 0.779954034 | -0.874811842 | 2.462344071 |
| Lesotho | Both | 2.515567953 | 1.952789908 | 3.081452526 |
| Lesotho | Male | 1.919223898 | 1.187529826 | 2.6562089 |
| Lesotho | Female | 3.559496053 | 2.539023524 | 4.590124366 |
| Greece | Both | -1.449859828 | -1.767314437 | -1.131379314 |
| Greece | Male | -1.427619684 | -1.73964577 | -1.114602759 |
| Greece | Female | -1.509968183 | -2.069080048 | -0.947664209 |
| Georgia | Both | -3.347897501 | -3.788738982 | -2.905036078 |
| Georgia | Male | -2.910259065 | -3.306381252 | -2.512514095 |
| Georgia | Female | -4.680816746 | -5.344656003 | -4.012321835 |
| Timor-Leste | Both | 1.432538579 | 0.769306591 | 2.100135751 |
| Timor-Leste | Male | 1.861452521 | 1.01449297 | 2.715513435 |
| Timor-Leste | Female | 0.786601538 | -0.318356004 | 1.903807384 |
| Bermuda | Both | -4.467133975 | -8.080590593 | -0.711628264 |
| Bermuda | Male | -4.048703959 | -8.126651895 | 0.210250326 |
| Bermuda | Female | -5.757126906 | -13.49236785 | 2.669774998 |
| Mexico | Both | -0.12067523 | -0.282570692 | 0.041483076 |
| Mexico | Male | 0.204648157 | 0.073235987 | 0.336232892 |
| Mexico | Female | -0.789010217 | -1.03602645 | -0.541377427 |
| Japan | Both | -2.420958602 | -2.838617866 | -2.001503982 |
| Japan | Male | -2.149486816 | -2.404202655 | -1.894106192 |
| Japan | Female | -3.243370526 | -3.816783731 | -2.666538817 |
| Liberia | Both | -0.359678223 | -0.766776754 | 0.049090407 |
| Liberia | Male | -0.540818992 | -1.084047292 | 0.005392617 |
| Liberia | Female | -0.167298369 | -0.786505636 | 0.455773469 |
| Haiti | Both | -0.785011909 | -0.951047163 | -0.61869833 |
| Haiti | Male | -0.917095469 | -1.167376346 | -0.666180788 |
| Haiti | Female | -0.642619402 | -0.869273761 | -0.415446816 |
| United States Virgin Islands | Both | -1.098386155 | -3.198996853 | 1.047808422 |
| United States Virgin Islands | Male | -0.480475001 | -2.946265182 | 2.047962138 |
| United States Virgin Islands | Female | -2.223476444 | -6.373496613 | 2.110494496 |
| France | Both | -3.114945867 | -3.488608613 | -2.739836412 |
| France | Male | -3.012334839 | -3.336045268 | -2.687540361 |
| France | Female | -3.27586817 | -3.758358176 | -2.790959288 |
| Poland | Both | -4.752565934 | -5.14293234 | -4.360593049 |
| Poland | Male | -4.736626937 | -5.090435186 | -4.381499746 |
| Poland | Female | -4.936962283 | -5.490763111 | -4.379916319 |
| Iceland | Both | -3.927535447 | -5.52809358 | -2.299860403 |
| Iceland | Male | -3.560048647 | -5.358359592 | -1.727567519 |
| Iceland | Female | -5.23287817 | -8.798782108 | -1.527549878 |
| Jamaica | Both | -0.611686537 | -1.190840673 | -0.02913778 |
| Jamaica | Male | -0.613674511 | -1.370948556 | 0.149413885 |
| Jamaica | Female | -0.652244515 | -1.577318174 | 0.281523901 |
| Nicaragua | Both | 0.170523712 | -0.147762533 | 0.489824517 |
| Nicaragua | Male | 0.043187704 | -0.371241584 | 0.459340907 |
| Nicaragua | Female | 0.281181205 | -0.221918729 | 0.786817864 |
| Lebanon | Both | -1.175802478 | -1.351744682 | -0.999546476 |
| Lebanon | Male | -0.771596048 | -0.97963784 | -0.56311716 |
| Lebanon | Female | -1.801697545 | -2.145247459 | -1.456941491 |
| Bhutan | Both | -0.264850586 | -0.905472813 | 0.379913108 |
| Bhutan | Male | 0.124521529 | -0.727750951 | 0.984110942 |
| Bhutan | Female | -0.981608033 | -1.998098631 | 0.04542576 |
| Uruguay | Both | -2.971781394 | -3.489484728 | -2.451300987 |
| Uruguay | Male | -2.760263411 | -3.36575711 | -2.150975791 |
| Uruguay | Female | -3.406756362 | -4.43746012 | -2.364935798 |
| Kazakhstan | Both | -2.896174074 | -3.312185781 | -2.478372423 |
| Kazakhstan | Male | -2.848994412 | -3.17719595 | -2.519680364 |
| Kazakhstan | Female | -3.14871393 | -3.704276611 | -2.58994602 |
| China | Both | 0.132814999 | -0.029631493 | 0.295525457 |
| China | Male | 0.67288388 | 0.521194242 | 0.824802421 |
| China | Female | -0.974658025 | -1.244007255 | -0.704574167 |
| Eritrea | Both | 0.387443495 | 0.025599362 | 0.750596605 |
| Eritrea | Male | -0.193930024 | -0.745509089 | 0.360714288 |
| Eritrea | Female | 1.368434968 | 0.689993703 | 2.051447516 |
| South Africa | Both | -1.187683775 | -1.479567637 | -0.894935156 |
| South Africa | Male | -1.09040269 | -1.343778436 | -0.836376208 |
| South Africa | Female | -1.286015824 | -1.741289036 | -0.828633143 |
| India | Both | -0.481071115 | -0.629513176 | -0.332407308 |
| India | Male | -0.346667871 | -0.500952882 | -0.192143623 |
| India | Female | -0.555835749 | -0.769964411 | -0.341245019 |
| Mali | Both | -0.444627418 | -0.663673194 | -0.225098626 |
| Mali | Male | -0.229848764 | -0.573651179 | 0.11514247 |
| Mali | Female | -0.559496348 | -0.845001707 | -0.273168908 |
| Cook Islands | Both | -0.913729725 | -5.161833515 | 3.524660176 |
| Cook Islands | Male | -0.636776614 | -5.581208517 | 4.566580515 |
| Cook Islands | Female | -1.369367968 | -10.17170942 | 8.295521516 |
| Ethiopia | Both | -2.234609428 | -2.342047172 | -2.127053487 |
| Ethiopia | Male | -2.23576592 | -2.37128814 | -2.100055576 |
| Ethiopia | Female | -2.247966338 | -2.39121596 | -2.104506484 |
| Viet Nam | Both | -0.724603214 | -0.805522197 | -0.643618221 |
| Viet Nam | Male | -0.38043528 | -0.477780387 | -0.282994956 |
| Viet Nam | Female | -1.592165804 | -1.745379244 | -1.438713449 |
| Burundi | Both | -0.931490148 | -1.164503564 | -0.697927382 |
| Burundi | Male | -1.266114382 | -1.566236879 | -0.965076818 |
| Burundi | Female | -0.724455481 | -1.116882277 | -0.330471304 |
| Romania | Both | -2.632933905 | -2.980984923 | -2.283634271 |
| Romania | Male | -2.495592169 | -2.745558154 | -2.244983714 |
| Romania | Female | -2.957239257 | -3.483789925 | -2.427815956 |
| Jordan | Both | -3.077304365 | -3.309980955 | -2.844067857 |
| Jordan | Male | -2.612628565 | -2.88934349 | -2.335125146 |
| Jordan | Female | -4.245766105 | -4.719380413 | -3.769797587 |
| Iraq | Both | -1.854925815 | -1.968368923 | -1.741351428 |
| Iraq | Male | -1.790845492 | -1.906027706 | -1.67552803 |
| Iraq | Female | -1.9602855 | -2.086115628 | -1.834293667 |
| Namibia | Both | -0.759891657 | -1.345193529 | -0.171117292 |
| Namibia | Male | -0.458910201 | -1.181444205 | 0.268906772 |
| Namibia | Female | -1.30807572 | -2.377075308 | -0.227370273 |
| Libya | Both | -0.424980829 | -0.602973078 | -0.246669845 |
| Libya | Male | -0.385716935 | -0.613276418 | -0.157636425 |
| Libya | Female | -0.423350929 | -0.711502111 | -0.134363486 |
| Grenada | Both | -2.35948949 | -4.57073042 | -0.097010753 |
| Grenada | Male | -2.271275231 | -5.035695546 | 0.573617633 |
| Grenada | Female | -2.446287877 | -6.225152762 | 1.484854726 |
| Guinea | Both | 1.114694025 | 0.862367612 | 1.367651682 |
| Guinea | Male | 1.288877458 | 0.950569221 | 1.628319442 |
| Guinea | Female | 0.92409925 | 0.542913105 | 1.306730578 |
| Armenia | Both | -1.832379248 | -2.136793779 | -1.5270178 |
| Armenia | Male | -1.613379116 | -1.95220284 | -1.273384519 |
| Armenia | Female | -2.489463206 | -3.231498061 | -1.74173832 |
| Sri Lanka | Both | -2.109547858 | -2.309809827 | -1.908875359 |
| Sri Lanka | Male | -1.902229992 | -2.104902803 | -1.699137587 |
| Sri Lanka | Female | -2.259842061 | -2.48711676 | -2.032037649 |
| Saint Lucia | Both | -3.206456169 | -5.326455249 | -1.038984522 |
| Saint Lucia | Male | -3.037569912 | -5.56534548 | -0.44213222 |
| Saint Lucia | Female | -3.716709381 | -7.688319962 | 0.425775467 |
| Canada | Both | -3.471494407 | -3.737724042 | -3.20452847 |
| Canada | Male | -3.46773519 | -3.723711952 | -3.211077843 |
| Canada | Female | -3.524105733 | -3.908963839 | -3.137706216 |
| Ireland | Both | -5.045027494 | -5.67260515 | -4.413274447 |
| Ireland | Male | -4.969285708 | -5.690938551 | -4.242110779 |
| Ireland | Female | -5.25886328 | -6.547996245 | -3.951947243 |
| Germany | Both | -4.134909769 | -4.669651379 | -3.597168604 |
| Germany | Male | -4.171836973 | -4.631657092 | -3.709799824 |
| Germany | Female | -4.277317482 | -5.121417972 | -3.425707334 |
| Greenland | Both | -3.483118692 | -8.063541642 | 1.325508333 |
| Greenland | Male | -2.997796038 | -8.634642734 | 2.986819677 |
| Greenland | Female | -4.273268483 | -12.14298909 | 4.301375973 |
| Mauritania | Both | -1.656683456 | -2.11569473 | -1.195519729 |
| Mauritania | Male | -2.122292245 | -2.752035599 | -1.488470898 |
| Mauritania | Female | -1.202710254 | -1.879775906 | -0.5209726 |
| Guatemala | Both | -2.503644704 | -2.656817711 | -2.350230675 |
| Guatemala | Male | -2.050473293 | -2.251757686 | -1.848774413 |
| Guatemala | Female | -2.94776198 | -3.189550744 | -2.705369337 |
| Serbia | Both | -2.529184632 | -3.121717254 | -1.933027928 |
| Serbia | Male | -2.34807078 | -2.953092673 | -1.739276984 |
| Serbia | Female | -2.942874578 | -3.780787901 | -2.097664388 |
| Singapore | Both | -3.819420594 | -4.339716341 | -3.296294962 |
| Singapore | Male | -3.589213894 | -4.152344007 | -3.022775244 |
| Singapore | Female | -5.080889572 | -6.631790069 | -3.504227712 |
| Republic of Korea | Both | -6.056283115 | -6.229410098 | -5.882836491 |
| Republic of Korea | Male | -5.7063684 | -5.870861384 | -5.541587961 |
| Republic of Korea | Female | -7.655059016 | -7.948962967 | -7.360216676 |
| Panama | Both | -1.875062822 | -2.335651018 | -1.412302476 |
| Panama | Male | -1.614539559 | -2.173429507 | -1.052456634 |
| Panama | Female | -2.333089235 | -3.157994682 | -1.501157198 |
| Tokelau | Both | -0.025394768 | -11.47548761 | 12.90569608 |
| Tokelau | Male | -0.172257561 | -15.11065355 | 17.39492147 |
| Tokelau | Female | 0.059510062 | -17.24679486 | 20.98510912 |
| Kyrgyzstan | Both | -0.562578984 | -0.992173283 | -0.131120679 |
| Kyrgyzstan | Male | -0.646885905 | -1.00674017 | -0.28572352 |
| Kyrgyzstan | Female | -0.615803339 | -1.283505832 | 0.056415386 |
| Sudan | Both | -1.545283186 | -1.612400032 | -1.478120555 |
| Sudan | Male | -1.548647377 | -1.635989491 | -1.461227709 |
| Sudan | Female | -1.552510251 | -1.659755684 | -1.445147862 |
| Democratic People's Republic of Korea | Both | 0.654786117 | 0.560396723 | 0.749264109 |
| Democratic People's Republic of Korea | Male | 0.531782525 | 0.409392049 | 0.654322185 |
| Democratic People's Republic of Korea | Female | 0.513188984 | 0.346281679 | 0.680373908 |
| Comoros | Both | -0.309063867 | -1.193374107 | 0.583160868 |
| Comoros | Male | -0.71525388 | -1.866066095 | 0.449053857 |
| Comoros | Female | 0.212473278 | -1.203506569 | 1.648747357 |
| Taiwan (Province of China) | Both | -1.864384394 | -2.096426467 | -1.631792355 |
| Taiwan (Province of China) | Male | -1.309476695 | -1.544362326 | -1.074030697 |
| Taiwan (Province of China) | Female | -3.155422805 | -3.590287366 | -2.718596748 |
| San Marino | Both | -1.363658598 | -9.096397529 | 7.026867811 |
| San Marino | Male | -1.333341242 | -10.22288264 | 8.436423849 |
| San Marino | Female | -1.407155943 | -16.93093643 | 17.0176776 |
| Madagascar | Both | -0.175914541 | -0.31484135 | -0.036794116 |
| Madagascar | Male | -0.259180551 | -0.425407765 | -0.092675841 |
| Madagascar | Female | 0.035899656 | -0.229791582 | 0.302298437 |
| Afghanistan | Both | -1.351028502 | -1.431159603 | -1.270832259 |
| Afghanistan | Male | -1.416357641 | -1.518117243 | -1.314492892 |
| Afghanistan | Female | -1.256547531 | -1.357281795 | -1.155710396 |
| Maldives | Both | -4.027246016 | -4.983989808 | -3.060868494 |
| Maldives | Male | -3.673637411 | -4.77763606 | -2.556839112 |
| Maldives | Female | -5.670556853 | -7.753681151 | -3.540391038 |
| Saint Vincent and the Grenadines | Both | -1.913811009 | -3.74268552 | -0.050188157 |
| Saint Vincent and the Grenadines | Male | -1.90077706 | -4.306331545 | 0.565248431 |
| Saint Vincent and the Grenadines | Female | -2.150772763 | -5.030903289 | 0.816703564 |
| South Sudan | Both | -0.068997615 | -0.389502667 | 0.25253869 |
| South Sudan | Male | -0.137910032 | -0.529220065 | 0.254939383 |
| South Sudan | Female | 0.493341864 | -0.093109522 | 1.083235708 |
| Morocco | Both | -1.07938363 | -1.245407053 | -0.913081093 |
| Morocco | Male | -1.281345723 | -1.565637787 | -0.996232585 |
| Morocco | Female | -0.85923581 | -0.966279869 | -0.752076048 |
| El Salvador | Both | -1.489984101 | -1.709924668 | -1.269551381 |
| El Salvador | Male | -1.105576061 | -1.382304436 | -0.828071167 |
| El Salvador | Female | -1.945025736 | -2.315027452 | -1.573622562 |
| United States of America | Both | -2.318638491 | -2.482168738 | -2.154834016 |
| United States of America | Male | -2.426781579 | -2.60932673 | -2.243894273 |
| United States of America | Female | -2.148965941 | -2.385933798 | -1.911422821 |
| Azerbaijan | Both | -0.953672018 | -1.229957567 | -0.676613627 |
| Azerbaijan | Male | -1.036758637 | -1.257289976 | -0.815734765 |
| Azerbaijan | Female | -1.118653834 | -1.523237249 | -0.712408222 |
| Croatia | Both | -3.044019432 | -3.863640749 | -2.217410341 |
| Croatia | Male | -2.983505524 | -3.49479839 | -2.469503785 |
| Croatia | Female | -3.508234366 | -4.69312318 | -2.308614594 |
| Tuvalu | Both | 0.248444642 | -3.287861945 | 3.914057277 |
| Tuvalu | Male | -0.064175604 | -4.615910984 | 4.704768906 |
| Tuvalu | Female | 0.208048894 | -6.074443552 | 6.910764682 |
| Bangladesh | Both | 0.06404193 | -0.127545505 | 0.255996891 |
| Bangladesh | Male | 0.197058707 | -0.039017312 | 0.433692262 |
| Bangladesh | Female | -0.00705797 | -0.285389017 | 0.272049976 |
| North Macedonia | Both | -1.81171631 | -2.16315078 | -1.459019471 |
| North Macedonia | Male | -1.778171008 | -2.20348523 | -1.351007108 |
| North Macedonia | Female | -2.042651827 | -2.684410553 | -1.396660949 |
| Czechia | Both | -4.195311457 | -4.72424212 | -3.663444394 |
| Czechia | Male | -4.243019015 | -4.687736632 | -3.796226389 |
| Czechia | Female | -4.304178437 | -5.118540986 | -3.482826257 |
| Ukraine | Both | 0.723228904 | 0.367478608 | 1.080240149 |
| Ukraine | Male | 0.964388888 | 0.630283928 | 1.299603117 |
| Ukraine | Female | 0.132472605 | -0.384940105 | 0.65257282 |
| Israel | Both | -5.877864925 | -6.289787859 | -5.464131297 |
| Israel | Male | -5.564728043 | -6.043025789 | -5.083995473 |
| Israel | Female | -6.844823158 | -7.719220921 | -5.962140122 |
| Vanuatu | Both | 0.419372952 | -0.399236144 | 1.244710117 |
| Vanuatu | Male | 0.444708967 | -0.553264732 | 1.45269759 |
| Vanuatu | Female | 0.561057131 | -1.019054267 | 2.166393101 |
| Venezuela (Bolivarian Republic of) | Both | -1.370636761 | -1.554470267 | -1.186459971 |
| Venezuela (Bolivarian Republic of) | Male | -1.22805105 | -1.407692814 | -1.048081967 |
| Venezuela (Bolivarian Republic of) | Female | -1.663140812 | -1.873696017 | -1.452133806 |
| Kenya | Both | 1.295516218 | 1.101960333 | 1.489442658 |
| Kenya | Male | 1.383107999 | 1.181099696 | 1.585519611 |
| Kenya | Female | 1.21719155 | 0.930298501 | 1.504900088 |
| Denmark | Both | -6.000669987 | -6.818189679 | -5.175977883 |
| Denmark | Male | -5.96259885 | -6.96911656 | -4.945191445 |
| Denmark | Female | -6.333246517 | -7.878801928 | -4.761760685 |
| Nigeria | Both | -1.153196394 | -1.239563494 | -1.066753766 |
| Nigeria | Male | -0.980907832 | -1.065450551 | -0.896292869 |
| Nigeria | Female | -1.110319138 | -1.222358045 | -0.99815315 |
| Brunei Darussalam | Both | -1.785016167 | -2.655069671 | -0.907186264 |
| Brunei Darussalam | Male | -1.626456643 | -2.993379917 | -0.240272011 |
| Brunei Darussalam | Female | -2.173722112 | -3.891847474 | -0.424881824 |
| American Samoa | Both | 0.110664371 | -2.173223 | 2.447871924 |
| American Samoa | Male | -0.128359273 | -2.873424985 | 2.69428959 |
| American Samoa | Female | 1.107982689 | -3.46989837 | 5.902966958 |
| Botswana | Both | -0.505599135 | -0.990237093 | -0.018588947 |
| Botswana | Male | -1.022721832 | -1.660691168 | -0.380613716 |
| Botswana | Female | 0.854204324 | -0.019394243 | 1.735436115 |
| Pakistan | Both | 0.871049232 | 0.80087081 | 0.941276512 |
| Pakistan | Male | 1.303962698 | 1.225210734 | 1.382775931 |
| Pakistan | Female | 0.229172547 | 0.166897827 | 0.291485983 |
| Luxembourg | Both | -4.808986859 | -6.237564855 | -3.358642843 |
| Luxembourg | Male | -4.840761427 | -6.479058813 | -3.17376439 |
| Luxembourg | Female | -4.901389476 | -7.909189719 | -1.795350742 |
| Eswatini | Both | 0.807615577 | 0.069405864 | 1.551271046 |
| Eswatini | Male | 0.766744003 | -0.226263935 | 1.76963495 |
| Eswatini | Female | 0.674895276 | -0.608263908 | 1.974620197 |
| Nepal | Both | -0.069708281 | -0.182272376 | 0.042982752 |
| Nepal | Male | 0.627285862 | 0.482475304 | 0.772305115 |
| Nepal | Female | -1.13288743 | -1.316901427 | -0.948530304 |
| Finland | Both | -4.057180337 | -4.677301562 | -3.433024918 |
| Finland | Male | -4.050253779 | -4.753109692 | -3.342211277 |
| Finland | Female | -4.279671677 | -5.6673934 | -2.871535256 |
| Guyana | Both | -1.426134907 | -1.945826139 | -0.903689289 |
| Guyana | Male | -1.343223836 | -1.998287105 | -0.683781991 |
| Guyana | Female | -1.567592915 | -2.451147707 | -0.676035271 |
| Tajikistan | Both | 0.515134955 | 0.13313775 | 0.898589439 |
| Tajikistan | Male | 0.593425908 | 0.333324305 | 0.854201792 |
| Tajikistan | Female | 0.413358129 | -0.137861062 | 0.967619941 |
| Honduras | Both | -0.493951957 | -0.741227213 | -0.246060684 |
| Honduras | Male | -0.524644741 | -0.862085589 | -0.186055329 |
| Honduras | Female | -0.437846687 | -0.804024543 | -0.0703171 |
| Palestine | Both | -2.088407392 | -2.377056755 | -1.798904556 |
| Palestine | Male | -2.168296813 | -2.523283102 | -1.812017751 |
| Palestine | Female | -2.138578261 | -2.659544168 | -1.614824146 |
| Niger | Both | -0.460577638 | -0.692807668 | -0.227804538 |
| Niger | Male | -0.487200204 | -0.801377393 | -0.172027968 |
| Niger | Female | -0.436391351 | -0.799130049 | -0.07232626 |
| Suriname | Both | -2.072659726 | -2.862267548 | -1.276633384 |
| Suriname | Male | -1.864761149 | -2.841937428 | -0.877756829 |
| Suriname | Female | -2.458915437 | -3.842531679 | -1.055390248 |
| Guinea-Bissau | Both | 0.157261019 | -0.355844529 | 0.673008741 |
| Guinea-Bissau | Male | -0.100336101 | -0.788040668 | 0.592135407 |
| Guinea-Bissau | Female | 0.54081919 | -0.262614797 | 1.350725236 |
| Montenegro | Both | -1.061905845 | -1.997717255 | -0.11715849 |
| Montenegro | Male | -1.245973355 | -2.338255318 | -0.141474942 |
| Montenegro | Female | -0.636650927 | -2.578853266 | 1.344271444 |
| Australia | Both | -4.344700264 | -4.696720167 | -3.991380113 |
| Australia | Male | -4.195906868 | -4.515751348 | -3.874991002 |
| Australia | Female | -4.754059262 | -5.30144683 | -4.203507621 |
| Indonesia | Both | 0.419454499 | 0.367801467 | 0.471134114 |
| Indonesia | Male | 0.758392451 | 0.699021296 | 0.817798611 |
| Indonesia | Female | -0.108907337 | -0.178450008 | -0.039316218 |
| Zimbabwe | Both | 1.511760472 | 1.278701666 | 1.745355584 |
| Zimbabwe | Male | 0.751767905 | 0.459390495 | 1.044996251 |
| Zimbabwe | Female | 3.002963391 | 2.565929118 | 3.441859871 |
| Kuwait | Both | -1.914087475 | -2.215999722 | -1.611243061 |
| Kuwait | Male | -1.063875519 | -1.412106368 | -0.714414654 |
| Kuwait | Female | -4.577427898 | -5.295710954 | -3.853697036 |
| Fiji | Both | -0.830400143 | -1.241291629 | -0.417799119 |
| Fiji | Male | -0.832892381 | -1.35594378 | -0.307067548 |
| Fiji | Female | -0.678944927 | -1.497094193 | 0.145999754 |
| Kiribati | Both | -0.343958351 | -1.592239044 | 0.920156507 |
| Kiribati | Male | -0.464062119 | -2.271712804 | 1.377024137 |
| Kiribati | Female | -0.096821348 | -2.153270753 | 2.002848553 |
| Antigua and Barbuda | Both | -2.497955274 | -5.287109099 | 0.373334984 |
| Antigua and Barbuda | Male | -2.816519391 | -6.211105293 | 0.700929815 |
| Antigua and Barbuda | Female | -2.179119354 | -7.209691076 | 3.124181838 |
| Hungary | Both | -3.186533504 | -3.49778934 | -2.874273751 |
| Hungary | Male | -3.270109346 | -3.579272193 | -2.959955201 |
| Hungary | Female | -3.085477359 | -3.625878169 | -2.542046347 |
| Trinidad and Tobago | Both | -2.897974393 | -3.446483005 | -2.346349771 |
| Trinidad and Tobago | Male | -2.673628971 | -3.382880969 | -1.959170459 |
| Trinidad and Tobago | Female | -3.477981242 | -4.424450006 | -2.522139755 |
| Marshall Islands | Both | 0.406168922 | -1.365142611 | 2.209290148 |
| Marshall Islands | Male | 0.140388649 | -2.253596237 | 2.593006522 |
| Marshall Islands | Female | 0.86765929 | -2.269078204 | 4.105072415 |
| Slovakia | Both | -3.27763827 | -3.690211559 | -2.863297593 |
| Slovakia | Male | -3.322259666 | -3.74493675 | -2.897726513 |
| Slovakia | Female | -3.292435402 | -3.992166256 | -2.587604722 |
| Cambodia | Both | -0.807081114 | -0.969768717 | -0.644126246 |
| Cambodia | Male | -0.526220198 | -0.739556416 | -0.312425465 |
| Cambodia | Female | -1.352667466 | -1.61496718 | -1.089668447 |
| Malawi | Both | -0.634618482 | -0.830879985 | -0.437968566 |
| Malawi | Male | -0.063224982 | -0.317036831 | 0.191233121 |
| Malawi | Female | -1.639795845 | -1.980471296 | -1.297936347 |
| Oman | Both | -2.599967148 | -3.030391063 | -2.16763269 |
| Oman | Male | -2.766185109 | -3.319050436 | -2.210158249 |
| Oman | Female | -2.272358583 | -2.751511717 | -1.790844613 |
| Slovenia | Both | -4.448397803 | -5.612582326 | -3.269854104 |
| Slovenia | Male | -4.243235402 | -5.598131068 | -2.868893699 |
| Slovenia | Female | -5.41191291 | -7.834410322 | -2.925742128 |
| Russian Federation | Both | -1.821844659 | -2.190466764 | -1.4518333 |
| Russian Federation | Male | -1.920425761 | -2.221042135 | -1.618885158 |
| Russian Federation | Female | -1.738906582 | -2.280547207 | -1.194263745 |
| Italy | Both | -3.355941068 | -3.658969799 | -3.051959198 |
| Italy | Male | -3.278718062 | -3.49309495 | -3.063864965 |
| Italy | Female | -3.649236686 | -4.186158332 | -3.109306238 |
| Turkmenistan | Both | -1.652306219 | -1.842995346 | -1.461246641 |
| Turkmenistan | Male | -1.656949214 | -1.870014008 | -1.443421804 |
| Turkmenistan | Female | -1.69150152 | -1.982556673 | -1.399582102 |
| Guam | Both | 0.274582005 | -0.881787133 | 1.444442 |
| Guam | Male | 0.484163834 | -0.926944734 | 1.915370979 |
| Guam | Female | 0.191355166 | -2.019027465 | 2.451602493 |
| Sao Tome and Principe | Both | 0.49392574 | -1.371956316 | 2.395107248 |
| Sao Tome and Principe | Male | 0.752154242 | -1.702071951 | 3.267655646 |
| Sao Tome and Principe | Female | 0.060541149 | -2.914015237 | 3.126233097 |
| Philippines | Both | 3.237243603 | 2.996520787 | 3.478529034 |
| Philippines | Male | 3.210192078 | 2.992934781 | 3.427907666 |
| Philippines | Female | 3.378382663 | 3.049613097 | 3.708201135 |
| New Zealand | Both | -4.184038594 | -4.742113615 | -3.622694052 |
| New Zealand | Male | -4.053856082 | -4.683373546 | -3.420180977 |
| New Zealand | Female | -4.525172573 | -5.751351224 | -3.283041288 |
| Senegal | Both | -0.439064783 | -0.67081339 | -0.206775475 |
| Senegal | Male | -0.583306689 | -0.879684375 | -0.28604281 |
| Senegal | Female | -0.289270244 | -0.668527103 | 0.091434652 |
| Netherlands | Both | -5.272098617 | -5.806343817 | -4.734823299 |
| Netherlands | Male | -5.346917778 | -5.788177349 | -4.903591482 |
| Netherlands | Female | -5.259395994 | -6.016808513 | -4.495879472 |
| Argentina | Both | -2.794698702 | -2.998695478 | -2.590272915 |
| Argentina | Male | -2.789692711 | -2.96538821 | -2.613679088 |
| Argentina | Female | -2.723176059 | -3.018875363 | -2.426575156 |
| Mongolia | Both | -1.944698286 | -2.179257163 | -1.709576974 |
| Mongolia | Male | -1.195275022 | -1.497735947 | -0.89188536 |
| Mongolia | Female | -3.300252612 | -3.652963283 | -2.946250726 |
| Angola | Both | -0.872608196 | -1.048676473 | -0.696226634 |
| Angola | Male | -0.786601807 | -1.023629558 | -0.549006424 |
| Angola | Female | -0.885683625 | -1.191343293 | -0.579078415 |
| Monaco | Both | -2.444616794 | -8.031882878 | 3.482088035 |
| Monaco | Male | -2.618606661 | -9.048855208 | 4.266260642 |
| Monaco | Female | -2.306573178 | -13.60129205 | 10.46468022 |
| Mozambique | Both | 1.705063021 | 1.514669497 | 1.895813632 |
| Mozambique | Male | 2.282006531 | 2.034197452 | 2.530417461 |
| Mozambique | Female | 0.680031718 | 0.358194451 | 1.002901081 |
| Nauru | Both | 0.221870922 | -3.171447434 | 3.734106778 |
| Nauru | Male | 0.250225166 | -4.034533061 | 4.72629339 |
| Nauru | Female | 0.632399491 | -5.294922244 | 6.930695452 |
| Thailand | Both | -2.375754913 | -2.57003903 | -2.181083376 |
| Thailand | Male | -2.156296449 | -2.351174823 | -1.961029156 |
| Thailand | Female | -3.143482492 | -3.466947584 | -2.818933526 |
| Benin | Both | -0.406835635 | -0.681863478 | -0.131046195 |
| Benin | Male | -0.638834803 | -0.983239717 | -0.293231963 |
| Benin | Female | -0.084420445 | -0.554616526 | 0.38799881 |
| Sweden | Both | -4.047849481 | -4.534533023 | -3.558684822 |
| Sweden | Male | -4.127714563 | -4.691178116 | -3.560919827 |
| Sweden | Female | -4.08378006 | -5.059679277 | -3.097849495 |
| Brazil | Both | -2.21548084 | -2.298658214 | -2.132232653 |
| Brazil | Male | -1.989905033 | -2.048990253 | -1.930784172 |
| Brazil | Female | -2.564346704 | -2.703911509 | -2.424581703 |
| Malta | Both | -3.0478882 | -4.257604387 | -1.82288711 |
| Malta | Male | -2.946916381 | -4.352301079 | -1.52088188 |
| Malta | Female | -3.54153042 | -5.994309646 | -1.024753728 |
| Saudi Arabia | Both | -0.131819027 | -0.282033096 | 0.018621324 |
| Saudi Arabia | Male | 0.185122815 | 0.036257059 | 0.334210101 |
| Saudi Arabia | Female | -0.703674545 | -0.882319167 | -0.524707943 |
| Sierra Leone | Both | 0.225794101 | -0.074137072 | 0.526625528 |
| Sierra Leone | Male | -0.256002929 | -0.655350425 | 0.14494987 |
| Sierra Leone | Female | 0.785450321 | 0.32787136 | 1.245116225 |
| Myanmar | Both | -2.075243223 | -2.159402808 | -1.991011246 |
| Myanmar | Male | -0.852855301 | -0.956538976 | -0.749063085 |
| Myanmar | Female | -3.353042886 | -3.470737086 | -3.235205186 |
| Austria | Both | -3.861592412 | -4.405107223 | -3.314987389 |
| Austria | Male | -3.878316182 | -4.338114074 | -3.416308276 |
| Austria | Female | -4.204209695 | -5.077680627 | -3.322701123 |
| Papua New Guinea | Both | 0.972683749 | 0.754404827 | 1.191435561 |
| Papua New Guinea | Male | 0.79460785 | 0.521951334 | 1.068003921 |
| Papua New Guinea | Female | 1.28871666 | 0.887771852 | 1.691254889 |
| Burkina Faso | Both | 0.696997289 | 0.495680767 | 0.898717095 |
| Burkina Faso | Male | 0.659217862 | 0.396645922 | 0.922476518 |
| Burkina Faso | Female | 0.776697694 | 0.454525815 | 1.099902823 |
| United Kingdom | Both | -4.053573469 | -4.451798663 | -3.653688553 |
| United Kingdom | Male | -3.984576309 | -4.346202828 | -3.621582633 |
| United Kingdom | Female | -4.377951546 | -4.912137656 | -3.840764475 |
| Paraguay | Both | -0.680047076 | -0.976480268 | -0.382726492 |
| Paraguay | Male | -0.231132467 | -0.594157346 | 0.133218159 |
| Paraguay | Female | -1.558513313 | -2.086692839 | -1.027484596 |
| Albania | Both | -0.024082239 | -0.321735446 | 0.274459803 |
| Albania | Male | -0.054093208 | -0.404162974 | 0.297207019 |
| Albania | Female | -0.133389018 | -0.720037546 | 0.456726036 |
| Andorra | Both | -2.301763278 | -7.037430245 | 2.675146392 |
| Andorra | Male | -2.28275555 | -8.015421756 | 3.807182086 |
| Andorra | Female | -2.051710798 | -10.70714772 | 7.442724843 |
| Togo | Both | -0.008781483 | -0.30418441 | 0.287496736 |
| Togo | Male | 0.210811689 | -0.170148769 | 0.593225929 |
| Togo | Female | -0.238964878 | -0.725522421 | 0.24997735 |
| Qatar | Both | -3.688775151 | -4.281738395 | -3.092138571 |
| Qatar | Male | -4.052984705 | -4.789594273 | -3.310676246 |
| Qatar | Female | -3.528107531 | -4.802154611 | -2.237009686 |
| Mauritius | Both | -4.080301932 | -4.518706314 | -3.639884607 |
| Mauritius | Male | -3.827019086 | -4.340884332 | -3.310393439 |
| Mauritius | Female | -4.833073947 | -5.745065259 | -3.912258395 |
| Samoa | Both | 0.091088972 | -0.913862585 | 1.10623295 |
| Samoa | Male | -0.251849269 | -1.494641358 | 1.006622496 |
| Samoa | Female | 0.74369735 | -1.090761831 | 2.612180051 |
| Lao People's Democratic Republic | Both | -0.529219334 | -0.735827466 | -0.322181168 |
| Lao People's Democratic Republic | Male | -0.482142883 | -0.761011612 | -0.202490513 |
| Lao People's Democratic Republic | Female | -0.725277493 | -1.061073324 | -0.38834198 |
| Bahamas | Both | -1.588415941 | -2.639654083 | -0.525827165 |
| Bahamas | Male | -1.583046936 | -2.85644081 | -0.292960942 |
| Bahamas | Female | -1.905626617 | -3.8511543 | 0.079267924 |
| Norway | Both | -5.414341101 | -6.048239606 | -4.776165642 |
| Norway | Male | -5.526453425 | -6.254574188 | -4.792677348 |
| Norway | Female | -5.369260063 | -6.69366439 | -4.02605694 |
| Niue | Both | -0.594475794 | -9.471675648 | 9.153221529 |
| Niue | Male | -0.682245572 | -10.97189841 | 10.79666048 |
| Niue | Female | -0.24577967 | -17.82548511 | 21.094776 |
| Rwanda | Both | -2.427417969 | -2.681862793 | -2.172307883 |
| Rwanda | Male | -2.664027883 | -2.991576758 | -2.33537304 |
| Rwanda | Female | -2.14207642 | -2.584543789 | -1.697599334 |
| Latvia | Both | -3.555742871 | -4.398769587 | -2.705282212 |
| Latvia | Male | -3.554512409 | -4.313637774 | -2.789364542 |
| Latvia | Female | -3.768008282 | -5.263516425 | -2.248892079 |
| Cabo Verde | Both | -0.690356493 | -1.740169901 | 0.370673178 |
| Cabo Verde | Male | -1.177389164 | -2.441702564 | 0.103309191 |
| Cabo Verde | Female | -0.59868043 | -2.539278307 | 1.380557835 |
| Cameroon | Both | 0.595892074 | 0.409734019 | 0.782395264 |
| Cameroon | Male | 0.648030503 | 0.410896836 | 0.885724194 |
| Cameroon | Female | 0.479109641 | 0.170101513 | 0.789071008 |
| Micronesia (Federated States of) | Both | 0.202119159 | -0.940592247 | 1.358012446 |
| Micronesia (Federated States of) | Male | 0.111802278 | -1.360132863 | 1.605702099 |
| Micronesia (Federated States of) | Female | 0.37737383 | -1.755510889 | 2.556563407 |
| Estonia | Both | -5.308861572 | -6.240346208 | -4.368122812 |
| Estonia | Male | -5.245273654 | -6.441569148 | -4.033681591 |
| Estonia | Female | -5.701588675 | -7.43658893 | -3.934067732 |
| Central African Republic | Both | -0.046240709 | -0.359957372 | 0.26846369 |
| Central African Republic | Male | -0.300102473 | -0.773741695 | 0.175797583 |
| Central African Republic | Female | 0.324343438 | -0.221992146 | 0.873670489 |
| Lithuania | Both | -2.358912215 | -3.074006398 | -1.638542257 |
| Lithuania | Male | -2.160746453 | -2.733570769 | -1.584548643 |
| Lithuania | Female | -2.890710535 | -3.841949448 | -1.930061536 |
| Northern Mariana Islands | Both | 0.782068963 | -2.136160537 | 3.787317974 |
| Northern Mariana Islands | Male | 1.273349675 | -2.35270409 | 5.03405403 |
| Northern Mariana Islands | Female | 0.264510567 | -5.143404067 | 5.980738402 |
| Barbados | Both | -2.768478929 | -4.41612635 | -1.092429834 |
| Barbados | Male | -3.165304281 | -5.203099203 | -1.083704044 |
| Barbados | Female | -2.29136057 | -5.169341934 | 0.67396361 |
| Bahrain | Both | -5.135733966 | -5.658656289 | -4.609913151 |
| Bahrain | Male | -5.309000505 | -5.997263959 | -4.615697765 |
| Bahrain | Female | -5.030832836 | -6.132728354 | -3.916002312 |
| Algeria | Both | -2.546698106 | -2.874069963 | -2.218222812 |
| Algeria | Male | -2.66340417 | -2.907313866 | -2.41888174 |
| Algeria | Female | -2.452070432 | -2.925314111 | -1.976519668 |
| Congo | Both | -1.414777328 | -1.74643553 | -1.081999604 |
| Congo | Male | -2.139320864 | -2.642820251 | -1.633217543 |
| Congo | Female | -0.561974972 | -1.092468832 | -0.028635791 |
| Malaysia | Both | -0.317796431 | -0.518165644 | -0.117023649 |
| Malaysia | Male | -0.249722646 | -0.475595891 | -0.023336776 |
| Malaysia | Female | -0.859152917 | -1.131721757 | -0.585832635 |
| Uzbekistan | Both | 1.984063394 | 1.795740489 | 2.172734698 |
| Uzbekistan | Male | 1.956596845 | 1.691890592 | 2.221992135 |
| Uzbekistan | Female | 1.953792104 | 1.66380359 | 2.244607789 |
| Syrian Arab Republic | Both | -1.318196766 | -1.446441892 | -1.189784758 |
| Syrian Arab Republic | Male | -1.211529828 | -1.330963686 | -1.091951402 |
| Syrian Arab Republic | Female | -1.345991529 | -1.520592746 | -1.171080749 |
| Belgium | Both | -4.114997679 | -4.451083524 | -3.777729678 |
| Belgium | Male | -4.107742956 | -4.516495952 | -3.69724014 |
| Belgium | Female | -4.133866972 | -4.749297492 | -3.514460055 |
| Ecuador | Both | -1.02137833 | -1.254619044 | -0.787586691 |
| Ecuador | Male | -0.585008979 | -0.802448144 | -0.367093191 |
| Ecuador | Female | -1.775650024 | -2.064420219 | -1.486028369 |
| Belarus | Both | -0.885458133 | -1.55886429 | -0.207445407 |
| Belarus | Male | -0.602933522 | -1.014764846 | -0.18938876 |
| Belarus | Female | -1.282876838 | -2.370785494 | -0.182845322 |
| Somalia | Both | 0.590007356 | 0.380979271 | 0.799470709 |
| Somalia | Male | 0.3282628 | 0.053439088 | 0.60384139 |
| Somalia | Female | 1.089768684 | 0.732796893 | 1.448005492 |
| Peru | Both | -2.777875009 | -2.91039654 | -2.645172594 |
| Peru | Male | -2.427328024 | -2.590532967 | -2.263849639 |
| Peru | Female | -3.406687771 | -3.637541704 | -3.175280785 |
| Democratic Republic of the Congo | Both | -0.539543724 | -0.636903715 | -0.442088334 |
| Democratic Republic of the Congo | Male | -0.725242461 | -0.854270084 | -0.596046923 |
| Democratic Republic of the Congo | Female | -0.510457526 | -0.67288182 | -0.347767628 |
| Spain | Both | -3.611334503 | -3.951077626 | -3.270389645 |
| Spain | Male | -3.488481208 | -3.745299059 | -3.230978139 |
| Spain | Female | -3.866444354 | -4.325586025 | -3.405099263 |
| Tonga | Both | 0.07459429 | -1.767959059 | 1.951708692 |
| Tonga | Male | 0.164637349 | -1.919578505 | 2.293142937 |
| Tonga | Female | -0.249818623 | -4.409346758 | 4.090707065 |
| Gabon | Both | -0.860591321 | -1.419515049 | -0.298498651 |
| Gabon | Male | -0.874155494 | -1.577431815 | -0.165853928 |
| Gabon | Female | -0.910218814 | -2.046563959 | 0.239308924 |
| Turkey | Both | -3.63115356 | -3.987297494 | -3.273688567 |
| Turkey | Male | -3.644012181 | -3.975177637 | -3.311704619 |
| Turkey | Female | -3.552668394 | -4.162043214 | -2.939418934 |
| Belize | Both | -2.184713124 | -3.427393437 | -0.926042207 |
| Belize | Male | -1.913896895 | -3.430034108 | -0.373956504 |
| Belize | Female | -2.601998773 | -4.813161842 | -0.339471017 |
| United Republic of Tanzania | Both | 0.062921241 | -0.071322613 | 0.197345438 |
| United Republic of Tanzania | Male | -0.176945691 | -0.341465883 | -0.012153902 |
| United Republic of Tanzania | Female | 0.83924289 | 0.587335237 | 1.091781412 |
| Gambia | Both | 0.46973665 | -0.144762102 | 1.088016963 |
| Gambia | Male | 0.251050315 | -0.532937424 | 1.041217354 |
| Gambia | Female | 0.944858042 | -0.104874568 | 2.005621607 |
| Portugal | Both | -4.59292836 | -4.978405238 | -4.205887707 |
| Portugal | Male | -4.423847425 | -4.864424936 | -3.981229578 |
| Portugal | Female | -5.049344182 | -5.86333222 | -4.228317691 |
| Bosnia and Herzegovina | Both | -2.493685197 | -2.821978843 | -2.164282487 |
| Bosnia and Herzegovina | Male | -2.417201741 | -2.804190814 | -2.028671856 |
| Bosnia and Herzegovina | Female | -2.772109239 | -3.410891605 | -2.12910235 |
| Uganda | Both | -0.106674888 | -0.274879098 | 0.061813028 |
| Uganda | Male | -0.209247732 | -0.414510176 | -0.003562208 |
| Uganda | Female | 0.365154952 | 0.034359744 | 0.697044039 |
| Republic of Moldova | Both | -1.347093957 | -1.896026663 | -0.795089743 |
| Republic of Moldova | Male | -0.943099522 | -1.434243063 | -0.44950866 |
| Republic of Moldova | Female | -2.470970584 | -3.320493449 | -1.613982962 |
| Chad | Both | 0.297812277 | 0.040035344 | 0.556253433 |
| Chad | Male | 0.060991605 | -0.265621086 | 0.388673895 |
| Chad | Female | 0.500876193 | 0.074521926 | 0.929046886 |
| Saint Kitts and Nevis | Both | -3.83218371 | -6.693226085 | -0.883413905 |
| Saint Kitts and Nevis | Male | -3.691873116 | -7.075975827 | -0.184528312 |
| Saint Kitts and Nevis | Female | -4.633736654 | -10.40426753 | 1.508452849 |
| United Arab Emirates | Both | -1.530179884 | -1.901963717 | -1.156987018 |
| United Arab Emirates | Male | -1.742344008 | -2.185000972 | -1.297683822 |
| United Arab Emirates | Female | -1.118649089 | -1.853924653 | -0.377865102 |
| Bulgaria | Both | -2.310350348 | -2.689899915 | -1.92932038 |
| Bulgaria | Male | -2.17382337 | -2.455582724 | -1.891250148 |
| Bulgaria | Female | -2.628783693 | -3.198821873 | -2.0553887 |
| Solomon Islands | Both | 0.242442251 | -0.298809403 | 0.78663222 |
| Solomon Islands | Male | 0.310977819 | -0.368141271 | 0.994725979 |
| Solomon Islands | Female | 0.254932274 | -0.648360891 | 1.166438072 |
| Seychelles | Both | -1.64737962 | -3.398700935 | 0.135692059 |
| Seychelles | Male | -2.086513558 | -4.163532521 | 0.035519666 |
| Seychelles | Female | -1.200651179 | -4.741586815 | 2.471907742 |
| Cuba | Both | -2.874564658 | -3.10756887 | -2.641000125 |
| Cuba | Male | -2.656919074 | -2.934567436 | -2.37847652 |
| Cuba | Female | -3.367559446 | -3.810899849 | -2.922175665 |
| Puerto Rico | Both | -3.486030204 | -3.945257617 | -3.024607275 |
| Puerto Rico | Male | -3.225972028 | -3.762779417 | -2.686170348 |
| Puerto Rico | Female | -4.024131426 | -4.920762331 | -3.119044975 |
| Equatorial Guinea | Both | -2.895879109 | -3.752854807 | -2.031272977 |
| Equatorial Guinea | Male | -3.657550071 | -4.836506467 | -2.463987884 |
| Equatorial Guinea | Female | -2.088267955 | -3.557601603 | -0.596548496 |
| Côte d'Ivoire | Both | -0.808329471 | -0.968158989 | -0.648242001 |
| Côte d'Ivoire | Male | -1.180267869 | -1.378063782 | -0.982075257 |
| Côte d'Ivoire | Female | 0.019510372 | -0.272590445 | 0.31246675 |
| Iran (Islamic Republic of) | Both | -2.803252013 | -2.893664539 | -2.712755307 |
| Iran (Islamic Republic of) | Male | -2.520352917 | -2.610941577 | -2.429679993 |
| Iran (Islamic Republic of) | Female | -3.313161363 | -3.423333286 | -3.202863758 |
| Tunisia | Both | -1.248136454 | -1.397374167 | -1.098672866 |
| Tunisia | Male | -1.075847488 | -1.263665922 | -0.887671782 |
| Tunisia | Female | -1.500768149 | -1.756380791 | -1.244490447 |
| Dominica | Both | -2.125970366 | -5.084860586 | 0.925160475 |
| Dominica | Male | -2.265854044 | -6.045881421 | 1.666254022 |
| Dominica | Female | -2.289495177 | -7.095650509 | 2.765293607 |
| Colombia | Both | -2.733182283 | -2.834767541 | -2.63149082 |
| Colombia | Male | -2.396491074 | -2.521867924 | -2.270952963 |
| Colombia | Female | -3.217515923 | -3.394792635 | -3.039913896 |
| Palau | Both | -0.110758221 | -2.915925966 | 2.775462634 |
| Palau | Male | -0.186150606 | -3.587935632 | 3.335662359 |
| Palau | Female | -0.206388008 | -5.999709764 | 5.943981337 |
| Costa Rica | Both | -2.152182888 | -2.531879149 | -1.771007484 |
| Costa Rica | Male | -1.677706974 | -2.127629973 | -1.225715663 |
| Costa Rica | Female | -3.005189796 | -3.729238272 | -2.27569578 |
| Cyprus | Both | -3.418240465 | -4.114335079 | -2.717092461 |
| Cyprus | Male | -2.922630553 | -3.666768873 | -2.172744042 |
| Cyprus | Female | -4.728896465 | -6.626822351 | -2.792392876 |
| Dominican Republic | Both | 1.505047022 | 1.327994646 | 1.682408766 |
| Dominican Republic | Male | 1.842756551 | 1.641335845 | 2.044576407 |
| Dominican Republic | Female | 0.944434839 | 0.622636419 | 1.267262392 |
| Bolivia (Plurinational State of) | Both | -1.662900707 | -1.878075391 | -1.44725416 |
| Bolivia (Plurinational State of) | Male | -1.533864486 | -1.818984046 | -1.247916933 |
| Bolivia (Plurinational State of) | Female | -1.896103392 | -2.228125604 | -1.562953671 |
| Switzerland | Both | -4.641333549 | -5.132621501 | -4.147501372 |
| Switzerland | Male | -4.637138278 | -5.194525307 | -4.076474221 |
| Switzerland | Female | -4.848419761 | -5.931944362 | -3.752414562 |
| Egypt | Both | -0.557032253 | -0.694649595 | -0.419224201 |
| Egypt | Male | -0.608695445 | -0.737029819 | -0.480195152 |
| Egypt | Female | -0.492850247 | -0.68328026 | -0.302055104 |
| Zambia | Both | -1.04175356 | -1.24475062 | -0.838339228 |
| Zambia | Male | -0.749613154 | -1.015071534 | -0.483442866 |
| Zambia | Female | -1.438562428 | -1.762844562 | -1.113209833 |
| Ghana | Both | -0.094281826 | -0.239944116 | 0.051593148 |
| Ghana | Male | 0.224496961 | 0.016663474 | 0.432762324 |
| Ghana | Female | -0.415219957 | -0.623329253 | -0.206674849 |
| Yemen | Both | -1.047509943 | -1.140358646 | -0.954574037 |
| Yemen | Male | -1.08563045 | -1.213717875 | -0.957376944 |
| Yemen | Female | -1.017375542 | -1.165127389 | -0.869402816 |

Abbreviations: CI, confidence interval.
